# Anxiety is associated with systemic pro-inflammatory profile and plasma lipid changes in Mexican young adults

**DOI:** 10.3389/fncel.2026.1777048

**Published:** 2026-02-27

**Authors:** Sofía Bernal-Vega, Natalia Vázquez-Manjarrez, Mariana Villegas-Romero, Rocío Ortiz-López, José Alfonso Ontiveros-Sánchez de la Barquera, Antonio Alí Pérez-Maya, Alberto Camacho-Morales

**Affiliations:** 1 Department of Biochemistry and Molecular Medicine, College of Medicine, Universidad Autónoma de Nuevo León, Monterrey, Mexico; 2 Neurometabolism Unit, Center for Research and Development in Health Sciences, Universidad Autónoma de Nuevo León, Monterrey, Mexico; 3 Department of Nutrition Physiology, National Institute of Medical Sciences and Nutrition Salvador Zubirán, Mexico City, Mexico; 4 The Institute for Obesity Research, Tecnológico de Monterrey, Escuela de Medicina y Ciencias de la Salud, Monterrey, Mexico; 5 Department of Psychiatry, Hospital Universitario “Dr. José Eleuterio González”, College of Medicine, Universidad Autónoma de Nuevo León, Monterrey, Mexico

**Keywords:** anxiety, ceramides, inflammation, lipids, nutrition

## Abstract

**Introduction:**

Systemic inflammation and altered lipid metabolism have been implicated in anxiety disorders, but the relationship between circulating cytokines, plasma lipid species and symptom severity remains unclear.

**Methods:**

We studied 34 young adults (17 healthy controls, 17 with clinician-confirmed anxiety; age 21–27 years). Anxiety severity was assessed with standardized clinical instruments. Plasma cytokines were quantified by multiplex immunoassay and targeted lipid profiling was performed by mass spectrometry. Group comparisons used nonparametric tests (Mann-Whitney U), and associations were examined with Spearman correlations and robust regression models appropriate for nonparametric data.

**Results:**

Compared with controls, individuals with anxiety showed elevated plasma IL-6, MCP-1 and TNF-α and reduced IL-17A. Targeted lipidomics detected no group differences in total ceramides, dihydroceramides, hexosylceramides or lactosylceramides; nominal reductions were observed for DG 18:2/22:4, DG 18:2/22:6, LPC 24:1 and TG 60:11 (FA 22:5). None of the lipid species correlated with anxiety severity, and combined lipid-cytokine regression models failed to identify independent lipid predictors. MCP-1 and TNF-α showed associations with anxiety severity that were restricted to the clinical group. (All results are presented as unadjusted values; lipid findings did not remain significant after correction for multiple testing.)

**Discussion:**

The data indicate a reproducible pro-inflammatory signal in clinical anxiety and point to modest, exploratory alterations in selected glycerolipids and lysophospholipids that require validation. These results should be considered hypothesis-generating; larger, longitudinal studies are needed to determine causality and the clinical relevance of immunometabolic signatures in anxiety.

## 1 Introduction

Anxiety disorders are among the most common psychiatric conditions worldwide, affecting up to 4.4% of the global population according to the World Health Organization. Anxiety disorders are defined by persistent and disproportionate fear, excessive worry, and sustained hypervigilance, which collectively impair daily functioning and reduce quality of life. Epidemiological data indicate that anxiety onset commonly occurs during adolescence or early adulthood, exhibiting a high prevalence in women (Remes et al., 2016). Accordingly, anxiety substantially affects socioeconomic stability, driven by reduced productivity, increased healthcare use, and high rates of comorbidity with depression and other chronic medical disorders (Kalin, 2020; Stein et al., 2017).

Although the biological traits underlying anxiety disorders remain incompletely understood, clinical and preclinical evidence suggest that neuroendocrine dysregulation, immune activation, mitochondrial dysfunction, and alterations in lipid metabolism might contribute to pathophysiology (Grogans et al., 2023; Mishra and Varma, 2023). Based on this context, elevated circulating levels of IL-6, TNF-α, and IL-1β have been reported in Chinese individuals diagnosed with generalized anxiety disorder compared with healthy controls, reflecting heightened immune system activation (Tang et al., 2018). IL-6 concentrations also positively correlate with Hamilton Anxiety Rating Scale (HAM-A) scores in anxious Chinese patients (Zou et al., 2020). Beyond primary psychiatric cohorts, increased IL-6, TNF-α, and IL-17A levels have been observed in Chinese patients with rheumatoid arthritis presenting comorbid anxiety symptoms (Liu et al., 2012). Consistent with these findings, IL-17A concentrations positively correlate with State-Trait Anxiety Inventory scores in postpartum women with depression and anxiety of an Asian cohort (Min et al., 2022), and also, TNF-α and IL-17A levels in Chinese individuals with coronary artery disease are associated with higher Hospital Anxiety and Depression Scale scores (Lu et al., 2022). Furthermore, the MCP1 chemokine has been linked to anxiety in pregnant Mexican women during the third trimester (Camacho-Arroyo et al., 2021) and in patients with alcohol use disorder in clinical psychiatric patients from Pakistan (Kazmi et al., 2022). Collectively, these data indicate that systemic immune activation, reflected by altered cytokine and chemokine profiles, may contribute to the pathophysiology of anxiety disorders and underscore the potential relevance of immunological biomarkers in clinical assessment and therapeutic strategies.

Moreover, evidence supporting the role of systemic inflammation in anxiety has prompted the exploration of novel anti-inflammatory interventions. Approaches that modulate peripheral inflammation range from dietary interventions designed to lower the dietary inflammatory index (Pang et al., 2025) to microbiome-directed treatments such as probiotics, prebiotics, and fecal microbiota transplantation (Peirce and Alviña, 2019; Adıgüzel et al., 2025). These lines of work motivate translational efforts to evaluate whether attenuating systemic inflammation can ameliorate anxiety in defined patient subgroups. Such strategies emphasize the potential relevance of immunological and metabolic biomarkers in clinical assessment and novel therapeutic designs. In parallel, accumulating evidence suggests that lipid metabolism might contribute to the pathophysiology of anxiety disorders. Individuals with depressive and anxious symptoms showed increased concentrations of ceramide species C20:0 and C22:0, together with elevated sphingomyelinase activity, and reduced levels of phosphatidylcholine PC O-36:4 and sphingomyelin SPM 23:1 inversely correlate with depression and anxiety scores in participants from the Netherlands (Demirkan et al., 2013). Also, Alzheimer’s individuals showing anxiety as a comorbidity, reported elevated C14:0, C16:0, C18:0, and C24:1 circulating ceramides compared with cognitively normal individuals without anxiety in a clinical cohort from China (Xing et al., 2016). Similarly, anxious patients diagnosed with Parkinson’s disease showed accumulation of C14:0, C16:0, C18:0, and C24:1 ceramides, whereas a major decrease in fatty acyls, glycerolipids, sterol lipids, and other sphingolipids species relative to both healthy controls without anxiety in a clinical cohort from China (Dong et al., 2021). These observations indicate that alterations in plasma sphingolipid profiles are consistently reported with the manifestation and severity of anxiety symptoms across independent worldwide clinical cohorts.

Therefore, understanding how lipid metabolism interacts with inflammatory pathways could provide novel insights into the molecular basis of anxiety disorders. The current study aimed to determine whether alterations in plasma lipid species are associated with inflammatory profiles and the severity of anxiety symptoms in a Mexican cohort. We aimed to enrich the comprehension of a potential lipid–immune axis contributing to the pathophysiology of anxiety and anxiety-related phenotypes.

## 2 Materials and methods

### 2.1 Subjects

All participants were consecutively recruited from the Psychiatry Department of the Hospital Universitario “Dr. José Eleuterio González.” The study was approved by the Institutional Ethics Research Committee of the Hospital Universitario “Dr. José Eleuterio González” – Universidad Autónoma José Alfonso Ontiveros-Sánchez de la Barquera Nuevo León (UANL), Monterrey, Mexico (Approval ID: BI18-00002). We included 34 individuals–17 patients with clinically diagnosed anxiety disorders and 17 healthy controls. Participants received detailed oral and written information about the study objectives and procedures before providing written informed consent. Inclusion criteria for the control group were age over 18 years, good physical health, and absence of psychiatric diagnosis or anxiety symptoms. Exclusion criteria included poor health status, presence of anxiety symptoms, or inability to provide informed consent. The clinical group comprised adults aged 18 years or older, in good physical condition, evaluated by a certified psychiatrist who confirmed the diagnosis of an anxiety disorder. Individuals with inadequate health status, failure to meet diagnostic criteria, those who were receiving pharmacological treatment at the time of recruitment, or those who showed an inability to sign the informed consent were excluded.

### 2.2 Clinical assessment

Demographic information, medical background, and mental health status were obtained through face-to-face interviews conducted by a trained psychiatrist using a structured questionnaire. Each participant received detailed oral and written information about the study objectives and procedures prior to participation. Written informed consent was obtained before the interview, which lasted approximately 1.5 h. All participants were informed of their right to withdraw or skip any questions at any time without consequence.

The questionnaire included sociodemographic variables such as age, sex, occupation, education level, lifestyle factors, and family history of psychiatric disorders. Medical and psychiatric histories were recorded using a standardized data collection form specifically designed for this study.

To evaluate anxiety symptoms, all participants completed three validated clinical instruments: the Clinical Global Impression Scale (CGI-S), the Generalized Anxiety Disorder 7-item scale (GAD-7), and the Beck Anxiety Inventory (BAI).

The CGI-S provides a clinician-based assessment of illness severity, ranging from 1 = normal (no symptoms) to 7 = among the most extremely ill patients, offering a global measure of clinical impairment. The GAD-7 quantifies self-reported generalized anxiety symptoms, with scores categorized as minimal (0–4), mild (5–9), moderate (10–14), and severe anxiety (≥15). The BAI evaluates the intensity of anxiety based on somatic and cognitive manifestations, where total scores of 0–7 indicate minimal, 8–15 mild, 16–25 moderate, and 26–63 severe anxiety.

Scores from these instruments were subsequently integrated into a composite anxiety index to facilitate comparison between groups and correlation analyses with biochemical parameters. Each clinical scale (BAI, GAD-7, and CGI-S) was transformed into a z-score using the mean and standard deviation of the control group as the reference, thereby anchoring all scores to the distribution of healthy subjects. A composite anxiety score was calculated for each participant as the arithmetic mean of the available z-scores:


$$z=\frac{X-\mu_{\text{control}}}{\sigma_{\text{control}}};\mathrm{Anxiety}\,\mathrm{Composite}+\frac{{\text{z}}_{\text{BAI}}+{\text{z}}_{\mathrm{GAD7}}+{\text{z}}_{\text{CGI}}}{{\text{n}}_{\text{tests}\,\text{available}}}$$

where *n*_*tests available*_ corresponds to the number of non-missing scales. A minimum of two available scales was required to compute the composite score (Andrade, 2021; Bushey et al., 2021; Kroenke et al., 2016).

### 2.3 Cytokines quantification by multiplex technology

Peripheral blood samples were collected immediately after the interview for subsequent analysis. Plasma was isolated from blood samples collected in EDTA tubes by centrifugation at 1000 × *g* for 10 min. A 25 μL aliquot was used for cytokine quantification using a multiplex immunoassay. The analysis was performed according to the manufacturer’s instructions for the MILLIPLEX^®^ Human Cytokine/Chemokine Magnetic Bead Panel – Immunology Multiplex Assay, which measures levels of IL-10, IL-17A, IL-1β, IL-6, IL-7, MCP-1, and TNF-α. Data acquisition was conducted on a Luminex^®^ 200™ Multiplexing Instrument.

### 2.4 Lipid quantification in plasma by Shotgun Lipidomics

Plasma lipid quantification was performed by mass spectrometry to determine the levels of ceramides, diacylglycerols (DG), free fatty acids (FA), dihydroceramides (DHCer), hexosylceramides (HexCer), lactosylceramides (LacCer), lysophosphatidylcholine (LPC), lysophosphatidylethanolamine (LPE), and triglycerides (TG). Lipid extraction was carried out using a modified Bligh and Dyer method optimized for recovery across lipid classes. Briefly, 50 μL of plasma were mixed with 975 μL of water, 2 mL of methanol, and 900 μL of dichloromethane (DCM). A mixture of internal standards (Usplash^®^, Avanti Polar Lipids Inc.) was added to monitor extraction efficiency and enable absolute quantification. Samples were incubated for 30 min at room temperature, followed by a second extraction with 1 mL of water and 900 μL of DCM. After centrifugation at 1400 × *g* for 10 min, the lower organic phase was collected. Combined organic phases were dried using a LabConco Centrivap concentrator at 30 °C for 90 min and reconstituted in 350 μL of 10 mM ammonium acetate prepared in DCM:MeOH (50:50).

Lipid profiling in human plasma was performed via direct-injection differential mobility spectrometry–mass spectrometry (DMS–MS) according to a previously published method (Su et al., 2021). 90 μL of each sample were injected by flow injection analysis at 8 μL/min using an LC–Exion AD 30 system. Analysis was conducted on a Sciex QTRAP 6500+ platform equipped with a SelexION unit for differential mobility separation. Targeted multiple reaction monitoring (MRM) acquisition was performed under two conditions, DMS on (Method 1) and DMS off (Method 2). Internal standard tuning was carried out using Usplash^®^ (Avanti Polar Lipids Inc.).

Data acquisition and processing were performed using Shotgun Lipidomics Assistant (SLA) v1.5, a Python-based software designed for lipidomics data analysis, including MRM transition selection, isotopic correction, and quantitative normalization. The intensity of each lipid species was normalized to its corresponding internal standard, and absolute concentrations (nmol) were calculated based on the analyzed sample volume (Su et al., 2021).

### 2.5 Statistical analysis

All analyses were conducted in RStudio (version 2024.09.1+394). Data distribution was assessed with normality tests. Group comparisons were performed using Mann–Whitney U test corrected by Benjamini-Hochberg. Associations between plasma lipid species, cytokine concentrations, and anxiety scores were examined with Spearman correlation coefficients. In addition, multiple correlation models were applied to evaluate the relationship between lipid species and cytokines, using anxiety scores as the dependent variable. All models were fitted using rank-based approaches. Statistical significance was set at *p* ≤ 0.05.

## 3 Results

### 3.1 Participants

A total of 34 individuals were included in the study: 17 healthy controls and 17 participants with clinically diagnosed anxiety. Groups were matched for age and sex, each comprising 3 men and 14 women, aged 21–27 years, all in good physical health. Control participants had no history of psychiatric illness and did not exhibit anxiety symptoms at the time of assessment, as confirmed by the CGI-S, GAD-7 and BAI scales. In contrast, individuals in the anxiety group presented clinically significant anxiety symptoms at the time of evaluation and had a confirmed diagnosis of an anxiety disorder established by a board-certified psychiatrist. Symptom severity was determined based on combined scores from the clinical instruments: 10 individuals were classified with mild anxiety, 4 with moderate, and 3 with severe symptoms (Table 1).

**TABLE 1:** Clinical characteristics of the study participants.

VARIABLE	CONTROL (*N* = 17)	ANXIETY (*N* = 17)
SEX (FEMALE/MALE)	14/3	14/3
ANXIETY SEVERITY	–	
• MILD, N (%)	–	10 (58.8)
• MODERATE, N (%)	–	4 (23.5)
• SEVERE, N (%)	–	3 (17.6)

### 3.2 Individuals with anxiety disorders exhibited a systemic pro-inflammatory profile

**FIGURE 1:**
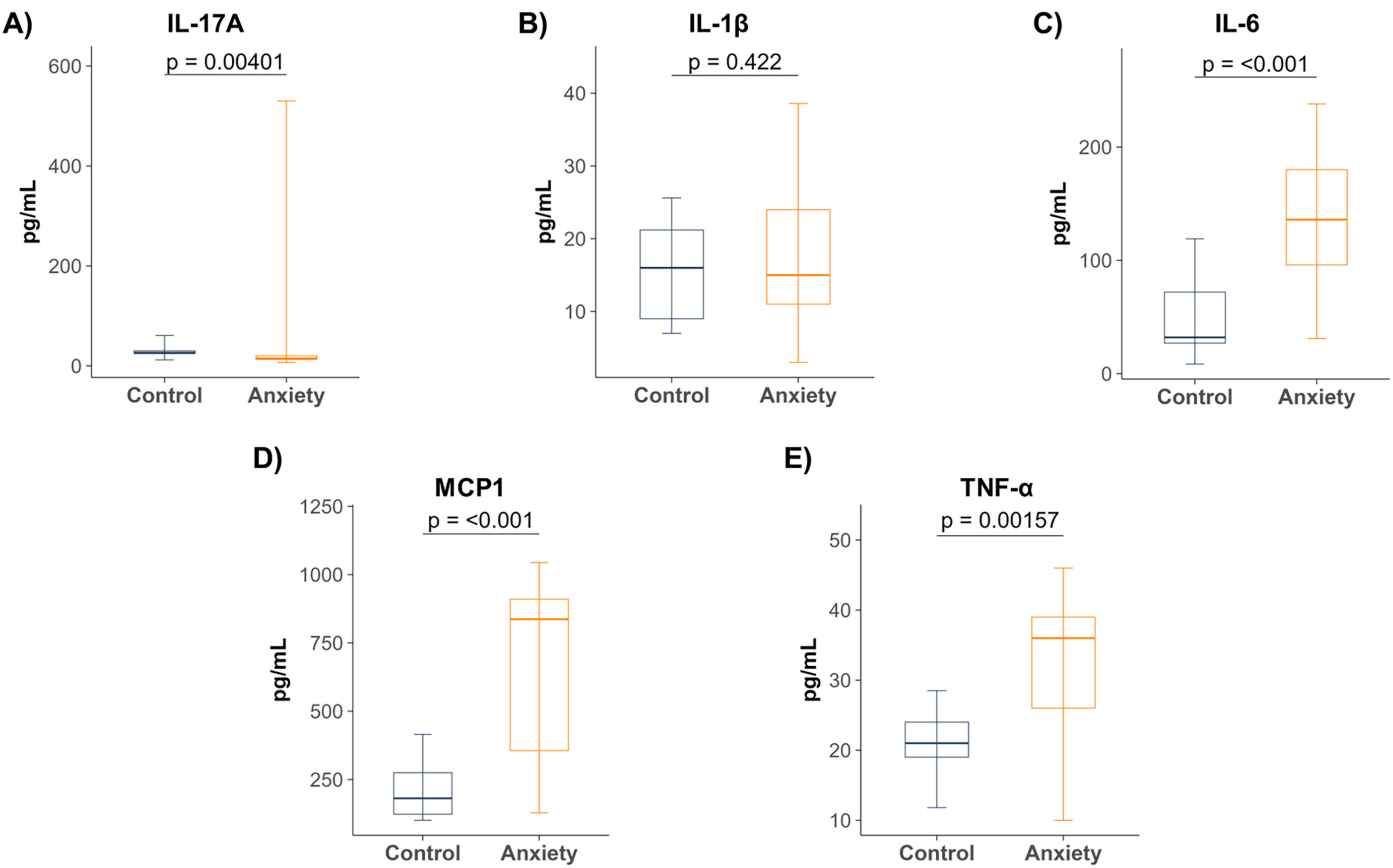
Plasma cytokine levels in individuals with and without anxiety. **(A)** IL-17A was significantly reduced in individuals with anxiety. **(B)** IL-1β showed a non-significant increase. **(C–E)** IL-6, MCP-1, and TNF-α were significantly elevated in the anxiety group. Data are presented as box-and-whisker plots (median, interquartile range, 5th–95th percentiles). Group comparisons were made using the Mann–Whitney U test. Control (*n* = 17), Anxiety (*n* = 17). MCP-1, monocyte chemoattractant protein-1; TNF-α, tumor necrosis factor alpha.

Plasma levels of IL-10, IL-17A, IL-1β, IL-6, IL-7, MCP-1, and TNF-α were quantified in individuals with anxiety and healthy controls. Anxious participants exhibited a general tendency toward elevated pro-inflammatory cytokines compared with controls. Significant increases were observed for IL-6 (*p* = 0.0001), MCP-1 (*p* = 0.0001), and TNF-α (*p* = 0.0001). IL-1β showed a non-significant increase. Conversely, IL-17A levels were significantly decreased in the anxiety group (*p* = 0.01) (Figure 1). IL-10 and IL-7 were inconsistently detected across samples and excluded from subsequent analyses.

### 3.3 Exploratory plasma lipidomic profiling suggests nominal reductions in specific glycerolipids and lysophospholipids

**FIGURE 2:**
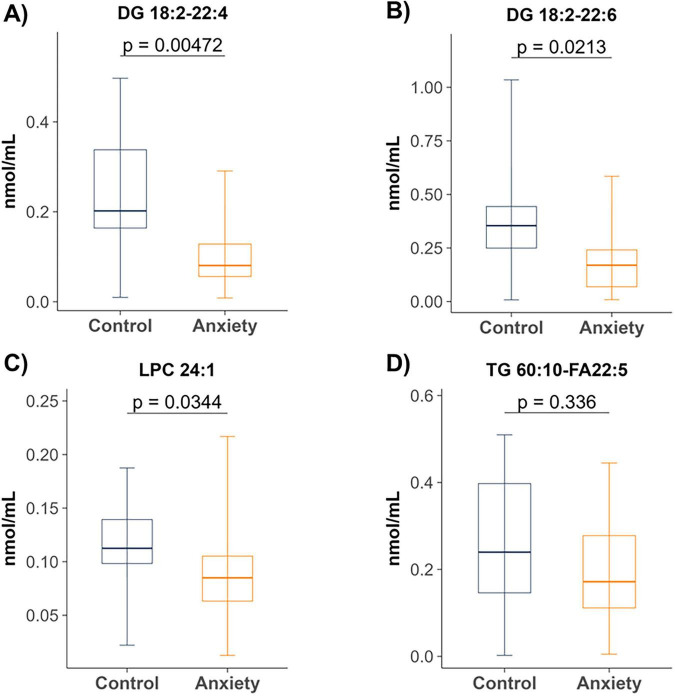
Plasma lipid species differentially expressed between individuals with and without anxiety. Levels of panels **(A)** DG 18:2/22:4, **(B)** DG 18:2/22:6, **(C)** LPC 24:1 and **(D)** TG 60:11 (FA 22:5) were significantly reduced in the anxiety group. Box-and-whisker plots represent median, interquartile range, and 5th–95th percentiles. Mann–Whitney U tests were used for group comparisons. Control (*n* = 17), Anxiety (*n* = 17). DG, diacylglycerol; LPC, lysophosphatidylcholine; TG, triglyceride; FA, fatty acid.

Targeted lipidomic analysis was performed on plasma samples to quantify ceramides and related sphingolipids, diacylglycerols, free fatty acids, lysophosphatidylcholine, lysophosphatidylethanolamine, and triglycerides. In the initial, unadjusted analyses, no significant differences were observed in plasma concentrations of total ceramides, dihydroceramides, hexosylceramides, or lactosylceramides between groups (Supplementary Material 1). In contrast, individuals with anxiety showed lower nominal plasma levels of DG 18:2/22:4 (*p* = 0.004), DG 18:2/22:6 (*p* = 0.02), LPC 24:1 (*p* = 0.03), and TG 60:11 (FA 22:5) (*p* = 0.04) compared with controls (Figure 2).

When *p*-values were adjusted for multiple testing using the Benjamini–Hochberg false discovery rate procedure, none of these group differences remained statistically significant (Supplementary Material 2). These results point toward specific lipid pathways that may be altered in clinical anxiety.

### 3.4 Plasma cytokine levels correlated with anxiety severity

**FIGURE 3:**
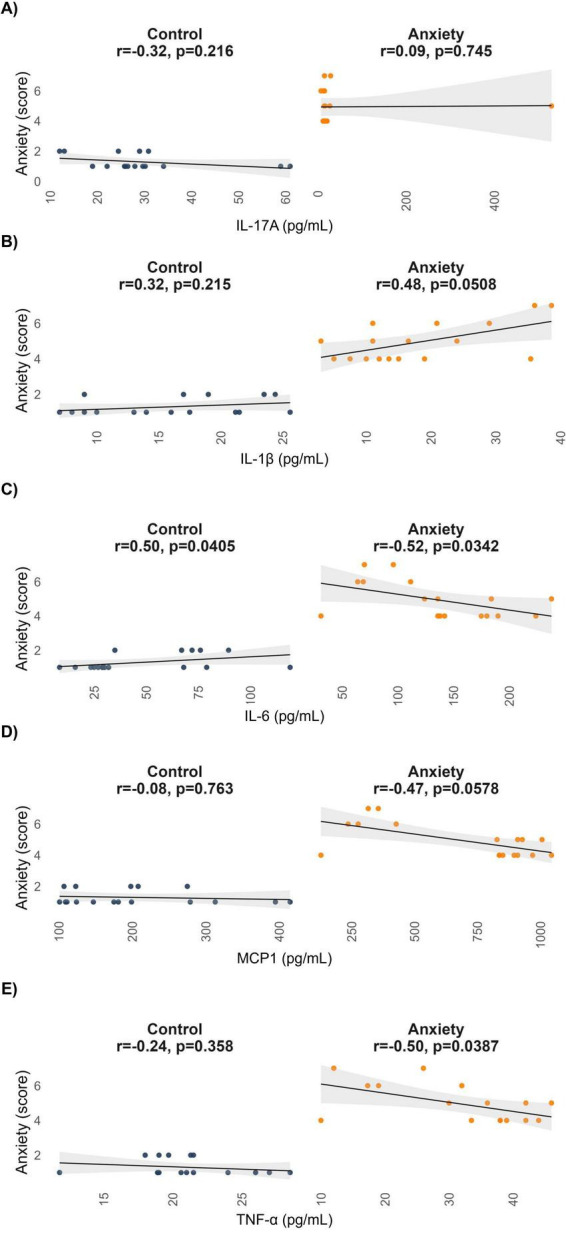
Correlations between cytokines levels and anxiety symptom scores. Scatterplots display relationships for panels **(A)** IL-17A, **(B)** IL-1β, **(C)** IL-6, **(D)** MCP-1, and **(E)** TNF-α with anxiety severity in control and anxiety group. Lines represent linear fits with 95% confidence interval (shaded). Spearman correlation was used for statistical analysis. Control (*n* = 17), Anxiety (*n* = 17). MCP-1, monocyte chemoattractant protein 1; TNF-α, tumor necrosis factor alpha.

To evaluate whether cytokine levels scaled with anxiety symptom severity, Spearman correlation analyses were conducted between plasma cytokine concentrations and the composite anxiety score. IL-17A and IL-1β showed no significant associations in either group. IL-6 was positively correlated with anxiety scores in both controls (rho = 0.50, *p* = 0.04) and anxiety groups (rho = 0.52, *p* = 0.03). MCP-1 showed no correlation in controls (rho = −0.08, *p* = 0.763) but was negatively associated with severity in the anxiety group (rho = −0.47, *p* = 0.05). TNF-α also showed a negative correlation in the anxiety group (rho = −0.50, *p* = 0.03), but, not in controls (rho = −0.24, *p* = 0.35) (Figure 3). Overall, IL-6 emerged as the only cytokine consistently associated with anxiety severity across groups, whereas MCP-1 and TNF-α showed group-specific associations limited to clinical anxiety cases.

### 3.5 Plasma lipid species do not correlate with anxiety severity

We next examined whether plasma lipid concentrations were associated with anxiety severity. Spearman correlation analyses were performed across all lipid classes, including ceramides, dihydroceramides, hexosylceramides, lactosylceramides, free fatty acids, lysophospholipids, diacylglycerols, and triacylglycerols. No lipid species showed a statistically significant association with the composite anxiety score (Supplementary Material 3).

### 3.6 Combined lipid–cytokine models were not associated with anxiety severity

Finally, we explored whether joint alterations in plasma lipids and inflammatory markers could predict anxiety severity. Rank-based regression models were fitted for each lipid–cytokine pair, with the composite anxiety score as the outcome. Across all models, lipid species did not exhibit independent associations with anxiety severity when cytokine levels were included as covariates. Full regression results are available in Supplementary Material 4.

## 4 Discussion

Neurobiological traits that predispose individuals to anxiety have been proposed in several reports. In this study, we enriched the characterization of inflammatory and lipidomic signatures in Mexicans individuals according to their anxiety symptom severity. We found that individuals with clinical anxiety exhibited elevated plasma levels of IL-6, MCP-1, and TNF-α, together with reduced IL-17A, supporting the presence of a pro-inflammatory systemic profile. In parallel, exploratory lipidomic analyses suggested a trend toward lower circulating levels of DG 18:2/22:4, DG 18:2/22:6, LPC 24:1, and TG 60:11 (FA 22:5) in the anxiety group, although these associations were not sustained after multiple testing correction. Regression analyses confirmed group-specific associations of MCP-1 and TNF-α with anxiety in the clinical group. These findings support the existence of a pro-inflammatory molecular profile in Mexicans individuals diagnosed with anxiety.

Both clinical and preclinical studies have consistently shown that anxiety disorders are associated with altered levels of circulating inflammatory mediators. Several studies have reported increased concentrations of IL-6, TNF-α and IL-1β in individuals with generalized anxiety disorder compared to healthy controls, suggesting a possible role for peripheral immune activation (Tang et al., 2018). Among these, IL-6 has emerged as a particularly robust marker. For example, elevated IL-6 levels correlate with Hamilton Anxiety Scale scores in Chinese clinical cohorts (Zou et al., 2020) and display weak to moderate positive correlations with Hamilton scores in individuals with atopic dermatitis (Yang et al., 2025). Similarly, patients with rheumatoid arthritis and comorbid anxiety show increased levels of IL-6, TNF-α and IL-17A (Liu et al., 2012). TNF-α and IL-17A have also been positively associated with anxiety scores in Chinese patients with coronary artery disease (Lu et al., 2022) and postpartum women with anxiety and depression (Min et al., 2022). More recently, a case–control study conducted in Bangladesh reported that serum IL-17A concentrations were higher in patients with generalized anxiety disorder and correlated positively with symptom severity (Roknuzzaman et al., 2024). It is important to note that several reports analyze populations with pre-existing inflammatory comorbidities. These underlying conditions often involve a high degree of systemic immune activation, which may differ from the inflammatory profiles observed in our cohort. Moreover, methodological and analytical factors can strongly affect IL-17A measurements. IL-17A is a low-abundance analyte and shows high assay variability across platforms; several validation studies note poor precision for IL-17A in multiplex and ELISA formats, especially near the lower limit of detection. In contrast, IL-17A is relatively stable to delay processing in common anticoagulants, but differences in kit sensitivity, sample dilution and calibration can still produce discrepant results between studies (Surenaud et al., 2016; Nakamizo et al., 2024). Together, these technical issues argue for caution when interpreting single-study differences in IL-17A.

Conversely, studies in Middle Eastern populations have reported lower IL-6 and TGF-β1 levels in GAD patients compared to controls, and a negative association between IL-6 and GAD-7 scores (Alfi et al., 2025). In Mexican cohorts, MCP-1 has been linked to anxiety scores during late pregnancy (Camacho-Arroyo et al., 2021), while elevated MCP-1 levels have also been observed in anxious patients with alcohol use disorder from South Asian populations (Kazmi et al., 2022). More recently, increased serum CCL5 concentrations have been documented in Indian patients with generalized anxiety disorder (Suresh et al., 2025).

Our findings align with these reports by confirming higher levels of IL-6, MCP-1, and TNF-α in Mexican individuals diagnosed with anxiety. Specifically, we observed that MCP-1 and TNF-α were positively associated with anxiety severity, supporting their relevance as pro-inflammatory markers at the clinical threshold. These results reinforce the growing body of literature linking peripheral inflammation to anxiety symptoms. Nevertheless, cross-study variability in inflammatory profiles likely reflects differences in sample characteristics, comorbid conditions, and methodological approaches.

In the current study, targeted lipidomic analysis identified selective, nominal alterations in glycerolipids and lysophospholipids, whereas no group-level changes were observed in sphingolipids such as ceramides, dihydroceramides, hexosylceramides, or lactosylceramides. Specifically, individuals with anxiety exhibited lower levels of diacylglycerol species DG 18:2/22:4 and DG 18:2/22:6, decreased lysophosphatidylcholine 24:1, and reduced TG 60:11 (FA 22:5). Notably, these exploratory findings did not withstand multiple comparison correction; however, they point toward specific lipid signatures that contrast with previous reports across diverse clinical populations. For example, Demirkan et al. (2013) observed lower concentrations of PC O-36:4 and a reduced sphingomyelin C23:1/SPM C16:0 ratio, both negatively associated with anxiety scores in a large Dutch cohort. Altered levels of lysophospholipids have also been described: LPC 16:0 and LPE 18:0 were elevated in anxiety patients compared to controls (Kui et al., 2022). Moreover, early studies reported increased plasma triglyceride concentrations in anxiety and anxiety–depression groups (Pistorio et al., 2011), and serum triglycerides were found to correlate with STAI-T scores in both anxiety and comorbid depression samples (Hu et al., 2018; Wang et al., 2016). A metabolomic study of Parkinson’s disease patients with comorbid anxiety identified reduced levels of several diacylglycerols and sphingolipid-related species, as well as oxidized lipid derivatives, compared to patients without anxiety (Dong et al., 2021). These discrepancies underscore the biological heterogeneity of anxiety disorders and suggest that lipidomic profiles may be highly sensitive to cohort-specific factors such as age, ethnicity, and metabolic state. Together, these studies highlight the potential contribution of altered lipid metabolism to anxiety pathophysiology. Rather than definitive markers, our findings provide a hypothesis-generating framework to further explore the lipid–immune axis.

Mechanistically, interactions between lipid signaling and inflammation may provide new insights into the biological basis of anxiety disorders. For instance, glucocorticoid signaling and phospholipase activation, both relevant to stress responses, can modulate PC/PE ratios, phosphatidic acid, DG and TAG pools (Zorkina et al., 2024). These pathways link psychological stress to circulating lipid signatures. Nonetheless, the direction and magnitude of lipid alterations vary across studies, likely reflecting differences in cohort characteristics, comorbidities, medication use and the analytical strategies (Wei et al., 2019). Several factors may help explain discrepancies between our results and prior reports, including differences in age, nutritional status, body composition, comorbid conditions and medication exposure (Demirkan et al., 2013; Dong et al., 2021; Kui et al., 2022). Moreover, the targeted nature of our lipidomic approach may have missed species reported in untargeted analyses. Importantly, our sample included young, physically healthy participants and excluded individuals receiving psychotropic treatment, which may have uncovered an early-stage or distinct metabolic signature compared to older or clinically complex cohorts.

Finally, we did not find significant correlations between individual lipid species and the composite anxiety severity scores, and regression models combining lipids and cytokines also failed to identify independent predictors. This lack of association may be partly explained by the limited sample size and by the multidimensional nature of anxiety, in which distinct symptom domains (e.g., somatic, cognitive, or affective components) may relate differentially to metabolic alterations. In this context, group-level lipid differences may reflect state-dependent metabolic shifts or biologically defined subgroups rather than linear scaling with overall symptom severity. Future studies with larger cohorts and dimensional symptom assessments will be necessary to determine whether specific lipid signatures associate with particular anxiety phenotypes or clinical subdomains.

## 5 Conclusion

The present study examined the interplay between lipidomic alterations and pro-inflammatory signatures in relation to anxiety symptomatology in a Mexican cohort of young adults. We identified MCP-1 and TNF-α as inflammatory markers showing group-specific associations restricted to individuals with clinically diagnosed anxiety. In parallel, targeted lipidomic analyses revealed selective reductions in specific glycerolipid and lysophospholipid species, including diacylglycerols, lysophosphatidylcholine, and triglycerides. Although these lipid alterations did not scale linearly with anxiety severity, they may reflect state-dependent metabolic changes or biologically distinct subgroups within anxiety phenotypes. Consequently, these lipidomic signatures represent preliminary insights that warrant further targeted investigation to define their clinical relevance. Elucidating whether these molecular shifts represent causal mechanisms, compensatory responses, or biomarkers of vulnerability will require larger, longitudinal, and multimodal studies. Future investigations integrating lipid flux analyses, expanded metabolomic profiling, and comprehensive immune phenotyping may help clarify the role of lipid–immune interactions in anxiety and identify biomarkers or therapeutic targets that complement current clinical approaches.

### 5.1 Limitations of the current study

Several limitations should be considered when interpreting these findings. First, the modest sample size limits statistical power and reduces the ability to detect subtle associations between lipid species, inflammatory markers, and anxiety severity. Given the sample size of 34 participants, our study was powered to detect large effect sizes with 80% power at a nominal alpha of 0.05. The associations identified in our exploratory lipidomic profiling should be interpreted within this context. Second, the cross-sectional design precludes causal inference regarding the temporal relationships between metabolic and inflammatory alterations and anxiety symptoms. Third, the targeted nature of the lipidomic platform restricted the analysis to a predefined subset of lipid species, potentially overlooking relevant metabolites identified in untargeted approaches. Finally, lifestyle-related variables that can influence lipid metabolism and inflammatory status, including dietary patterns, body mass index, and body composition, were not systematically assessed and therefore could not be included in the analyses. Although participants were young and medication-free, unmeasured lifestyle factors may have contributed to interindividual variability in plasma lipid and cytokine levels.

Despite these limitations, the selective alterations observed provide a focused molecular framework for future studies. These findings support further investigation into the interaction between lipid metabolism and inflammation as a contributing factor in anxiety-related pathophysiology.

## Data Availability

The data supporting the findings of this study, including cytokine measurements, lipidomic profiles, and clinical variables, are available from the corresponding author upon reasonable request.
