# Supplementary material for: Anxiety is associated with systemic pro-inflammatory profile and plasma lipid changes in Mexican young adults
Source: Front Cell Neurosci. 2026 Feb 27;20:1777048. doi: 10.3389/fncel.2026.1777048 (PMC12982057; doi:10.3389/fncel.2026.1777048)
Supplement: Supplementary file 1 [file Data_Sheet_1.pdf]

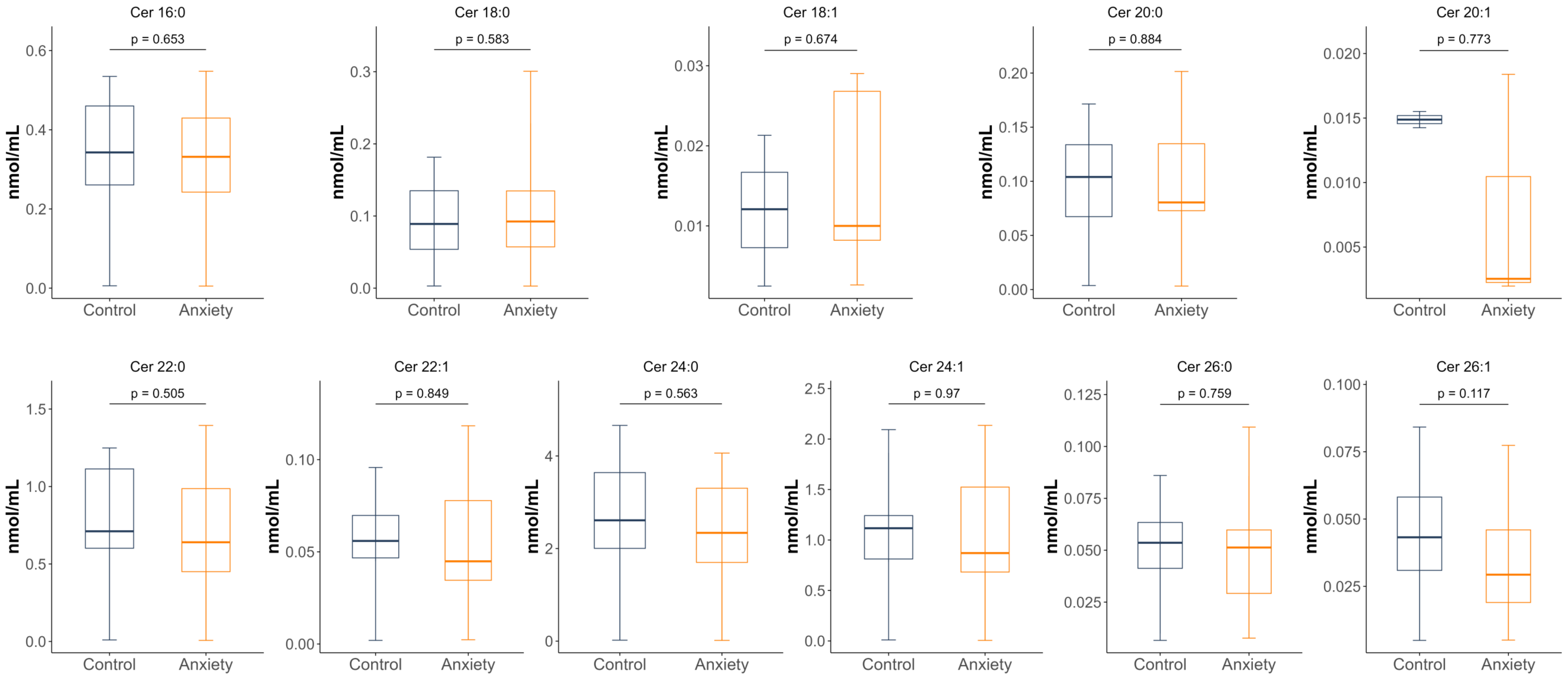

Supplementary Figure 1. Plasma ceramide species profile. Results are presented as box-and-whisker plots showing the median, interquartile range, and 5th–95th percentiles. Differences between group were assessed using the Mann–Whitney U test. Control (n = 17), Anxiety (n = 17).

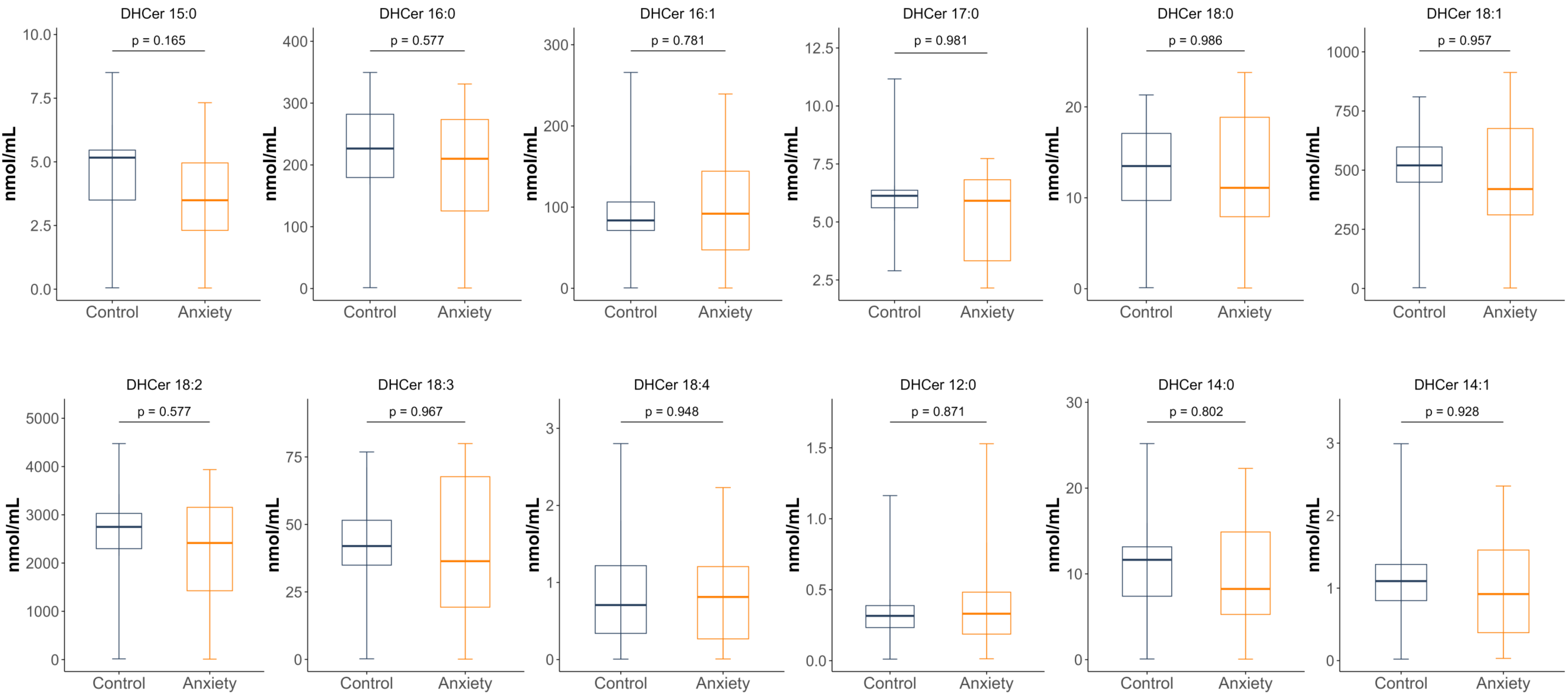

Supplementary Figure 2. Plasma dihydroceramide species profile. Results are presented as box-and-whisker plots showing the median, interquartile range, and 5th–95th percentiles. Differences between group were assessed using the Mann–Whitney U test. Control (n = 17), Anxiety (n = 17).

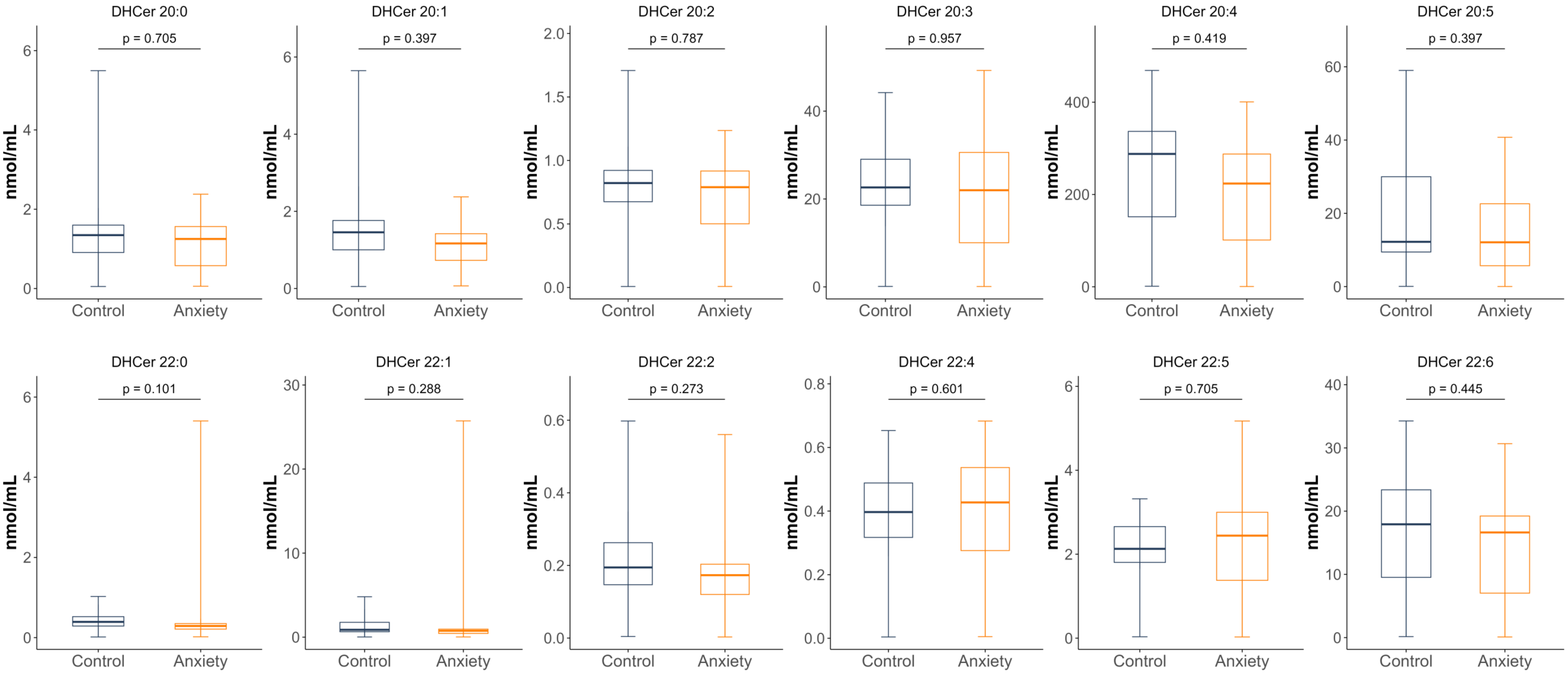

Supplementary Figure 3. Plasma dihydroceramide species profile. Results are presented as box-and-whisker plots showing the median, interquartile range, and 5th–95th percentiles. Differences between group were assessed using the Mann–Whitney U test. Control (n = 17), Anxiety (n = 17).

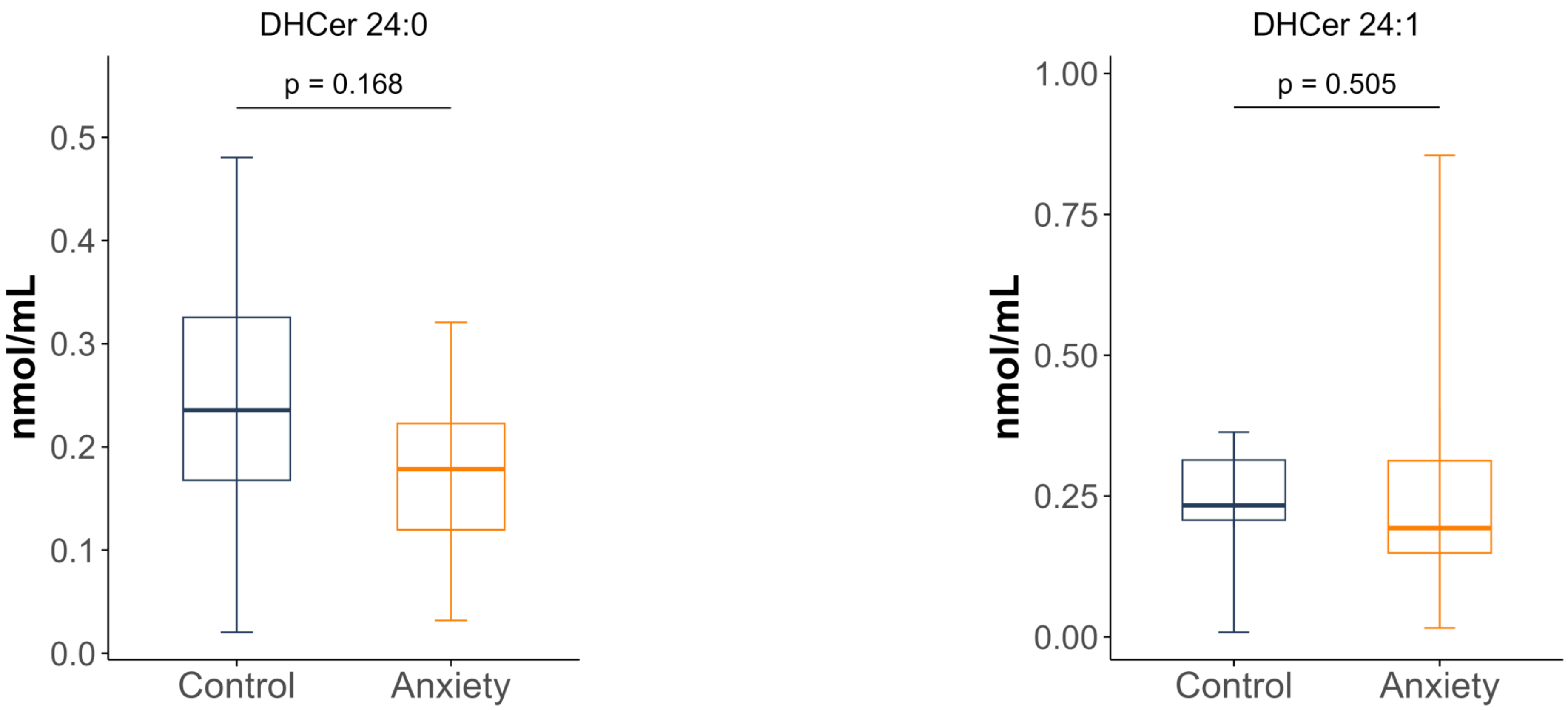

Supplementary Figure 4. Plasma dihydroceramide species profile. Results are presented as box-and-whisker plots showing the median, interquartile range, and 5th–95th percentiles. Differences between group were assessed using the Mann–Whitney U test. Control (n = 17), Anxiety (n = 17).

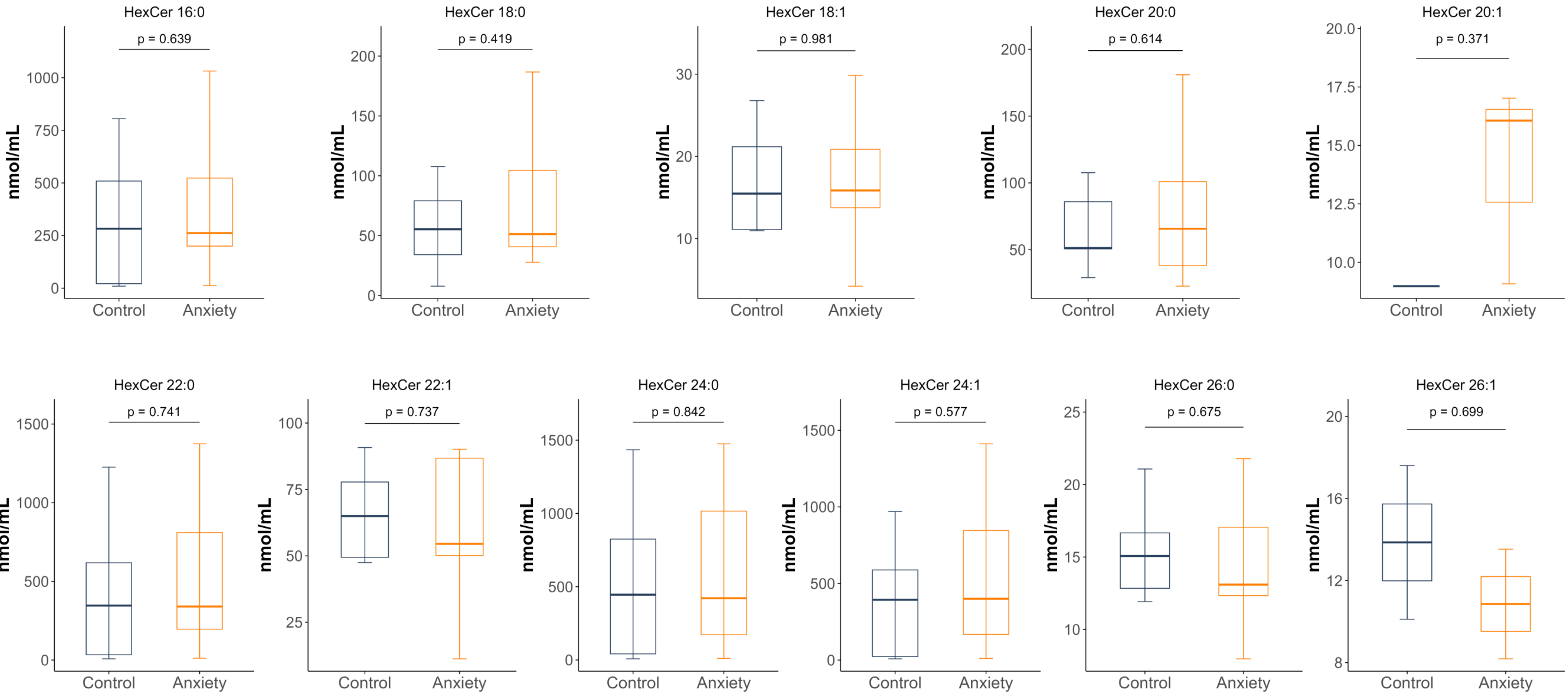

Supplementary Figure 5. Plasma hexosylceramide species profile. Results are presented as box-and-whisker plots showing the median, interquartile range, and 5th–95th percentiles. Differences between group were assessed using the Mann–Whitney U test. Control (n = 17), Anxiety (n = 17).

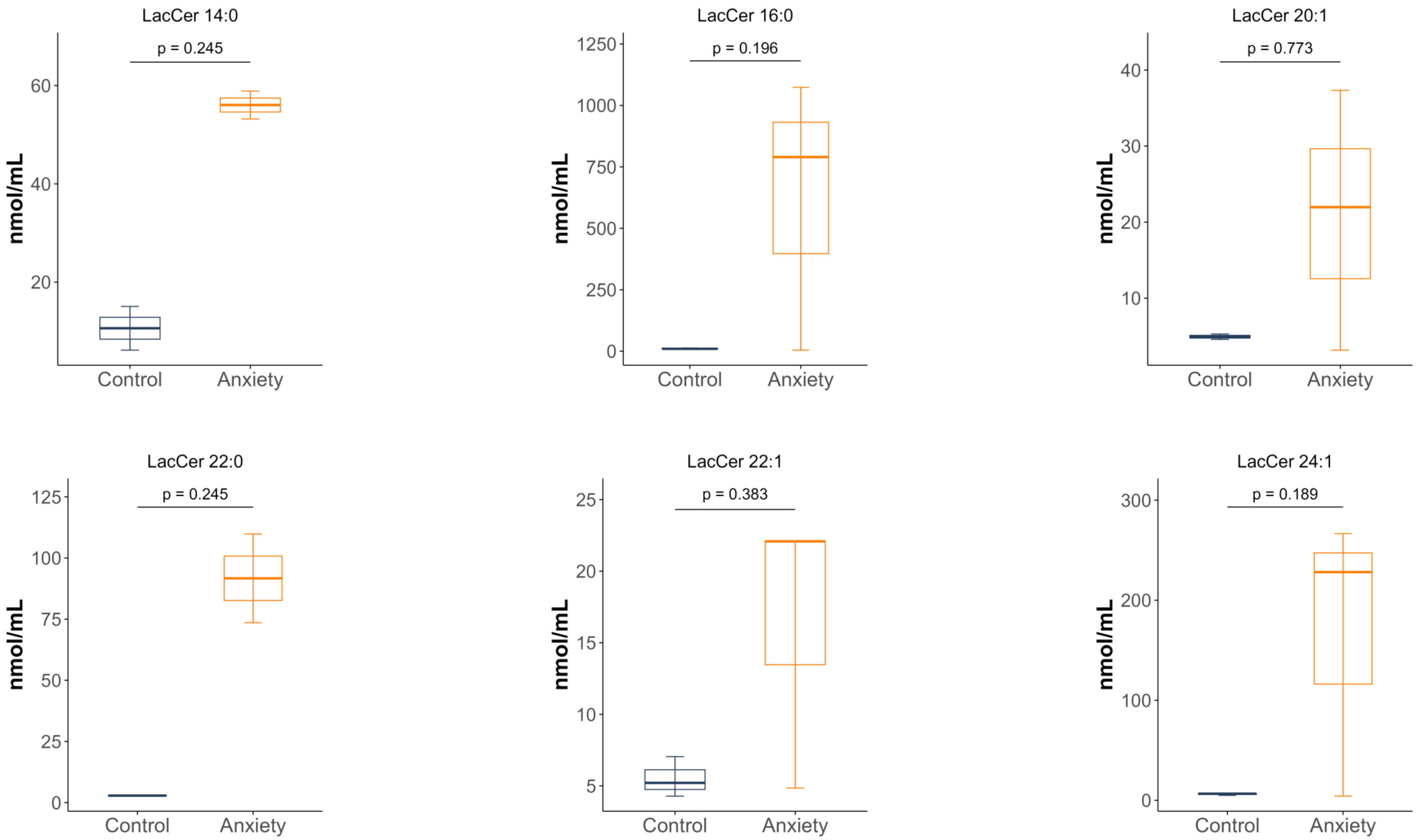

Supplementary Figure 6. Plasma lactosylceramide species profile. Results are presented as box-and-whisker plots showing the median, interquartile range, and 5th–95th percentiles. Differences between group were assessed using the Mann–Whitney U test. Control (n = 17), Anxiety (n = 17).

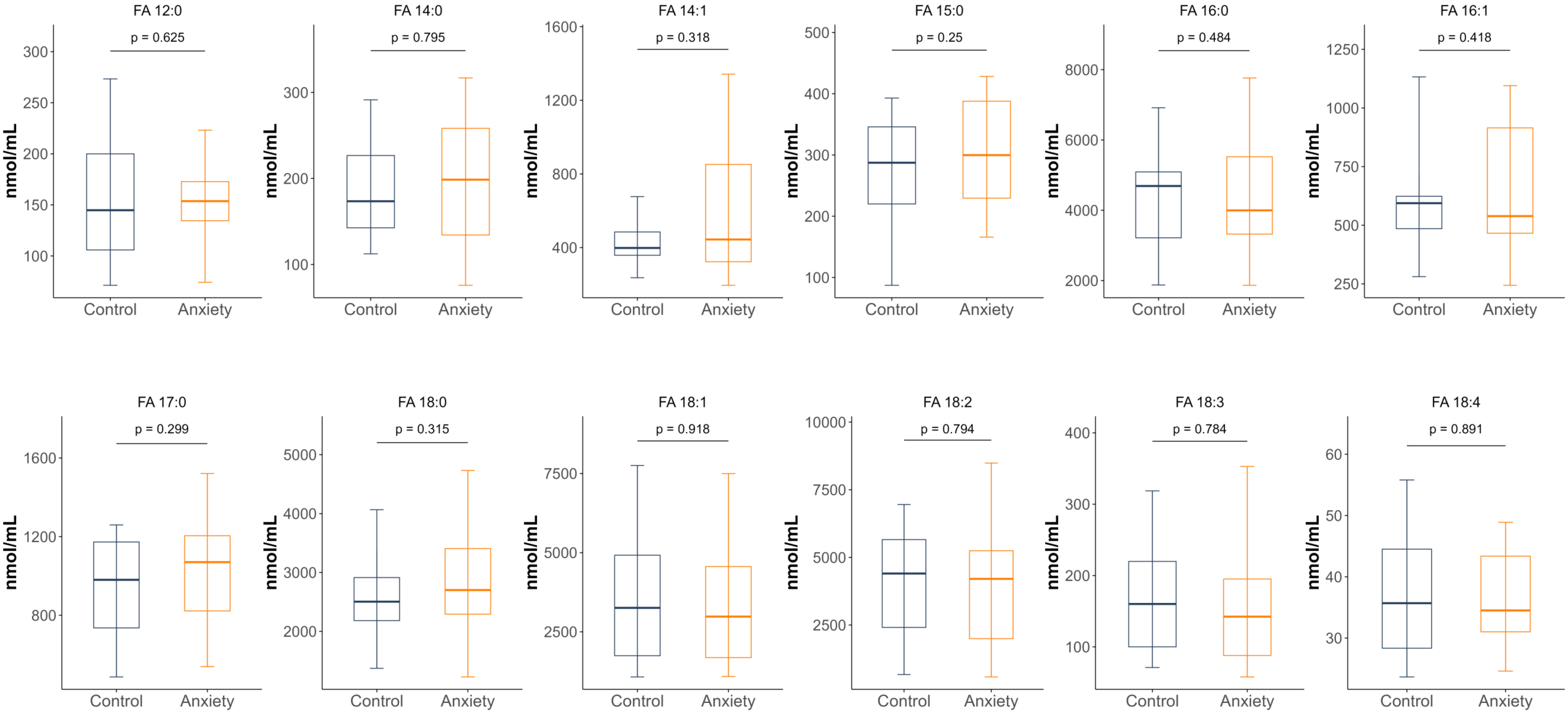

Supplementary Figure 7. Plasma fatty acids profile. Results are presented as box-and-whisker plots showing the median, interquartile range, and 5th–95th percentiles. Differences between group were assessed using the Mann–Whitney U test. Control (n = 17), Anxiety (n = 17).

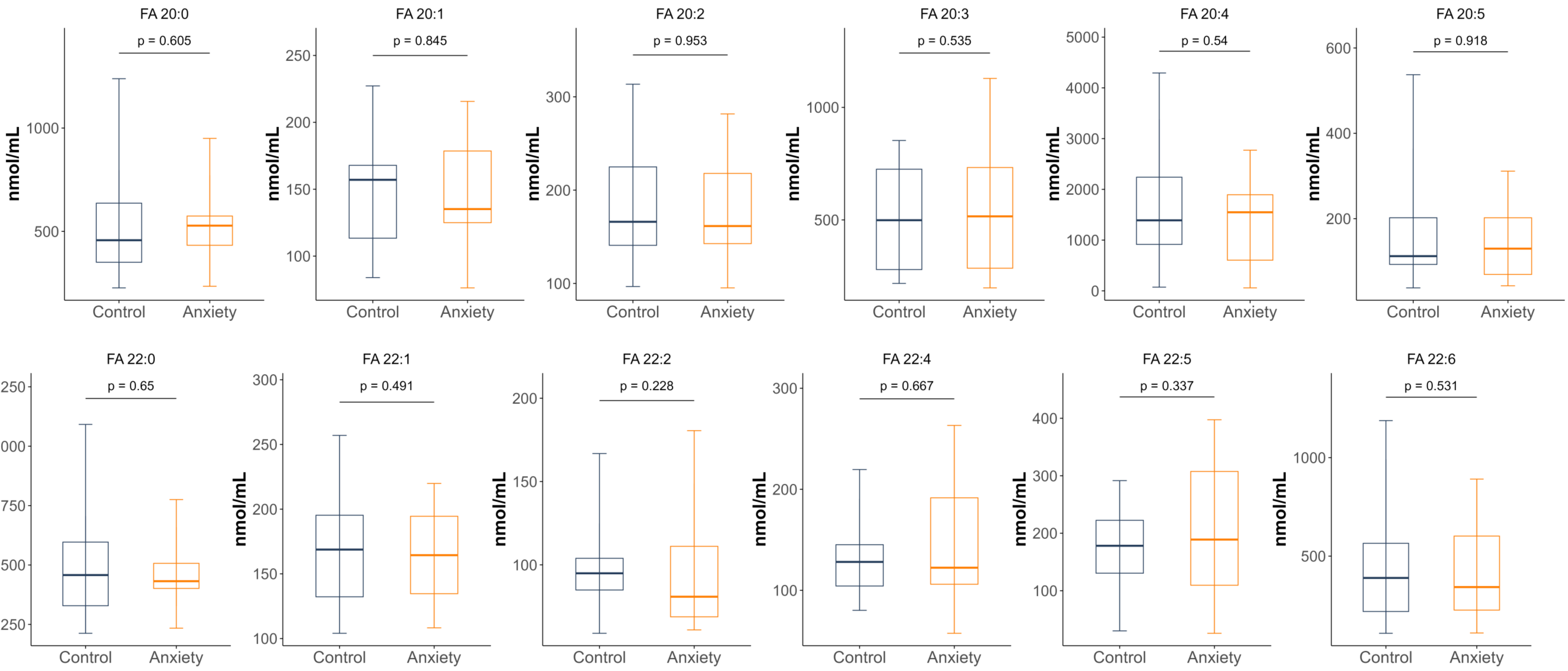

Supplementary Figure 8. Plasma fatty acids profile. Results are presented as box-and-whisker plots showing the median, interquartile range, and 5th–95th percentiles. Differences between group were assessed using the Mann–Whitney U test. Control (n = 17), Anxiety (n = 17).

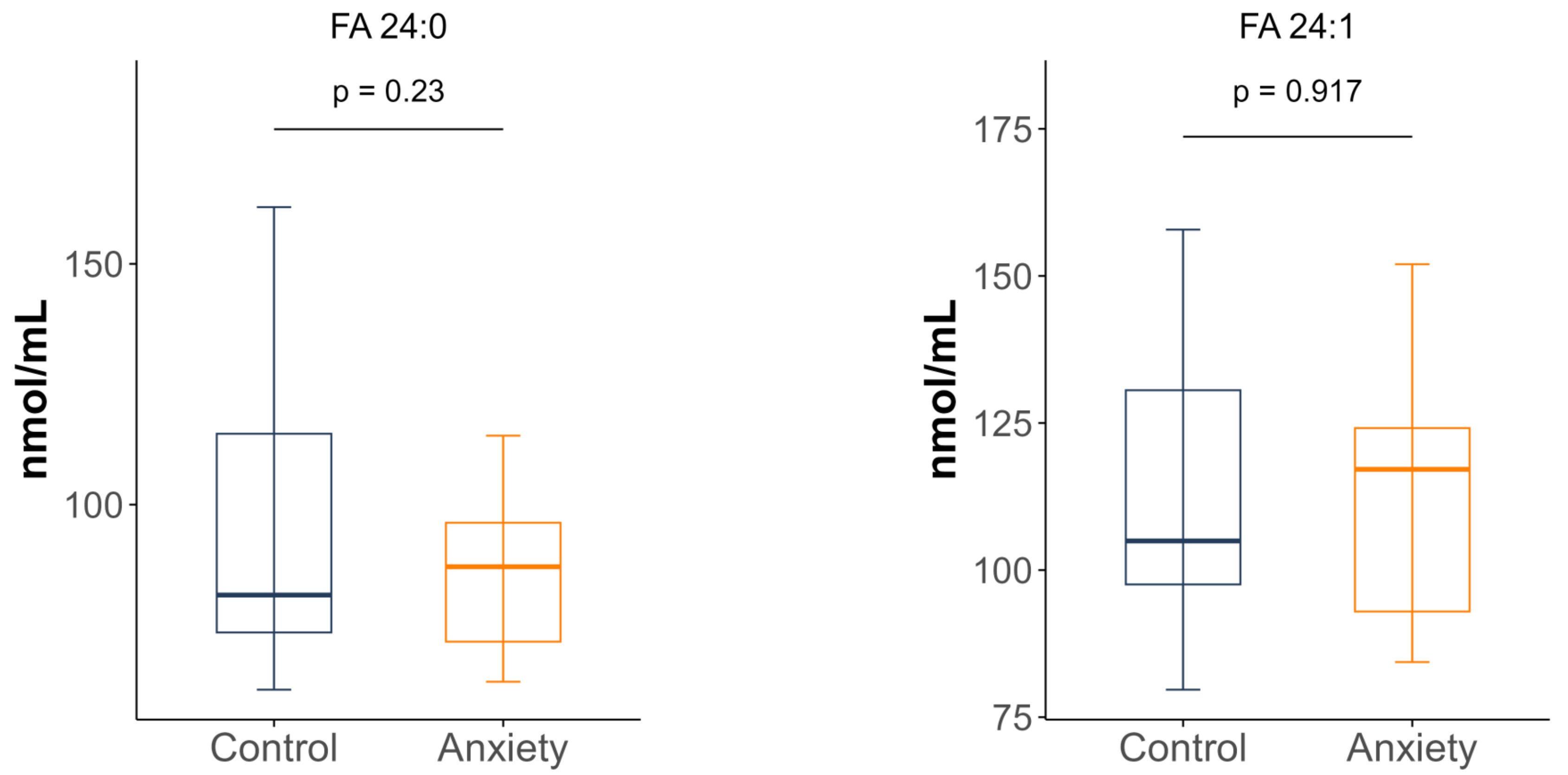

Supplementary Figure 9. Plasma fatty acids profile. Results are presented as box-and-whisker plots showing the median, interquartile range, and 5th–95th percentiles. Differences between group were assessed using the Mann–Whitney U test. Control (n = 17), Anxiety (n = 17).

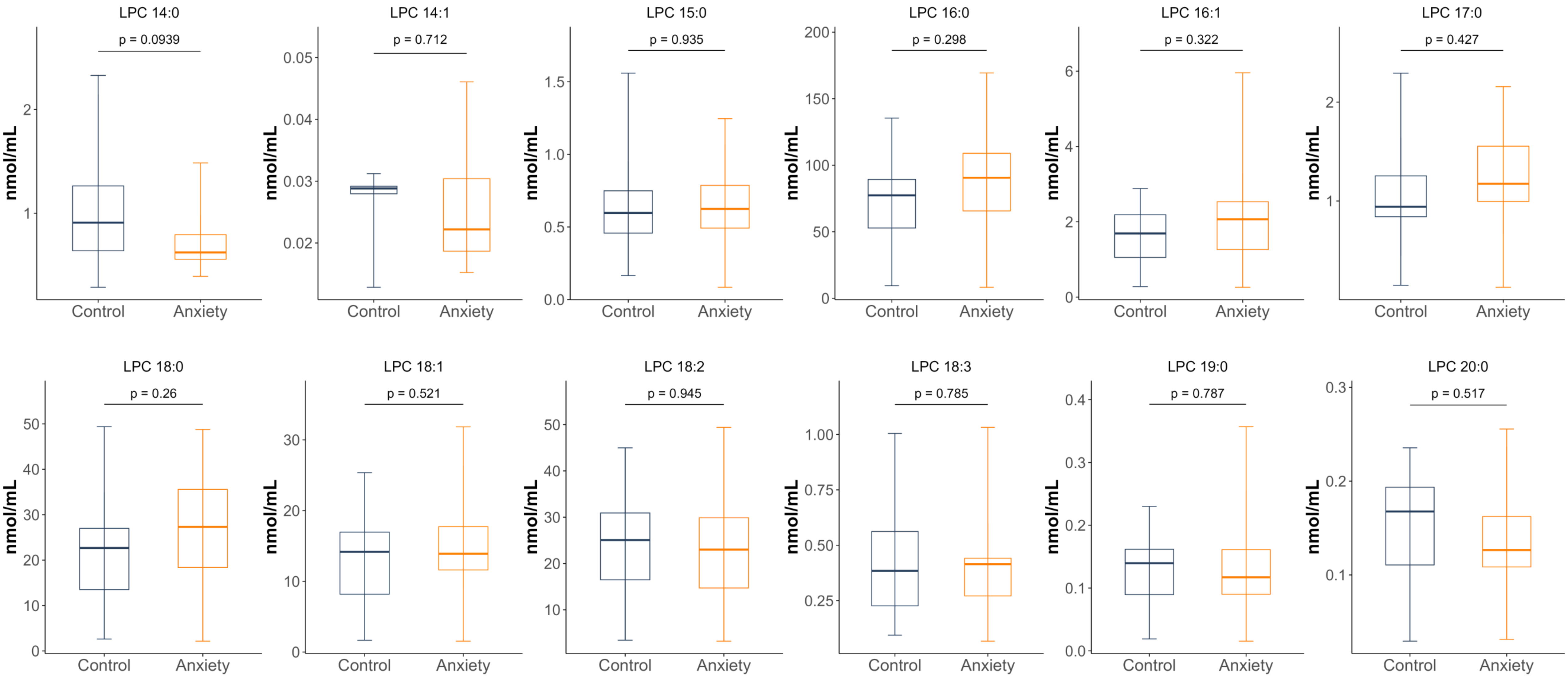

Supplementary Figure 10. Plasma lysophophatidylcholine species profile. Results are presented as box-and-whisker plots showing the median, interquartile range, and 5th–95th percentiles. Differences between group were assessed using the Mann–Whitney U test. Control (n = 17), Anxiety (n = 17).

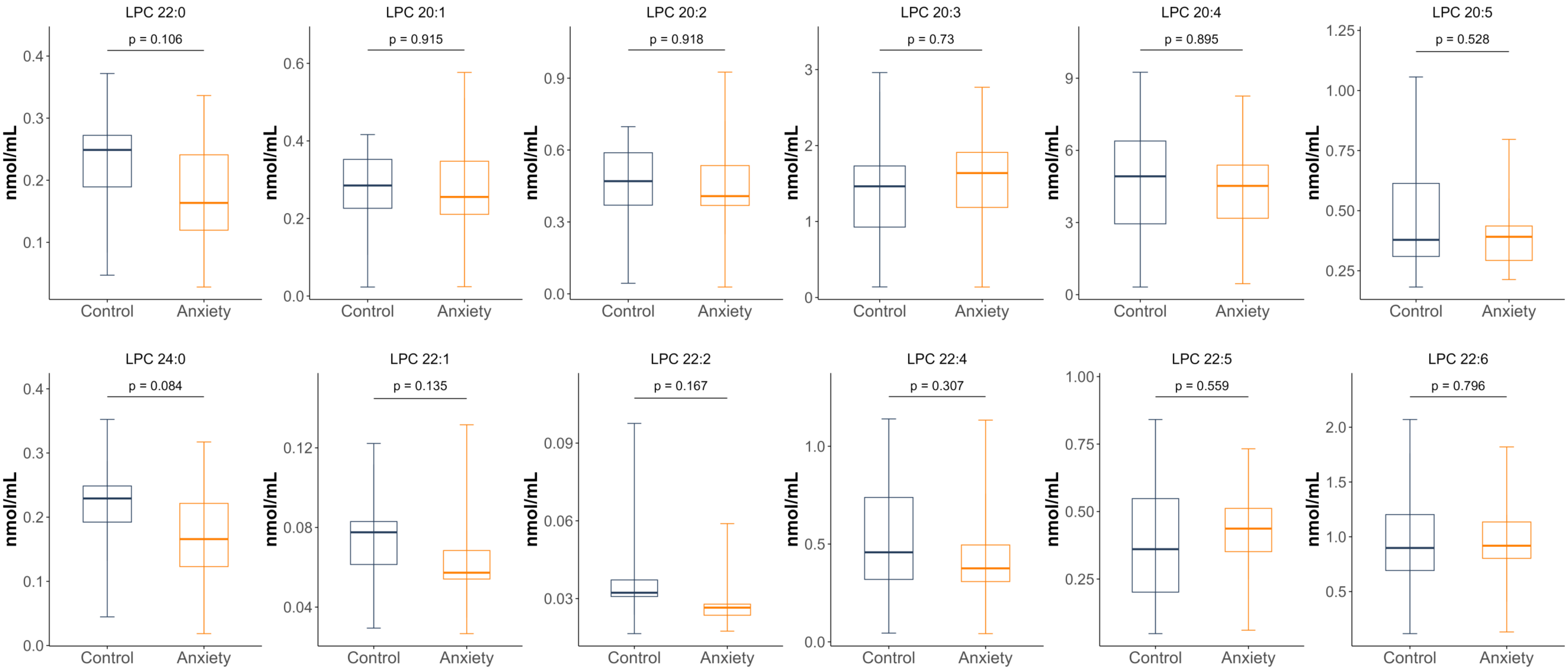

Supplementary Figure 11. Plasma lysophophatidylcholine species profile. Results are presented as box-and-whisker plots showing the median, interquartile range, and 5th–95th percentiles. Differences between group were assessed using the Mann–Whitney U test. Control (n = 17), Anxiety (n = 17).

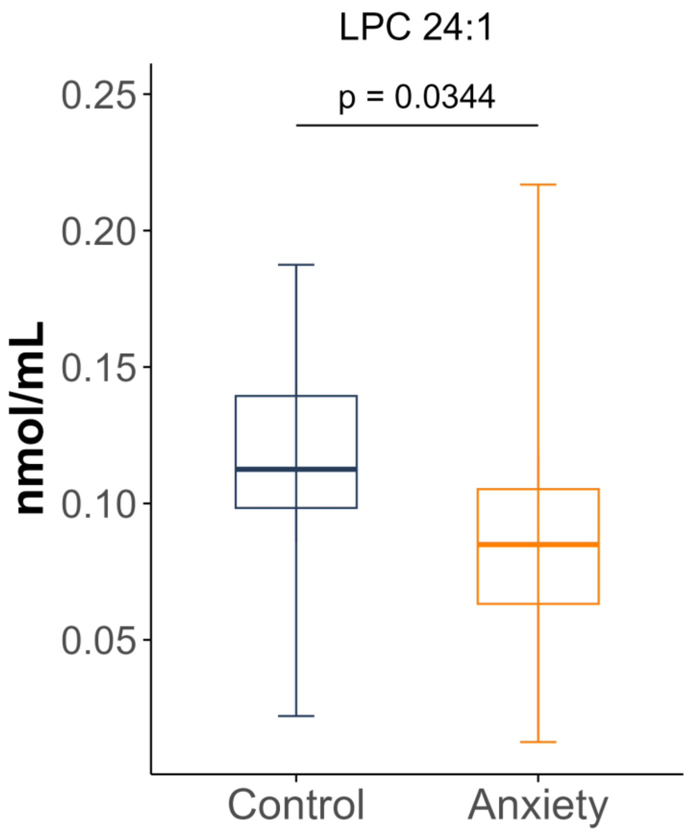

Supplementary Figure 12. Plasma lysophophatidylcholine species profile. Results are presented as box-and-whisker plots showing the median, interquartile range, and 5th–95th percentiles. Differences between group were assessed using the Mann–Whitney U test. Control (n = 17), Anxiety (n = 17).

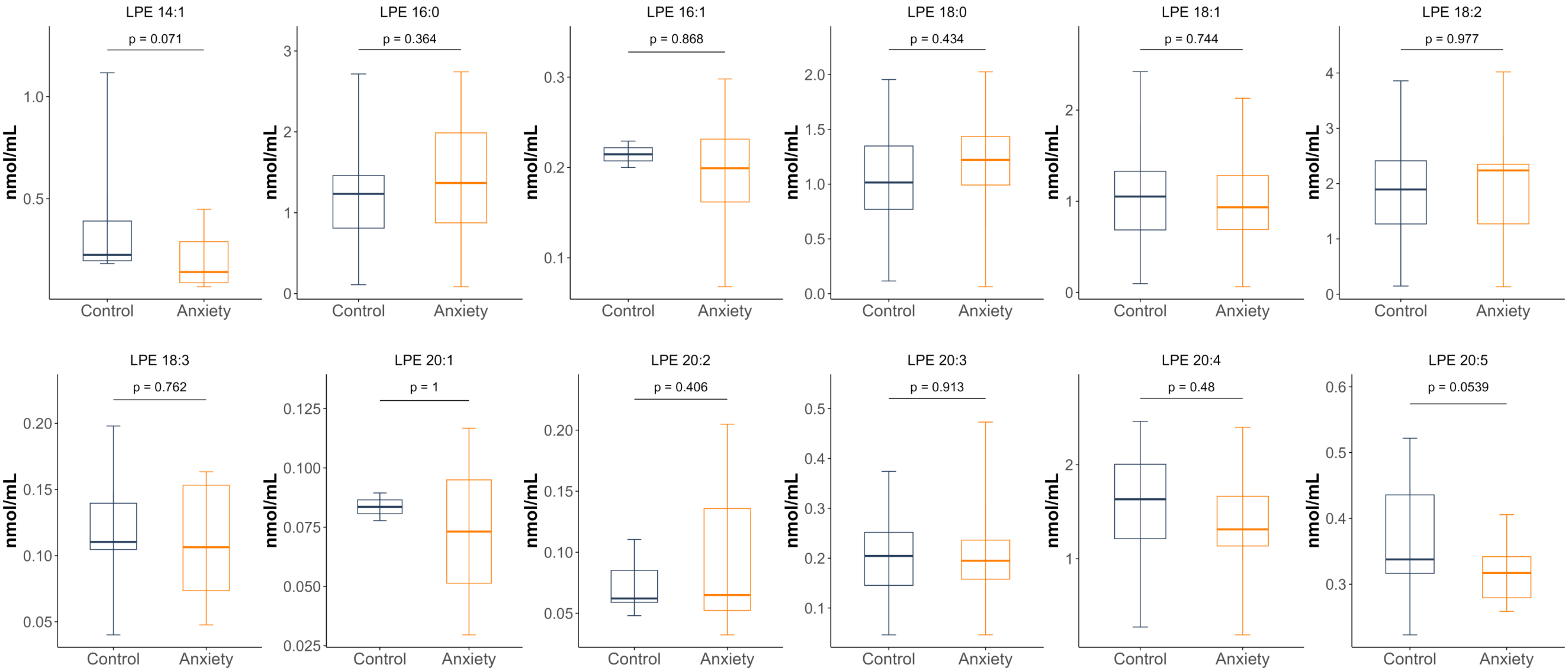

Supplementary Figure 13. Plasma lysophophatidylethanolamine species profile. Results are presented as box-and-whisker plots showing the median, interquartile range, and 5th–95th percentiles. Differences between group were assessed using the Mann–Whitney U test. Control (n = 17), Anxiety (n = 17).

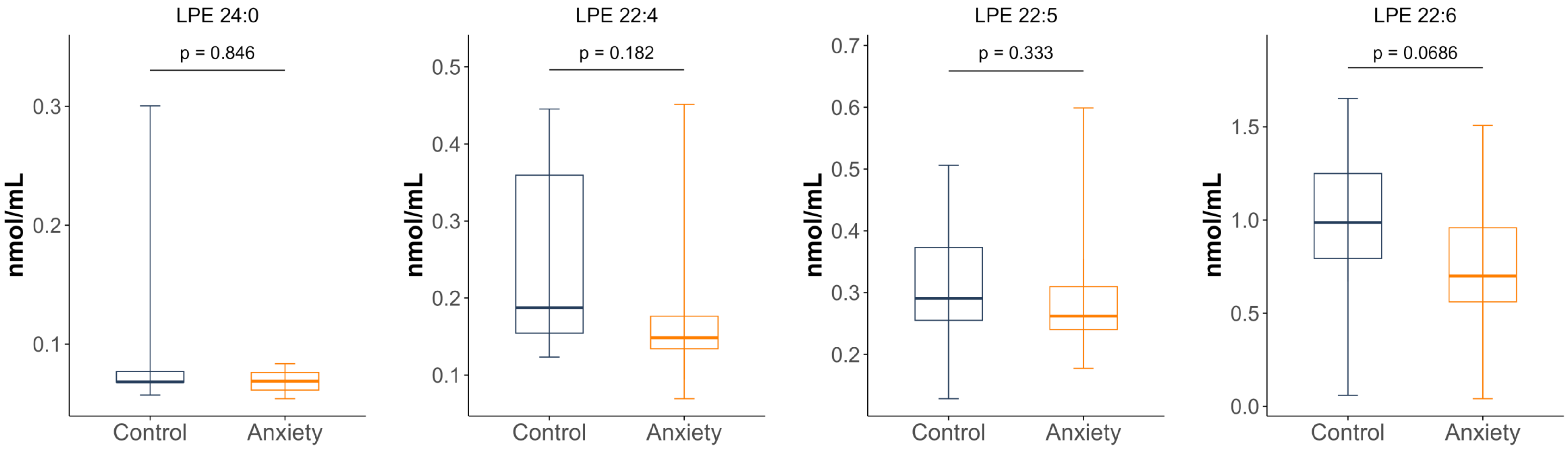

Supplementary Figure 14. Plasma lysophophatidylethanolamine species profile. Results are presented as box-and-whisker plots showing the median, interquartile range, and 5th–95th percentiles. Differences between group were assessed using the Mann–Whitney U test. Control (n = 17), Anxiety (n = 17).

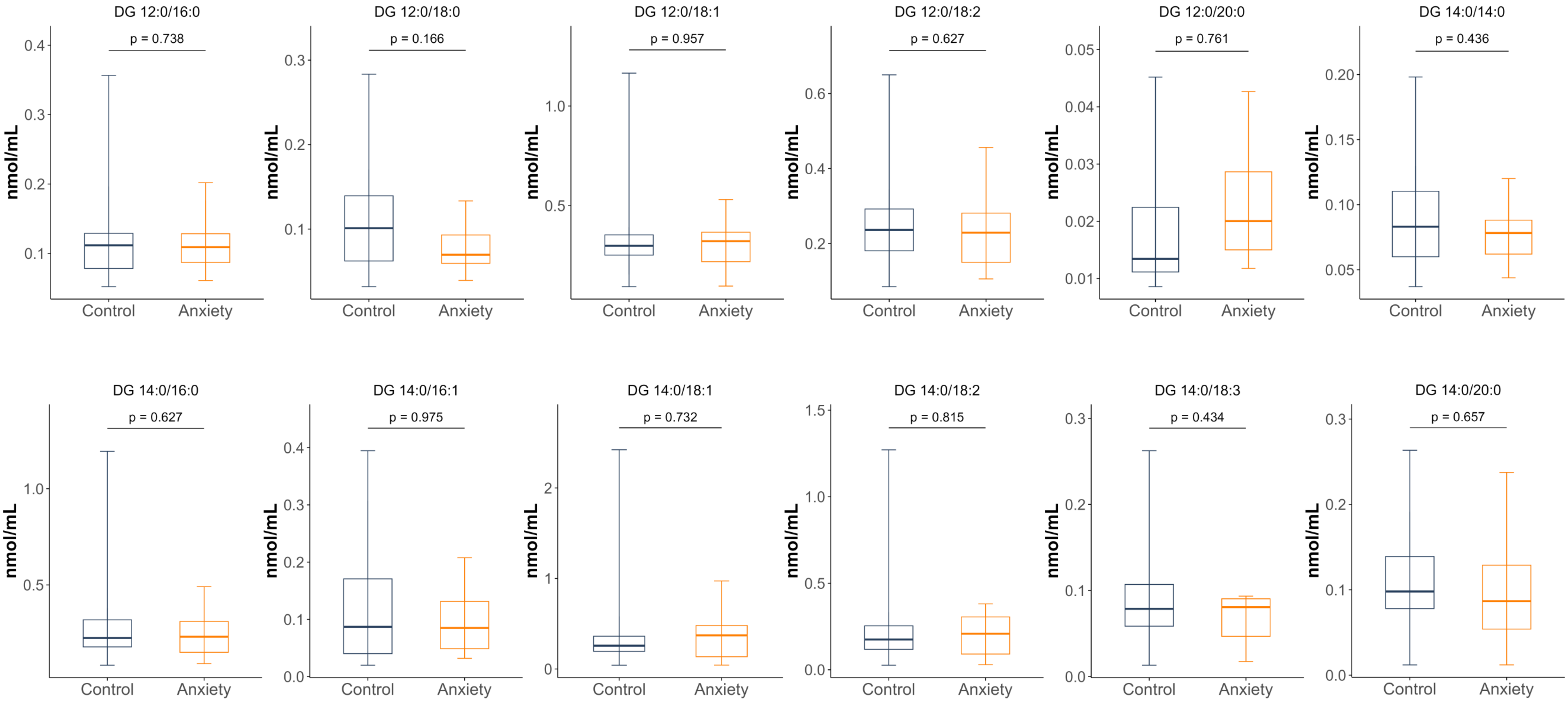

Supplementary Figure 15. Plasma diacylglycerol species profile. Results are presented as box-and-whisker plots showing the median, interquartile range, and 5th–95th percentiles. Differences between group were assessed using the Mann–Whitney U test. Control (n = 17), Anxiety (n = 17).

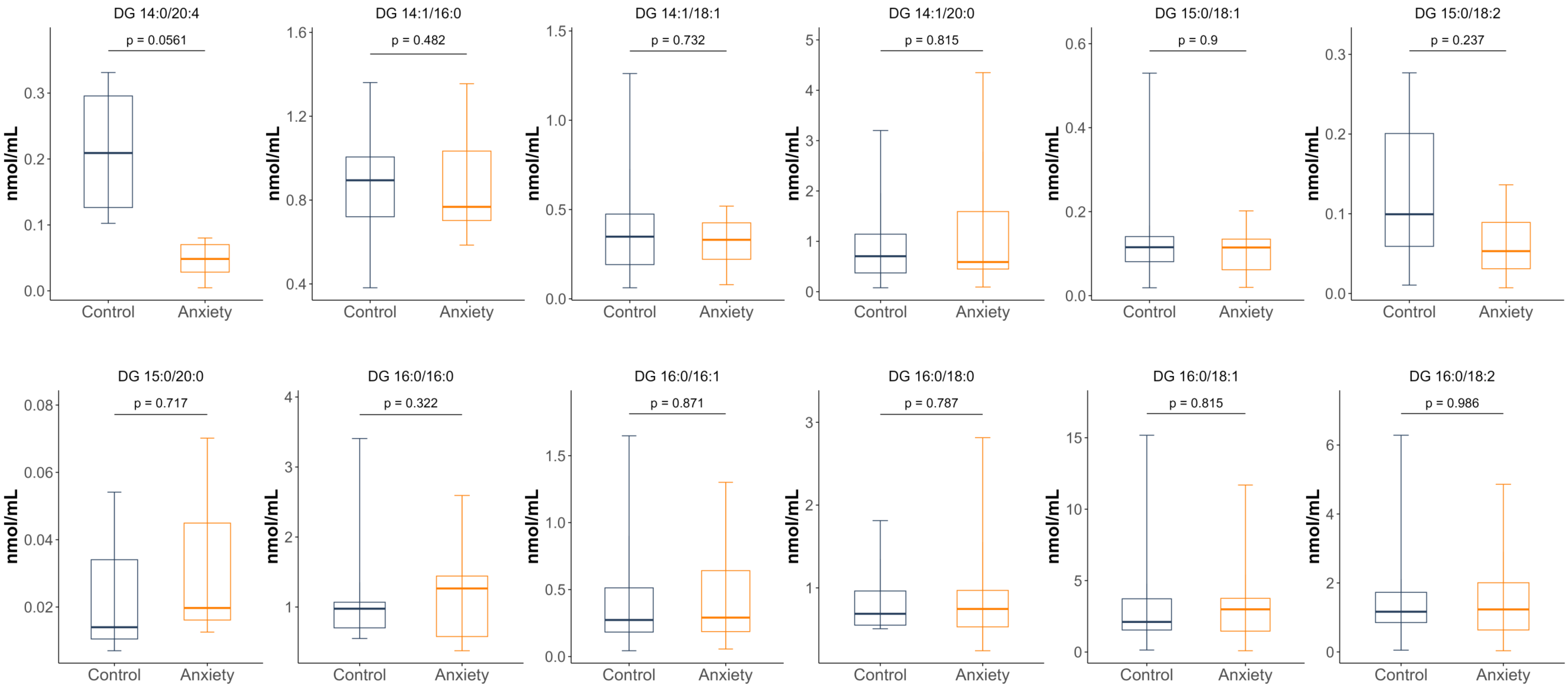

Supplementary Figure 16. Plasma diacylglycerol species profile. Results are presented as box-and-whisker plots showing the median, interquartile range, and 5th–95th percentiles. Differences between group were assessed using the Mann–Whitney U test. Control (n = 17), Anxiety (n = 17).

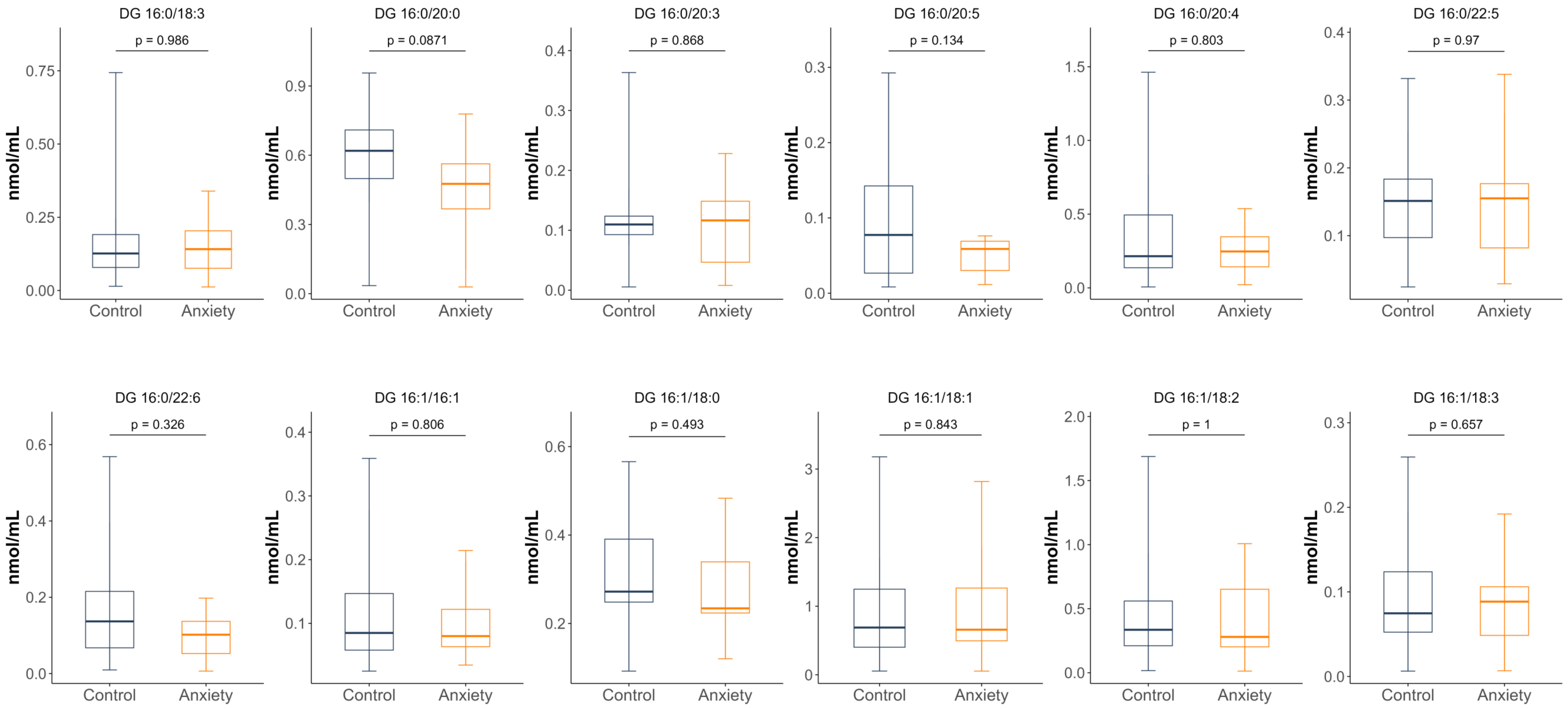

Supplementary Figure 17. Plasma diacylglycerol species profile. Results are presented as box-and-whisker plots showing the median, interquartile range, and 5th–95th percentiles. Differences between group were assessed using the Mann–Whitney U test. Control (n = 17), Anxiety (n = 17).

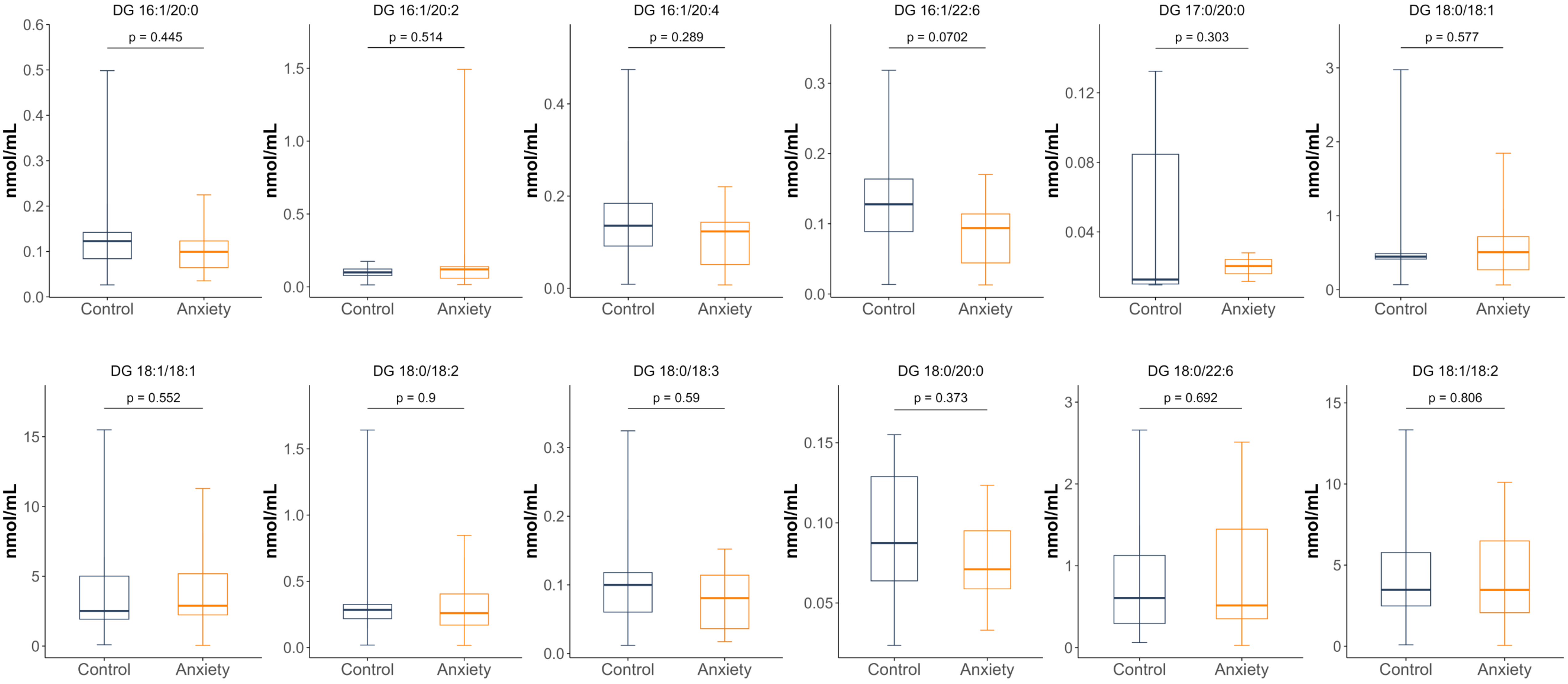

Supplementary Figure 18. Plasma diacylglycerol species profile. Results are presented as box-and-whisker plots showing the median, interquartile range, and 5th–95th percentiles. Differences between group were assessed using the Mann–Whitney U test. Control (n = 17), Anxiety (n = 17).

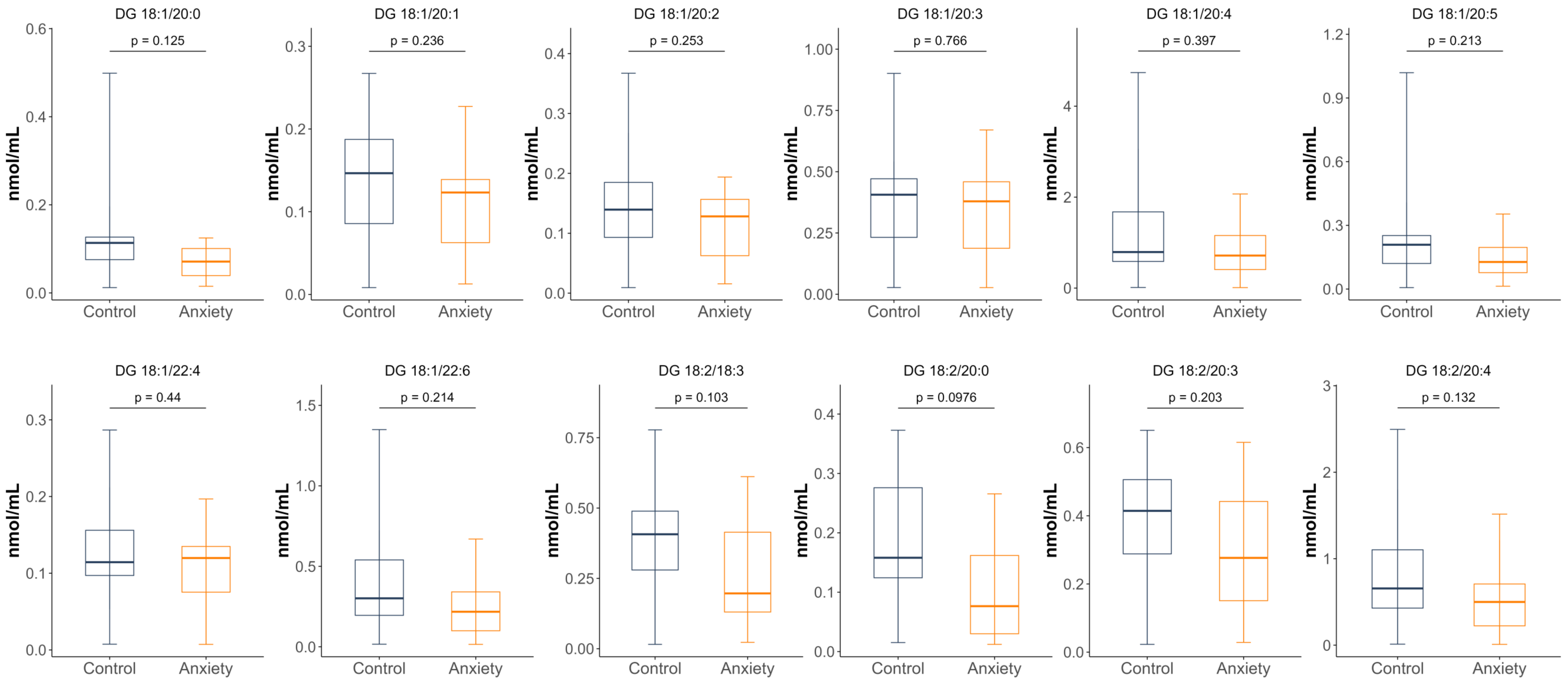

Supplementary Figure 19. Plasma diacylglycerol species profile. Results are presented as box-and-whisker plots showing the median, interquartile range, and 5th–95th percentiles. Differences between group were assessed using the Mann–Whitney U test. Control (n = 17), Anxiety (n = 17).

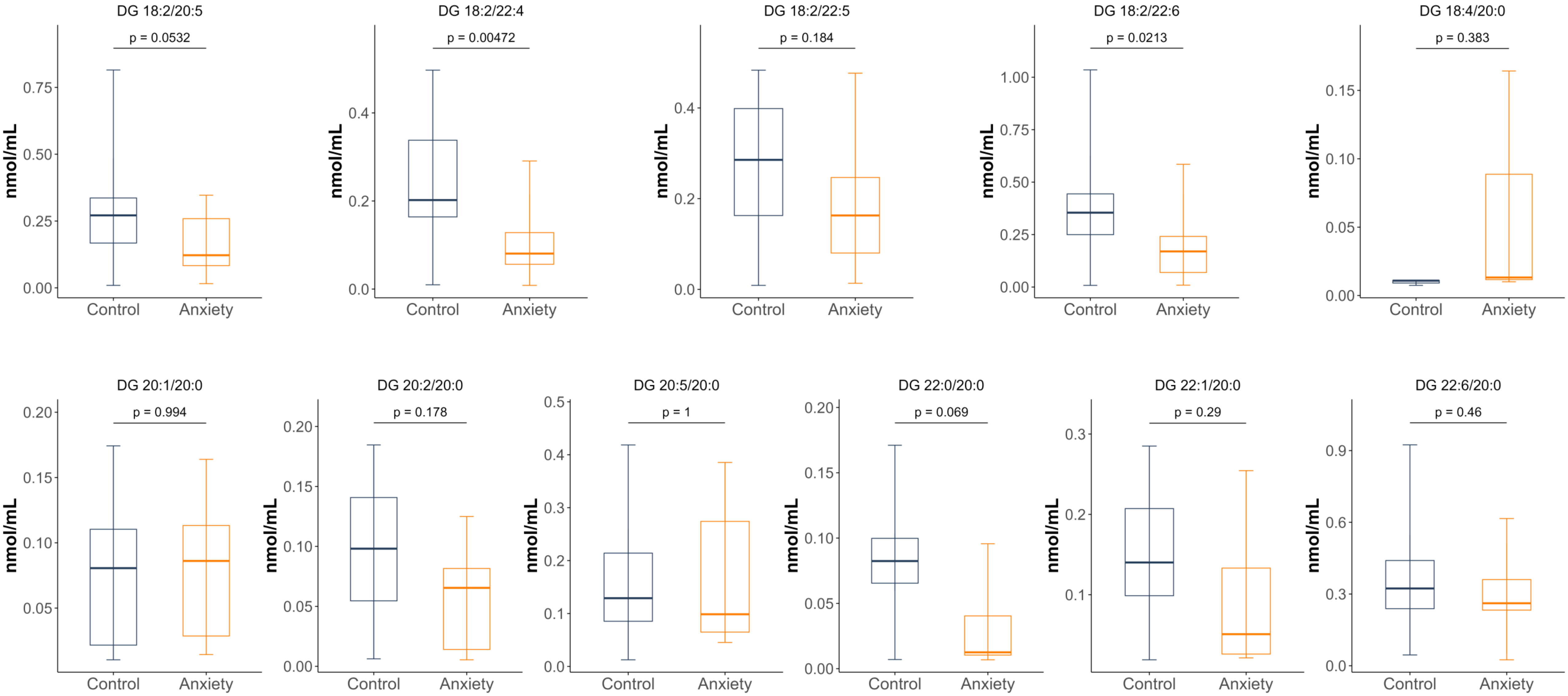

Supplementary Figure 20. Plasma diacylglycerol species profile. Results are presented as box-and-whisker plots showing the median, interquartile range, and 5th–95th percentiles. Differences between group were assessed using the Mann–Whitney U test. Control (n = 17), Anxiety (n = 17).

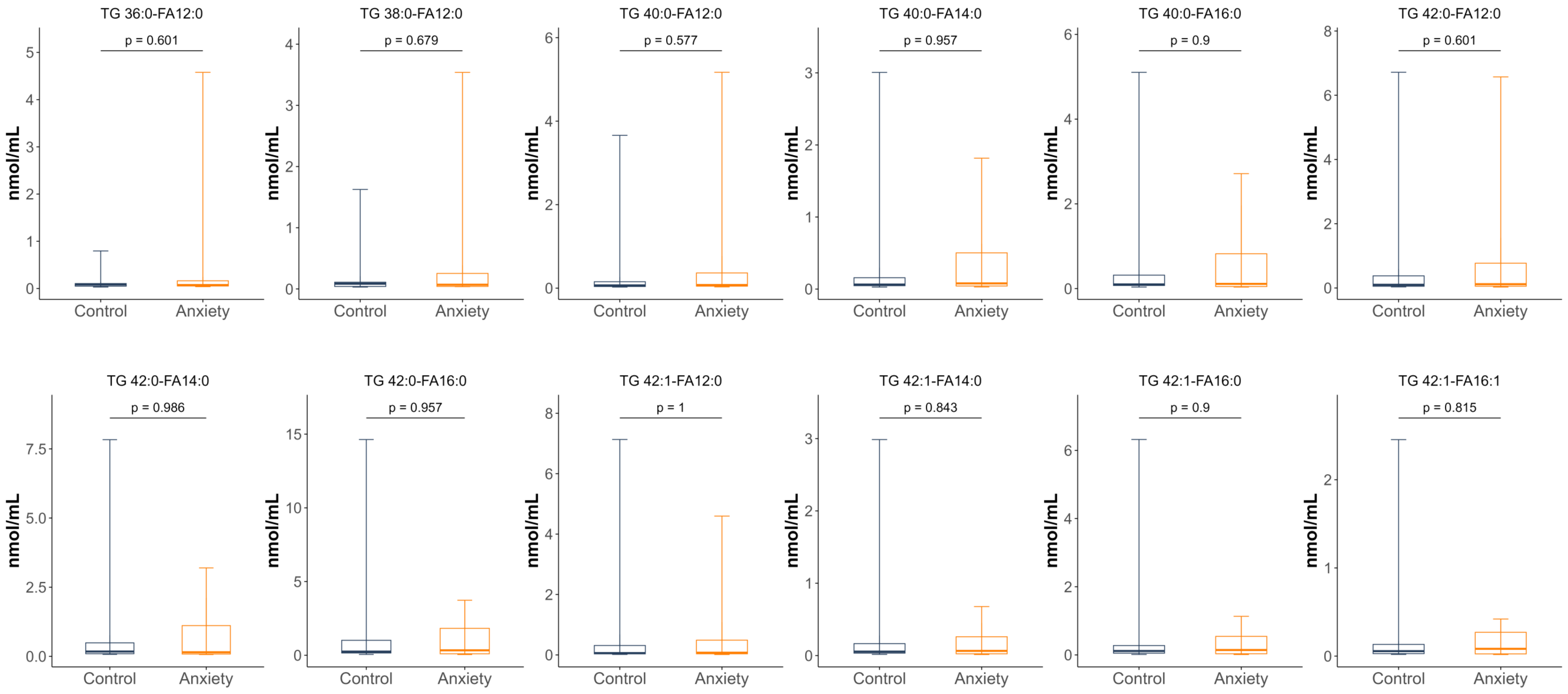

Supplementary Figure 21. Plasma triacylglycerol species profile. Results are presented as box-and-whisker plots showing the median, interquartile range, and 5th–95th percentiles. Differences between group were assessed using the Mann–Whitney U test. Control (n = 17), Anxiety (n = 17).

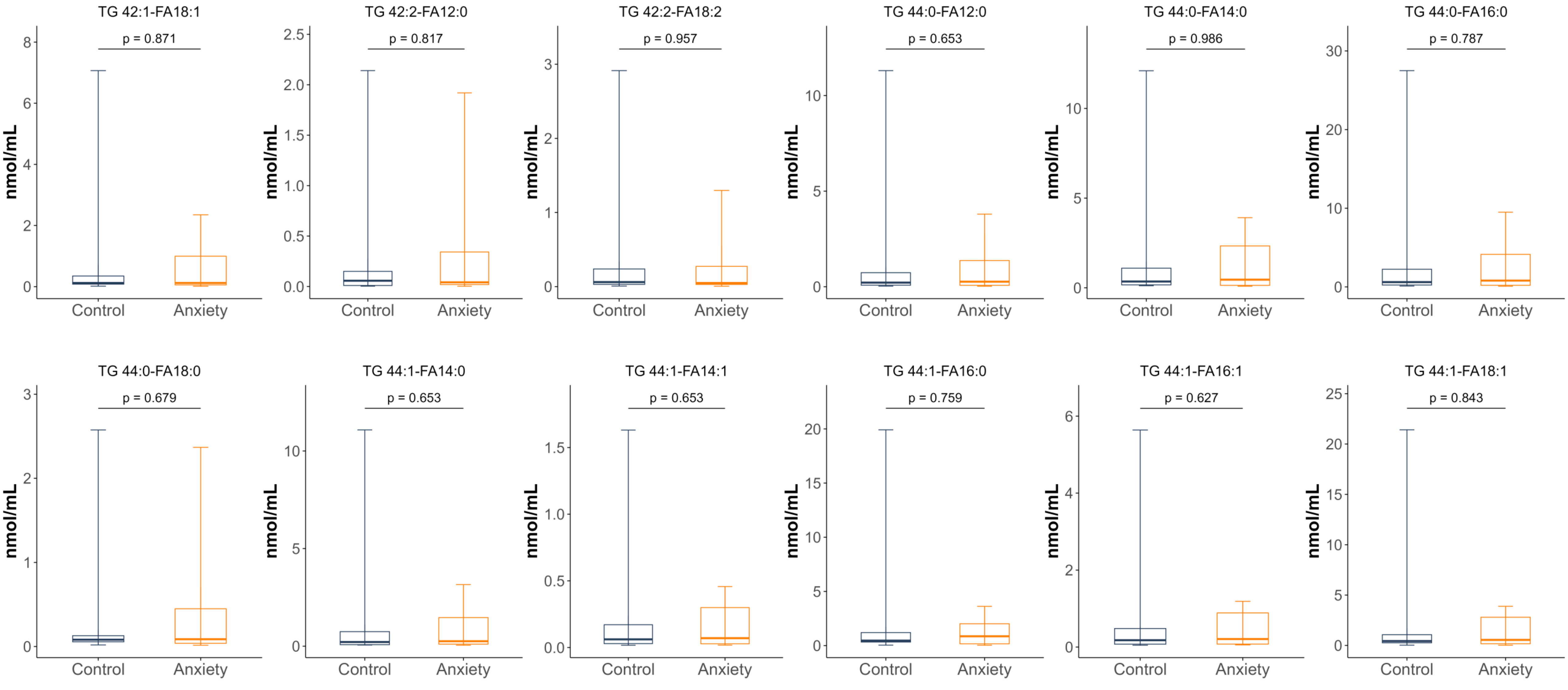

Supplementary Figure 22. Plasma triacylglycerol species profile. Results are presented as box-and-whisker plots showing the median, interquartile range, and 5th–95th percentiles. Differences between group were assessed using the Mann–Whitney U test. Control (n = 17), Anxiety (n = 17).

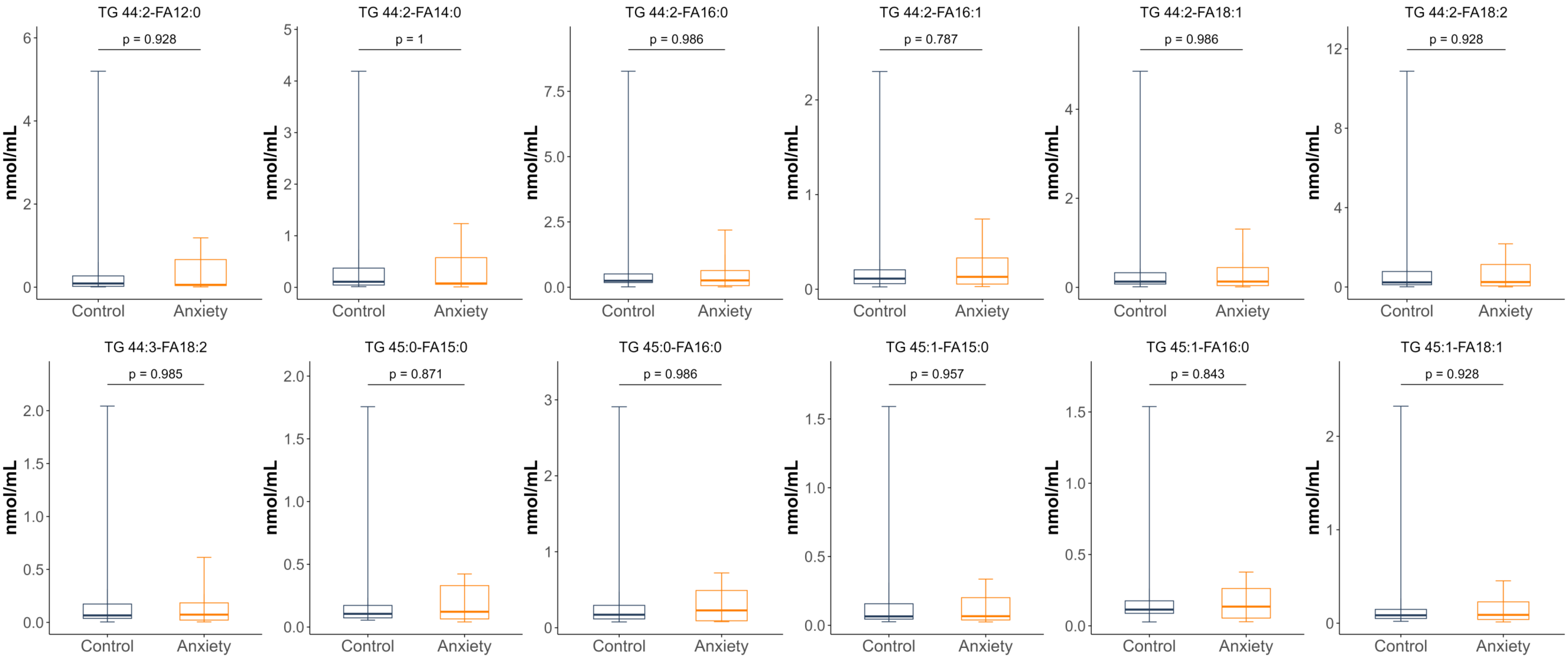

Supplementary Figure 23. Plasma triacylglycerol species profile. Results are presented as box-and-whisker plots showing the median, interquartile range, and 5th–95th percentiles. Differences between group were assessed using the Mann–Whitney U test. Control (n = 17), Anxiety (n = 17).

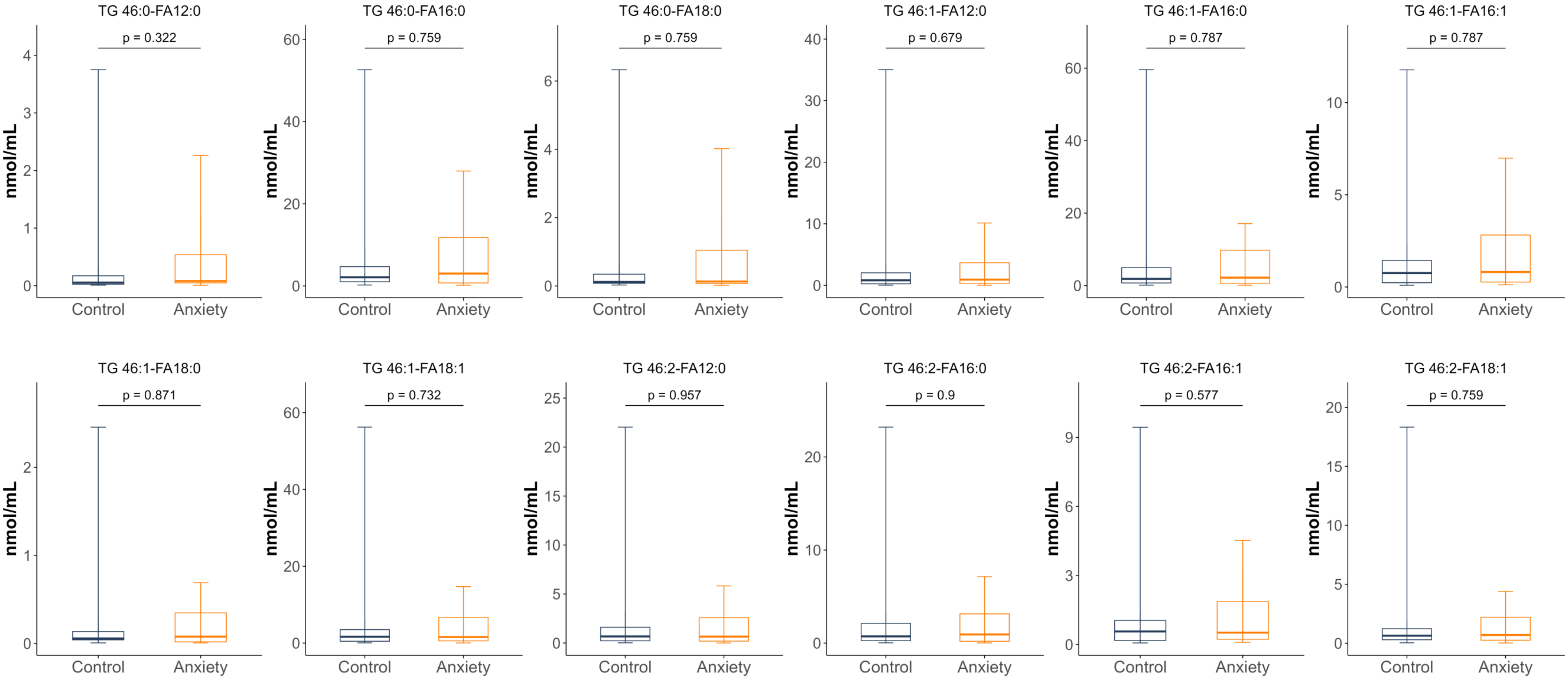

Supplementary Figure 24. Plasma triacylglycerol species profile. Results are presented as box-and-whisker plots showing the median, interquartile range, and 5th–95th percentiles. Differences between group were assessed using the Mann–Whitney U test. Control (n = 17), Anxiety (n = 17).

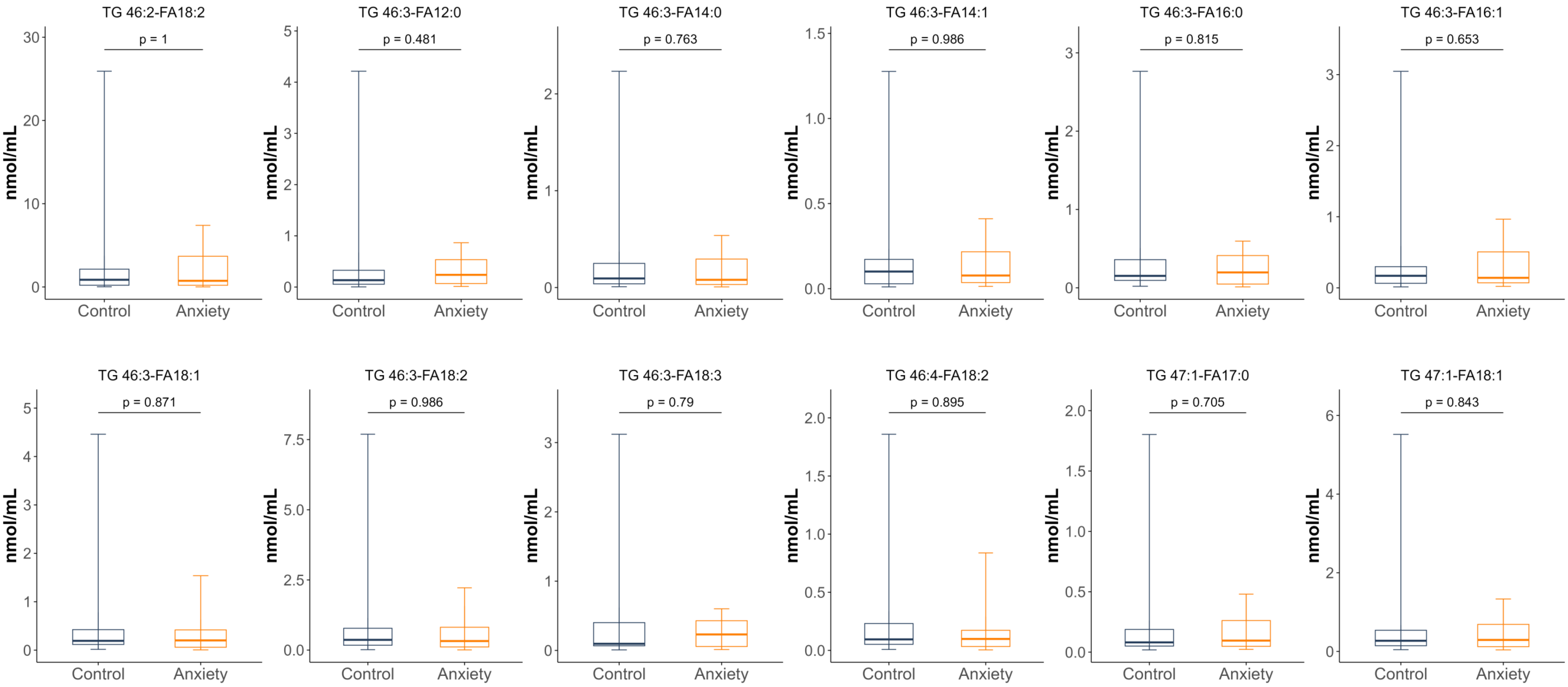

Supplementary Figure 25. Plasma triacylglycerol species profile. Results are presented as box-and-whisker plots showing the median, interquartile range, and 5th–95th percentiles. Differences between group were assessed using the Mann–Whitney U test. Control (n = 17), Anxiety (n = 17).

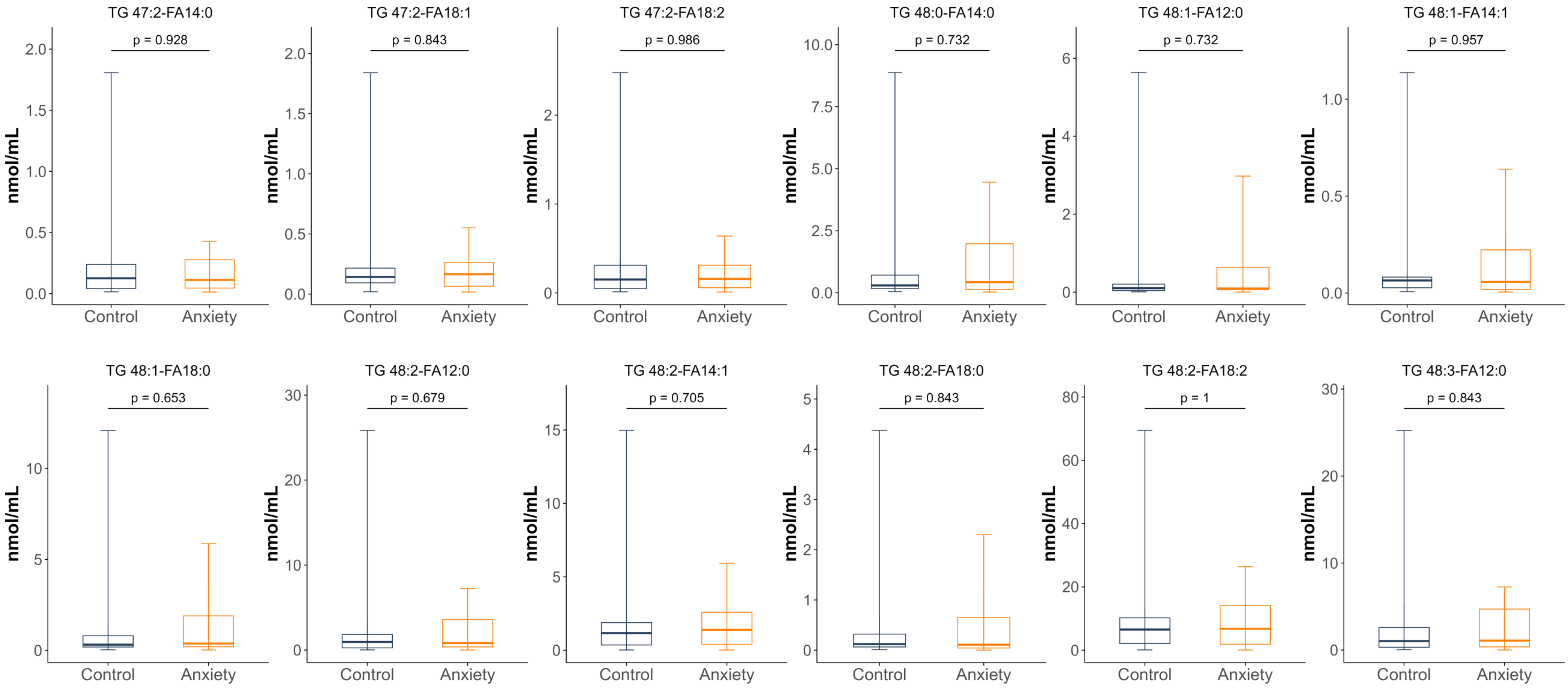

Supplementary Figure 26. Plasma triacylglycerol species profile. Results are presented as box-and-whisker plots showing the median, interquartile range, and 5th–95th percentiles. Differences between group were assessed using the Mann–Whitney U test. Control (n = 17), Anxiety (n = 17).

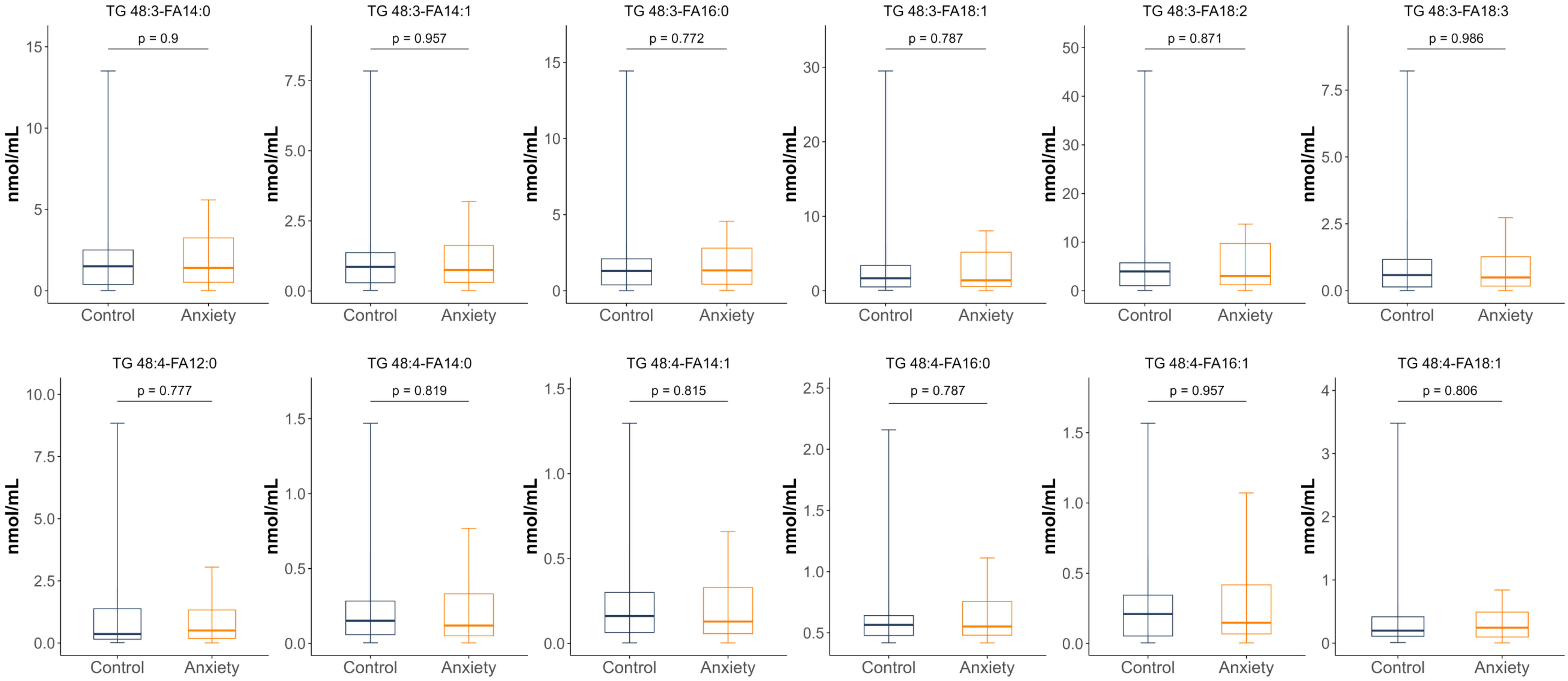

Supplementary Figure 27. Plasma triacylglycerol species profile. Results are presented as box-and-whisker plots showing the median, interquartile range, and 5th–95th percentiles. Differences between group were assessed using the Mann–Whitney U test. Control (n = 17), Anxiety (n = 17).

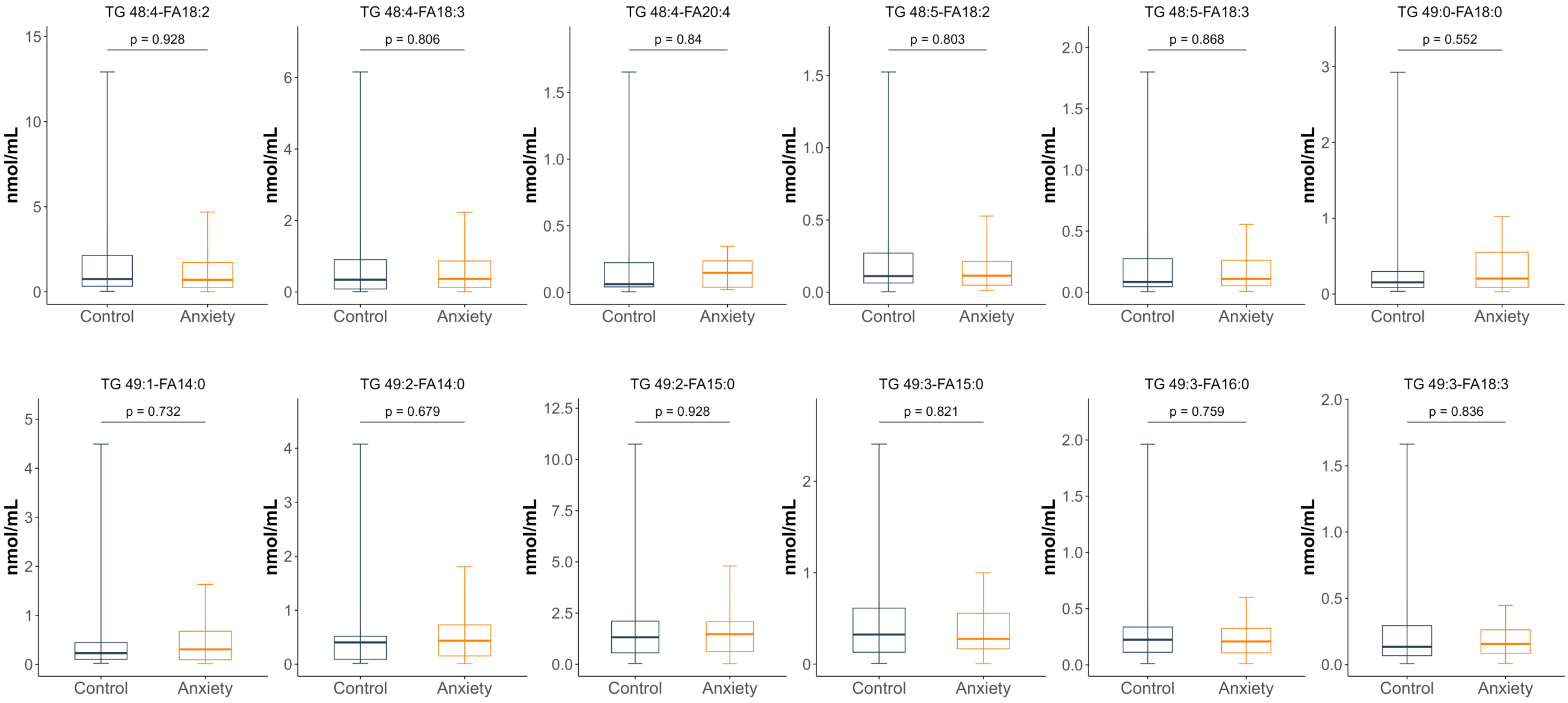

Supplementary Figure 28. Plasma triacylglycerol species profile. Results are presented as box-and-whisker plots showing the median, interquartile range, and 5th–95th percentiles. Differences between group were assessed using the Mann–Whitney U test. Control (n = 17), Anxiety (n = 17).

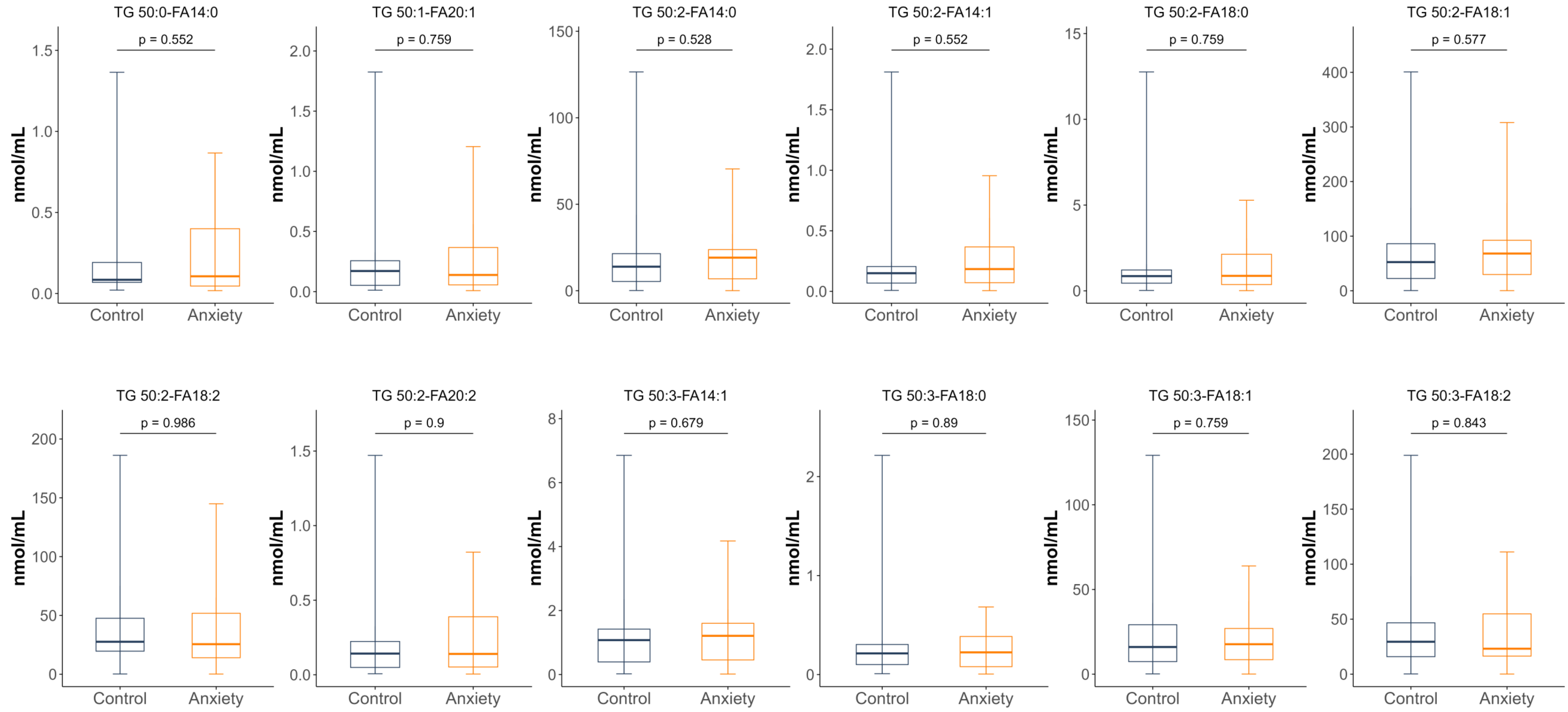

Supplementary Figure 29. Plasma triacylglycerol species profile. Results are presented as box-and-whisker plots showing the median, interquartile range, and 5th–95th percentiles. Differences between group were assessed using the Mann–Whitney U test. Control (n = 17), Anxiety (n = 17).

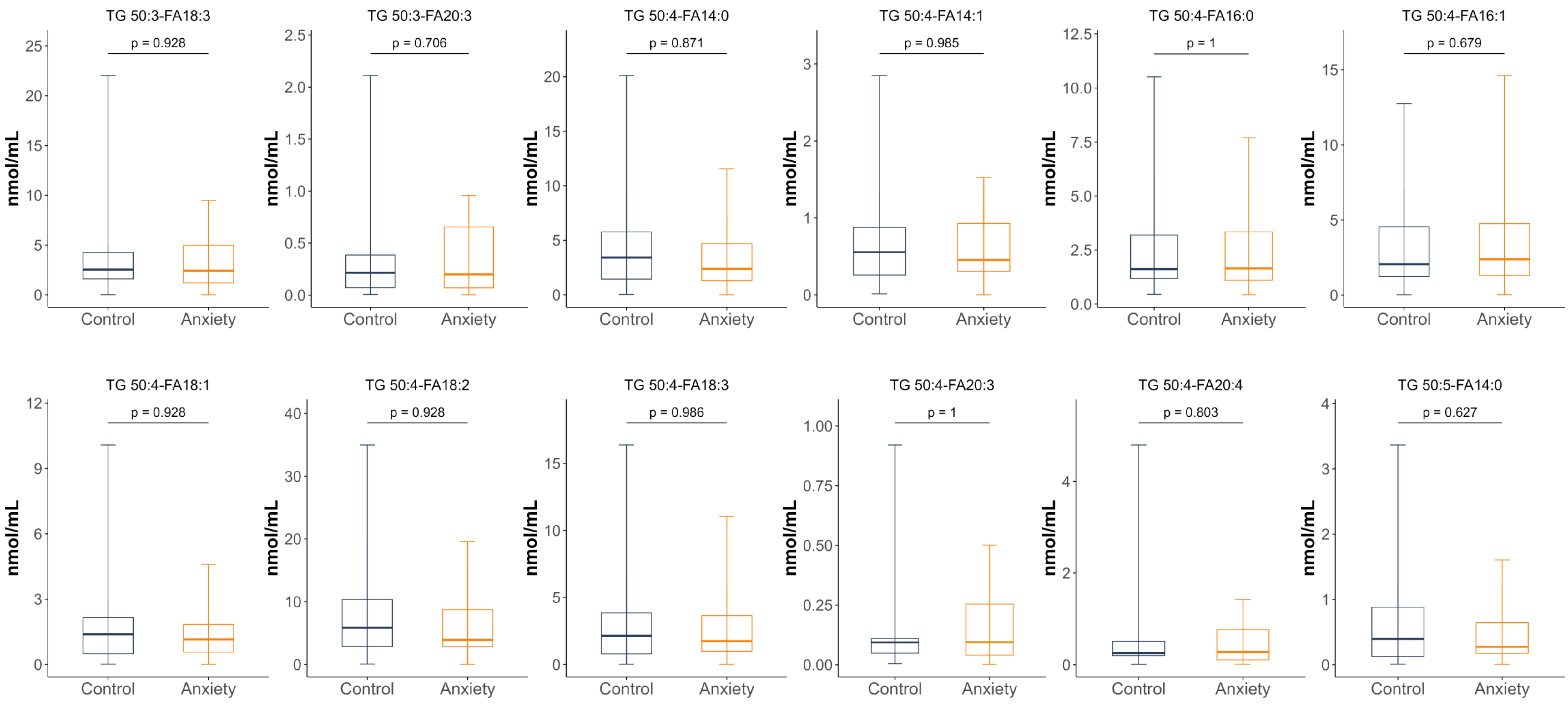

Supplementary Figure 30. Plasma triacylglycerol species profile. Results are presented as box-and-whisker plots showing the median, interquartile range, and 5th–95th percentiles. Differences between group were assessed using the Mann–Whitney U test. Control (n = 17), Anxiety (n = 17).

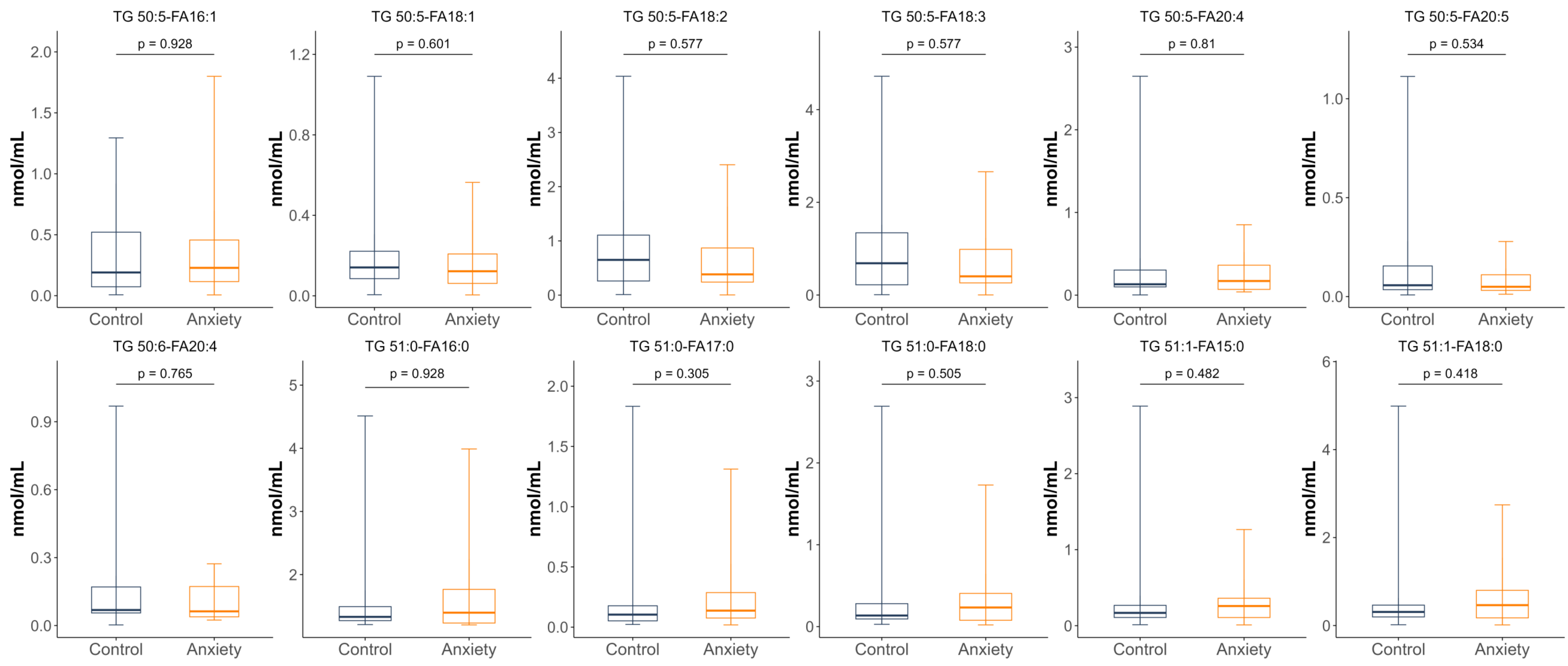

Supplementary Figure 31. Plasma triacylglycerol species profile. Results are presented as box-and-whisker plots showing the median, interquartile range, and 5th–95th percentiles. Differences between group were assessed using the Mann–Whitney U test. Control (n = 17), Anxiety (n = 17).

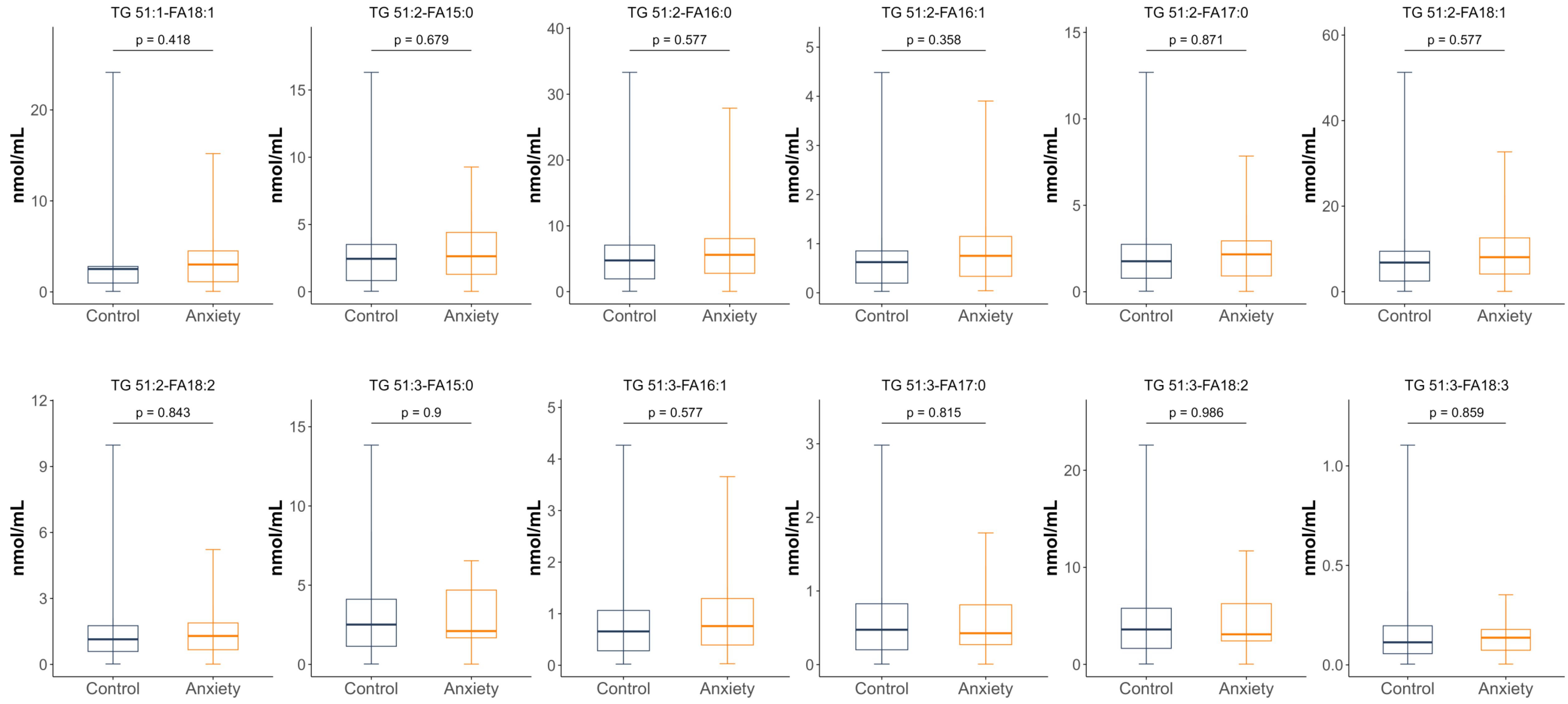

Supplementary Figure 32. Plasma triacylglycerol species profile. Results are presented as box-and-whisker plots showing the median, interquartile range, and 5th–95th percentiles. Differences between group were assessed using the Mann–Whitney U test. Control (n = 17), Anxiety (n = 17).

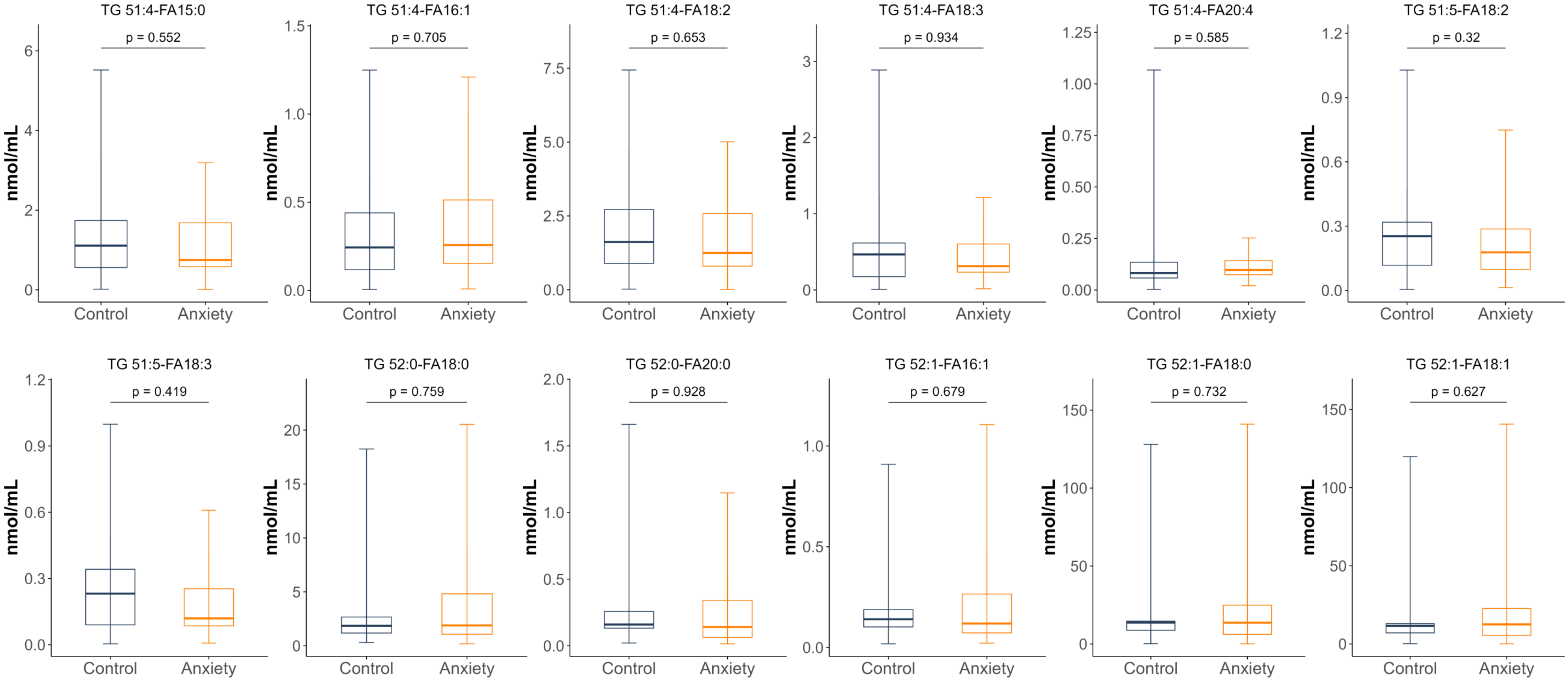

Supplementary Figure 33. Plasma triacylglycerol species profile. Results are presented as box-and-whisker plots showing the median, interquartile range, and 5th–95th percentiles. Differences between group were assessed using the Mann–Whitney U test. Control (n = 17), Anxiety (n = 17).

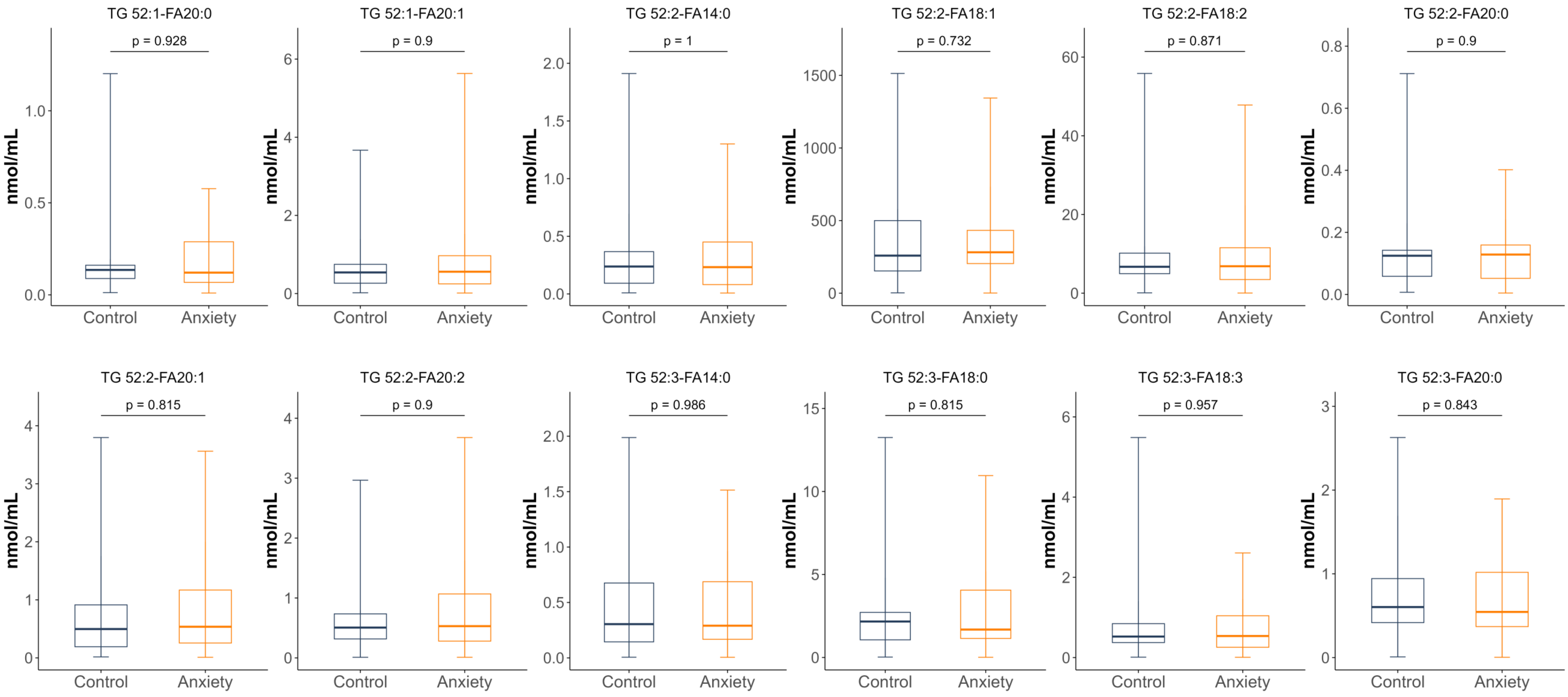

Supplementary Figure 34. Plasma triacylglycerol species profile. Results are presented as box-and-whisker plots showing the median, interquartile range, and 5th–95th percentiles. Differences between group were assessed using the Mann–Whitney U test. Control (n = 17), Anxiety (n = 17).

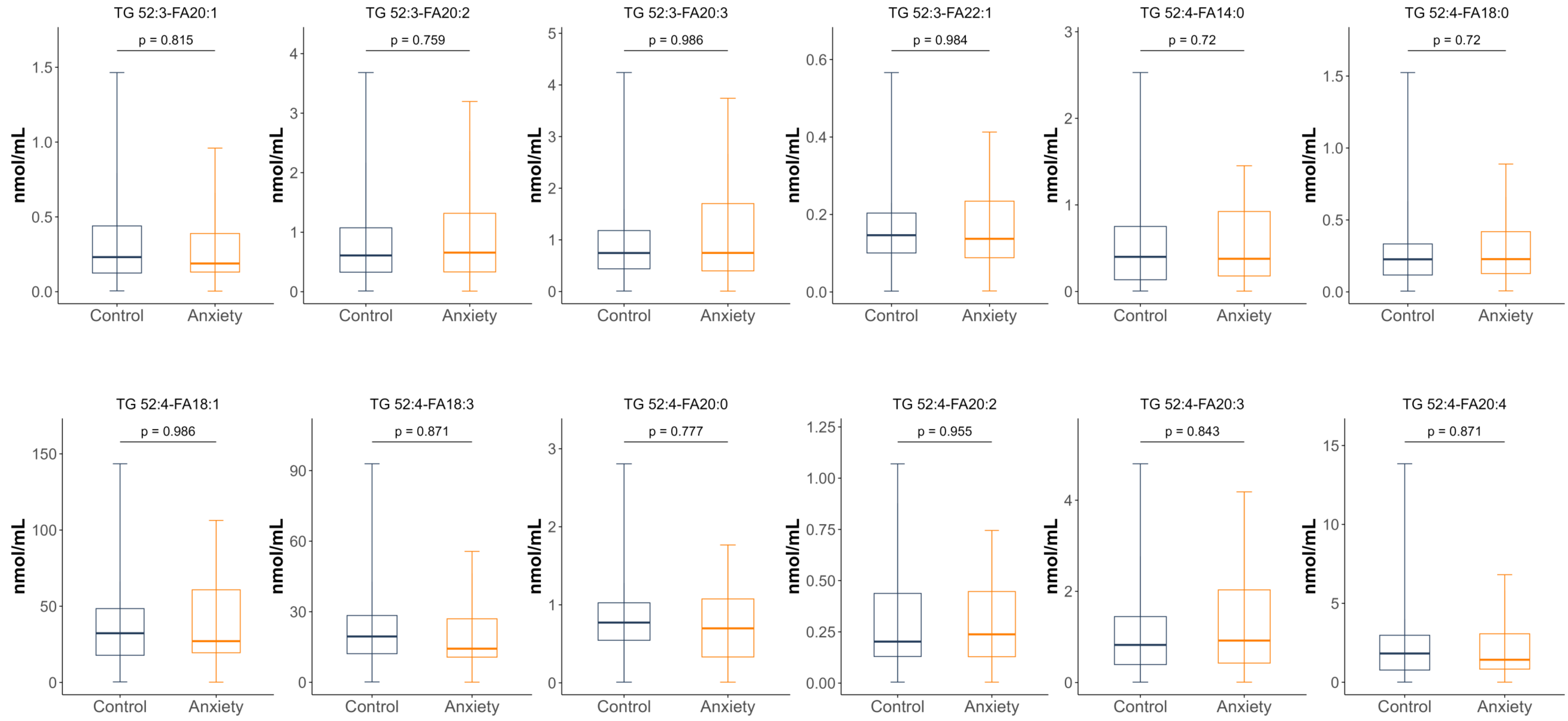

Supplementary Figure 35. Plasma triacylglycerol species profile. Results are presented as box-and-whisker plots showing the median, interquartile range, and 5th–95th percentiles. Differences between group were assessed using the Mann–Whitney U test. Control (n = 17), Anxiety (n = 17).

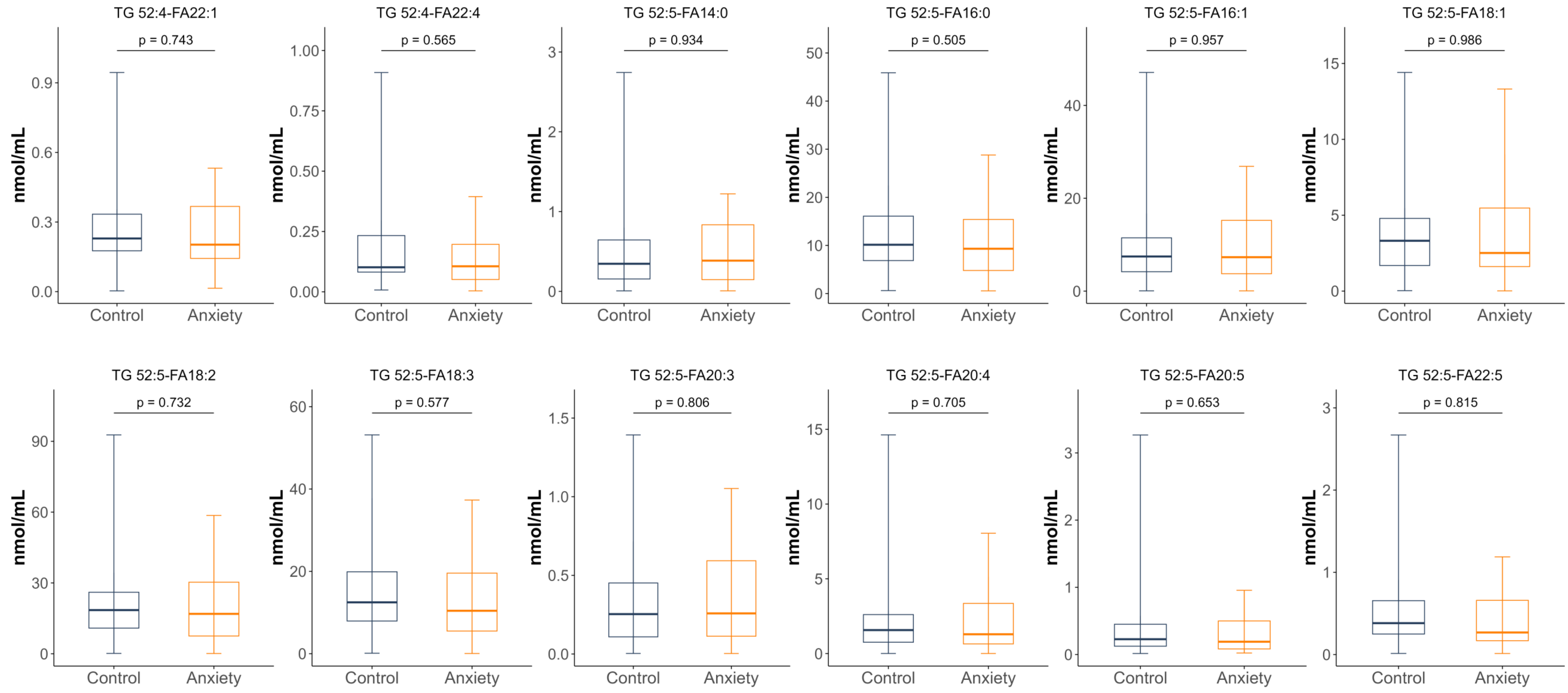

Supplementary Figure 36. Plasma triacylglycerol species profile. Results are presented as box-and-whisker plots showing the median, interquartile range, and 5th–95th percentiles. Differences between group were assessed using the Mann–Whitney U test. Control (n = 17), Anxiety (n = 17).

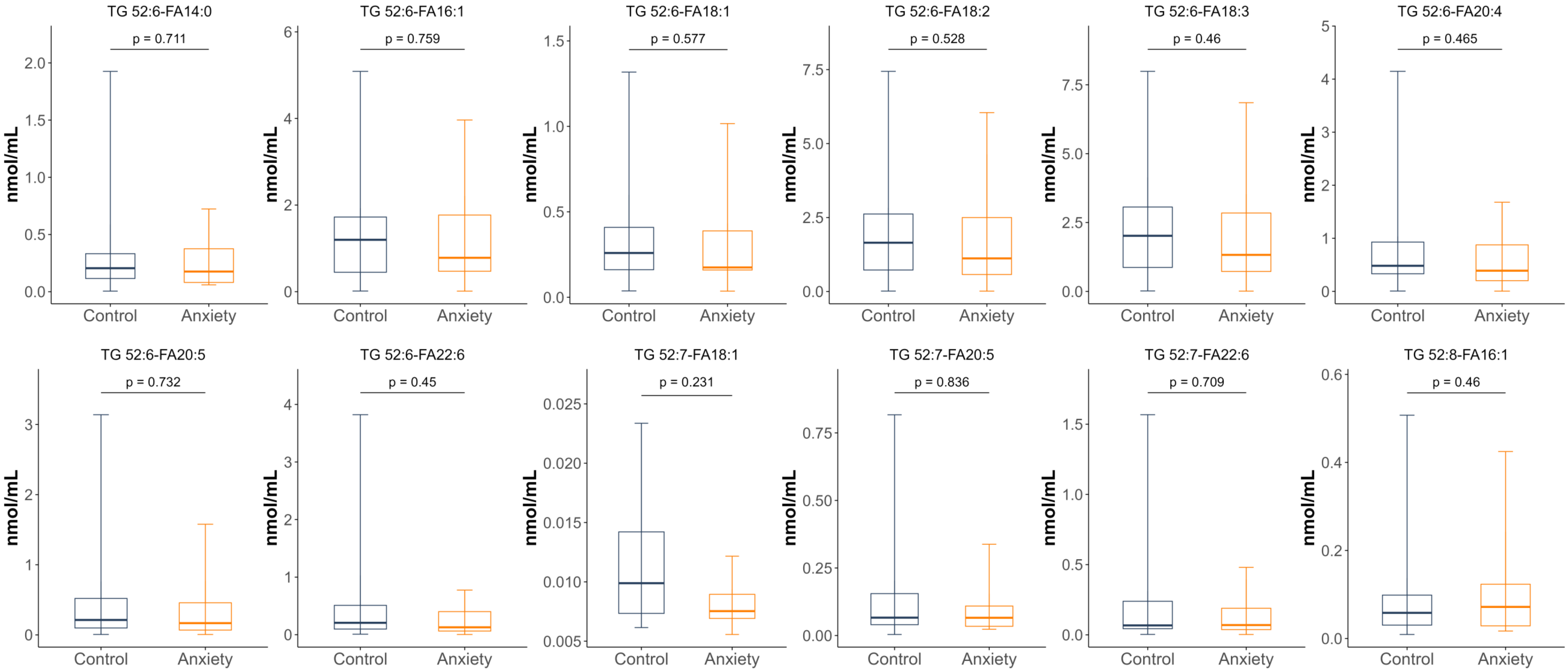

Supplementary Figure 37. Plasma triacylglycerol species profile. Results are presented as box-and-whisker plots showing the median, interquartile range, and 5th–95th percentiles. Differences between group were assessed using the Mann–Whitney U test. Control (n = 17), Anxiety (n = 17).

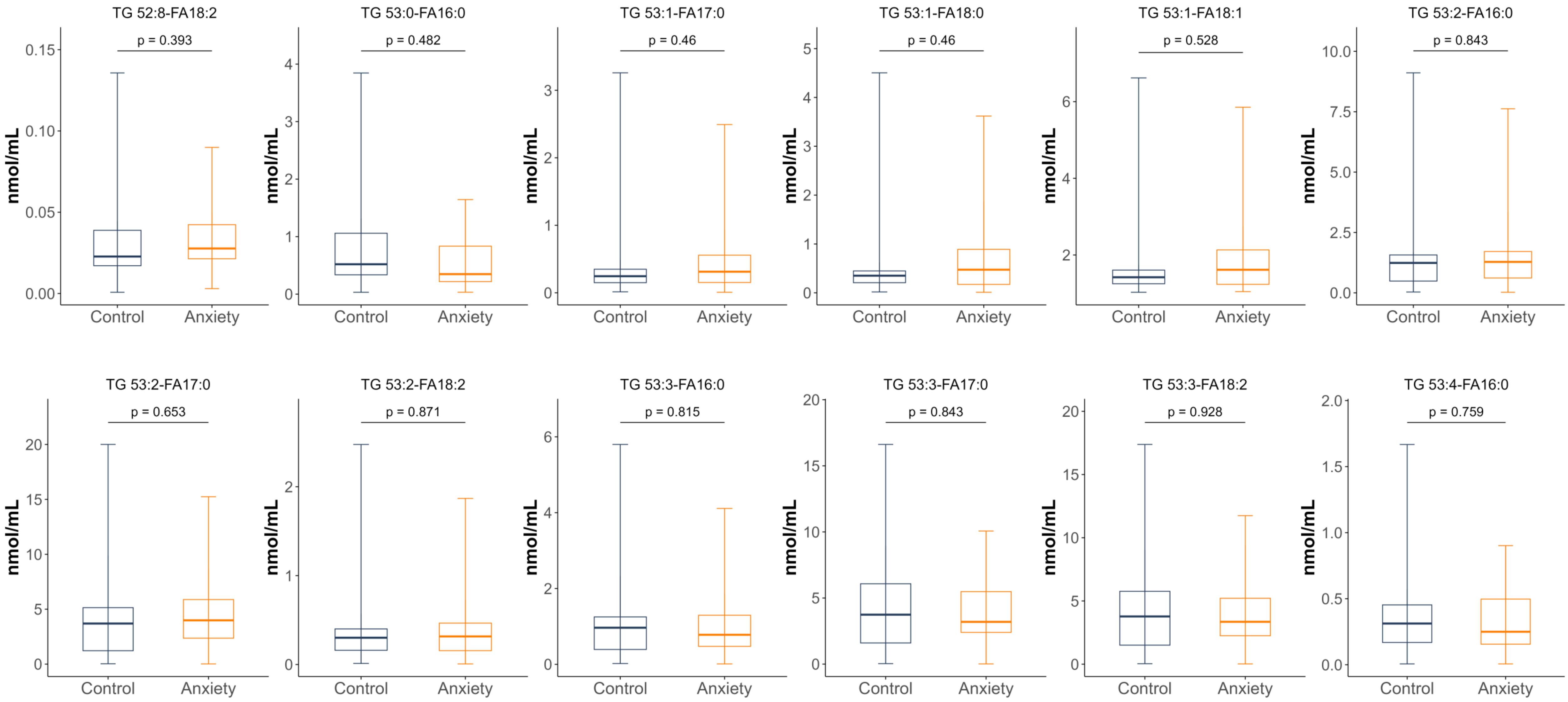

Supplementary Figure 38. Plasma triacylglycerol species profile. Results are presented as box-and-whisker plots showing the median, interquartile range, and 5th–95th percentiles. Differences between group were assessed using the Mann–Whitney U test. Control (n = 17), Anxiety (n = 17).

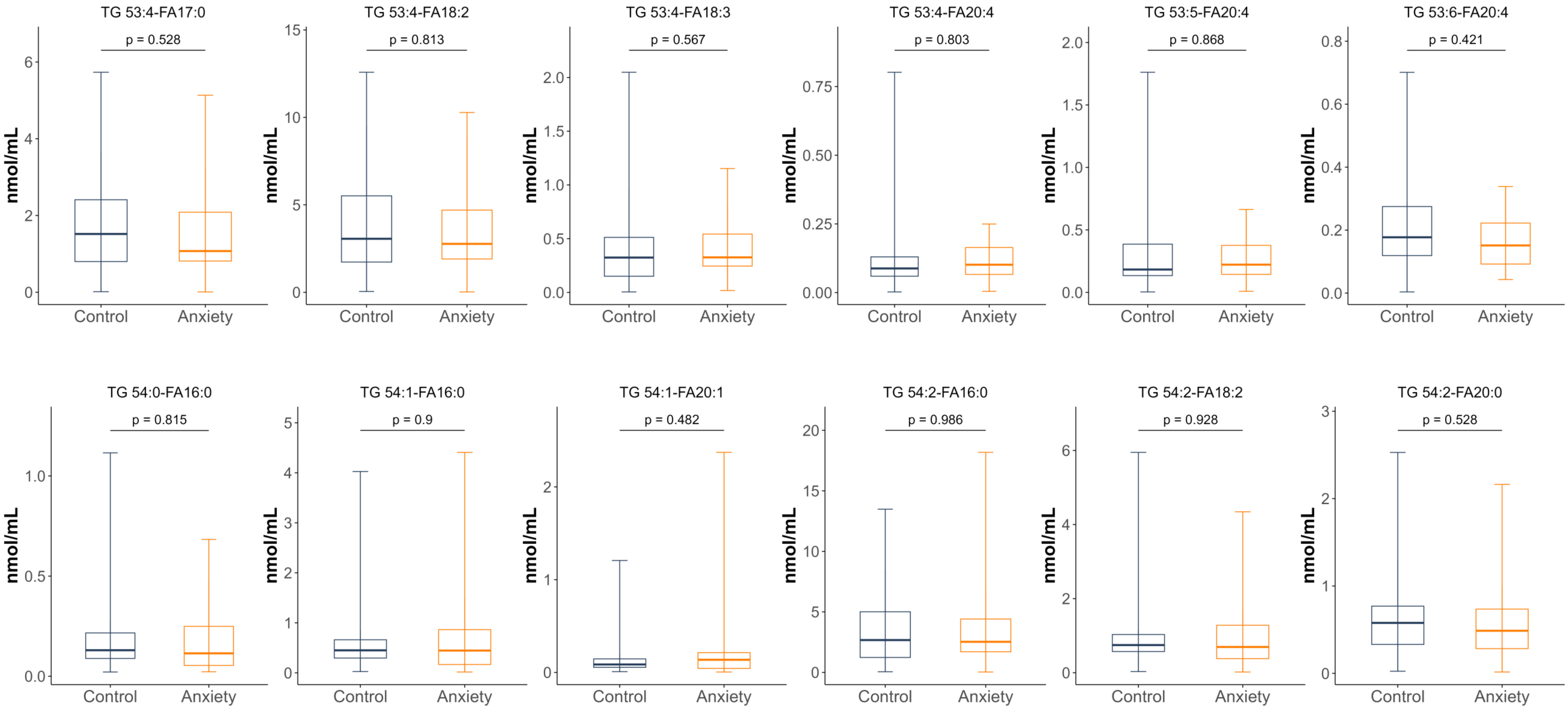

Supplementary Figure 39. Plasma triacylglycerol species profile. Results are presented as box-and-whisker plots showing the median, interquartile range, and 5th–95th percentiles. Differences between group were assessed using the Mann–Whitney U test. Control (n = 17), Anxiety (n = 17).

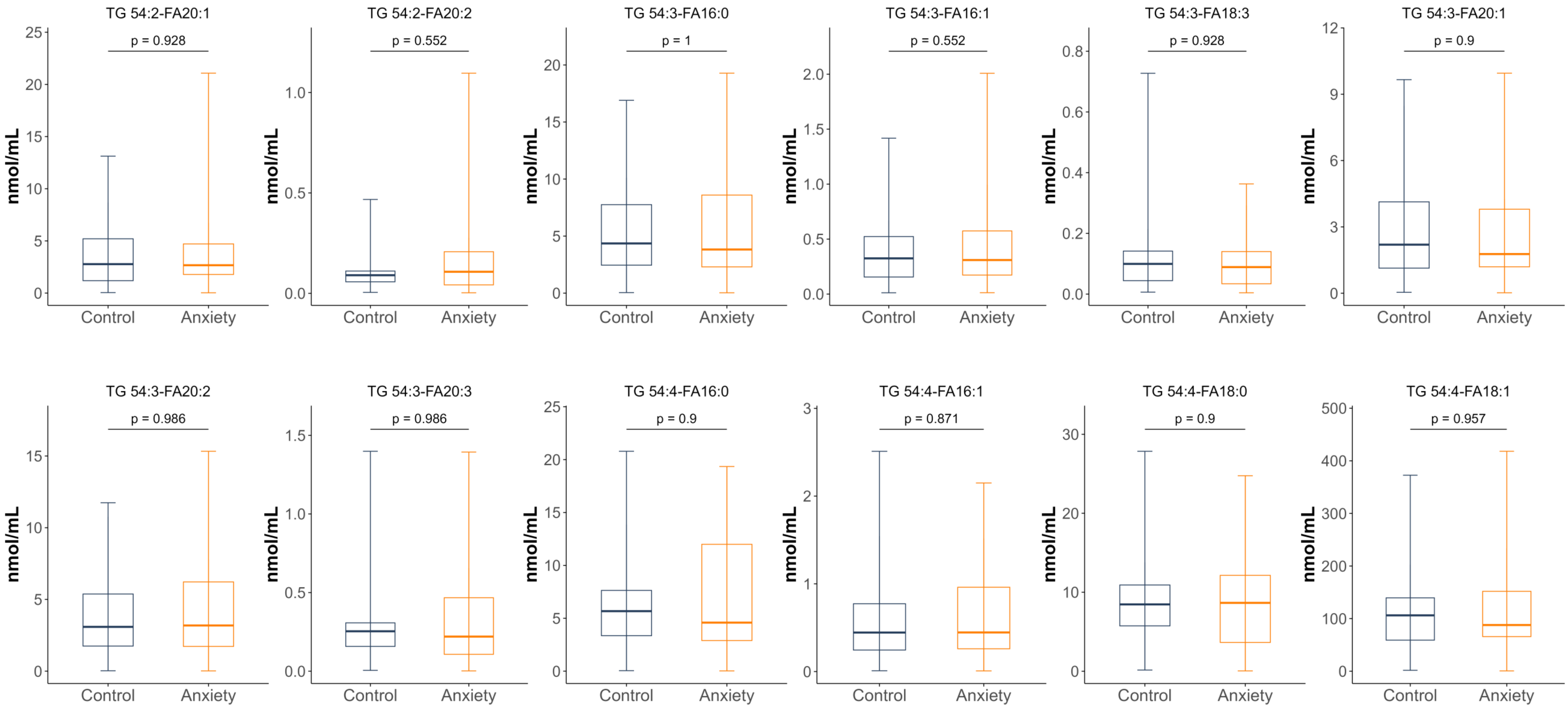

Supplementary Figure 40. Plasma triacylglycerol species profile. Results are presented as box-and-whisker plots showing the median, interquartile range, and 5th–95th percentiles. Differences between group were assessed using the Mann–Whitney U test. Control (n = 17), Anxiety (n = 17).

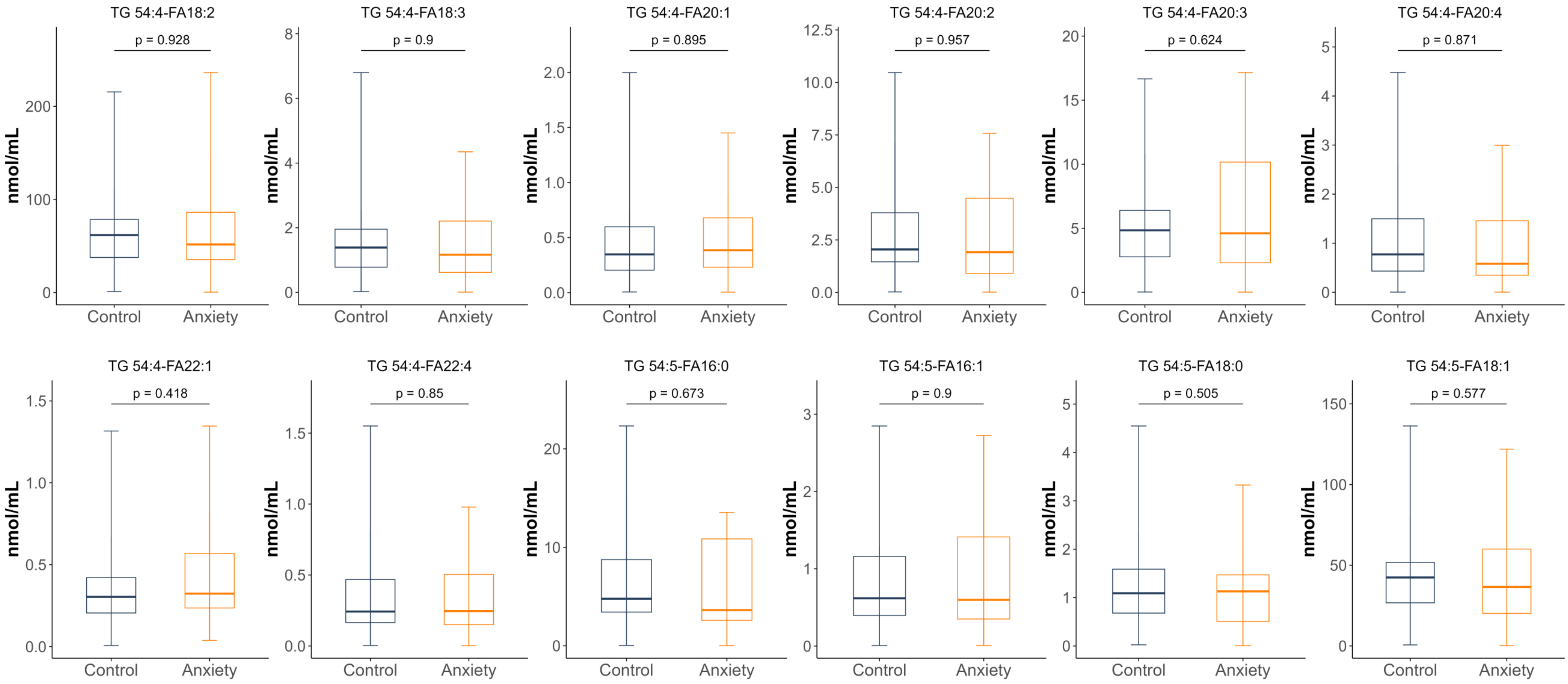

Supplementary Figure 41. Plasma triacylglycerol species profile. Results are presented as box-and-whisker plots showing the median, interquartile range, and 5th–95th percentiles. Differences between group were assessed using the Mann–Whitney U test. Control (n = 17), Anxiety (n = 17).

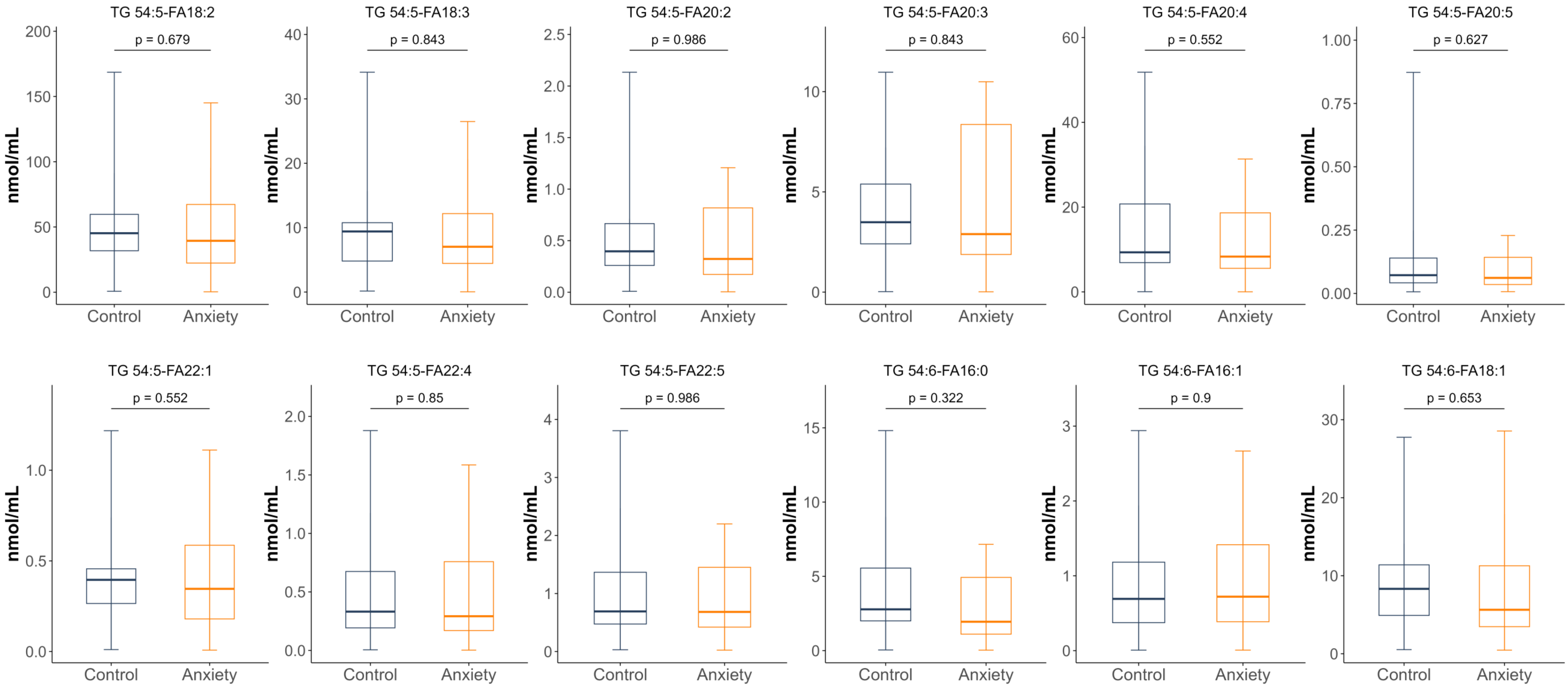

Supplementary Figure 42. Plasma triacylglycerol species profile. Results are presented as box-and-whisker plots showing the median, interquartile range, and 5th–95th percentiles. Differences between group were assessed using the Mann–Whitney U test. Control (n = 17), Anxiety (n = 17).

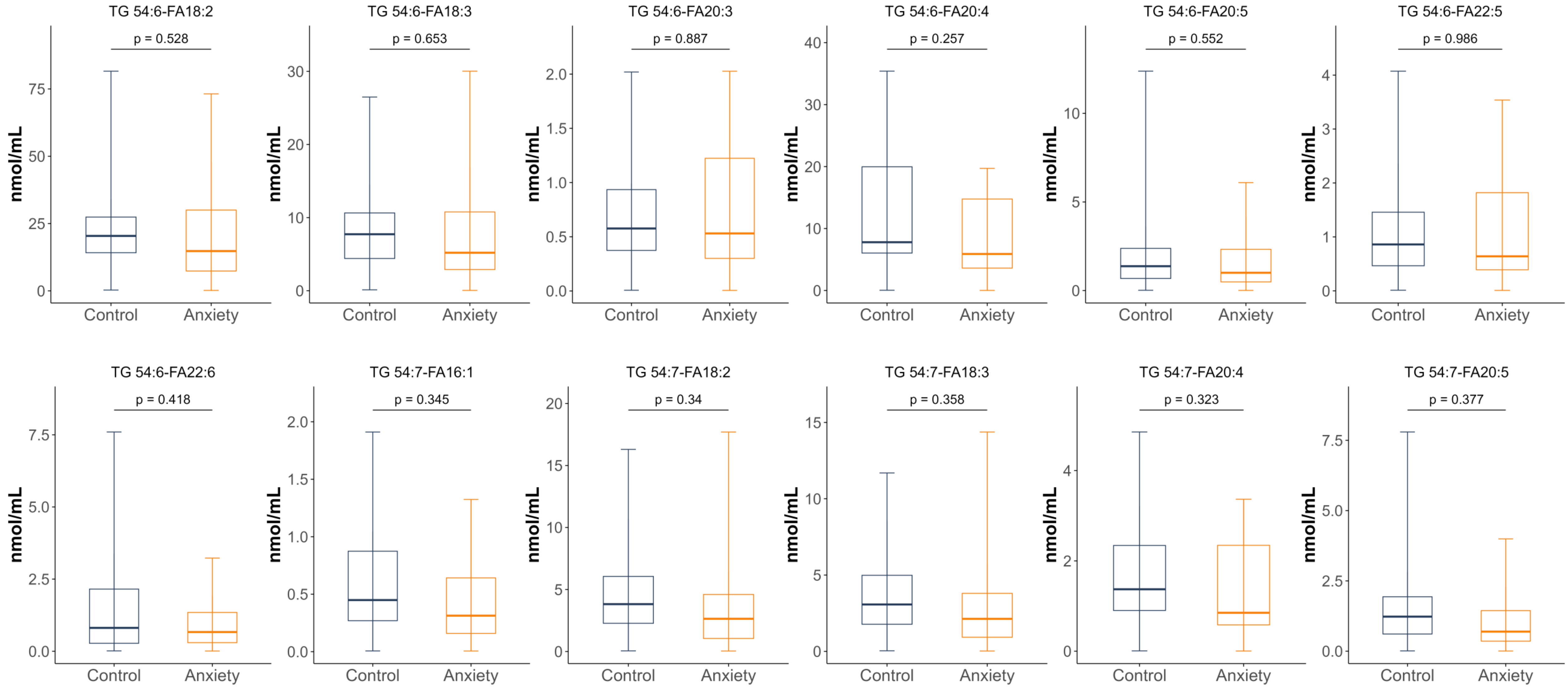

Supplementary Figure 43. Plasma triacylglycerol species profile. Results are presented as box-and-whisker plots showing the median, interquartile range, and 5th–95th percentiles. Differences between group were assessed using the Mann–Whitney U test. Control (n = 17), Anxiety (n = 17).

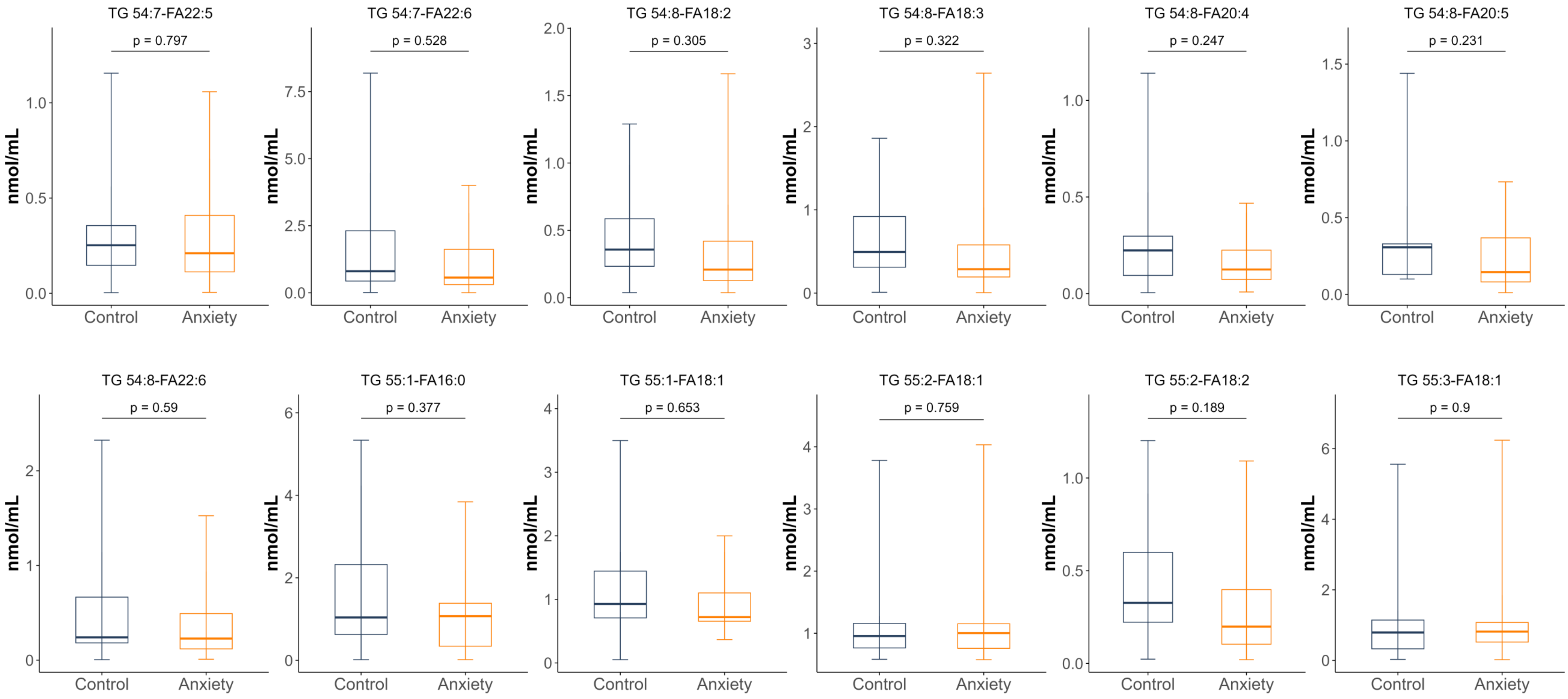

Supplementary Figure 44. Plasma triacylglycerol species profile. Results are presented as box-and-whisker plots showing the median, interquartile range, and 5th–95th percentiles. Differences between group were assessed using the Mann–Whitney U test. Control (n = 17), Anxiety (n = 17).

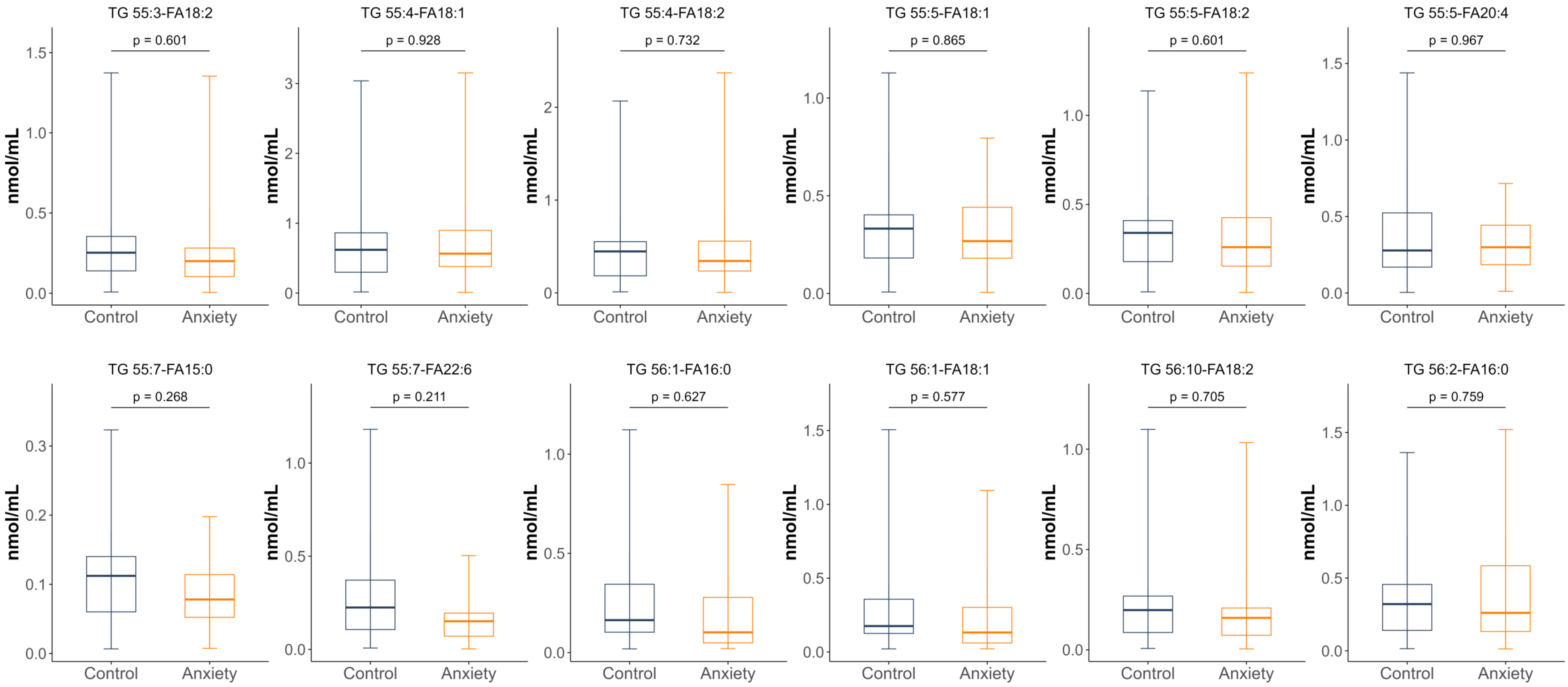

Supplementary Figure 45. Plasma triacylglycerol species profile. Results are presented as box-and-whisker plots showing the median, interquartile range, and 5th–95th percentiles. Differences between group were assessed using the Mann–Whitney U test. Control (n = 17), Anxiety (n = 17).

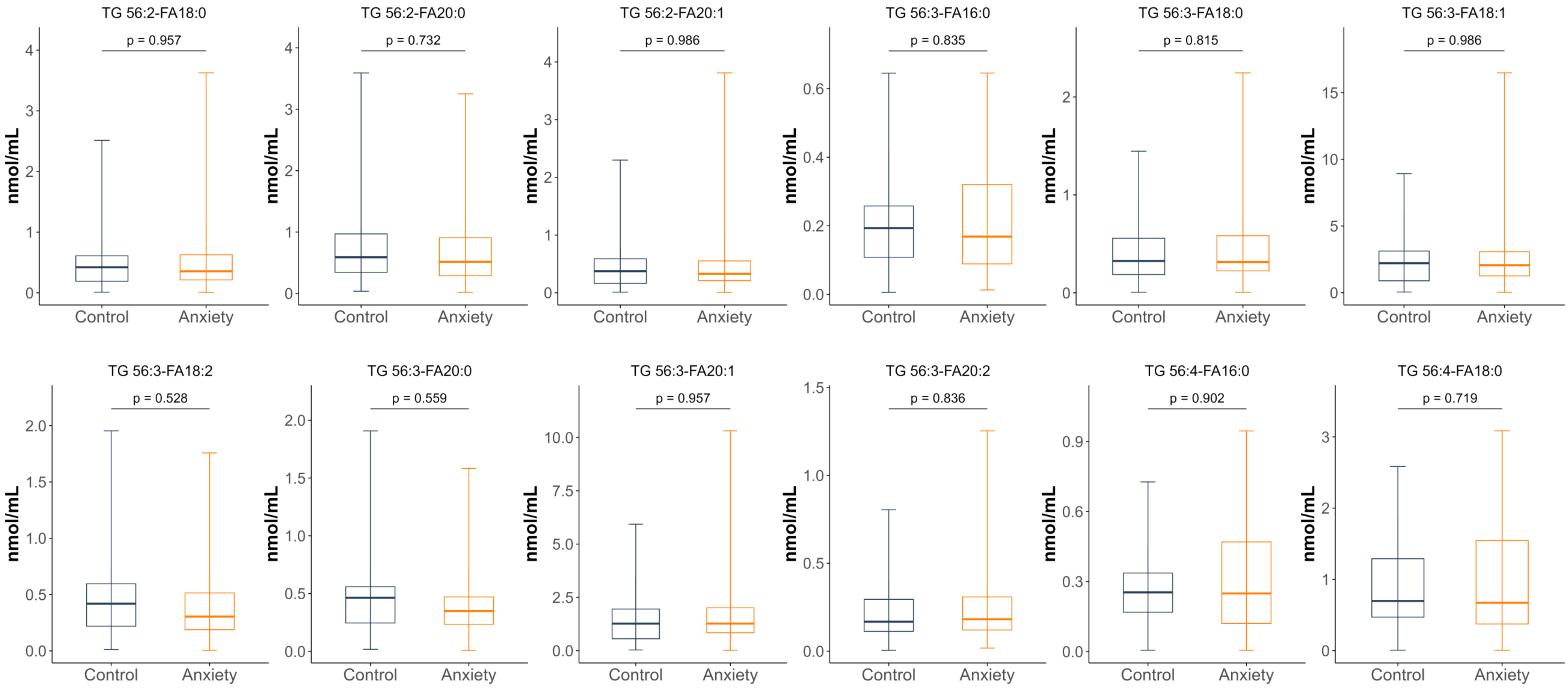

Supplementary Figure 46. Plasma triacylglycerol species profile. Results are presented as box-and-whisker plots showing the median, interquartile range, and 5th–95th percentiles. Differences between group were assessed using the Mann–Whitney U test. Control (n = 17), Anxiety (n = 17).

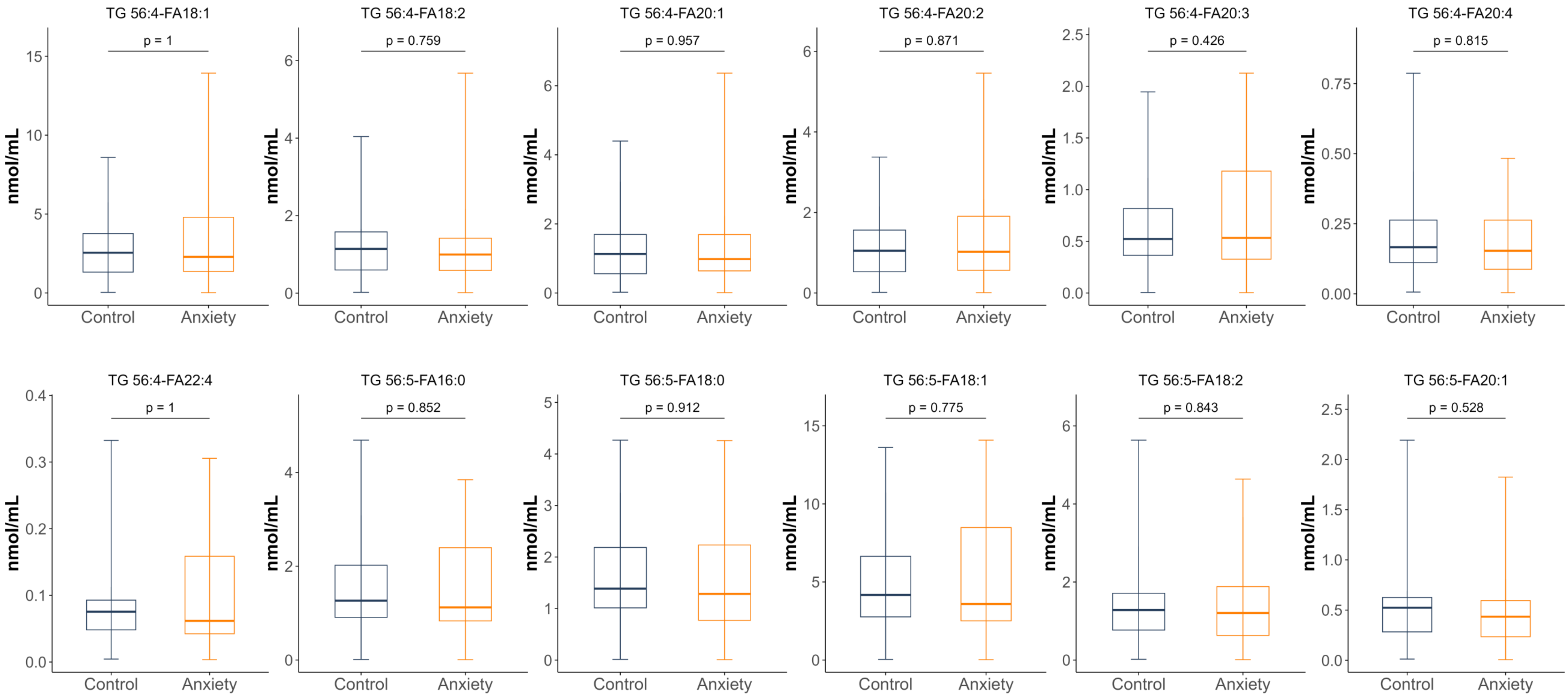

Supplementary Figure 47. Plasma triacylglycerol species profile. Results are presented as box-and-whisker plots showing the median, interquartile range, and 5th–95th percentiles. Differences between group were assessed using the Mann–Whitney U test. Control (n = 17), Anxiety (n = 17).

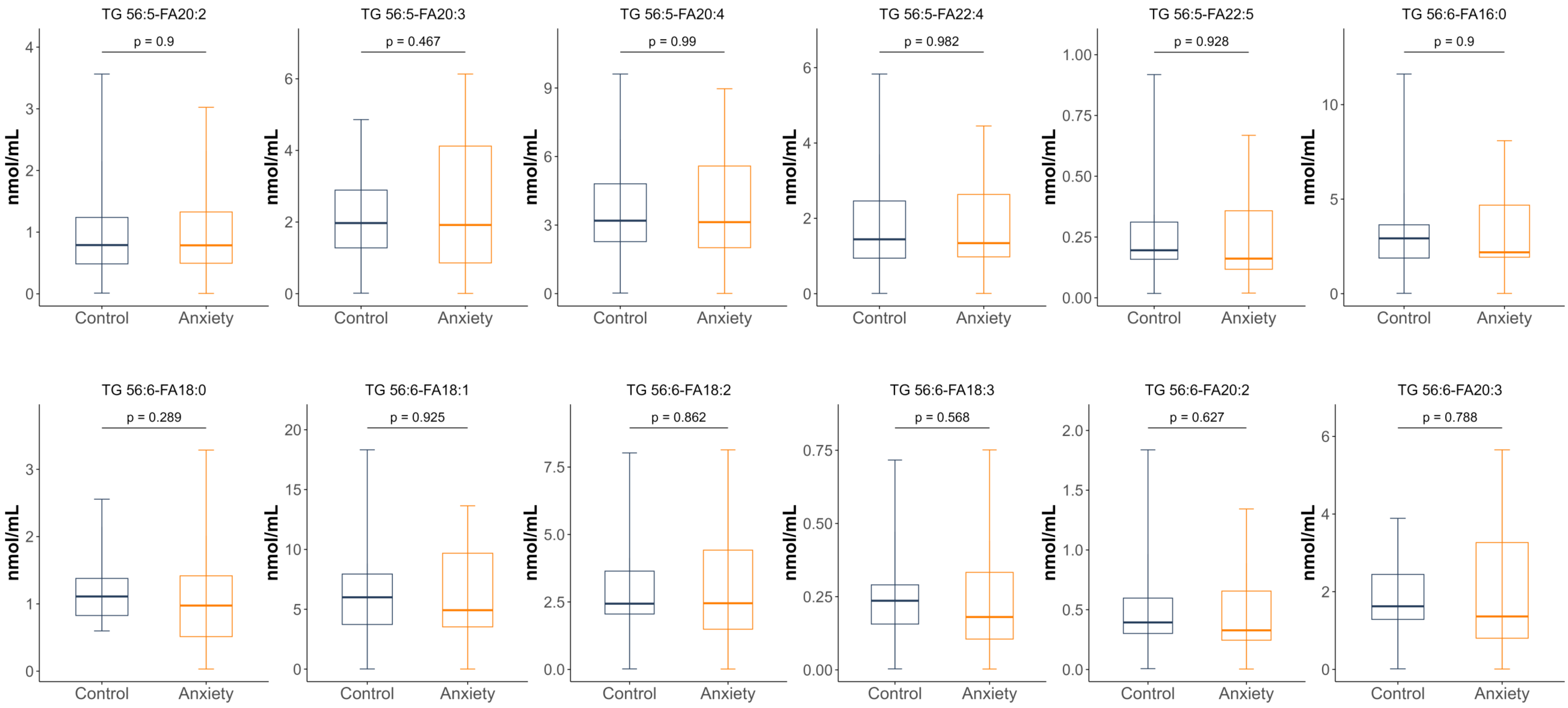

Supplementary Figure 48. Plasma triacylglycerol species profile. Results are presented as box-and-whisker plots showing the median, interquartile range, and 5th–95th percentiles. Differences between group were assessed using the Mann–Whitney U test. Control (n = 17), Anxiety (n = 17).

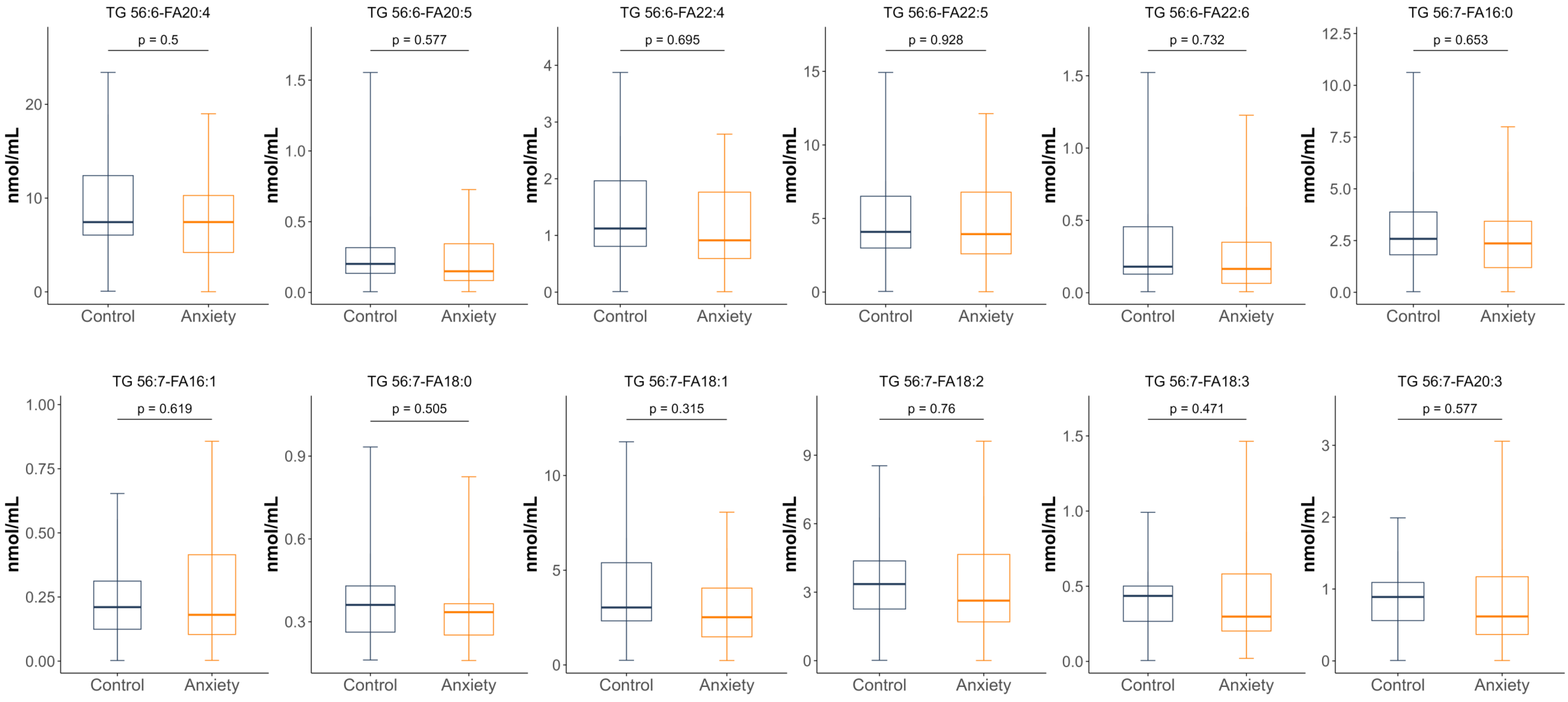

Supplementary Figure 49. Plasma triacylglycerol species profile. Results are presented as box-and-whisker plots showing the median, interquartile range, and 5th–95th percentiles. Differences between group were assessed using the Mann–Whitney U test. Control (n = 17), Anxiety (n = 17).

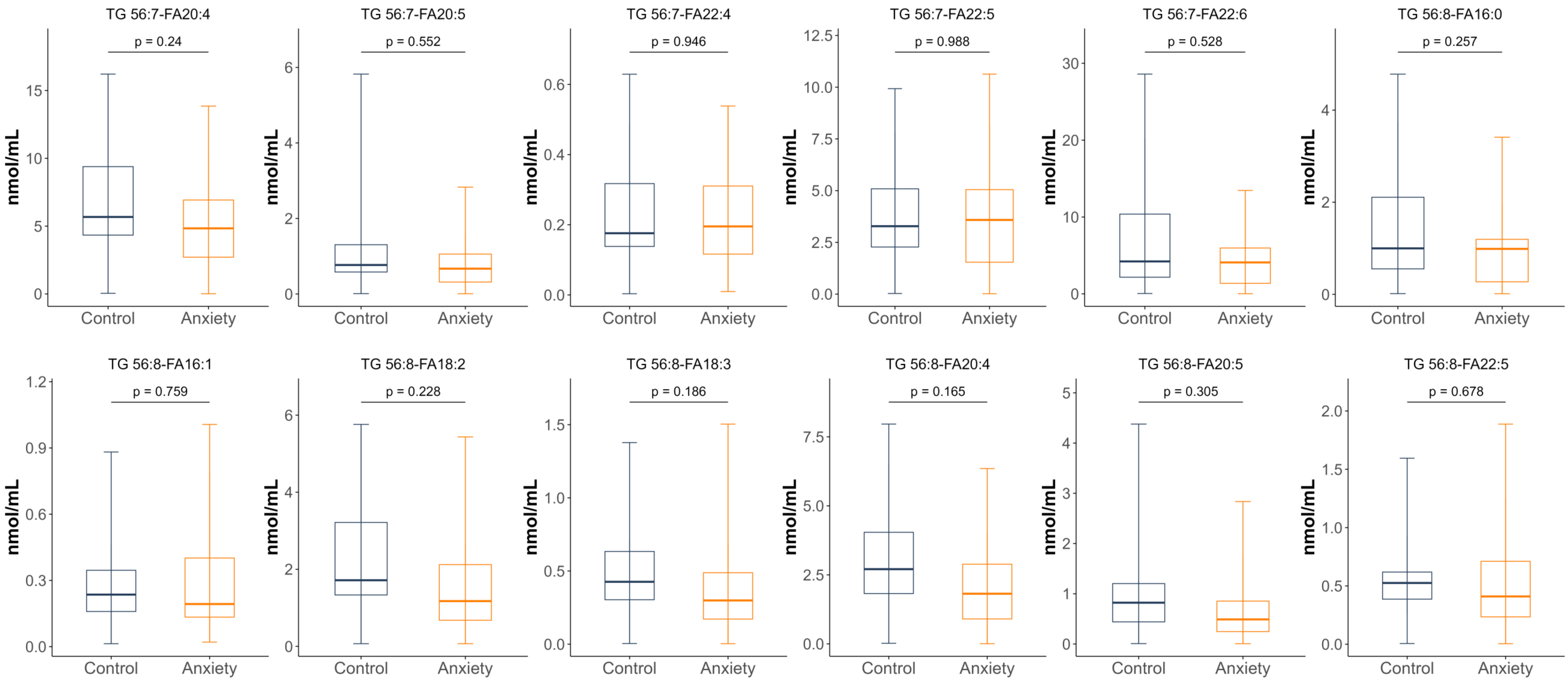

Supplementary Figure 50. Plasma triacylglycerol species profile. Results are presented as box-and-whisker plots showing the median, interquartile range, and 5th–95th percentiles. Differences between group were assessed using the Mann–Whitney U test. Control (n = 17), Anxiety (n = 17).

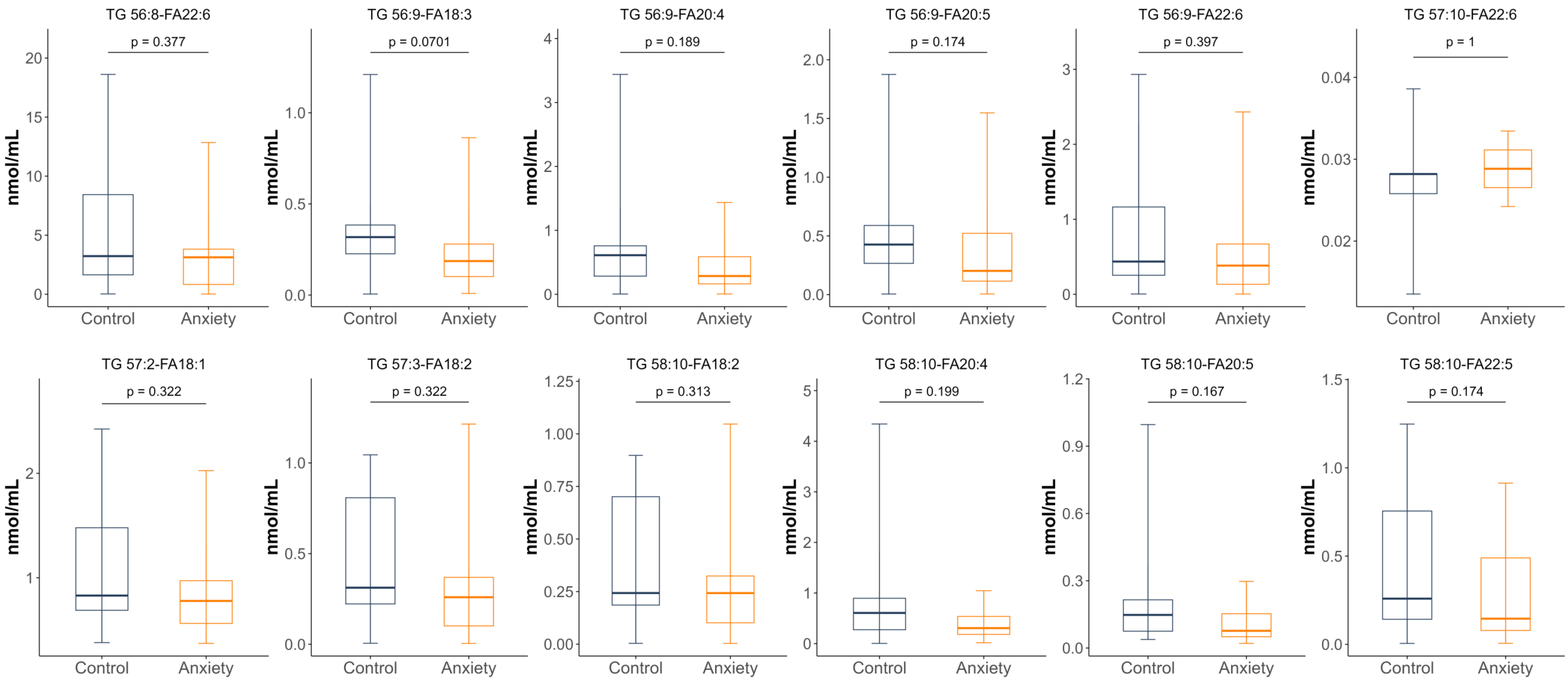

Supplementary Figure 51. Plasma triacylglycerol species profile. Results are presented as box-and-whisker plots showing the median, interquartile range, and 5th–95th percentiles. Differences between group were assessed using the Mann–Whitney U test. Control (n = 17), Anxiety (n = 17).

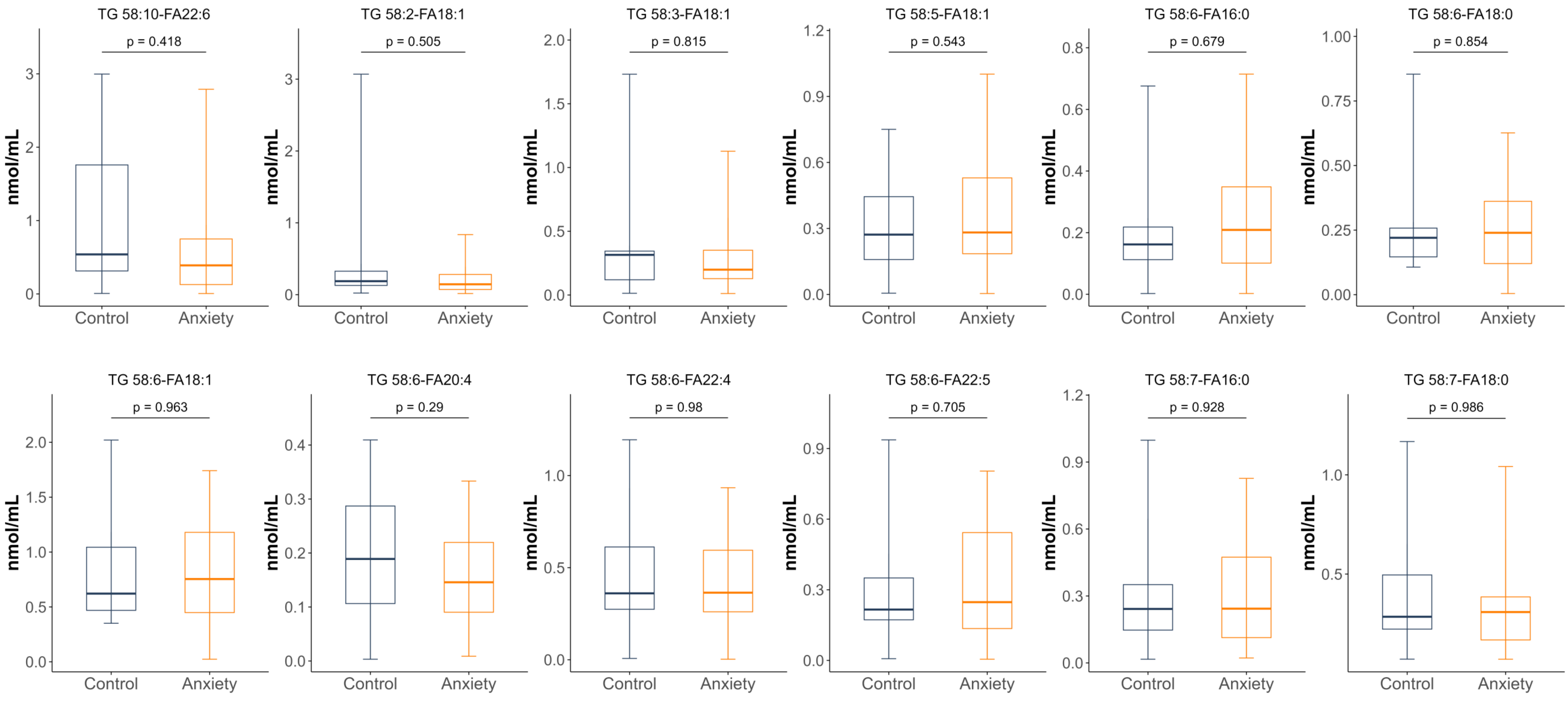

Supplementary Figure 52. Plasma triacylglycerol species profile. Results are presented as box-and-whisker plots showing the median, interquartile range, and 5th–95th percentiles. Differences between group were assessed using the Mann–Whitney U test. Control (n = 17), Anxiety (n = 17).

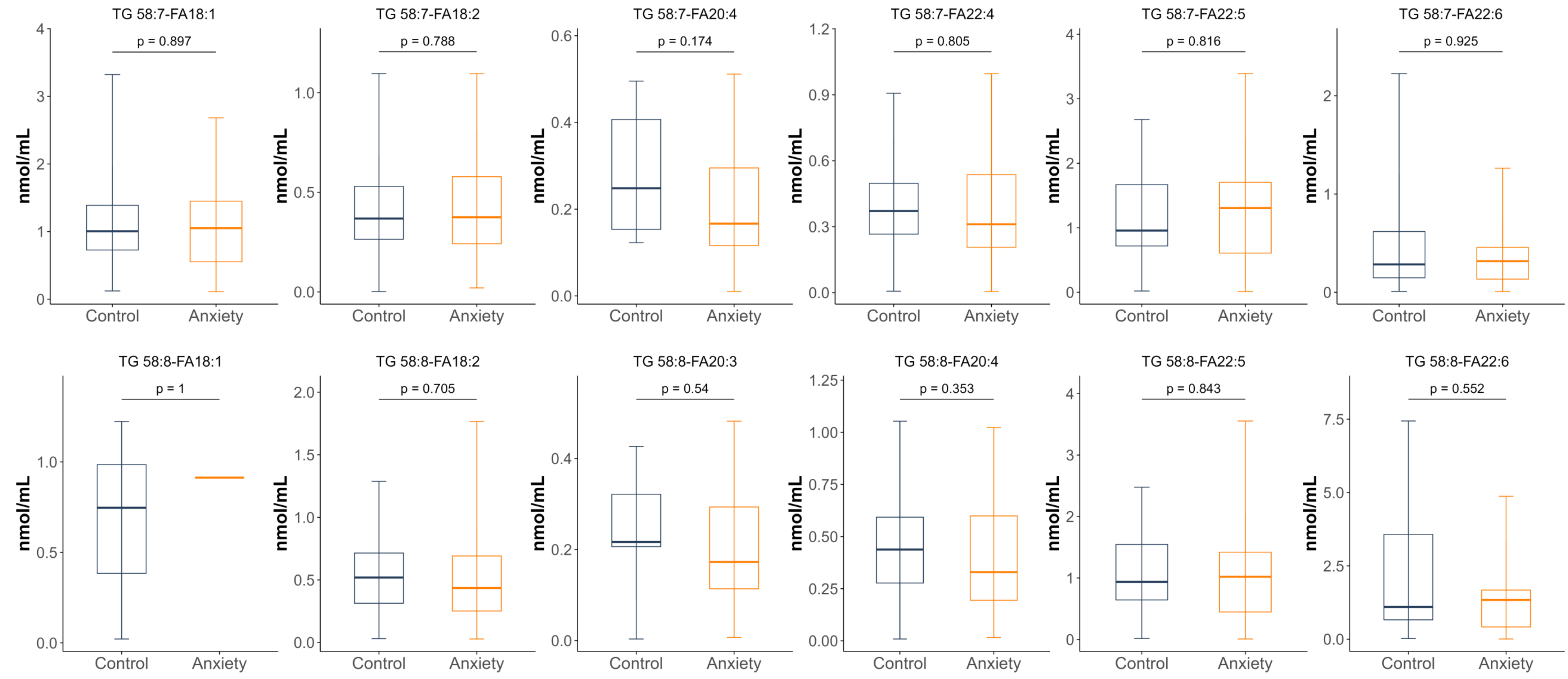

Supplementary Figure 53. Plasma triacylglycerol species profile. Results are presented as box-and-whisker plots showing the median, interquartile range, and 5th–95th percentiles. Differences between group were assessed using the Mann–Whitney U test. Control (n = 17), Anxiety (n = 17).

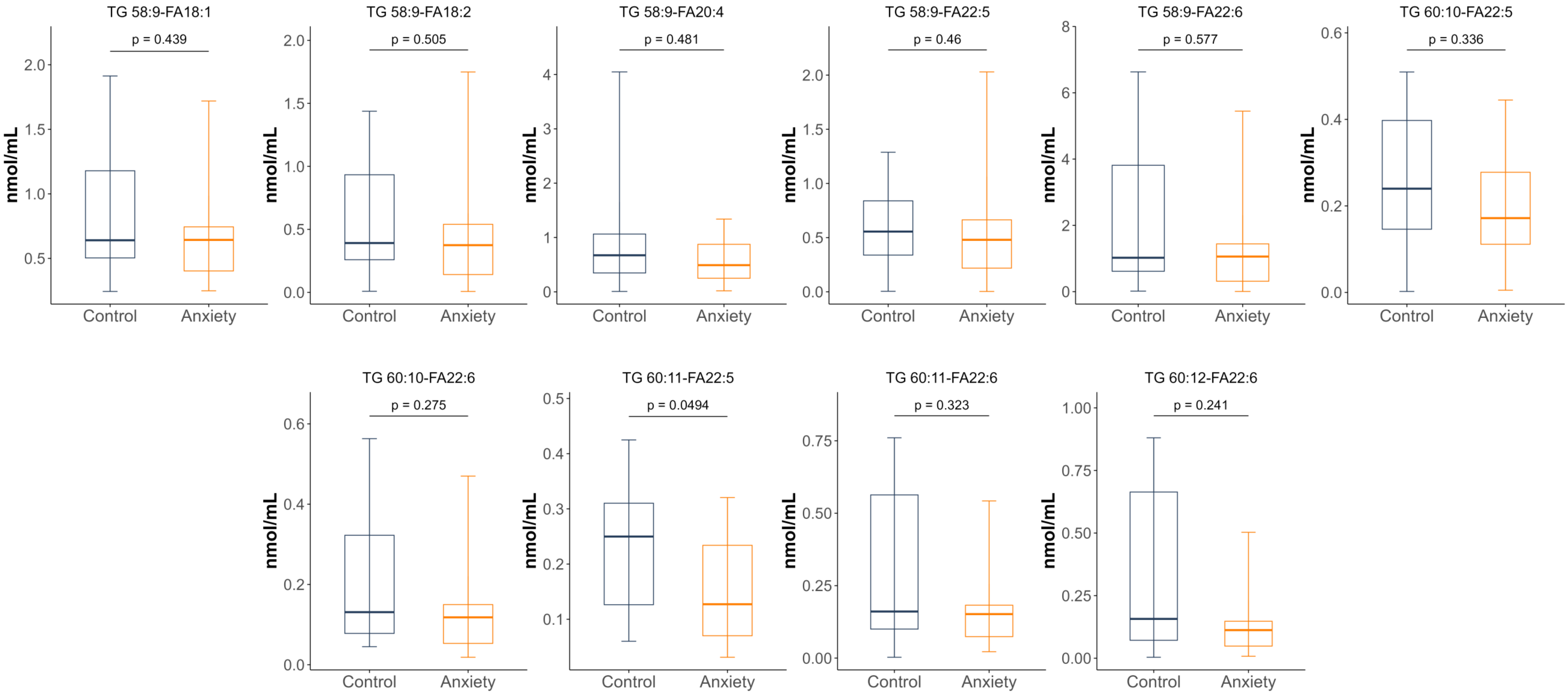

Supplementary Figure 54. Plasma triacylglycerol species profile. Results are presented as box-and-whisker plots showing the median, interquartile range, and 5th–95th percentiles. Differences between group were assessed using the Mann–Whitney U test. Control (n = 17), Anxiety (n = 17).
