# Supplementary material for: Anxiety is associated with systemic pro-inflammatory profile and plasma lipid changes in Mexican young adults
Source: Front Cell Neurosci. 2026 Feb 27;20:1777048. doi: 10.3389/fncel.2026.1777048 (PMC12982057; doi:10.3389/fncel.2026.1777048)
Supplement: Supplementary file 2 [file Data_Sheet_2.pdf]

Lipid	p_value	Significance	p_BH	Significance_BH
DHCer 12:0	0.871228	ns	1	ns
DHCer 14:0	0.802137	ns	1	ns
DHCer 14:1	0.928244	ns	1	ns
DHCer 15:0	0.16549	ns	1	ns
DHCer 16:0	0.576613	ns	1	ns
DHCer 16:1	0.781213	ns	1	ns
DHCer 17:0	0.980643	ns	1	ns
DHCer 18:0	0.98563	ns	1	ns
DHCer 18:1	0.956909	ns	1	ns
DHCer 18:2	0.576613	ns	1	ns
DHCer 18:3	0.966999	ns	1	ns
DHCer 18:4	0.948241	ns	1	ns
DHCer 20:0	0.70526	ns	1	ns
DHCer 20:1	0.397266	ns	1	ns
DHCer 20:2	0.787034	ns	1	ns
DHCer 20:3	0.956609	ns	1	ns
DHCer 20:4	0.418701	ns	1	ns
DHCer 20:5	0.397266	ns	1	ns
DHCer 22:0	0.101214	ns	1	ns
DHCer 22:1	0.287942	ns	1	ns
DHCer 22:2	0.273464	ns	1	ns
DHCer 22:4	0.601449	ns	1	ns
DHCer 22:5	0.70526	ns	1	ns
DHCer 22:6	0.445121	ns	1	ns
DHCer 24:0	0.167853	ns	1	ns
DHCer 24:1	0.505152	ns	1	ns
Cer 16:0	0.652513	ns	1	ns
Cer 18:0	0.583152	ns	1	ns
Cer 18:1	0.674334	ns	1	ns
Cer 20:0	0.884127	ns	1	ns
Cer 20:1	0.77283	ns	1	ns
Cer 22:0	0.505152	ns	1	ns
Cer 22:1	0.848899	ns	1	ns
Cer 24:0	0.563099	ns	1	ns
Cer 24:1	0.970267	ns	1	ns
Cer 26:0	0.75923	ns	1	ns
Cer 26:1	0.117052	ns	1	ns
DG 12:0 16:0	0.737765	ns	1	ns
DG 12:0 18:0	0.166405	ns	1	ns
DG 12:0 18:1	0.956909	ns	1	ns
DG 12:0 18:2	0.626757	ns	1	ns
DG 12:0 20:0	0.760804	ns	1	ns
DG 14:0 14:0	0.435652	ns	1	ns
DG 14:0 16:0	0.626757	ns	1	ns
DG 14:0 16:1	0.975263	ns	1	ns
DG 14:0 18:1	0.732194	ns	1	ns
DG 14:0 18:2	0.814874	ns	1	ns
DG 14:0 18:3	0.433989	ns	1	ns
DG 14:0 20:0	0.657255	ns	1	ns

DG 14:0 20:4	0.056112	ns	1	ns
DG 14:0 22:6	0.77283	ns	1	ns
DG 14:1 16:0	0.482413	ns	1	ns
DG 14:1 18:1	0.732194	ns	1	ns
DG 14:1 20:0	0.814874	ns	1	ns
DG 15:0 18:1	0.899671	ns	1	ns
DG 15:0 18:2	0.236632	ns	1	ns
DG 15:0 20:0	0.717187	ns	1	ns
DG 16:0 16:0	0.32188	ns	1	ns
DG 16:0 16:1	0.871228	ns	1	ns
DG 16:0 18:0	0.787034	ns	1	ns
DG 16:0 18:1	0.814874	ns	1	ns
DG 16:0 18:2	0.98563	ns	1	ns
DG 16:0 18:3	0.98563	ns	1	ns
DG 16:0 20:0	0.087074	ns	1	ns
DG 16:0 20:3	0.868226	ns	1	ns
DG 16:0 20:4	0.803463	ns	1	ns
DG 16:0 20:5	0.134264	ns	1	ns
DG 16:0 22:5	0.970065	ns	1	ns
DG 16:0 22:6	0.326109	ns	1	ns
DG 16:1 16:1	0.806473	ns	1	ns
DG 16:1 18:0	0.49254	ns	1	ns
DG 16:1 18:1	0.842951	ns	1	ns
DG 16:1 18:2	1	ns	1	ns
DG 16:1 18:3	0.657339	ns	1	ns
DG 16:1 20:0	0.445009	ns	1	ns
DG 16:1 20:2	0.514259	ns	1	ns
DG 16:1 20:4	0.289371	ns	1	ns
DG 16:1 22:6	0.070196	ns	1	ns
DG 17:0 20:0	0.303481	ns	1	ns
DG 18:0 18:1	0.576613	ns	1	ns
DG 18:0 18:2	0.899671	ns	1	ns
DG 18:0 18:3	0.589739	ns	1	ns
DG 18:0 20:0	0.372832	ns	1	ns
DG 18:0 22:6	0.692302	ns	1	ns
DG 18:1 18:1	0.55227	ns	1	ns
DG 18:1 18:2	0.806379	ns	1	ns
DG 18:1 20:0	0.12486	ns	1	ns
DG 18:1 20:1	0.235748	ns	1	ns
DG 18:1 20:2	0.252826	ns	1	ns
DG 18:1 20:3	0.766144	ns	1	ns
DG 18:1 20:4	0.397266	ns	1	ns
DG 18:1 20:5	0.213374	ns	1	ns
DG 18:1 22:4	0.439709	ns	1	ns
DG 18:1 22:5	0.916013	ns	1	ns
DG 18:1 22:6	0.213958	ns	1	ns
DG 18:2 18:3	0.102794	ns	1	ns
DG 18:2 20:0	0.097617	ns	1	ns
DG 18:2 20:3	0.202597	ns	1	ns
DG 18:2 20:4	0.131982	ns	1	ns

DG 18:2 20:5	0.053161	ns	1	ns
DG 18:2 22:4	0.004717	**	0.6500026	ns
DG 18:2 22:5	0.184316	ns	1	ns
DG 18:2 22:6	0.021318	*	1	ns
DG 18:4 20:0	0.382733	ns	1	ns
DG 20:1 20:0	0.994257	ns	1	ns
DG 20:2 20:0	0.177852	ns	1	ns
DG 20:4 20:0	1	ns	1	ns
DG 20:5 20:0	1	ns	1	ns
DG 22:0 20:0	0.06899	ns	1	ns
DG 22:1 20:0	0.290096	ns	1	ns
DG 22:5 20:0	1	ns	1	ns
DG 22:6 20:0	0.460242	ns	1	ns
FA 12:0	0.62467	ns	1	ns
FA 14:0	0.795413	ns	1	ns
FA 14:1	0.317861	ns	1	ns
FA 15:0	0.250284	ns	1	ns
FA 16:0	0.484267	ns	1	ns
FA 16:1	0.418333	ns	1	ns
FA 17:0	0.29919	ns	1	ns
FA 18:0	0.315358	ns	1	ns
FA 18:1	0.917701	ns	1	ns
FA 18:2	0.794133	ns	1	ns
FA 18:3	0.784404	ns	1	ns
FA 18:4	0.890656	ns	1	ns
FA 20:0	0.605398	ns	1	ns
FA 20:1	0.845478	ns	1	ns
FA 20:2	0.952567	ns	1	ns
FA 20:3	0.535269	ns	1	ns
FA 20:4	0.539666	ns	1	ns
FA 20:5	0.917701	ns	1	ns
FA 22:0	0.650234	ns	1	ns
FA 22:1	0.491412	ns	1	ns
FA 22:2	0.228001	ns	1	ns
FA 22:4	0.666909	ns	1	ns
FA 22:5	0.336514	ns	1	ns
FA 22:6	0.530739	ns	1	ns
FA 24:0	0.229518	ns	1	ns
FA 24:1	0.917219	ns	1	ns
HexCer 14:0	1	ns	1	ns
HexCer 16:0	0.639005	ns	1	ns
HexCer 18:0	0.41916	ns	1	ns
HexCer 18:1	0.981017	ns	1	ns
HexCer 20:0	0.613715	ns	1	ns
HexCer 20:1	0.371093	ns	1	ns
HexCer 22:0	0.740572	ns	1	ns
HexCer 22:1	0.737383	ns	1	ns
HexCer 24:0	0.842135	ns	1	ns
HexCer 24:1	0.576567	ns	1	ns
HexCer 26:0	0.674816	ns	1	ns

HexCer 26:1	0.698535	ns	1	ns
LPC 14:0	0.093931	ns	1	ns
LPC 14:1	0.712178	ns	1	ns
LPC 15:0	0.934982	ns	1	ns
LPC 16:0	0.297711	ns	1	ns
LPC 16:1	0.32188	ns	1	ns
LPC 17:0	0.427414	ns	1	ns
LPC 18:0	0.259532	ns	1	ns
LPC 18:1	0.521029	ns	1	ns
LPC 18:2	0.945201	ns	1	ns
LPC 18:3	0.785302	ns	1	ns
LPC 19:0	0.787116	ns	1	ns
LPC 20:0	0.516601	ns	1	ns
LPC 20:1	0.915403	ns	1	ns
LPC 20:2	0.918465	ns	1	ns
LPC 20:3	0.729996	ns	1	ns
LPC 20:4	0.895192	ns	1	ns
LPC 20:5	0.528444	ns	1	ns
LPC 22:0	0.10575	ns	1	ns
LPC 22:1	0.134938	ns	1	ns
LPC 22:2	0.166666	ns	1	ns
LPC 22:4	0.306761	ns	1	ns
LPC 22:5	0.559434	ns	1	ns
LPC 22:6	0.796243	ns	1	ns
LPC 24:0	0.08399	ns	1	ns
LPC 24:1	0.034449	*	1	ns
LPE 14:1	0.07101	ns	1	ns
LPE 16:0	0.364131	ns	1	ns
LPE 16:1	0.867632	ns	1	ns
LPE 18:0	0.434057	ns	1	ns
LPE 18:1	0.744065	ns	1	ns
LPE 18:2	0.976658	ns	1	ns
LPE 18:3	0.761909	ns	1	ns
LPE 20:1	1	ns	1	ns
LPE 20:2	0.406436	ns	1	ns
LPE 20:3	0.913414	ns	1	ns
LPE 20:4	0.479577	ns	1	ns
LPE 20:5	0.0539	ns	1	ns
LPE 22:4	0.182409	ns	1	ns
LPE 22:5	0.333358	ns	1	ns
LPE 22:6	0.068645	ns	1	ns
LPE 24:0	0.846451	ns	1	ns
LacCer 14:0	0.245278	ns	1	ns
LacCer 16:0	0.195778	ns	1	ns
LacCer 20:1	0.77283	ns	1	ns
LacCer 22:0	0.245278	ns	1	ns
LacCer 22:1	0.382733	ns	1	ns
LacCer 24:1	0.189415	ns	1	ns
TG 36:0 FA12:0	0.601449	ns	1	ns
TG 38:0 FA12:0	0.67869	ns	1	ns

TG 40:0 FA12:0	0.576613	ns	1	ns
TG 40:0 FA14:0	0.956909	ns	1	ns
TG 40:0 FA16:0	0.899671	ns	1	ns
TG 42:0 FA12:0	0.601449	ns	1	ns
TG 42:0 FA14:0	0.98563	ns	1	ns
TG 42:0 FA16:0	0.956909	ns	1	ns
TG 42:1 FA12:0	1	ns	1	ns
TG 42:1 FA14:0	0.842951	ns	1	ns
TG 42:1 FA16:0	0.899671	ns	1	ns
TG 42:1 FA16:1	0.814874	ns	1	ns
TG 42:1 FA18:1	0.871228	ns	1	ns
TG 42:2 FA12:0	0.816735	ns	1	ns
TG 42:2 FA18:2	0.956909	ns	1	ns
TG 44:0 FA12:0	0.652513	ns	1	ns
TG 44:0 FA14:0	0.98563	ns	1	ns
TG 44:0 FA16:0	0.787034	ns	1	ns
TG 44:0 FA18:0	0.67869	ns	1	ns
TG 44:1 FA12:0	0.652513	ns	1	ns
TG 44:1 FA14:0	0.652513	ns	1	ns
TG 44:1 FA14:1	0.652513	ns	1	ns
TG 44:1 FA16:0	0.759463	ns	1	ns
TG 44:1 FA16:1	0.626757	ns	1	ns
TG 44:1 FA18:1	0.842951	ns	1	ns
TG 44:2 FA12:0	0.928244	ns	1	ns
TG 44:2 FA14:0	1	ns	1	ns
TG 44:2 FA16:0	0.98563	ns	1	ns
TG 44:2 FA16:1	0.787034	ns	1	ns
TG 44:2 FA18:1	0.98563	ns	1	ns
TG 44:2 FA18:2	0.928244	ns	1	ns
TG 44:3 FA18:2	0.984965	ns	1	ns
TG 45:0 FA14:0	0.814874	ns	1	ns
TG 45:0 FA15:0	0.871228	ns	1	ns
TG 45:0 FA16:0	0.98563	ns	1	ns
TG 45:1 FA15:0	0.956909	ns	1	ns
TG 45:1 FA16:0	0.842951	ns	1	ns
TG 45:1 FA18:1	0.928244	ns	1	ns
TG 46:0 FA12:0	0.32188	ns	1	ns
TG 46:0 FA14:0	0.759463	ns	1	ns
TG 46:0 FA16:0	0.759463	ns	1	ns
TG 46:0 FA18:0	0.759463	ns	1	ns
TG 46:1 FA12:0	0.67869	ns	1	ns
TG 46:1 FA14:0	0.67869	ns	1	ns
TG 46:1 FA14:1	0.871228	ns	1	ns
TG 46:1 FA16:0	0.787034	ns	1	ns
TG 46:1 FA16:1	0.787034	ns	1	ns
TG 46:1 FA18:0	0.871228	ns	1	ns
TG 46:1 FA18:1	0.732194	ns	1	ns
TG 46:2 FA12:0	0.956909	ns	1	ns
TG 46:2 FA14:0	0.928244	ns	1	ns
TG 46:2 FA14:1	0.652513	ns	1	ns

TG 46:2 FA16:0	0.899671	ns	1	ns
TG 46:2 FA16:1	0.576613	ns	1	ns
TG 46:2 FA18:1	0.759463	ns	1	ns
TG 46:2 FA18:2	1	ns	1	ns
TG 46:3 FA12:0	0.480731	ns	1	ns
TG 46:3 FA14:0	0.762574	ns	1	ns
TG 46:3 FA14:1	0.98563	ns	1	ns
TG 46:3 FA16:0	0.814874	ns	1	ns
TG 46:3 FA16:1	0.652513	ns	1	ns
TG 46:3 FA18:1	0.871228	ns	1	ns
TG 46:3 FA18:2	0.98563	ns	1	ns
TG 46:3 FA18:3	0.789551	ns	1	ns
TG 46:4 FA18:2	0.895055	ns	1	ns
TG 47:0 FA14:0	0.67869	ns	1	ns
TG 47:0 FA15:0	0.759463	ns	1	ns
TG 47:0 FA16:0	0.732194	ns	1	ns
TG 47:0 FA17:0	0.842951	ns	1	ns
TG 47:1 FA14:0	0.67869	ns	1	ns
TG 47:1 FA15:0	0.626757	ns	1	ns
TG 47:1 FA16:0	0.732194	ns	1	ns
TG 47:1 FA16:1	0.732194	ns	1	ns
TG 47:1 FA17:0	0.70526	ns	1	ns
TG 47:1 FA18:1	0.842951	ns	1	ns
TG 47:2 FA14:0	0.928244	ns	1	ns
TG 47:2 FA15:0	0.928244	ns	1	ns
TG 47:2 FA16:1	0.842951	ns	1	ns
TG 47:2 FA18:1	0.842951	ns	1	ns
TG 47:2 FA18:2	0.98563	ns	1	ns
TG 48:0 FA14:0	0.732194	ns	1	ns
TG 48:0 FA16:0	0.70526	ns	1	ns
TG 48:0 FA18:0	0.787034	ns	1	ns
TG 48:1 FA12:0	0.732194	ns	1	ns
TG 48:1 FA14:0	0.787034	ns	1	ns
TG 48:1 FA14:1	0.956909	ns	1	ns
TG 48:1 FA16:0	0.842951	ns	1	ns
TG 48:1 FA16:1	0.67869	ns	1	ns
TG 48:1 FA18:0	0.652513	ns	1	ns
TG 48:1 FA18:1	0.787034	ns	1	ns
TG 48:2 FA12:0	0.67869	ns	1	ns
TG 48:2 FA14:0	0.759463	ns	1	ns
TG 48:2 FA14:1	0.70526	ns	1	ns
TG 48:2 FA16:0	0.814874	ns	1	ns
TG 48:2 FA16:1	0.732194	ns	1	ns
TG 48:2 FA18:0	0.842951	ns	1	ns
TG 48:2 FA18:1	0.67869	ns	1	ns
TG 48:2 FA18:2	1	ns	1	ns
TG 48:3 FA12:0	0.842951	ns	1	ns
TG 48:3 FA14:0	0.899671	ns	1	ns
TG 48:3 FA14:1	0.956909	ns	1	ns
TG 48:3 FA16:0	0.771551	ns	1	ns

TG 48:3 FA16:1	0.787034	ns	1	ns
TG 48:3 FA18:1	0.787034	ns	1	ns
TG 48:3 FA18:2	0.871228	ns	1	ns
TG 48:3 FA18:3	0.98563	ns	1	ns
TG 48:4 FA12:0	0.777432	ns	1	ns
TG 48:4 FA14:0	0.81915	ns	1	ns
TG 48:4 FA14:1	0.814874	ns	1	ns
TG 48:4 FA16:0	0.787034	ns	1	ns
TG 48:4 FA16:1	0.956909	ns	1	ns
TG 48:4 FA18:1	0.806473	ns	1	ns
TG 48:4 FA18:2	0.928244	ns	1	ns
TG 48:4 FA18:3	0.806473	ns	1	ns
TG 48:4 FA20:4	0.83986	ns	1	ns
TG 48:5 FA18:2	0.803463	ns	1	ns
TG 48:5 FA18:3	0.868226	ns	1	ns
TG 49:0 FA15:0	0.55227	ns	1	ns
TG 49:0 FA16:0	0.460242	ns	1	ns
TG 49:0 FA17:0	0.601449	ns	1	ns
TG 49:0 FA18:0	0.55227	ns	1	ns
TG 49:1 FA14:0	0.732194	ns	1	ns
TG 49:1 FA15:0	0.67869	ns	1	ns
TG 49:1 FA16:0	0.601449	ns	1	ns
TG 49:1 FA16:1	0.652513	ns	1	ns
TG 49:1 FA17:0	0.601449	ns	1	ns
TG 49:1 FA18:1	0.576613	ns	1	ns
TG 49:2 FA14:0	0.67869	ns	1	ns
TG 49:2 FA15:0	0.928244	ns	1	ns
TG 49:2 FA16:0	0.871228	ns	1	ns
TG 49:2 FA16:1	0.55227	ns	1	ns
TG 49:2 FA17:0	0.67869	ns	1	ns
TG 49:2 FA18:1	0.576613	ns	1	ns
TG 49:2 FA18:2	0.956909	ns	1	ns
TG 49:3 FA15:0	0.820753	ns	1	ns
TG 49:3 FA16:0	0.759463	ns	1	ns
TG 49:3 FA16:1	0.787034	ns	1	ns
TG 49:3 FA18:2	0.956909	ns	1	ns
TG 49:3 FA18:3	0.835705	ns	1	ns
TG 50:0 FA14:0	0.55227	ns	1	ns
TG 50:0 FA16:0	0.70526	ns	1	ns
TG 50:0 FA18:0	0.652513	ns	1	ns
TG 50:1 FA14:0	0.732194	ns	1	ns
TG 50:1 FA16:0	0.787034	ns	1	ns
TG 50:1 FA16:1	0.55227	ns	1	ns
TG 50:1 FA18:0	0.67869	ns	1	ns
TG 50:1 FA18:1	0.899671	ns	1	ns
TG 50:1 FA20:1	0.759463	ns	1	ns
TG 50:2 FA14:0	0.528444	ns	1	ns
TG 50:2 FA14:1	0.55227	ns	1	ns
TG 50:2 FA16:0	0.899671	ns	1	ns
TG 50:2 FA16:1	0.67869	ns	1	ns

TG 50:2 FA18:0	0.759463	ns	1	ns
TG 50:2 FA18:1	0.576613	ns	1	ns
TG 50:2 FA18:2	0.98563	ns	1	ns
TG 50:2 FA20:2	0.899671	ns	1	ns
TG 50:3 FA14:0	0.899671	ns	1	ns
TG 50:3 FA14:1	0.67869	ns	1	ns
TG 50:3 FA16:0	0.956909	ns	1	ns
TG 50:3 FA16:1	0.732194	ns	1	ns
TG 50:3 FA18:0	0.889964	ns	1	ns
TG 50:3 FA18:1	0.759463	ns	1	ns
TG 50:3 FA18:2	0.842951	ns	1	ns
TG 50:3 FA18:3	0.928244	ns	1	ns
TG 50:3 FA20:3	0.705707	ns	1	ns
TG 50:4 FA14:0	0.871228	ns	1	ns
TG 50:4 FA14:1	0.984965	ns	1	ns
TG 50:4 FA16:0	1	ns	1	ns
TG 50:4 FA16:1	0.67869	ns	1	ns
TG 50:4 FA18:1	0.928244	ns	1	ns
TG 50:4 FA18:2	0.928244	ns	1	ns
TG 50:4 FA18:3	0.98563	ns	1	ns
TG 50:4 FA20:3	1	ns	1	ns
TG 50:4 FA20:4	0.803463	ns	1	ns
TG 50:5 FA14:0	0.626757	ns	1	ns
TG 50:5 FA16:1	0.928244	ns	1	ns
TG 50:5 FA18:1	0.601449	ns	1	ns
TG 50:5 FA18:2	0.576613	ns	1	ns
TG 50:5 FA18:3	0.576613	ns	1	ns
TG 50:5 FA20:4	0.810299	ns	1	ns
TG 50:5 FA20:5	0.534029	ns	1	ns
TG 50:6 FA20:4	0.7652	ns	1	ns
TG 51:0 FA16:0	0.928244	ns	1	ns
TG 51:0 FA17:0	0.304597	ns	1	ns
TG 51:0 FA18:0	0.505152	ns	1	ns
TG 51:1 FA15:0	0.482413	ns	1	ns
TG 51:1 FA16:0	0.482413	ns	1	ns
TG 51:1 FA17:0	0.576613	ns	1	ns
TG 51:1 FA18:0	0.417657	ns	1	ns
TG 51:1 FA18:1	0.417657	ns	1	ns
TG 51:2 FA15:0	0.67869	ns	1	ns
TG 51:2 FA16:0	0.576613	ns	1	ns
TG 51:2 FA16:1	0.358326	ns	1	ns
TG 51:2 FA17:0	0.871228	ns	1	ns
TG 51:2 FA18:1	0.576613	ns	1	ns
TG 51:2 FA18:2	0.842951	ns	1	ns
TG 51:3 FA15:0	0.899671	ns	1	ns
TG 51:3 FA16:1	0.576613	ns	1	ns
TG 51:3 FA17:0	0.814874	ns	1	ns
TG 51:3 FA18:2	0.98563	ns	1	ns
TG 51:3 FA18:3	0.858819	ns	1	ns
TG 51:4 FA15:0	0.55227	ns	1	ns

TG 51:4 FA16:1	0.70526	ns	1	ns
TG 51:4 FA18:2	0.652513	ns	1	ns
TG 51:4 FA18:3	0.933886	ns	1	ns
TG 51:4 FA20:4	0.585379	ns	1	ns
TG 51:5 FA18:2	0.319506	ns	1	ns
TG 51:5 FA18:3	0.419432	ns	1	ns
TG 52:0 FA16:0	0.576613	ns	1	ns
TG 52:0 FA18:0	0.759463	ns	1	ns
TG 52:0 FA20:0	0.928244	ns	1	ns
TG 52:1 FA16:0	0.626757	ns	1	ns
TG 52:1 FA16:1	0.67869	ns	1	ns
TG 52:1 FA18:0	0.732194	ns	1	ns
TG 52:1 FA18:1	0.626757	ns	1	ns
TG 52:1 FA20:0	0.928244	ns	1	ns
TG 52:1 FA20:1	0.899671	ns	1	ns
TG 52:2 FA14:0	1	ns	1	ns
TG 52:2 FA16:0	0.70526	ns	1	ns
TG 52:2 FA16:1	0.505152	ns	1	ns
TG 52:2 FA18:0	0.67869	ns	1	ns
TG 52:2 FA18:1	0.732194	ns	1	ns
TG 52:2 FA18:2	0.871228	ns	1	ns
TG 52:2 FA20:0	0.899671	ns	1	ns
TG 52:2 FA20:1	0.814874	ns	1	ns
TG 52:2 FA20:2	0.899671	ns	1	ns
TG 52:3 FA14:0	0.98563	ns	1	ns
TG 52:3 FA16:0	0.871228	ns	1	ns
TG 52:3 FA16:1	0.55227	ns	1	ns
TG 52:3 FA18:0	0.814874	ns	1	ns
TG 52:3 FA18:1	0.899671	ns	1	ns
TG 52:3 FA18:2	0.842951	ns	1	ns
TG 52:3 FA18:3	0.956909	ns	1	ns
TG 52:3 FA20:0	0.842951	ns	1	ns
TG 52:3 FA20:1	0.814874	ns	1	ns
TG 52:3 FA20:2	0.759463	ns	1	ns
TG 52:3 FA20:3	0.98563	ns	1	ns
TG 52:3 FA22:1	0.984231	ns	1	ns
TG 52:4 FA14:0	0.72031	ns	1	ns
TG 52:4 FA16:0	0.70526	ns	1	ns
TG 52:4 FA16:1	0.759463	ns	1	ns
TG 52:4 FA18:0	0.72031	ns	1	ns
TG 52:4 FA18:1	0.98563	ns	1	ns
TG 52:4 FA18:2	0.732194	ns	1	ns
TG 52:4 FA18:3	0.871228	ns	1	ns
TG 52:4 FA20:0	0.777432	ns	1	ns
TG 52:4 FA20:2	0.954917	ns	1	ns
TG 52:4 FA20:3	0.842951	ns	1	ns
TG 52:4 FA20:4	0.871228	ns	1	ns
TG 52:4 FA22:1	0.743421	ns	1	ns
TG 52:4 FA22:4	0.564909	ns	1	ns
TG 52:5 FA14:0	0.933886	ns	1	ns

TG 52:5 FA16:0	0.505152	ns	1	ns
TG 52:5 FA16:1	0.956909	ns	1	ns
TG 52:5 FA18:1	0.98563	ns	1	ns
TG 52:5 FA18:2	0.732194	ns	1	ns
TG 52:5 FA18:3	0.576613	ns	1	ns
TG 52:5 FA20:3	0.806473	ns	1	ns
TG 52:5 FA20:4	0.70526	ns	1	ns
TG 52:5 FA20:5	0.652513	ns	1	ns
TG 52:5 FA22:5	0.814874	ns	1	ns
TG 52:6 FA14:0	0.71066	ns	1	ns
TG 52:6 FA16:1	0.759463	ns	1	ns
TG 52:6 FA18:1	0.576613	ns	1	ns
TG 52:6 FA18:2	0.528444	ns	1	ns
TG 52:6 FA18:3	0.460242	ns	1	ns
TG 52:6 FA20:4	0.46461	ns	1	ns
TG 52:6 FA20:5	0.732194	ns	1	ns
TG 52:6 FA22:6	0.450096	ns	1	ns
TG 52:7 FA16:0	0.33979	ns	1	ns
TG 52:7 FA18:1	0.2305	ns	1	ns
TG 52:7 FA20:5	0.836194	ns	1	ns
TG 52:7 FA22:6	0.708923	ns	1	ns
TG 52:8 FA16:1	0.460242	ns	1	ns
TG 52:8 FA18:2	0.393153	ns	1	ns
TG 53:0 FA16:0	0.482413	ns	1	ns
TG 53:1 FA16:0	0.787034	ns	1	ns
TG 53:1 FA17:0	0.460242	ns	1	ns
TG 53:1 FA18:0	0.460242	ns	1	ns
TG 53:1 FA18:1	0.528444	ns	1	ns
TG 53:2 FA16:0	0.842951	ns	1	ns
TG 53:2 FA17:0	0.652513	ns	1	ns
TG 53:2 FA18:1	0.528444	ns	1	ns
TG 53:2 FA18:2	0.871228	ns	1	ns
TG 53:3 FA16:0	0.814874	ns	1	ns
TG 53:3 FA17:0	0.842951	ns	1	ns
TG 53:3 FA18:2	0.928244	ns	1	ns
TG 53:4 FA16:0	0.759463	ns	1	ns
TG 53:4 FA17:0	0.528444	ns	1	ns
TG 53:4 FA18:2	0.812555	ns	1	ns
TG 53:4 FA18:3	0.566534	ns	1	ns
TG 53:4 FA20:4	0.803463	ns	1	ns
TG 53:5 FA20:4	0.868226	ns	1	ns
TG 53:6 FA20:4	0.421348	ns	1	ns
TG 54:0 FA16:0	0.814874	ns	1	ns
TG 54:0 FA18:0	0.871228	ns	1	ns
TG 54:1 FA16:0	0.899671	ns	1	ns
TG 54:1 FA18:0	0.601449	ns	1	ns
TG 54:1 FA18:1	0.626757	ns	1	ns
TG 54:1 FA20:0	0.871228	ns	1	ns
TG 54:1 FA20:1	0.482413	ns	1	ns
TG 54:2 FA16:0	0.98563	ns	1	ns

TG 54:2 FA18:0	0.787034	ns	1	ns
TG 54:2 FA18:1	0.732194	ns	1	ns
TG 54:2 FA18:2	0.928244	ns	1	ns
TG 54:2 FA20:0	0.528444	ns	1	ns
TG 54:2 FA20:1	0.928244	ns	1	ns
TG 54:2 FA20:2	0.55227	ns	1	ns
TG 54:3 FA16:0	1	ns	1	ns
TG 54:3 FA16:1	0.55227	ns	1	ns
TG 54:3 FA18:0	0.98563	ns	1	ns
TG 54:3 FA18:1	0.732194	ns	1	ns
TG 54:3 FA18:2	0.98563	ns	1	ns
TG 54:3 FA18:3	0.928244	ns	1	ns
TG 54:3 FA20:1	0.899671	ns	1	ns
TG 54:3 FA20:2	0.98563	ns	1	ns
TG 54:3 FA20:3	0.98563	ns	1	ns
TG 54:4 FA16:0	0.899671	ns	1	ns
TG 54:4 FA16:1	0.871228	ns	1	ns
TG 54:4 FA18:0	0.899671	ns	1	ns
TG 54:4 FA18:1	0.956909	ns	1	ns
TG 54:4 FA18:2	0.928244	ns	1	ns
TG 54:4 FA18:3	0.899671	ns	1	ns
TG 54:4 FA20:1	0.895055	ns	1	ns
TG 54:4 FA20:2	0.956909	ns	1	ns
TG 54:4 FA20:3	0.623882	ns	1	ns
TG 54:4 FA20:4	0.871228	ns	1	ns
TG 54:4 FA22:1	0.417748	ns	1	ns
TG 54:4 FA22:4	0.850239	ns	1	ns
TG 54:5 FA16:0	0.672663	ns	1	ns
TG 54:5 FA16:1	0.899671	ns	1	ns
TG 54:5 FA18:0	0.505152	ns	1	ns
TG 54:5 FA18:1	0.576613	ns	1	ns
TG 54:5 FA18:2	0.67869	ns	1	ns
TG 54:5 FA18:3	0.842951	ns	1	ns
TG 54:5 FA20:2	0.98563	ns	1	ns
TG 54:5 FA20:3	0.842951	ns	1	ns
TG 54:5 FA20:4	0.55227	ns	1	ns
TG 54:5 FA20:5	0.626757	ns	1	ns
TG 54:5 FA22:1	0.55227	ns	1	ns
TG 54:5 FA22:4	0.850239	ns	1	ns
TG 54:5 FA22:5	0.98563	ns	1	ns
TG 54:6 FA16:0	0.32188	ns	1	ns
TG 54:6 FA16:1	0.899658	ns	1	ns
TG 54:6 FA18:1	0.652513	ns	1	ns
TG 54:6 FA18:2	0.528444	ns	1	ns
TG 54:6 FA18:3	0.652513	ns	1	ns
TG 54:6 FA20:3	0.886728	ns	1	ns
TG 54:6 FA20:4	0.257154	ns	1	ns
TG 54:6 FA20:5	0.55227	ns	1	ns
TG 54:6 FA22:5	0.98563	ns	1	ns
TG 54:6 FA22:6	0.417657	ns	1	ns

TG 54:7 FA16:1	0.345135	ns	1	ns
TG 54:7 FA18:2	0.33979	ns	1	ns
TG 54:7 FA18:3	0.358326	ns	1	ns
TG 54:7 FA20:4	0.322538	ns	1	ns
TG 54:7 FA20:5	0.377487	ns	1	ns
TG 54:7 FA22:5	0.797229	ns	1	ns
TG 54:7 FA22:6	0.528444	ns	1	ns
TG 54:8 FA18:2	0.304597	ns	1	ns
TG 54:8 FA18:3	0.32188	ns	1	ns
TG 54:8 FA20:4	0.247454	ns	1	ns
TG 54:8 FA20:5	0.231034	ns	1	ns
TG 54:8 FA22:6	0.589739	ns	1	ns
TG 55:1 FA16:0	0.377487	ns	1	ns
TG 55:1 FA18:1	0.652513	ns	1	ns
TG 55:2 FA18:1	0.759463	ns	1	ns
TG 55:2 FA18:2	0.188577	ns	1	ns
TG 55:3 FA18:1	0.899671	ns	1	ns
TG 55:3 FA18:2	0.601449	ns	1	ns
TG 55:4 FA18:1	0.928244	ns	1	ns
TG 55:4 FA18:2	0.732194	ns	1	ns
TG 55:5 FA18:1	0.865325	ns	1	ns
TG 55:5 FA18:2	0.601449	ns	1	ns
TG 55:5 FA20:4	0.966915	ns	1	ns
TG 55:7 FA15:0	0.268305	ns	1	ns
TG 55:7 FA22:6	0.211165	ns	1	ns
TG 56:1 FA16:0	0.626757	ns	1	ns
TG 56:1 FA18:1	0.576613	ns	1	ns
TG 56:10 FA18:2	0.70526	ns	1	ns
TG 56:2 FA16:0	0.759463	ns	1	ns
TG 56:2 FA18:0	0.956909	ns	1	ns
TG 56:2 FA20:0	0.732194	ns	1	ns
TG 56:2 FA20:1	0.98563	ns	1	ns
TG 56:3 FA16:0	0.835203	ns	1	ns
TG 56:3 FA18:0	0.814874	ns	1	ns
TG 56:3 FA18:1	0.98563	ns	1	ns
TG 56:3 FA18:2	0.528444	ns	1	ns
TG 56:3 FA20:0	0.5591	ns	1	ns
TG 56:3 FA20:1	0.956909	ns	1	ns
TG 56:3 FA20:2	0.835705	ns	1	ns
TG 56:4 FA16:0	0.90233	ns	1	ns
TG 56:4 FA18:0	0.718506	ns	1	ns
TG 56:4 FA18:1	1	ns	1	ns
TG 56:4 FA18:2	0.759463	ns	1	ns
TG 56:4 FA20:1	0.956909	ns	1	ns
TG 56:4 FA20:2	0.871228	ns	1	ns
TG 56:4 FA20:3	0.425889	ns	1	ns
TG 56:4 FA20:4	0.814874	ns	1	ns
TG 56:4 FA22:4	1	ns	1	ns
TG 56:5 FA16:0	0.852279	ns	1	ns
TG 56:5 FA18:0	0.911814	ns	1	ns

TG 56:5 FA18:1	0.77531	ns	1	ns
TG 56:5 FA18:2	0.842951	ns	1	ns
TG 56:5 FA20:1	0.528444	ns	1	ns
TG 56:5 FA20:2	0.899671	ns	1	ns
TG 56:5 FA20:3	0.466647	ns	1	ns
TG 56:5 FA20:4	0.989972	ns	1	ns
TG 56:5 FA22:4	0.981744	ns	1	ns
TG 56:5 FA22:5	0.928244	ns	1	ns
TG 56:6 FA16:0	0.899671	ns	1	ns
TG 56:6 FA18:0	0.289371	ns	1	ns
TG 56:6 FA18:1	0.924933	ns	1	ns
TG 56:6 FA18:2	0.862245	ns	1	ns
TG 56:6 FA18:3	0.567799	ns	1	ns
TG 56:6 FA20:2	0.626757	ns	1	ns
TG 56:6 FA20:3	0.788161	ns	1	ns
TG 56:6 FA20:4	0.499619	ns	1	ns
TG 56:6 FA20:5	0.576613	ns	1	ns
TG 56:6 FA22:4	0.694992	ns	1	ns
TG 56:6 FA22:5	0.928244	ns	1	ns
TG 56:6 FA22:6	0.732194	ns	1	ns
TG 56:7 FA16:0	0.652513	ns	1	ns
TG 56:7 FA16:1	0.618829	ns	1	ns
TG 56:7 FA18:0	0.505152	ns	1	ns
TG 56:7 FA18:1	0.315295	ns	1	ns
TG 56:7 FA18:2	0.759853	ns	1	ns
TG 56:7 FA18:3	0.471452	ns	1	ns
TG 56:7 FA20:3	0.576613	ns	1	ns
TG 56:7 FA20:4	0.240153	ns	1	ns
TG 56:7 FA20:5	0.55227	ns	1	ns
TG 56:7 FA22:4	0.946357	ns	1	ns
TG 56:7 FA22:5	0.987511	ns	1	ns
TG 56:7 FA22:6	0.528444	ns	1	ns
TG 56:8 FA16:0	0.256505	ns	1	ns
TG 56:8 FA16:1	0.759463	ns	1	ns
TG 56:8 FA18:2	0.227535	ns	1	ns
TG 56:8 FA18:3	0.186269	ns	1	ns
TG 56:8 FA20:4	0.16465	ns	1	ns
TG 56:8 FA20:5	0.304597	ns	1	ns
TG 56:8 FA22:5	0.677856	ns	1	ns
TG 56:8 FA22:6	0.377487	ns	1	ns
TG 56:9 FA18:3	0.070109	ns	1	ns
TG 56:9 FA20:4	0.188577	ns	1	ns
TG 56:9 FA20:5	0.174001	ns	1	ns
TG 56:9 FA22:6	0.397266	ns	1	ns
TG 57:10 FA22:6	1	ns	1	ns
TG 57:2 FA18:1	0.32188	ns	1	ns
TG 57:3 FA18:2	0.32188	ns	1	ns
TG 58:10 FA18:2	0.313466	ns	1	ns
TG 58:10 FA20:4	0.198506	ns	1	ns
TG 58:10 FA20:5	0.166666	ns	1	ns

TG 58:10 FA22:5	0.174001	ns	1	ns
TG 58:10 FA22:6	0.417657	ns	1	ns
TG 58:2 FA18:1	0.505152	ns	1	ns
TG 58:3 FA18:1	0.814874	ns	1	ns
TG 58:5 FA18:1	0.543265	ns	1	ns
TG 58:6 FA16:0	0.67869	ns	1	ns
TG 58:6 FA18:0	0.853808	ns	1	ns
TG 58:6 FA18:1	0.963258	ns	1	ns
TG 58:6 FA20:4	0.289503	ns	1	ns
TG 58:6 FA22:4	0.979906	ns	1	ns
TG 58:6 FA22:5	0.70526	ns	1	ns
TG 58:7 FA16:0	0.928244	ns	1	ns
TG 58:7 FA18:0	0.98563	ns	1	ns
TG 58:7 FA18:1	0.89692	ns	1	ns
TG 58:7 FA18:2	0.788161	ns	1	ns
TG 58:7 FA20:4	0.174422	ns	1	ns
TG 58:7 FA22:4	0.805462	ns	1	ns
TG 58:7 FA22:5	0.815709	ns	1	ns
TG 58:7 FA22:6	0.924933	ns	1	ns
TG 58:8 FA18:1	1	ns	1	ns
TG 58:8 FA18:2	0.70526	ns	1	ns
TG 58:8 FA20:3	0.540463	ns	1	ns
TG 58:8 FA20:4	0.353279	ns	1	ns
TG 58:8 FA22:5	0.842951	ns	1	ns
TG 58:8 FA22:6	0.55227	ns	1	ns
TG 58:9 FA18:1	0.438652	ns	1	ns
TG 58:9 FA18:2	0.505152	ns	1	ns
TG 58:9 FA20:4	0.480731	ns	1	ns
TG 58:9 FA22:5	0.460242	ns	1	ns
TG 58:9 FA22:6	0.576613	ns	1	ns
TG 60:10 FA22:5	0.335849	ns	1	ns
TG 60:10 FA22:6	0.274904	ns	1	ns
TG 60:11 FA22:5	0.04941	*	1	ns
TG 60:11 FA22:6	0.323214	ns	1	ns
TG 60:12 FA22:6	0.241331	ns	1	ns
