# Supplementary material for: Anxiety is associated with systemic pro-inflammatory profile and plasma lipid changes in Mexican young adults
Source: Front Cell Neurosci. 2026 Feb 27;20:1777048. doi: 10.3389/fncel.2026.1777048 (PMC12982057; doi:10.3389/fncel.2026.1777048)
Supplement: Supplementary file 3 [file Data_Sheet_3.pdf]

**Independent Variable**	**rho**	***p***
DHCer 12:0	0.1397	0.4381
DHCer 14:0	0.0690	0.7028
DHCer 14:1	0.0730	0.6866
DHCer 15:0	-0.0427	0.8136
DHCer 16:0	-0.0132	0.9417
DHCer 16:1	0.0334	0.8537
DHCer 17:0	0.1923	0.3366
DHCer 18:0	0.1117	0.5361
DHCer 18:1	0.0896	0.6198
DHCer 18:2	0.1017	0.5734
DHCer 18:3	0.0478	0.7915
DHCer 18:4	0.1376	0.4527
DHCer 20:0	0.0828	0.6470
DHCer 20:1	0.0750	0.6782
DHCer 20:2	0.0645	0.7213
DHCer 20:3	0.0692	0.7021
DHCer 20:4	-0.0810	0.6539
DHCer 20:5	-0.0408	0.8217
DHCer 22:0	-0.1241	0.4916
DHCer 22:1	-0.1180	0.5130
DHCer 22:2	-0.1261	0.4915
DHCer 22:4	0.1536	0.3933
DHCer 22:5	0.1554	0.3879
DHCer 22:6	-0.0280	0.8769
DHCer 24:0	-0.1111	0.5380
DHCer 24:1	-0.0549	0.7616
Cer 16:0	0.0201	0.9115
Cer 18:0	0.1600	0.3737
Cer 20:0	0.1368	0.4478
Cer 22:0	0.0681	0.7064
Cer 22:1	0.0566	0.7544
Cer 24:0	0.0368	0.8388
Cer 24:1	0.0642	0.7227
Cer 26:0	0.1098	0.5431
Cer 26:1	-0.0767	0.6712
DG 12:0 16:0	0.2421	0.1975
DG 12:0 18:0	-0.0953	0.6578
DG 12:0 18:1	0.1418	0.4313
DG 12:0 18:2	0.1194	0.5081
DG 14:0 14:0	0.1509	0.4019
DG 14:0 16:0	-0.0010	0.9955
DG 14:0 16:1	0.1253	0.5688
DG 14:0 18:1	0.1428	0.4279
DG 14:0 18:2	0.1469	0.4145
DG 14:0 20:0	0.0106	0.9541
DG 14:1 16:0	-0.0970	0.5911
DG 14:1 18:1	0.0831	0.6457
DG 14:1 20:0	-0.0267	0.8829
DG 15:0 18:1	0.1043	0.5636

DG 16:0 16:0	0.0322	0.8589
DG 16:0 16:1	0.0750	0.6782
DG 16:0 18:0	-0.1602	0.3732
DG 16:0 18:1	0.0993	0.5825
DG 16:0 18:2	0.0554	0.7594
DG 16:0 18:3	0.0526	0.7711
DG 16:0 20:0	-0.1523	0.3976
DG 16:0 20:3	0.1108	0.5598
DG 16:0 20:4	-0.0339	0.8588
DG 16:0 20:5	-0.1071	0.6531
DG 16:0 22:5	0.0539	0.7696
DG 16:0 22:6	-0.0870	0.6537
DG 16:1 16:1	0.0121	0.9475
DG 16:1 18:0	-0.0330	0.8552
DG 16:1 18:1	0.0404	0.8232
DG 16:1 18:2	0.0186	0.9182
DG 16:1 18:3	0.0028	0.9888
DG 16:1 20:0	0.0424	0.8270
DG 16:1 20:2	0.2985	0.1028
DG 16:1 20:4	-0.2523	0.1952
DG 16:1 22:6	-0.1338	0.5058
DG 18:0 18:1	0.1222	0.4983
DG 18:0 18:2	0.0552	0.7602
DG 18:0 18:3	0.0863	0.6501
DG 18:0 20:0	-0.0582	0.7869
DG 18:0 22:6	0.0170	0.9263
DG 18:1 18:1	0.1622	0.3670
DG 18:1 18:2	0.0482	0.7901
DG 18:1 20:0	-0.0460	0.8092
DG 18:1 20:1	-0.0391	0.8291
DG 18:1 20:2	-0.0322	0.8589
DG 18:1 20:3	0.0466	0.7967
DG 18:1 20:4	-0.1124	0.5336
DG 18:1 20:5	-0.0983	0.6054
DG 18:1 22:4	0.0060	0.9770
DG 18:1 22:5	0.0738	0.6831
DG 18:1 22:6	-0.0626	0.7292
DG 18:2 18:3	-0.0623	0.7306
DG 18:2 20:0	-0.1350	0.5703
DG 18:2 20:3	0.0021	0.9909
DG 18:2 20:4	-0.1905	0.3047
DG 18:2 20:5	-0.1004	0.5974
DG 18:2 22:4	-0.3027	0.1174
DG 18:2 22:5	0.0168	0.9271
DG 18:2 22:6	-0.2212	0.2238
DG 20:1 20:0	0.0758	0.7884
DG 20:5 20:0	0.0817	0.6853
DG 22:1 20:0	0.0526	0.8163
DG 22:6 20:0	0.0709	0.6998
FA 12:0	-0.0162	0.9288

FA 14:0	-0.1516	0.3997
FA 14:1	-0.0281	0.8747
FA 15:0	0.0735	0.6795
FA 16:0	-0.1303	0.4627
FA 16:1	-0.1712	0.3331
FA 17:0	-0.0202	0.9098
FA 18:0	-0.1767	0.3175
FA 18:1	-0.1901	0.2816
FA 18:2	-0.2458	0.1612
FA 18:3	-0.2546	0.1527
FA 18:4	-0.1977	0.2625
FA 20:0	-0.1147	0.5184
FA 20:1	-0.2384	0.1746
FA 20:2	-0.2420	0.1680
FA 20:3	-0.0139	0.9379
FA 20:4	-0.2010	0.2544
FA 20:5	-0.0393	0.8254
FA 22:0	-0.1994	0.2582
FA 22:1	-0.1593	0.3681
FA 22:2	-0.3127	0.0718
FA 22:4	-0.2109	0.2312
FA 22:5	-0.0251	0.8880
FA 22:6	-0.1735	0.3264
FA 24:0	-0.0497	0.7802
FA 24:1	-0.0969	0.5858
HexCer 16:0	0.1301	0.6186
HexCer 18:0	0.1850	0.5450
HexCer 22:0	0.1952	0.4528
HexCer 24:0	0.1952	0.4528
HexCer 24:1	0.1709	0.5118
LPC 14:0	-0.3059	0.0834
LPC 15:0	0.0630	0.7277
LPC 16:0	0.0771	0.6698
LPC 16:1	0.0344	0.8492
LPC 17:0	0.1729	0.3359
LPC 18:0	0.1676	0.3512
LPC 18:1	0.0215	0.9054
LPC 18:2	-0.1208	0.5031
LPC 18:3	-0.0804	0.6567
LPC 19:0	0.0508	0.7823
LPC 20:0	-0.1247	0.4891
LPC 20:1	-0.0833	0.6450
LPC 20:2	-0.1812	0.3130
LPC 20:3	0.0138	0.9394
LPC 20:4	-0.1395	0.4386
LPC 20:5	-0.1203	0.5050
LPC 22:0	-0.1622	0.3670
LPC 22:1	-0.2517	0.1576
LPC 22:2	-0.2273	0.2541
LPC 22:4	-0.1839	0.3055

LPC 22:5	0.0520	0.7740
LPC 22:6	-0.0045	0.9803
LPC 24:0	-0.2232	0.2119
LPC 24:1	-0.3248	0.0746
LPE 14:1	-0.3246	0.2036
LPE 16:0	-0.0819	0.6558
LPE 18:0	0.0546	0.7667
LPE 18:1	-0.1301	0.4706
LPE 18:2	-0.0642	0.7227
LPE 18:3	-0.0718	0.7915
LPE 20:3	0.0096	0.9584
LPE 20:4	-0.2142	0.2313
LPE 20:5	-0.1552	0.3885
LPE 22:4	-0.3274	0.1273
LPE 22:5	-0.2451	0.2087
LPE 22:6	-0.3728	0.0425
TG 36:0 FA12:0	0.0872	0.6293
TG 38:0 FA12:0	0.0576	0.7500
TG 40:0 FA12:0	0.1182	0.5124
TG 40:0 FA14:0	0.0590	0.7442
TG 40:0 FA16:0	0.0268	0.8821
TG 42:0 FA12:0	0.1196	0.5074
TG 42:0 FA14:0	0.0740	0.6824
TG 42:0 FA16:0	0.0428	0.8129
TG 42:1 FA12:0	0.0663	0.7186
TG 42:1 FA14:0	0.1268	0.4819
TG 42:1 FA16:0	0.0282	0.8761
TG 42:1 FA16:1	0.1032	0.5675
TG 42:1 FA18:1	0.1099	0.5425
TG 42:2 FA12:0	0.0629	0.7702
TG 42:2 FA18:2	0.0678	0.7078
TG 44:0 FA12:0	0.1204	0.5044
TG 44:0 FA14:0	0.0363	0.8410
TG 44:0 FA16:0	0.0908	0.6151
TG 44:0 FA18:0	0.1309	0.4677
TG 44:1 FA12:0	0.1315	0.4659
TG 44:1 FA14:0	0.1392	0.4398
TG 44:1 FA14:1	0.1345	0.4554
TG 44:1 FA16:0	0.0946	0.6004
TG 44:1 FA16:1	0.0912	0.6138
TG 44:1 FA18:1	0.1053	0.5598
TG 44:2 FA12:0	0.0874	0.6286
TG 44:2 FA14:0	0.0838	0.6485
TG 44:2 FA16:0	0.0418	0.8173
TG 44:2 FA16:1	0.0989	0.5839
TG 44:2 FA18:1	0.0652	0.7184
TG 44:2 FA18:2	0.0800	0.6581
TG 44:3 FA18:2	0.1228	0.5031
TG 45:0 FA14:0	0.0953	0.5977
TG 45:0 FA15:0	0.0602	0.7392

TG 45:0 FA16:0	0.0392	0.8284
TG 45:1 FA15:0	0.1129	0.5317
TG 45:1 FA16:0	0.0845	0.6402
TG 45:1 FA18:1	0.0977	0.5885
TG 46:0 FA12:0	0.2058	0.2506
TG 46:0 FA14:0	0.0877	0.6273
TG 46:0 FA16:0	0.0898	0.6192
TG 46:0 FA18:0	0.1041	0.5643
TG 46:1 FA12:0	0.1358	0.4513
TG 46:1 FA14:0	0.1075	0.5514
TG 46:1 FA14:1	0.0592	0.7435
TG 46:1 FA16:0	0.0867	0.6313
TG 46:1 FA16:1	0.0521	0.7732
TG 46:1 FA18:0	0.0917	0.6118
TG 46:1 FA18:1	0.1339	0.4577
TG 46:2 FA12:0	0.0717	0.6915
TG 46:2 FA14:0	0.0545	0.7631
TG 46:2 FA14:1	0.1012	0.5753
TG 46:2 FA16:0	0.0650	0.7192
TG 46:2 FA16:1	0.1120	0.5349
TG 46:2 FA18:1	0.1099	0.5425
TG 46:2 FA18:2	0.0674	0.7092
TG 46:3 FA12:0	0.1743	0.3569
TG 46:3 FA14:0	-0.0023	0.9902
TG 46:3 FA14:1	0.0609	0.7363
TG 46:3 FA16:0	0.0115	0.9492
TG 46:3 FA16:1	0.1068	0.5540
TG 46:3 FA18:1	0.0535	0.7674
TG 46:3 FA18:2	0.0592	0.7435
TG 46:3 FA18:3	0.1295	0.5197
TG 46:4 FA18:2	0.0969	0.5978
TG 47:0 FA14:0	0.1294	0.4730
TG 47:0 FA15:0	0.0986	0.5852
TG 47:0 FA16:0	0.1044	0.5630
TG 47:0 FA17:0	0.0848	0.6388
TG 47:1 FA14:0	0.1438	0.4245
TG 47:1 FA15:0	0.1449	0.4212
TG 47:1 FA16:0	0.1081	0.5495
TG 47:1 FA16:1	0.0876	0.6279
TG 47:1 FA17:0	0.1401	0.4369
TG 47:1 FA18:1	0.1039	0.5649
TG 47:2 FA14:0	0.0859	0.6347
TG 47:2 FA15:0	0.0838	0.6429
TG 47:2 FA16:1	0.0902	0.6178
TG 47:2 FA18:1	0.0881	0.6259
TG 47:2 FA18:2	0.0812	0.6532
TG 48:0 FA14:0	0.1048	0.5617
TG 48:0 FA16:0	0.0697	0.7000
TG 48:0 FA18:0	0.0862	0.6334
TG 48:1 FA12:0	0.1352	0.4530

TG 48:1 FA14:0	0.0915	0.6124
TG 48:1 FA14:1	0.0643	0.7220
TG 48:1 FA16:0	0.0497	0.7835
TG 48:1 FA16:1	0.0516	0.7754
TG 48:1 FA18:0	0.1339	0.4577
TG 48:1 FA18:1	0.0967	0.5924
TG 48:2 FA12:0	0.1542	0.3917
TG 48:2 FA14:0	0.0690	0.7028
TG 48:2 FA14:1	0.0798	0.6588
TG 48:2 FA16:0	0.0506	0.7798
TG 48:2 FA16:1	0.0475	0.7930
TG 48:2 FA18:0	0.0907	0.6158
TG 48:2 FA18:1	0.1182	0.5124
TG 48:2 FA18:2	0.0372	0.8373
TG 48:3 FA12:0	0.1134	0.5298
TG 48:3 FA14:0	0.0323	0.8582
TG 48:3 FA14:1	-0.0017	0.9924
TG 48:3 FA16:0	0.0996	0.6004
TG 48:3 FA16:1	0.0518	0.7747
TG 48:3 FA18:1	0.1134	0.5298
TG 48:3 FA18:2	0.0724	0.6887
TG 48:3 FA18:3	0.0368	0.8388
TG 48:4 FA12:0	0.1673	0.3601
TG 48:4 FA14:0	-0.0622	0.7440
TG 48:4 FA14:1	-0.0100	0.9560
TG 48:4 FA16:0	-0.0466	0.7967
TG 48:4 FA16:1	-0.0112	0.9507
TG 48:4 FA18:1	0.1720	0.3465
TG 48:4 FA18:2	0.0416	0.8180
TG 48:4 FA18:3	0.1192	0.5158
TG 48:4 FA20:4	0.0111	0.9588
TG 48:5 FA18:2	0.0607	0.7500
TG 48:5 FA18:3	0.1223	0.5196
TG 49:0 FA15:0	0.1561	0.3858
TG 49:0 FA16:0	0.1573	0.3821
TG 49:0 FA17:0	0.1259	0.4849
TG 49:0 FA18:0	0.1667	0.3538
TG 49:1 FA14:0	0.1328	0.4612
TG 49:1 FA15:0	0.1222	0.4983
TG 49:1 FA16:0	0.1299	0.4712
TG 49:1 FA16:1	0.1111	0.5380
TG 49:1 FA17:0	0.1342	0.4565
TG 49:1 FA18:1	0.1573	0.3821
TG 49:2 FA14:0	0.1223	0.4976
TG 49:2 FA15:0	0.0573	0.7515
TG 49:2 FA16:0	0.0650	0.7192
TG 49:2 FA16:1	0.1223	0.4976
TG 49:2 FA17:0	0.1198	0.5068
TG 49:2 FA18:1	0.1192	0.5087
TG 49:2 FA18:2	0.0719	0.6908

TG 49:3 FA15:0	-0.0043	0.9812
TG 49:3 FA16:0	-0.0167	0.9266
TG 49:3 FA16:1	0.0721	0.6901
TG 49:3 FA18:2	0.0540	0.7652
TG 49:3 FA18:3	0.1159	0.5419
TG 50:0 FA14:0	0.1272	0.4807
TG 50:0 FA16:0	0.0654	0.7177
TG 50:0 FA18:0	0.0802	0.6574
TG 50:1 FA14:0	0.1294	0.4730
TG 50:1 FA16:0	0.0578	0.7493
TG 50:1 FA16:1	0.1024	0.5708
TG 50:1 FA18:0	0.1029	0.5688
TG 50:1 FA18:1	0.0296	0.8701
TG 50:1 FA20:1	0.0719	0.6908
TG 50:2 FA14:0	0.1550	0.3890
TG 50:2 FA14:1	0.1481	0.4107
TG 50:2 FA16:0	0.0358	0.8433
TG 50:2 FA16:1	0.0477	0.7923
TG 50:2 FA18:0	0.0960	0.5951
TG 50:2 FA18:1	0.0988	0.5845
TG 50:2 FA18:2	0.0251	0.8896
TG 50:2 FA20:2	0.0251	0.8896
TG 50:3 FA14:0	0.0814	0.6525
TG 50:3 FA14:1	0.1173	0.5155
TG 50:3 FA16:0	-0.0175	0.9228
TG 50:3 FA16:1	0.0205	0.9100
TG 50:3 FA18:0	0.1248	0.5036
TG 50:3 FA18:1	0.0919	0.6111
TG 50:3 FA18:2	0.0465	0.7974
TG 50:3 FA18:3	-0.0053	0.9765
TG 50:3 FA20:3	-0.0484	0.7926
TG 50:4 FA14:0	0.0263	0.8844
TG 50:4 FA14:1	0.0306	0.8681
TG 50:4 FA16:0	-0.0212	0.9069
TG 50:4 FA16:1	0.0422	0.8158
TG 50:4 FA18:1	0.0208	0.9084
TG 50:4 FA18:2	0.0358	0.8433
TG 50:4 FA18:3	0.0058	0.9742
TG 50:4 FA20:3	-0.0636	0.7432
TG 50:4 FA20:4	0.0573	0.7637
TG 50:5 FA14:0	-0.0358	0.8433
TG 50:5 FA16:1	-0.0040	0.9826
TG 50:5 FA18:1	-0.0165	0.9273
TG 50:5 FA18:2	-0.0468	0.7959
TG 50:5 FA18:3	-0.0683	0.7057
TG 50:5 FA20:4	0.0297	0.8785
TG 50:5 FA20:5	-0.0796	0.6648
TG 50:6 FA20:4	-0.0705	0.7213
TG 51:0 FA16:0	0.0497	0.7835
TG 51:0 FA17:0	0.2271	0.2037

TG 51:0 FA18:0	0.1462	0.4167
TG 51:1 FA15:0	0.2049	0.2526
TG 51:1 FA16:0	0.1719	0.3388
TG 51:1 FA17:0	0.1364	0.4490
TG 51:1 FA18:0	0.1991	0.2667
TG 51:1 FA18:1	0.1832	0.3074
TG 51:2 FA15:0	0.1626	0.3660
TG 51:2 FA16:0	0.1117	0.5361
TG 51:2 FA16:1	0.1724	0.3374
TG 51:2 FA17:0	0.0917	0.6118
TG 51:2 FA18:1	0.1579	0.3800
TG 51:2 FA18:2	0.0914	0.6131
TG 51:3 FA15:0	0.0834	0.6443
TG 51:3 FA16:1	0.1084	0.5482
TG 51:3 FA17:0	0.0764	0.6726
TG 51:3 FA18:2	0.0797	0.6594
TG 51:3 FA18:3	0.1312	0.4817
TG 51:4 FA15:0	0.0096	0.9576
TG 51:4 FA16:1	0.0717	0.6915
TG 51:4 FA18:2	0.0267	0.8829
TG 51:4 FA18:3	0.1099	0.5630
TG 51:4 FA20:4	0.1081	0.5768
TG 51:5 FA18:2	-0.0172	0.9282
TG 51:5 FA18:3	-0.0584	0.7634
TG 52:0 FA16:0	0.0933	0.6057
TG 52:0 FA18:0	0.0444	0.8062
TG 52:0 FA20:0	0.0318	0.8604
TG 52:1 FA16:0	0.1167	0.5180
TG 52:1 FA16:1	0.1017	0.5734
TG 52:1 FA18:0	0.0865	0.6320
TG 52:1 FA18:1	0.1149	0.5242
TG 52:1 FA20:0	0.0585	0.7464
TG 52:1 FA20:1	0.0315	0.8619
TG 52:2 FA14:0	0.0578	0.7493
TG 52:2 FA16:0	0.1068	0.5540
TG 52:2 FA16:1	0.1065	0.5553
TG 52:2 FA18:0	0.0884	0.6246
TG 52:2 FA18:1	0.1163	0.5192
TG 52:2 FA18:2	0.0666	0.7127
TG 52:2 FA20:0	0.1046	0.5623
TG 52:2 FA20:1	0.0582	0.7479
TG 52:2 FA20:2	0.0132	0.9417
TG 52:3 FA14:0	0.0666	0.7127
TG 52:3 FA16:0	0.0322	0.8589
TG 52:3 FA16:1	0.1253	0.4873
TG 52:3 FA18:0	0.0649	0.7199
TG 52:3 FA18:1	0.0303	0.8671
TG 52:3 FA18:2	0.0272	0.8806
TG 52:3 FA18:3	0.0317	0.8612
TG 52:3 FA20:0	0.0392	0.8284

TG 52:3 FA20:1	0.0127	0.9439
TG 52:3 FA20:2	0.0415	0.8188
TG 52:3 FA20:3	-0.0136	0.9402
TG 52:3 FA22:1	0.0472	0.8009
TG 52:4 FA14:0	0.1404	0.4434
TG 52:4 FA16:0	-0.0031	0.9864
TG 52:4 FA16:1	0.0831	0.6457
TG 52:4 FA18:0	0.1166	0.5252
TG 52:4 FA18:1	0.0392	0.8284
TG 52:4 FA18:2	0.0019	0.9917
TG 52:4 FA18:3	0.0100	0.9560
TG 52:4 FA20:0	0.0778	0.6722
TG 52:4 FA20:2	0.0344	0.8519
TG 52:4 FA20:3	0.0210	0.9077
TG 52:4 FA20:4	-0.0573	0.7515
TG 52:4 FA22:1	0.0218	0.9108
TG 52:4 FA22:4	-0.0895	0.6322
TG 52:5 FA14:0	0.0719	0.7057
TG 52:5 FA16:0	-0.0633	0.7263
TG 52:5 FA16:1	0.0392	0.8284
TG 52:5 FA18:1	0.0091	0.9598
TG 52:5 FA18:2	-0.0005	0.9977
TG 52:5 FA18:3	-0.0477	0.7923
TG 52:5 FA20:3	0.0351	0.8487
TG 52:5 FA20:4	-0.0948	0.5997
TG 52:5 FA20:5	-0.0688	0.7035
TG 52:5 FA22:5	0.0089	0.9606
TG 52:6 FA14:0	-0.0348	0.8579
TG 52:6 FA16:1	-0.0291	0.8724
TG 52:6 FA18:1	-0.0638	0.7242
TG 52:6 FA18:2	-0.0819	0.6505
TG 52:6 FA18:3	-0.0976	0.5891
TG 52:6 FA20:4	-0.0947	0.6124
TG 52:6 FA20:5	-0.0561	0.7565
TG 52:6 FA22:6	-0.0617	0.7371
TG 52:7 FA16:0	0.1722	0.3379
TG 52:7 FA18:1	-0.0612	0.8502
TG 52:7 FA20:5	-0.0040	0.9841
TG 52:7 FA22:6	-0.0539	0.7771
TG 52:8 FA16:1	0.1583	0.3790
TG 52:8 FA18:2	0.4106	0.0334
TG 53:0 FA16:0	-0.0964	0.5938
TG 53:1 FA16:0	0.0743	0.6810
TG 53:1 FA17:0	0.1918	0.2848
TG 53:1 FA18:0	0.1915	0.2857
TG 53:1 FA18:1	0.1636	0.3629
TG 53:2 FA16:0	0.0654	0.7177
TG 53:2 FA17:0	0.1831	0.3078
TG 53:2 FA18:1	0.1937	0.2800
TG 53:2 FA18:2	0.0637	0.7249

TG 53:3 FA16:0	-0.0015	0.9932
TG 53:3 FA17:0	0.0797	0.6594
TG 53:3 FA18:2	0.0805	0.6560
TG 53:4 FA16:0	-0.0165	0.9273
TG 53:4 FA17:0	0.0201	0.9115
TG 53:4 FA18:2	0.0551	0.7609
TG 53:4 FA18:3	0.2243	0.2252
TG 53:4 FA20:4	0.0861	0.6509
TG 53:5 FA20:4	0.0350	0.8541
TG 53:6 FA20:4	-0.1388	0.4811
TG 54:0 FA16:0	0.0322	0.8589
TG 54:0 FA18:0	-0.0375	0.8358
TG 54:1 FA16:0	0.0960	0.5951
TG 54:1 FA18:0	0.1103	0.5412
TG 54:1 FA18:1	0.1399	0.4375
TG 54:1 FA20:0	0.0373	0.8366
TG 54:1 FA20:1	0.1803	0.3153
TG 54:2 FA16:0	0.0370	0.8381
TG 54:2 FA18:0	0.1352	0.4530
TG 54:2 FA18:1	0.1480	0.4112
TG 54:2 FA18:2	0.0014	0.9939
TG 54:2 FA20:0	-0.0329	0.8559
TG 54:2 FA20:1	0.0456	0.8011
TG 54:2 FA20:2	0.1769	0.3248
TG 54:3 FA16:0	0.0437	0.8092
TG 54:3 FA16:1	0.1437	0.4251
TG 54:3 FA18:0	0.0929	0.6071
TG 54:3 FA18:1	0.1438	0.4245
TG 54:3 FA18:2	0.0979	0.5878
TG 54:3 FA18:3	0.1038	0.5656
TG 54:3 FA20:1	0.0067	0.9704
TG 54:3 FA20:2	0.0241	0.8941
TG 54:3 FA20:3	-0.0022	0.9901
TG 54:4 FA16:0	0.0048	0.9788
TG 54:4 FA16:1	0.0425	0.8143
TG 54:4 FA18:0	0.0719	0.6908
TG 54:4 FA18:1	0.0910	0.6144
TG 54:4 FA18:2	0.0731	0.6859
TG 54:4 FA18:3	0.0471	0.7945
TG 54:4 FA20:1	0.0328	0.8584
TG 54:4 FA20:2	0.0353	0.8455
TG 54:4 FA20:3	0.0354	0.8448
TG 54:4 FA20:4	-0.0280	0.8769
TG 54:4 FA22:1	0.2688	0.1437
TG 54:4 FA22:4	-0.0674	0.7140
TG 54:5 FA16:0	-0.0613	0.7349
TG 54:5 FA16:1	0.0206	0.9092
TG 54:5 FA18:0	-0.0248	0.8911
TG 54:5 FA18:1	-0.0089	0.9606
TG 54:5 FA18:2	0.0212	0.9069

TG 54:5 FA18:3	0.0456	0.8011
TG 54:5 FA20:2	0.0327	0.8567
TG 54:5 FA20:3	-0.0191	0.9160
TG 54:5 FA20:4	-0.0874	0.6286
TG 54:5 FA20:5	-0.0502	0.7813
TG 54:5 FA22:1	-0.0181	0.9205
TG 54:5 FA22:4	-0.0587	0.7495
TG 54:5 FA22:5	0.0361	0.8418
TG 54:6 FA16:0	-0.1370	0.4472
TG 54:6 FA16:1	0.0717	0.6965
TG 54:6 FA18:1	0.0102	0.9553
TG 54:6 FA18:2	-0.0263	0.8844
TG 54:6 FA18:3	0.0065	0.9712
TG 54:6 FA20:3	0.0058	0.9742
TG 54:6 FA20:4	-0.1808	0.3139
TG 54:6 FA20:5	-0.0752	0.6775
TG 54:6 FA22:5	0.0353	0.8455
TG 54:6 FA22:6	-0.0864	0.6327
TG 54:7 FA16:1	-0.1683	0.3572
TG 54:7 FA18:2	-0.0693	0.7014
TG 54:7 FA18:3	-0.0721	0.6901
TG 54:7 FA20:4	-0.1585	0.3784
TG 54:7 FA20:5	-0.1318	0.4647
TG 54:7 FA22:5	0.0513	0.7841
TG 54:7 FA22:6	-0.0456	0.8011
TG 54:8 FA18:2	-0.0838	0.6429
TG 54:8 FA18:3	-0.0791	0.6615
TG 54:8 FA20:4	-0.2843	0.1350
TG 54:8 FA20:5	-0.2193	0.2622
TG 54:8 FA22:6	0.0174	0.9272
TG 55:1 FA16:0	-0.0662	0.7142
TG 55:1 FA18:1	-0.0640	0.7234
TG 55:2 FA18:1	0.1256	0.4861
TG 55:2 FA18:2	-0.1526	0.3965
TG 55:3 FA18:1	0.1070	0.5533
TG 55:3 FA18:2	-0.0191	0.9160
TG 55:4 FA18:1	0.0545	0.7631
TG 55:4 FA18:2	0.0329	0.8559
TG 55:5 FA18:1	0.0853	0.6423
TG 55:5 FA18:2	0.0005	0.9977
TG 55:5 FA20:4	0.0479	0.8016
TG 55:7 FA15:0	-0.0201	0.9115
TG 55:7 FA22:6	-0.1248	0.5035
TG 56:1 FA16:0	-0.0014	0.9939
TG 56:1 FA18:1	0.0108	0.9523
TG 56:10 FA18:2	0.0077	0.9659
TG 56:2 FA16:0	-0.0062	0.9727
TG 56:2 FA18:0	0.0950	0.5991
TG 56:2 FA20:0	0.0570	0.7529
TG 56:2 FA20:1	0.0738	0.6831

TG 56:3 FA16:0	0.0923	0.6276
TG 56:3 FA18:0	0.1223	0.4976
TG 56:3 FA18:1	0.0680	0.7071
TG 56:3 FA18:2	-0.0022	0.9901
TG 56:3 FA20:0	0.0183	0.9208
TG 56:3 FA20:1	0.0869	0.6307
TG 56:3 FA20:2	0.1427	0.4519
TG 56:4 FA16:0	-0.0318	0.8628
TG 56:4 FA18:0	0.0993	0.5825
TG 56:4 FA18:1	0.0862	0.6334
TG 56:4 FA18:2	0.0384	0.8321
TG 56:4 FA20:1	0.0785	0.6643
TG 56:4 FA20:2	0.0900	0.6185
TG 56:4 FA20:3	0.2099	0.2490
TG 56:4 FA20:4	0.0143	0.9371
TG 56:4 FA22:4	0.0777	0.6831
TG 56:5 FA16:0	0.0298	0.8694
TG 56:5 FA18:0	0.0998	0.5806
TG 56:5 FA18:1	0.0590	0.7442
TG 56:5 FA18:2	0.0456	0.8011
TG 56:5 FA20:1	-0.0284	0.8754
TG 56:5 FA20:2	0.0461	0.7989
TG 56:5 FA20:3	0.0898	0.6192
TG 56:5 FA20:4	0.0819	0.6505
TG 56:5 FA22:4	0.0222	0.9024
TG 56:5 FA22:5	0.0251	0.8896
TG 56:6 FA16:0	0.0657	0.7163
TG 56:6 FA18:0	-0.0628	0.7508
TG 56:6 FA18:1	0.1274	0.4873
TG 56:6 FA18:2	0.0071	0.9689
TG 56:6 FA18:3	-0.0722	0.7044
TG 56:6 FA20:2	0.0084	0.9629
TG 56:6 FA20:3	-0.0057	0.9750
TG 56:6 FA20:4	-0.0344	0.8492
TG 56:6 FA20:5	-0.0286	0.8746
TG 56:6 FA22:4	-0.0640	0.7234
TG 56:6 FA22:5	0.0824	0.6484
TG 56:6 FA22:6	0.0120	0.9470
TG 56:7 FA16:0	-0.0040	0.9826
TG 56:7 FA16:1	0.0630	0.7277
TG 56:7 FA18:0	-0.0009	0.9962
TG 56:7 FA18:1	-0.0852	0.6375
TG 56:7 FA18:2	-0.0616	0.7335
TG 56:7 FA18:3	-0.0653	0.7366
TG 56:7 FA20:3	-0.0609	0.7363
TG 56:7 FA20:4	-0.1574	0.3816
TG 56:7 FA20:5	-0.0420	0.8166
TG 56:7 FA22:4	0.0350	0.8541
TG 56:7 FA22:5	0.0518	0.7747
TG 56:7 FA22:6	-0.0213	0.9062

TG 56:8 FA16:0	-0.0939	0.6031
TG 56:8 FA16:1	0.0267	0.8829
TG 56:8 FA18:2	-0.1289	0.4748
TG 56:8 FA18:3	-0.1832	0.3156
TG 56:8 FA20:4	-0.2240	0.2101
TG 56:8 FA20:5	-0.1342	0.4565
TG 56:8 FA22:5	-0.0019	0.9918
TG 56:8 FA22:6	-0.0621	0.7313
TG 56:9 FA18:3	-0.2620	0.1697
TG 56:9 FA20:4	-0.1936	0.2804
TG 56:9 FA20:5	-0.1892	0.2997
TG 56:9 FA22:6	-0.0592	0.7435
TG 57:2 FA18:1	-0.0755	0.6761
TG 57:3 FA18:2	-0.0585	0.7464
TG 58:10 FA18:2	-0.1037	0.5787
TG 58:10 FA20:4	-0.2149	0.2542
TG 58:10 FA20:5	-0.2479	0.2124
TG 58:10 FA22:5	-0.0292	0.8740
TG 58:10 FA22:6	-0.0375	0.8358
TG 58:2 FA18:1	0.0031	0.9864
TG 58:3 FA18:1	0.0516	0.7754
TG 58:5 FA18:1	0.0912	0.6197
TG 58:6 FA16:0	0.0876	0.6279
TG 58:6 FA18:0	0.1643	0.4034
TG 58:6 FA18:1	0.0645	0.7444
TG 58:6 FA20:4	-0.0496	0.7984
TG 58:6 FA22:4	0.0356	0.8440
TG 58:6 FA22:5	0.1795	0.3177
TG 58:7 FA16:0	0.0009	0.9962
TG 58:7 FA18:0	0.1034	0.5669
TG 58:7 FA18:1	0.0532	0.7689
TG 58:7 FA18:2	0.1426	0.4440
TG 58:7 FA20:4	-0.2100	0.2834
TG 58:7 FA22:4	-0.0246	0.8953
TG 58:7 FA22:5	0.1242	0.4910
TG 58:7 FA22:6	0.1359	0.4584
TG 58:8 FA18:2	0.0215	0.9054
TG 58:8 FA20:3	-0.0959	0.6208
TG 58:8 FA20:4	-0.1801	0.3499
TG 58:8 FA22:5	0.0828	0.6470
TG 58:8 FA22:6	-0.0100	0.9560
TG 58:9 FA18:1	-0.0270	0.8814
TG 58:9 FA18:2	0.0036	0.9841
TG 58:9 FA20:4	-0.1283	0.4993
TG 58:9 FA22:5	0.0102	0.9553
TG 58:9 FA22:6	0.0112	0.9507
TG 60:10 FA22:5	0.0311	0.8727
TG 60:10 FA22:6	-0.1341	0.5048
TG 60:11 FA22:5	-0.1423	0.4791
TG 60:11 FA22:6	-0.0930	0.6378

TG 60:12 FA22:6	-0.0907	0.6463

**significance**
ns
ns
ns
ns
ns
ns
ns
ns
ns
ns
ns
ns
ns
ns
ns
ns
ns
ns
ns
ns
ns
ns
ns
ns
ns
ns
ns
ns
ns
ns
ns
ns
ns
ns
ns
ns
ns
ns
ns
ns
ns
ns
ns
ns
ns
ns
ns
ns
ns

ns
ns
ns
ns
ns
ns
ns
ns
ns
ns
ns
ns
ns
ns
ns
ns
ns
ns
ns
ns
ns
ns
ns
ns
ns
ns
ns
ns
ns
ns
ns
ns
ns
ns
ns
ns
ns
ns
ns
ns
ns
ns
ns
ns
ns
ns
ns
ns
ns
ns

ns
ns
ns
ns
ns
ns
ns
ns
ns
ns
ns
ns
ns
ns
ns
ns
ns
ns
ns
ns
ns
ns
ns
ns
ns
ns
ns
ns
ns
ns
ns
ns
ns
ns
ns
ns
ns
ns
ns
ns
ns
ns
ns
ns
ns
ns
ns
ns
ns
ns

ns
ns
ns
ns
ns
ns
ns
ns
ns
ns
ns
ns
ns
ns
ns
*
ns
ns
ns
ns
ns
ns
ns
ns
ns
ns
ns
ns
ns
ns
ns
ns
ns
ns
ns
ns
ns
ns
ns
ns
ns
ns
ns
ns
ns
ns
ns
ns
ns
ns

ns
ns
ns
ns
ns
ns
ns
ns
ns
ns
ns
ns
ns
ns
ns
ns
ns
ns
ns
ns
ns
ns
ns
ns
ns
ns
ns
ns
ns
ns
ns
ns
ns
ns
ns
ns
ns
ns
ns
ns
ns
ns
ns
ns
ns
ns
ns
ns
ns
ns

ns
ns
ns
ns
ns
ns
ns
ns
ns
ns
ns
ns
ns
ns
ns
ns
ns
ns
ns
ns
ns
ns
ns
ns
ns
ns
ns
ns
ns
ns
ns
ns
ns
ns
ns
ns
ns
ns
ns
ns
ns
ns
ns
ns
ns
ns
ns
ns
ns
ns

ns
ns
ns
ns
ns
ns
ns
ns
ns
ns
ns
ns
ns
ns
ns
ns
ns
ns
ns
ns
ns
ns
ns
ns
ns
ns
ns
ns
ns
ns
ns
ns
ns
ns
ns
ns
ns
ns
ns
ns
ns
ns
ns
ns
ns
ns
ns
ns
ns
ns

ns
ns
ns
ns
ns
ns
ns
ns
ns
ns
ns
ns
ns
ns
ns
ns
ns
ns
ns
ns
ns
ns
ns
ns
ns
ns
ns
ns
ns
ns
ns
ns
ns
ns
ns
ns
ns
ns
ns
ns
ns
ns
ns
ns
ns
ns
ns
ns
ns
ns

ns
ns
ns
ns
ns
ns
ns
ns
ns
ns
ns
ns
ns
ns
ns
ns
ns
ns
ns
ns
ns
ns
ns
ns
ns
ns
ns
ns
ns
ns
ns
ns
ns
ns
ns
ns
ns
ns
ns
ns
*
ns
ns
ns
ns
ns
ns
ns
ns
ns

ns
ns
ns
ns
ns
ns
ns
ns
ns
ns
ns
ns
ns
ns
ns
ns
ns
ns
ns
ns
ns
ns
ns
ns
ns
ns
ns
ns
ns
ns
ns
ns
ns
ns
ns
ns
ns
ns
ns
ns
ns
ns
ns
ns
ns
ns
ns
ns
ns
ns

ns
ns
ns
ns
ns
ns
ns
ns
ns
ns
ns
ns
ns
ns
ns
ns
ns
ns
ns
ns
ns
ns
ns
ns
ns
ns
ns
ns
ns
ns
ns
ns
ns
ns
ns
ns
ns
ns
ns
ns
ns
ns
ns
ns
ns
ns
ns
ns
ns
ns

ns
ns
ns
ns
ns
ns
ns
ns
ns
ns
ns
ns
ns
ns
ns
ns
ns
ns
ns
ns
ns
ns
ns
ns
ns
ns
ns
ns
ns
ns
ns
ns
ns
ns
ns
ns
ns
ns
ns
ns
ns
ns
ns
ns
ns
ns
ns
ns
ns
ns

ns
ns
ns
ns
ns
ns
ns
ns
ns
ns
ns
ns
ns
ns
ns
ns
ns
ns
ns
ns
ns
ns
ns
ns
ns
ns
ns
ns
ns
ns
ns
ns
ns
ns
ns
ns
ns
ns
ns
ns
ns
ns
ns
ns
ns
ns
ns
ns
ns
ns

ns
