# Supplementary material for: Anxiety is associated with systemic pro-inflammatory profile and plasma lipid changes in Mexican young adults
Source: Front Cell Neurosci. 2026 Feb 27;20:1777048. doi: 10.3389/fncel.2026.1777048 (PMC12982057; doi:10.3389/fncel.2026.1777048)
Supplement: Supplementary file 4 [file Data_Sheet_4.pdf]

X1	X2	Beta_X1	SE_X1	P_X1	Beta_X2	SE_X2	P_X2
DHCer 12:0	IL-17A	0.853036683	0.838259979	0.316991926	0.00091621	0.003301051	0.783260724
DHCer 12:0	IL-1β	0.518490012	0.813542056	0.528748005	0.064164574	0.031495938	0.050532619
DHCer 12:0	IL-6	0.725633589	0.612214558	0.245218747	0.018963047	0.003403534	4.6413E-06
DHCer 12:0	MCP1	0.905753236	0.54004591	0.103898623	0.003664386	0.000555698	2.6837E-07
DHCer 12:0	TNF-α	0.859229843	0.774691264	0.276188352	0.111451417	0.026984784	0.000266702
DHCer 14:0	IL-17A	0.000854269	0.056424675	0.988020745	0.00192666	0.004182931	0.648406788
DHCer 14:0	IL-1β	0.006429035	0.047042083	0.892208153	0.07586881	0.034284609	0.034655353
DHCer 14:0	IL-6	0.005107792	0.036310221	0.889070225	0.018999459	0.003800086	2.33135E-05
DHCer 14:0	MCP1	0.016785926	0.031401456	0.596894825	0.003651445	0.00060827	1.38263E-06
DHCer 14:0	TNF-α	0.020645574	0.041399915	0.621637274	0.111794334	0.027147376	0.00027578
DHCer 14:1	IL-17A	1.28133E-15	0.547656162	1	3.21245E-17	0.004347413	1
DHCer 14:1	IL-1β	-0.063771781	0.426569121	0.882160197	0.072215279	0.03328996	0.038114339
DHCer 14:1	IL-6	0.012504536	0.341115543	0.971000721	0.018920295	0.003822762	2.68756E-05
DHCer 14:1	MCP1	0.123190416	0.291901963	0.676014676	0.003676404	0.000605473	1.14044E-06
DHCer 14:1	TNF-α	0.050732603	0.380188412	0.894735988	0.108319071	0.026695526	0.000325887
DHCer 15:0	IL-1β	-0.037381107	0.13897646	0.789790632	0.075074143	0.035078674	0.040591049
DHCer 15:0	IL-6	0.024862021	0.103694189	0.812144734	0.019190739	0.003758445	1.72682E-05
DHCer 15:0	MCP1	0.055884815	0.087504393	0.527895697	0.003708432	0.000587037	5.7651E-07
DHCer 15:0	TNF-α	0.060574038	0.118430727	0.61276465	0.112766258	0.026895651	0.000224264
DHCer 16:0	IL-17A	1.40289E-05	0.003323682	0.996660163	0.001935258	0.004163878	0.645449833
DHCer 16:0	IL-1β	0.000339791	0.002701219	0.900736135	0.075820862	0.033268966	0.029949056
DHCer 16:0	IL-6	0.000291449	0.002118033	0.891472959	0.019122171	0.00374597	1.73317E-05
DHCer 16:0	MCP1	0.000239725	0.001852516	0.897900978	0.00368222	0.000606423	1.14018E-06
DHCer 16:0	TNF-α	0.00052132	0.002430246	0.831598446	0.107956245	0.026930573	0.000372835
DHCer 16:1	IL-17A	3.82727E-05	0.005600145	0.994592351	0.001927845	0.004357314	0.661345213
DHCer 16:1	IL-1β	0.000555734	0.004398025	0.900290131	0.075360333	0.033641721	0.032645982
DHCer 16:1	IL-6	-0.000998139	0.003343213	0.767336528	0.018678695	0.003672286	1.82531E-05
DHCer 16:1	MCP1	1.89927E-14	0.003033343	1	0.003708511	0.000616703	1.34284E-06
DHCer 16:1	TNF-α	0.002236322	0.003991082	0.579415327	0.110508716	0.027467996	0.000358264
DHCer 17:0	IL-17A	0.001795914	0.252732655	0.994389031	0.00193098	0.005093785	0.707957585
DHCer 17:0	IL-1β	0.055771728	0.176420784	0.754637594	0.057646604	0.041525282	0.177819775
DHCer 17:0	IL-6	0.171845788	0.114829759	0.147552608	0.022324073	0.003596847	2.05481E-06
DHCer 17:0	MCP1	0.155884374	0.115885504	0.191147759	0.004062919	0.00066607	2.66694E-06
DHCer 17:0	TNF-α	0.140596162	0.135292131	0.309066604	0.125699055	0.025530656	5.04732E-05
DHCer 18:0	IL-17A	0.00204574	0.051376576	0.96850162	0.001886859	0.00417651	0.654677843
DHCer 18:0	IL-1β	0.026854109	0.041817943	0.525641879	0.078954992	0.033420431	0.024835711
DHCer 18:0	IL-6	0.034399673	0.031515293	0.283731727	0.019850962	0.003616784	5.86461E-06
DHCer 18:0	MCP1	0.018513792	0.028419136	0.51971241	0.003629725	0.000603662	1.34513E-06
DHCer 18:0	TNF-α	0.043000229	0.036657393	0.250013688	0.112545378	0.026358865	0.00018108
DHCer 18:1	IL-17A	2.81668E-05	0.001456979	0.984703996	0.001912685	0.004256991	0.656441366
DHCer 18:1	IL-1β	0.000542046	0.00113412	0.636154132	0.074856093	0.032576863	0.028720225
DHCer 18:1	IL-6	0.000304642	0.0008802	0.731679186	0.019221197	0.003630643	1.01506E-05
DHCer 18:1	MCP1	0.000359639	0.000785028	0.650165929	0.003607064	0.000599334	1.3242E-06
DHCer 18:1	TNF-α	0.000919928	0.000977038	0.353944495	0.111557829	0.025250936	0.000119763
DHCer 18:2	IL-17A	4.08454E-06	0.000286515	0.988720179	0.001926182	0.004112071	0.642868979
DHCer 18:2	IL-1β	5.47802E-05	0.000229513	0.812976304	0.073624564	0.032383225	0.030316453
DHCer 18:2	IL-6	5.28566E-05	0.000180567	0.771747009	0.019264676	0.003658506	1.09993E-05
DHCer 18:2	MCP1	6.74292E-05	0.000161537	0.679341067	0.003638245	0.000605785	1.37173E-06

DHCer 18:2	TNF-α	0.000203175	0.00020555	0.330839281	0.115953357	0.026094356	0.000111473
DHCer 18:3	IL-17A	0.000341945	0.014467329	0.981299725	0.001935547	0.00412991	0.642696397
DHCer 18:3	IL-1β	0.004664008	0.012179929	0.70447472	0.06894006	0.034182038	0.052738266
DHCer 18:3	IL-6	0.001921484	0.009178932	0.835600728	0.019053328	0.00369911	1.52176E-05
DHCer 18:3	MCP1	0.003332732	0.00817561	0.686430152	0.003660668	0.000609828	1.38345E-06
DHCer 18:3	TNF-α	0.007753666	0.010146836	0.45074891	0.113379811	0.025621208	0.000117355
DHCer 18:4	IL-17A	0.005382628	0.384499289	0.9889266	0.001942843	0.003045398	0.528505411
DHCer 18:4	IL-1β	-0.086526788	0.407460343	0.833314619	0.053778015	0.035137865	0.136732995
DHCer 18:4	IL-6	0.007892272	0.31246719	0.980022243	0.019137569	0.003502041	6.97745E-06
DHCer 18:4	MCP1	0.031883978	0.284590687	0.911567931	0.003712266	0.000589714	7.11321E-07
DHCer 18:4	TNF-α	0.077160565	0.371969184	0.837117572	0.109786862	0.026057885	0.000223617
DHCer 20:0	IL-17A	-0.003018082	0.324420479	0.992638982	0.001944561	0.004090929	0.637991126
DHCer 20:0	IL-1β	-0.038860342	0.268374905	0.885838224	0.070649561	0.033270289	0.042071933
DHCer 20:0	IL-6	-0.004152272	0.219392413	0.985025271	0.018967368	0.003905604	3.49325E-05
DHCer 20:0	MCP1	0.053963697	0.191301464	0.779813586	0.003712991	0.000630328	1.89391E-06
DHCer 20:0	TNF-α	-0.055503328	0.25311711	0.827917649	0.107741422	0.028232668	0.000631157
DHCer 20:1	IL-17A	-0.002910277	0.347235962	0.993368284	0.00194464	0.004088765	0.637800276
DHCer 20:1	IL-1β	-0.020892171	0.284598385	0.941967691	0.071528343	0.032945858	0.037965209
DHCer 20:1	IL-6	-0.004055489	0.235752433	0.986389112	0.018969071	0.003919011	3.65633E-05
DHCer 20:1	MCP1	0.040490137	0.199966334	0.840904734	0.003713487	0.000615261	1.26215E-06
DHCer 20:1	TNF-α	-0.066229834	0.271541461	0.808966089	0.107508806	0.028282663	0.000657365
DHCer 20:2	IL-17A	0.000480776	0.897022046	0.999575907	0.001940174	0.004138535	0.642596602
DHCer 20:2	IL-1β	1.44997E-05	0.727232977	0.999984224	0.072464324	0.032985118	0.035892256
DHCer 20:2	IL-6	0.040224066	0.589623312	0.946063226	0.018878353	0.003840352	2.95482E-05
DHCer 20:2	MCP1	0.115182075	0.496413876	0.818091543	0.003673318	0.000598442	9.48283E-07
DHCer 20:2	TNF-α	0.121605956	0.668487136	0.856875254	0.108155683	0.02728056	0.000420859
DHCer 20:3	IL-17A	0.000608294	0.026721483	0.981989086	0.001908175	0.004267884	0.658013668
DHCer 20:3	IL-1β	0.011640323	0.020192194	0.568593701	0.070420339	0.031705645	0.034041007
DHCer 20:3	IL-6	0.007245033	0.015840883	0.650705238	0.019266771	0.003571777	7.65388E-06
DHCer 20:3	MCP1	0.00781039	0.01476455	0.600700685	0.003556977	0.000616179	2.63654E-06
DHCer 20:3	TNF-α	0.018417515	0.017341033	0.296676032	0.113978555	0.024498742	6.20557E-05
DHCer 20:4	IL-1β	-0.000955182	0.002282041	0.678515609	0.071194732	0.034456812	0.047537675
DHCer 20:4	IL-6	-8.10804E-05	0.001804018	0.964449499	0.018956193	0.003911511	3.5951E-05
DHCer 20:4	MCP1	-0.000389737	0.001541309	0.802100329	0.003687812	0.000618551	1.5505E-06
DHCer 20:4	TNF-α	-0.000266815	0.002062885	0.897951331	0.106678063	0.028024792	0.000647907
DHCer 20:5	IL-17A	-2.82425E-18	0.0230947	1	1.35088E-17	0.00429659	1
DHCer 20:5	IL-1β	-0.013761808	0.019315037	0.481665339	0.077243637	0.035327107	0.036711895
DHCer 20:5	IL-6	0.002021317	0.014712959	0.891645607	0.0191384	0.003864243	2.66255E-05
DHCer 20:5	MCP1	-0.000696422	0.012766832	0.956859176	0.003714917	0.000620625	1.45089E-06
DHCer 20:5	TNF-α	-0.001388416	0.01704576	0.935623263	0.108409546	0.028050761	0.000552968
DHCer 22:0	IL-17A	0.379558135	0.310458363	0.231009185	0.001861978	0.003184253	0.563091049
DHCer 22:0	IL-1β	0.161416813	0.344948402	0.643205904	0.068606884	0.034782381	0.057836041
DHCer 22:0	IL-6	0.678984302	0.241100679	0.00850912	0.019736935	0.003491045	3.68438E-06
DHCer 22:0	MCP1	0.74855628	0.224346707	0.002271585	0.003833716	0.000601255	4.89611E-07
DHCer 22:0	TNF-α	0.856463893	0.284539733	0.00525602	0.120065697	0.025814507	6.22883E-05
DHCer 22:1	IL-17A	0.078110042	0.064430893	0.234856054	0.001886101	0.003200714	0.560088541
DHCer 22:1	IL-1β	0.033444582	0.070318795	0.637794686	0.068309485	0.034341967	0.055877213
DHCer 22:1	IL-6	0.137218722	0.050527394	0.010864635	0.019438004	0.003543501	5.91492E-06
DHCer 22:1	MCP1	0.148958911	0.047369813	0.003734	0.003769287	0.000614878	9.69653E-07
DHCer 22:1	TNF-α	0.171289654	0.056471483	0.004957147	0.116085034	0.024814109	5.77185E-05

DHCer 22:2	IL-17A	-0.035996183	2.859143787	0.990041235	0.001952374	0.004307692	0.653756587
DHCer 22:2	IL-1β	-0.749356912	2.475488727	0.764271078	0.069049207	0.036735393	0.070239252
DHCer 22:2	IL-6	0.106536664	2.004683549	0.957981676	0.019046846	0.004370204	0.000150083
DHCer 22:2	MCP1	-0.128225485	1.666631968	0.93920211	0.003630309	0.000667126	7.43648E-06
DHCer 22:2	TNF-α	-0.881946155	1.983253598	0.659840354	0.109002226	0.026414563	0.000283395
DHCer 22:4	IL-17A	0.023519523	1.843295379	0.989904147	0.001922039	0.004222235	0.652229628
DHCer 22:4	IL-1β	0.495108605	1.468368458	0.73832683	0.072710644	0.033066141	0.035730657
DHCer 22:4	IL-6	0.139420305	1.153497794	0.90460196	0.01895431	0.003730071	1.85078E-05
DHCer 22:4	MCP1	0.23267391	1.01974709	0.821063525	0.003646637	0.000610344	1.49639E-06
DHCer 22:4	TNF-α	1.205267295	1.36788581	0.385259474	0.108149263	0.027714947	0.000499239
DHCer 22:5	IL-17A	0.032874121	0.283402179	0.908427045	0.001837354	0.004068266	0.65478327
DHCer 22:5	IL-1β	0.175223981	0.238306447	0.467873597	0.072603475	0.03363124	0.038989014
DHCer 22:5	IL-6	0.095340977	0.174969031	0.589849144	0.019225275	0.003545848	7.0776E-06
DHCer 22:5	MCP1	0.110953945	0.162514665	0.50001483	0.003578181	0.000609584	2.00698E-06
DHCer 22:5	TNF-α	0.309490547	0.194105674	0.121319333	0.112415832	0.02464683	8.02115E-05
DHCer 22:6	IL-1β	-0.010322228	0.034101462	0.764213769	0.073883116	0.035261489	0.044689009
DHCer 22:6	IL-6	0.006683391	0.025943691	0.798465713	0.019371818	0.003852222	2.14809E-05
DHCer 22:6	MCP1	-3.05539E-10	0.02207375	0.999999989	0.003708511	0.000606649	1.01678E-06
DHCer 22:6	TNF-α	-0.001282511	0.029814985	0.965974091	0.109164284	0.027738181	0.000455734
DHCer 24:0	IL-17A	3.56303E-15	3.220328576	1	7.31733E-17	0.004289216	1
DHCer 24:0	IL-1β	-1.674392395	2.890038122	0.566667189	0.076809666	0.037842716	0.051337302
DHCer 24:0	IL-6	-0.150305344	2.101818892	0.94346487	0.018867205	0.003952082	4.40734E-05
DHCer 24:0	MCP1	-0.044852128	1.776406571	0.98002372	0.003713788	0.000618237	1.36703E-06
DHCer 24:0	TNF-α	-0.9832059	2.380379306	0.682510426	0.103754261	0.028044044	0.000865324
DHCer 24:1	IL-17A	1.48455E-07	2.264244101	0.999999948	0.001940617	0.004099071	0.639334487
DHCer 24:1	IL-1β	0.000160835	1.857871549	0.999931501	0.072464638	0.033065784	0.03631372
DHCer 24:1	IL-6	0.447982241	1.382276641	0.748118606	0.019062213	0.003532726	7.61674E-06
DHCer 24:1	MCP1	3.9188E-09	1.281148943	0.999999998	0.003708511	0.000606034	9.99328E-07
DHCer 24:1	TNF-α	-1.9712E-10	1.718933884	1	0.109289608	0.02752568	0.000414113
Cer 16:0	IL-17A	0.019226537	2.180340718	0.993022618	0.0019292	0.004184956	0.648134336
Cer 16:0	IL-1β	0.474625947	1.692931612	0.781127577	0.074225647	0.031945299	0.027114844
Cer 16:0	IL-6	0.153346978	1.369871113	0.911614549	0.018931854	0.003711925	1.75511E-05
Cer 16:0	MCP1	-0.014535343	1.223750643	0.990601827	0.003713372	0.000613754	1.21163E-06
Cer 16:0	TNF-α	0.561295324	1.569988022	0.723208797	0.108545842	0.026655058	0.000312969
Cer 18:0	IL-17A	0.463742882	5.985929049	0.938762338	0.001749998	0.004659687	0.709885559
Cer 18:0	IL-1β	3.655714465	4.402126973	0.412848791	0.075226385	0.033689049	0.033162303
Cer 18:0	IL-6	4.48939064	3.070389737	0.154095811	0.019057391	0.003374206	3.74317E-06
Cer 18:0	MCP1	2.482293043	2.859533817	0.392244034	0.003570234	0.000581641	9.48092E-07
Cer 18:0	TNF-α	4.392504912	3.889795109	0.267746391	0.104775051	0.026783582	0.000486155
Cer 20:0	IL-17A	0.344567454	6.639864048	0.958957359	0.001855609	0.004259647	0.666227309
Cer 20:0	IL-1β	3.244924151	5.072805794	0.527241926	0.070664763	0.031993641	0.03497555
Cer 20:0	IL-6	3.883171293	3.681126323	0.299899033	0.019237603	0.003333865	2.65341E-06
Cer 20:0	MCP1	2.959586923	3.348507084	0.383804401	0.003643575	0.000561307	3.56315E-07
Cer 20:0	TNF-α	5.510226661	4.470559897	0.227312918	0.10974427	0.025368401	0.00015481
Cer 22:0	IL-17A	0.014283375	0.896011578	0.987386959	0.001918932	0.004244195	0.654426923
Cer 22:0	IL-1β	0.288454027	0.667771137	0.668855496	0.069470078	0.031096415	0.033085042
Cer 22:0	IL-6	0.180826313	0.538578887	0.739398414	0.019007881	0.003601504	1.06316E-05
Cer 22:0	MCP1	0.446117177	0.456346498	0.336097815	0.003659396	0.000564822	3.68689E-07
Cer 22:0	TNF-α	0.671486965	0.578279036	0.254724358	0.115577222	0.024229022	4.45471E-05
Cer 22:1	IL-17A	0.36296828	11.7954576	0.975655286	0.001914141	0.004150723	0.648011069

Cer 22:1	IL-1β	5.398425471	9.23664562	0.563282469	0.069302417	0.031953926	0.038152209
Cer 22:1	IL-6	6.008237299	6.818181792	0.385210885	0.019694233	0.003387119	2.3445E-06
Cer 22:1	MCP1	4.523124426	6.253939881	0.475131631	0.003643642	0.000575039	5.46735E-07
Cer 22:1	TNF-α	10.45725116	7.952714277	0.198497222	0.113630102	0.024753754	7.38671E-05
Cer 24:0	IL-17A	0.002426577	0.265240559	0.992761153	0.00193184	0.00413344	0.643613386
Cer 24:0	IL-1β	0.046638572	0.213527181	0.828581056	0.071971154	0.032713413	0.035644525
Cer 24:0	IL-6	0.031736054	0.167192261	0.850730499	0.019053942	0.003678246	1.40056E-05
Cer 24:0	MCP1	0.105311166	0.142166444	0.464598069	0.003708316	0.0005789	4.51114E-07
Cer 24:0	TNF-α	0.183218835	0.17915435	0.314632302	0.116096375	0.024695385	5.41093E-05
Cer 24:1	IL-17A	0.007711285	0.59772489	0.989792143	0.001926587	0.004150725	0.645886248
Cer 24:1	IL-1β	0.26366277	0.470057876	0.579020942	0.07304141	0.032090388	0.030143537
Cer 24:1	IL-6	0.326175741	0.33831103	0.342688749	0.019389052	0.003316589	2.14543E-06
Cer 24:1	MCP1	0.070967805	0.330512539	0.83143821	0.00365083	0.000599716	1.09173E-06
Cer 24:1	TNF-α	0.290764569	0.427216939	0.501343072	0.11013907	0.026241451	0.000221538
Cer 26:0	IL-17A	1.279790204	14.26470421	0.929108207	0.00190293	0.004058182	0.642522422
Cer 26:0	IL-1β	6.604515786	11.20032749	0.559826054	0.069102432	0.031325673	0.03518875
Cer 26:0	IL-6	11.14429748	8.81368126	0.215810676	0.020176019	0.003539802	3.23567E-06
Cer 26:0	MCP1	12.15051142	7.527182701	0.116950836	0.00369721	0.000559546	2.58731E-07
Cer 26:0	TNF-α	19.43720794	10.47392338	0.073334311	0.123800461	0.02635693	5.47314E-05
Cer 26:1	IL-1β	-17.30325014	14.96883989	0.256824274	0.089155043	0.036713307	0.021371813
Cer 26:1	IL-6	0.400522207	11.32469167	0.972021252	0.018930605	0.003988537	4.76553E-05
Cer 26:1	MCP1	4.680998592	9.408813323	0.622455072	0.003698652	0.000613344	1.28107E-06
Cer 26:1	TNF-α	2.233604623	13.26564063	0.867418185	0.109474716	0.029273816	0.000776717
DG 12:0 16:0	IL-17A	0.12927442	7.004289982	0.985410425	0.001916761	0.004615188	0.681192699
DG 12:0 16:0	IL-1β	-0.238932421	5.249895902	0.964034287	0.088973754	0.035349598	0.018080116
DG 12:0 16:0	IL-6	2.087684049	4.117997544	0.616294454	0.018248151	0.00391663	7.61243E-05
DG 12:0 16:0	MCP1	3.165632475	3.054964971	0.309289	0.003759263	0.000537249	1.60357E-07
DG 12:0 16:0	TNF-α	2.176404146	5.204992635	0.679153409	0.101316937	0.031235967	0.003136699
DG 12:0 18:0	IL-17A	-0.299752434	5.362010905	0.955947562	0.001981568	0.002817248	0.489550684
DG 12:0 18:0	IL-1β	-7.002586072	6.083716652	0.262654606	0.083456869	0.0375468	0.037347133
DG 12:0 18:0	IL-6	-0.182189759	4.990223024	0.971221061	0.018313236	0.004009495	0.000167383
DG 12:0 18:0	MCP1	1.379566232	3.487679935	0.696423687	0.003731687	0.000553529	1.14004E-06
DG 12:0 18:0	TNF-α	2.463078235	4.576388042	0.596088088	0.12176436	0.02302448	3.04273E-05
DG 12:0 18:1	IL-17A	-0.013021884	1.754655835	0.99412782	0.001948488	0.004135632	0.64094442
DG 12:0 18:1	IL-1β	-0.89894926	1.420001652	0.531489641	0.073445153	0.032903327	0.033221686
DG 12:0 18:1	IL-6	-0.053082611	1.155521497	0.963663922	0.018946567	0.003844863	2.85664E-05
DG 12:0 18:1	MCP1	0.340651198	0.93503189	0.718174181	0.003672448	0.000575853	4.87958E-07
DG 12:0 18:1	TNF-α	0.027550564	1.340395591	0.983737492	0.109032668	0.027944716	0.000499884
DG 12:0 18:2	IL-17A	0.094258343	3.289325098	0.977328868	0.001921919	0.004125914	0.644711157
DG 12:0 18:2	IL-1β	1.193453835	2.609879407	0.65076195	0.065906729	0.032183611	0.049417793
DG 12:0 18:2	IL-6	1.694342169	1.996785015	0.402860069	0.019131604	0.00353588	7.30495E-06
DG 12:0 18:2	MCP1	1.806517237	1.756042078	0.311823475	0.003762229	0.00057555	3.14311E-07
DG 12:0 18:2	TNF-α	2.096191531	2.435824822	0.396302023	0.116743373	0.027025649	0.000157551
DG 14:0 14:0	IL-17A	-0.094507748	11.28798301	0.993375281	0.001944336	0.004094	0.63828093
DG 14:0 14:0	IL-1β	-6.011095483	9.498601346	0.531632692	0.079090766	0.033868199	0.026409431
DG 14:0 14:0	IL-6	-2.680827331	7.061334129	0.706880426	0.019020863	0.003615523	1.11505E-05
DG 14:0 14:0	MCP1	2.016567117	6.320879855	0.751912577	0.003670846	0.000599024	9.75299E-07
DG 14:0 14:0	TNF-α	3.64444069	8.211031664	0.660338273	0.109236408	0.026341829	0.000254643
DG 14:0 16:0	IL-17A	-0.012768551	1.715141432	0.994109405	0.001948316	0.004130308	0.64054519
DG 14:0 16:0	IL-1β	0.000643887	1.41430709	0.999639763	0.072467301	0.03348322	0.038529306

DG 14:0 16:0	IL-6	0.026992466	1.129008324	0.981084219	0.018934822	0.003838244	2.81313E-05
DG 14:0 16:0	MCP1	0.316156798	0.932515613	0.736944066	0.003700092	0.000586778	5.95055E-07
DG 14:0 16:0	TNF-α	-0.037712426	1.286645658	0.976810955	0.108968468	0.027406792	0.000407902
DG 14:0 16:1	IL-17A	-0.011242442	4.243574408	0.997912426	0.001946407	0.003727689	0.60730242
DG 14:0 16:1	IL-1β	-2.406871624	4.44532633	0.594185994	0.079717629	0.044790845	0.090313425
DG 14:0 16:1	IL-6	0.315650233	3.630236898	0.93157567	0.017925443	0.005156793	0.00238346
DG 14:0 16:1	MCP1	1.415316787	2.275681307	0.541012924	0.003903868	0.000621222	3.9017E-06
DG 14:0 16:1	TNF-α	0.599512798	3.52008655	0.866475899	0.107358824	0.031352481	0.002686731
DG 14:0 18:1	IL-17A	0.00592504	0.801623189	0.99415158	0.001933935	0.004107526	0.641169825
DG 14:0 18:1	IL-1β	0.35263546	0.59936809	0.560704172	0.078181416	0.030192891	0.014691062
DG 14:0 18:1	IL-6	0.281214446	0.485506446	0.566765829	0.018989573	0.003512028	7.3815E-06
DG 14:0 18:1	MCP1	0.138914548	0.442844755	0.755929746	0.003661048	0.000592921	8.56712E-07
DG 14:0 18:1	TNF-α	0.031601253	0.606926711	0.958819944	0.108496229	0.027508257	0.000445112
DG 14:0 18:2	IL-17A	-0.009924123	1.486263753	0.994716587	0.001946097	0.004100145	0.638479875
DG 14:0 18:2	IL-1β	0.123691763	1.222842996	0.920103632	0.074655903	0.033164616	0.031863858
DG 14:0 18:2	IL-6	0.178787822	0.933624737	0.849425217	0.019045267	0.003636039	1.18979E-05
DG 14:0 18:2	MCP1	0.000224244	0.83720005	0.99978806	0.003708494	0.000603486	9.30009E-07
DG 14:0 18:2	TNF-α	-2.81039E-10	1.125437802	1	0.109289544	0.027462592	0.000403877
DG 14:0 20:0	IL-17A	-0.023292733	5.692751138	0.996763364	0.001947084	0.003983324	0.628653344
DG 14:0 20:0	IL-1β	-3.890717475	4.899594149	0.433593493	0.090237794	0.033902321	0.012544522
DG 14:0 20:0	IL-6	-3.041653086	3.16475764	0.344446796	0.01895942	0.003228986	2.26648E-06
DG 14:0 20:0	MCP1	-0.090320622	3.488841634	0.979523658	0.003646627	0.000665933	6.76271E-06
DG 14:0 20:0	TNF-α	1.240855274	4.168265931	0.768061263	0.104422765	0.026137339	0.00040536
DG 14:1 16:0	IL-1β	-0.986649706	1.345694134	0.469133377	0.084065595	0.034336324	0.020417276
DG 14:1 16:0	IL-6	-0.325095582	1.035266662	0.755678315	0.019204556	0.003793251	1.95096E-05
DG 14:1 16:0	MCP1	0.022999569	0.886389422	0.979471066	0.003719803	0.000601127	8.25237E-07
DG 14:1 16:0	TNF-α	2.91273E-05	1.195350416	0.999980719	0.109289201	0.027442168	0.00040062
DG 14:1 18:1	IL-17A	-0.017774287	1.632464595	0.991384883	0.001947914	0.004093426	0.637618394
DG 14:1 18:1	IL-1β	-1.369358409	1.3837552	0.330286618	0.088202292	0.034111707	0.014820305
DG 14:1 18:1	IL-6	-0.676723878	0.974599352	0.492798406	0.019029651	0.003450025	5.43118E-06
DG 14:1 18:1	MCP1	-0.40051835	0.938062672	0.672454757	0.003692872	0.000614625	1.36216E-06
DG 14:1 18:1	TNF-α	0.013645101	1.251503124	0.991373045	0.109310265	0.027758236	0.000452728
DG 14:1 20:0	IL-17A	0.001420795	0.374730901	0.996999922	0.001939245	0.00408714	0.638597054
DG 14:1 20:0	IL-1β	-0.111696827	0.298706947	0.711082924	0.075424863	0.032029215	0.025266201
DG 14:1 20:0	IL-6	-0.264480972	0.230553347	0.260383387	0.02024347	0.003549967	3.21139E-06
DG 14:1 20:0	MCP1	-0.187407648	0.228601444	0.418795581	0.003866873	0.000651499	1.67068E-06
DG 14:1 20:0	TNF-α	9.95578E-11	0.297805932	1	0.10928577	0.028730955	0.000652849
DG 15:0 18:1	IL-17A	-0.017954337	3.829734576	0.996290455	0.00194424	0.004098007	0.638624769
DG 15:0 18:1	IL-1β	0.348023084	3.113990748	0.911757251	0.07377868	0.032758374	0.031783856
DG 15:0 18:1	IL-6	1.251927551	2.336163243	0.595985506	0.018958109	0.003529069	8.14835E-06
DG 15:0 18:1	MCP1	0.715067465	2.112400576	0.737337144	0.003669379	0.00059063	7.70655E-07
DG 15:0 18:1	TNF-α	0.661529865	2.801374436	0.814925198	0.108733615	0.026515018	0.000289221
DG 16:0 16:0	IL-17A	0.007383732	0.554210091	0.98945832	0.001924199	0.004331679	0.660074713
DG 16:0 16:0	IL-1β	0.517544452	0.438387154	0.247051577	0.096057918	0.033685319	0.007799274
DG 16:0 16:0	IL-6	0.241458849	0.311118406	0.443766128	0.018645529	0.003432894	6.89004E-06
DG 16:0 16:0	MCP1	0.196336154	0.303590526	0.522734807	0.003637309	0.000620019	2.02635E-06
DG 16:0 16:0	TNF-α	0.282920609	0.380379098	0.462792069	0.106268791	0.026297547	0.000341099
DG 16:0 16:1	IL-17A	0.016952446	0.936123878	0.985671652	0.00192859	0.004235631	0.652153615
DG 16:0 16:1	IL-1β	0.539618336	0.735257492	0.468696479	0.078832978	0.032705834	0.02227322
DG 16:0 16:1	IL-6	0.538377224	0.491108468	0.281692196	0.019306439	0.003137004	9.06227E-07

DG 16:0 16:1	MCP1	0.204667941	0.504666936	0.687950793	0.003621312	0.000596657	1.14879E-06
DG 16:0 16:1	TNF-α	0.335745483	0.653651716	0.611262226	0.110229625	0.026160614	0.000211665
DG 16:0 18:0	IL-17A	-0.007165356	0.741663921	0.992355567	0.001955765	0.004311268	0.653352424
DG 16:0 18:0	IL-1β	0.120468676	0.59871028	0.841889593	0.07593026	0.03421492	0.034179622
DG 16:0 18:0	IL-6	-0.166217987	0.459159004	0.719885638	0.018953293	0.003768025	2.14025E-05
DG 16:0 18:0	MCP1	-0.070804007	0.409491449	0.863885708	0.003690327	0.000621982	1.68086E-06
DG 16:0 18:0	TNF-α	0.08303025	0.543960195	0.879703947	0.108944777	0.027969306	0.000508944
DG 16:0 18:1	IL-17A	0.004968102	0.108915599	0.96392001	0.001894164	0.004189683	0.65444756
DG 16:0 18:1	IL-1β	0.095880469	0.07699332	0.22265762	0.087835219	0.029116898	0.00516877
DG 16:0 18:1	IL-6	0.068891217	0.057801668	0.242661423	0.019205868	0.00313895	1.00142E-06
DG 16:0 18:1	MCP1	0.029638214	0.059818583	0.623879596	0.00364799	0.00060126	1.15553E-06
DG 16:0 18:1	TNF-α	0.06151908	0.079285286	0.443870221	0.107355287	0.02697742	0.00040402
DG 16:0 18:2	IL-17A	0.005684616	0.242010878	0.981415685	0.001922425	0.004112201	0.643525274
DG 16:0 18:2	IL-1β	0.190231977	0.18113073	0.301991763	0.086732765	0.030257467	0.007518426
DG 16:0 18:2	IL-6	0.137754501	0.131134866	0.301886542	0.019419581	0.003145651	8.59416E-07
DG 16:0 18:2	MCP1	0.027371562	0.138472385	0.844638085	0.003643381	0.000614805	1.71461E-06
DG 16:0 18:2	TNF-α	0.085905663	0.179933099	0.636515247	0.10953588	0.02704377	0.000332469
DG 16:0 18:3	IL-1β	-7.02597E-09	1.889215383	0.999999997	0.072463768	0.033334611	0.037739844
DG 16:0 18:3	IL-6	0.233910674	1.452999291	0.873184655	0.019098032	0.003681554	1.3719E-05
DG 16:0 18:3	MCP1	-0.508732927	1.334111253	0.70564788	0.003734111	0.000625662	1.5237E-06
DG 16:0 18:3	TNF-α	-0.276370668	1.736748349	0.874632078	0.108339389	0.027571892	0.000463514
DG 16:0 20:0	IL-1β	-0.643288123	1.296733266	0.623450588	0.071156825	0.036590988	0.061244183
DG 16:0 20:0	IL-6	-0.127457459	0.965845491	0.895893352	0.018816377	0.00391366	4.006E-05
DG 16:0 20:0	MCP1	-0.042598177	0.811248103	0.958470879	0.003715502	0.000608431	1.03511E-06
DG 16:0 20:0	TNF-α	-0.101941473	1.121642586	0.928187408	0.107916279	0.028476966	0.00067845
DG 16:0 20:3	IL-17A	0.054382389	5.256269624	0.991821148	0.001938799	0.004371009	0.660895814
DG 16:0 20:3	IL-1β	2.187111784	3.851706155	0.574842861	0.07225977	0.032135798	0.032903945
DG 16:0 20:3	IL-6	1.83595464	2.945768639	0.538348942	0.019601514	0.003631066	1.04649E-05
DG 16:0 20:3	MCP1	0.328570538	2.799358435	0.907432854	0.003814959	0.00064516	2.66023E-06
DG 16:0 20:3	TNF-α	0.088867296	3.545283055	0.980186389	0.114338067	0.026787121	0.000216852
DG 16:0 20:4	IL-17A	-0.815089369	1.077548152	0.455941623	0.002122877	0.003670686	0.567832809
DG 16:0 20:4	IL-1β	-0.709321638	1.099225331	0.524183935	0.040678542	0.043045961	0.353038218
DG 16:0 20:4	IL-6	-0.021889827	0.784474359	0.977944096	0.019137679	0.003925358	4.25713E-05
DG 16:0 20:4	MCP1	-0.25634447	0.644855025	0.6941075	0.003787633	0.000594075	7.91103E-07
DG 16:0 20:4	TNF-α	-0.290105869	0.730998008	0.694588313	0.113455205	0.023038032	3.7288E-05
DG 16:0 20:5	IL-17A	-9.914585572	6.55780848	0.148932023	-0.100261405	0.038860761	0.019467498
DG 16:0 20:5	IL-1β	-6.761098398	7.114395967	0.35525568	0.057591853	0.055853849	0.316933465
DG 16:0 20:5	IL-6	-0.855628144	5.990452806	0.888102153	0.016827864	0.006307087	0.016218846
DG 16:0 20:5	MCP1	-1.237044225	3.818389584	0.749912455	0.003785524	0.000757714	0.000110504
DG 16:0 20:5	TNF-α	-2.231171986	7.417467385	0.767214992	0.088622022	0.053390126	0.115267542
DG 16:0 22:5	IL-17A	0.012348293	4.674524218	0.997910387	0.001935316	0.004231156	0.650794342
DG 16:0 22:5	IL-1β	0.893136732	3.590276748	0.805295593	0.074447896	0.032310192	0.028571729
DG 16:0 22:5	IL-6	-3.09134E-05	3.003086623	0.999991857	0.018987289	0.003889462	3.5238E-05
DG 16:0 22:5	MCP1	-0.215517796	2.650039969	0.9357412	0.003736995	0.000631418	1.99282E-06
DG 16:0 22:5	TNF-α	2.41755132	3.285991696	0.467813762	0.109408708	0.026397982	0.000269803
DG 16:0 22:6	IL-17A	-9.42866E-15	3.502075132	1	6.02011E-17	0.004404278	1
DG 16:0 22:6	IL-1β	-2.471411222	3.010811419	0.419197112	0.089773268	0.038707865	0.028502514
DG 16:0 22:6	IL-6	0.990037856	2.331712325	0.674623592	0.020091021	0.004345562	9.0848E-05
DG 16:0 22:6	MCP1	-0.226446126	1.723002942	0.896450609	0.004058196	0.000618353	5.85643E-07
DG 16:0 22:6	TNF-α	-0.183653144	2.767115452	0.947591071	0.115071517	0.033354654	0.00192581

DG 16:1 16:1	IL-1β	-2.249626968	4.03968577	0.581880958	0.052726177	0.035377006	0.14691438
DG 16:1 16:1	IL-6	0.591704332	3.018638213	0.845963979	0.019121456	0.003513716	7.43164E-06
DG 16:1 16:1	MCP1	0.516313225	2.627649398	0.845595288	0.003711347	0.000567651	3.6817E-07
DG 16:1 16:1	TNF-α	1.085300829	3.303860177	0.744897887	0.119442633	0.024963515	4.6138E-05
DG 16:1 18:0	IL-17A	-0.014075512	3.198711641	0.996518156	0.001946927	0.004184342	0.645084806
DG 16:1 18:0	IL-1β	0.410360117	2.585375989	0.874950357	0.074159866	0.033248861	0.033346299
DG 16:1 18:0	IL-6	-0.266537021	2.105224933	0.900095918	0.018727208	0.003887796	3.90512E-05
DG 16:1 18:0	MCP1	-0.079755643	1.77775724	0.964513754	0.003721811	0.000607658	9.84058E-07
DG 16:1 18:0	TNF-α	-0.375826226	2.398375165	0.876530829	0.107005396	0.027751466	0.000566576
DG 16:1 18:1	IL-17A	0.008038076	0.468906872	0.986436707	0.001936452	0.004152975	0.644382701
DG 16:1 18:1	IL-1β	0.30015888	0.332292487	0.373564519	0.081734705	0.02893305	0.008327933
DG 16:1 18:1	IL-6	0.286302372	0.245156729	0.252071607	0.019360222	0.003065275	5.78462E-07
DG 16:1 18:1	MCP1	-0.012493964	0.275470532	0.964124919	0.003726968	0.000637504	2.14477E-06
DG 16:1 18:1	TNF-α	0.117483643	0.33374764	0.727288995	0.106133947	0.026146144	0.000324377
DG 16:1 18:2	IL-17A	0.003975296	0.92662683	0.99660543	0.001937609	0.004100253	0.6399482
DG 16:1 18:2	IL-1β	0.320183763	0.717187523	0.658482914	0.079142322	0.031198917	0.016629289
DG 16:1 18:2	IL-6	0.380211785	0.519291831	0.469744395	0.019188922	0.00324392	1.76689E-06
DG 16:1 18:2	MCP1	-0.072302575	0.547079287	0.895739336	0.003720077	0.000632544	1.94464E-06
DG 16:1 18:2	TNF-α	3.88903E-10	0.701464482	1	0.1092896	0.027455428	0.000402725
DG 16:1 18:3	IL-17A	-5.80018E-15	8.277465618	1	3.2752E-17	0.005164843	1
DG 16:1 18:3	IL-1β	1.18488E-05	5.447171905	0.999998282	0.079999998	0.035107463	0.031872473
DG 16:1 18:3	IL-6	-0.413440271	5.905179338	0.944763302	0.018542567	0.006303114	0.007122851
DG 16:1 18:3	MCP1	-1.103192414	4.124604218	0.791395308	0.003760407	0.000766834	5.30535E-05
DG 16:1 18:3	TNF-α	0.587400501	6.32553097	0.926784157	0.088454973	0.039359319	0.034078784
DG 16:1 20:0	IL-17A	-0.039480827	4.138277048	0.992460804	0.001949847	0.00403097	0.632636124
DG 16:1 20:0	IL-1β	-3.951311564	3.563525562	0.277659066	0.095451323	0.037293817	0.016647687
DG 16:1 20:0	IL-6	-1.971060462	2.523898179	0.441879865	0.018847859	0.00365848	2.25208E-05
DG 16:1 20:0	MCP1	-1.535487637	2.545271104	0.551555491	0.003656891	0.00071325	2.40375E-05
DG 16:1 20:0	TNF-α	0.566228194	3.556809144	0.874745008	0.101971743	0.031899856	0.003633047
DG 16:1 20:2	IL-17A	1.41006056	1.593605324	0.383786966	0.001766664	0.00444268	0.693898388
DG 16:1 20:2	IL-1β	1.670227361	1.194466456	0.173000037	0.070379477	0.033154261	0.042747635
DG 16:1 20:2	IL-6	0.557213723	1.00990487	0.5854976	0.018757383	0.004176014	0.000111141
DG 16:1 20:2	MCP1	0.156206356	0.8898241	0.861912872	0.003737689	0.000675474	6.4683E-06
DG 16:1 20:2	TNF-α	0.104408267	1.260524711	0.934576657	0.10522313	0.031718088	0.002525193
DG 16:1 20:4	IL-17A	-4.763206047	3.70930164	0.210871807	0.001691265	0.004361954	0.701496775
DG 16:1 20:4	IL-1β	-4.776160036	3.261614595	0.155559725	0.06347169	0.038529275	0.111997266
DG 16:1 20:4	IL-6	-1.09810599	2.836726687	0.701955575	0.018593518	0.00517221	0.001390623
DG 16:1 20:4	MCP1	-2.688011291	2.252627752	0.243962165	0.003777062	0.000794023	6.99666E-05
DG 16:1 20:4	TNF-α	-2.826035001	3.192997485	0.384551264	0.097683403	0.036036743	0.011959208
DG 16:1 22:6	IL-17A	-0.684633128	5.34504641	0.899147065	0.002050163	0.004533849	0.655192446
DG 16:1 22:6	IL-1β	-5.768452344	3.461196125	0.108592186	0.104611459	0.029829524	0.001810834
DG 16:1 22:6	IL-6	-1.056311404	3.264223114	0.749041851	0.017884879	0.004463262	0.000517497
DG 16:1 22:6	MCP1	-1.843774856	2.918680031	0.533544499	0.003832787	0.000783187	5.44137E-05
DG 16:1 22:6	TNF-α	-1.539757668	4.226574422	0.718821184	0.097576272	0.036691147	0.013723344
DG 18:0 18:1	IL-17A	0.038177672	0.632589666	0.952276024	0.001849847	0.004286119	0.669125144
DG 18:0 18:1	IL-1β	0.462976405	0.458265576	0.320440761	0.081625283	0.030525329	0.012011224
DG 18:0 18:1	IL-6	0.314720371	0.353584996	0.380503329	0.019040102	0.003382119	3.94128E-06
DG 18:0 18:1	MCP1	0.229958738	0.332444492	0.494431724	0.003654162	0.000588567	7.79462E-07
DG 18:0 18:1	TNF-α	0.318045259	0.449530489	0.484712864	0.108259577	0.026941267	0.000363052
DG 18:0 18:2	IL-17A	0.019346723	1.114096294	0.986260095	0.001921398	0.004141269	0.646023064

DG 18:0 18:2	IL-1β	0.659701791	0.842148539	0.43955918	0.080726553	0.030775168	0.013563211
DG 18:0 18:2	IL-6	0.522980435	0.67009818	0.441238914	0.019138258	0.003516431	6.67778E-06
DG 18:0 18:2	MCP1	0.247610252	0.611681129	0.688494431	0.003666653	0.000594115	8.63856E-07
DG 18:0 18:2	TNF-α	0.331823087	0.792251148	0.678319803	0.109622534	0.026048958	0.000214769
DG 18:0 18:3	IL-17A	-0.003952918	6.405264855	0.999512134	0.001946023	0.004370535	0.659681691
DG 18:0 18:3	IL-1β	-0.961256144	5.181146237	0.854200389	0.068748189	0.03534143	0.062225562
DG 18:0 18:3	IL-6	2.03722413	3.769993989	0.593365986	0.019533521	0.003809439	2.16144E-05
DG 18:0 18:3	MCP1	1.132823434	3.297749944	0.733872391	0.003831203	0.000623629	1.45116E-06
DG 18:0 18:3	TNF-α	1.162849108	4.405097394	0.793801275	0.1102327	0.027581461	0.00044654
DG 18:0 20:0	IL-17A	-0.149852062	9.855253561	0.988011931	0.001972517	0.003351809	0.56247804
DG 18:0 20:0	IL-1β	-12.49626435	10.42472471	0.243991818	0.124136746	0.041890711	0.007416869
DG 18:0 20:0	IL-6	-5.186808904	6.177913708	0.410604437	0.017626856	0.003295881	2.64688E-05
DG 18:0 20:0	MCP1	-1.529223598	7.602180365	0.842512261	0.003523783	0.000820199	0.000319945
DG 18:0 20:0	TNF-α	3.213706029	7.993168858	0.691705206	0.103798068	0.025936826	0.000646684
DG 18:0 22:6	IL-17A	0.000325967	0.496552287	0.999480715	0.001945631	0.003987156	0.629237053
DG 18:0 22:6	IL-1β	-0.245971591	0.397284968	0.540663525	0.085496926	0.031546147	0.011173498
DG 18:0 22:6	IL-6	-0.324041154	0.293998741	0.279444534	0.019402392	0.003442279	4.33603E-06
DG 18:0 22:6	MCP1	-0.335008533	0.346556044	0.341697452	0.003843593	0.000759098	2.12692E-05
DG 18:0 22:6	TNF-α	-4.8735E-10	0.402627654	0.999999999	0.105263158	0.028972397	0.001072083
DG 18:1 18:1	IL-17A	0.004169539	0.107318351	0.969265741	0.001916971	0.004126103	0.645575226
DG 18:1 18:1	IL-1β	0.082781483	0.07564501	0.282514137	0.08231386	0.028592188	0.007291438
DG 18:1 18:1	IL-6	0.067482369	0.056444542	0.241236655	0.019422005	0.003063663	5.42005E-07
DG 18:1 18:1	MCP1	0.022832472	0.060148757	0.706916262	0.003633615	0.000604265	1.34349E-06
DG 18:1 18:1	TNF-α	0.060046339	0.080123277	0.459438122	0.107653592	0.027248435	0.000437032
DG 18:1 18:2	IL-17A	0.00167228	0.109400367	0.987905322	0.001931591	0.004089718	0.640127535
DG 18:1 18:2	IL-1β	0.064371692	0.082113971	0.439226585	0.074849366	0.030178153	0.018965177
DG 18:1 18:2	IL-6	0.058569144	0.060760913	0.342788264	0.019502831	0.003206652	1.10894E-06
DG 18:1 18:2	MCP1	0.009342293	0.061162122	0.879621016	0.003653185	0.000597437	1.01209E-06
DG 18:1 18:2	TNF-α	0.026910907	0.079745293	0.73811943	0.109444999	0.026369167	0.000252111
DG 18:1 20:0	IL-17A	-0.188268225	4.574714337	0.967475872	0.00195382	0.004380196	0.659112367
DG 18:1 20:0	IL-1β	-3.813189777	3.605530666	0.299611268	0.088237495	0.03449336	0.016451646
DG 18:1 20:0	IL-6	0.548350132	3.097280157	0.860796448	0.018587812	0.004548007	0.000351428
DG 18:1 20:0	MCP1	0.879462778	2.66471583	0.743916884	0.003760833	0.00073812	2.3586E-05
DG 18:1 20:0	TNF-α	-0.526358262	3.336975031	0.875839246	0.09946144	0.02936534	0.002181279
DG 18:1 20:1	IL-1β	-0.891674268	4.580947838	0.846980383	0.071015038	0.035101261	0.052047972
DG 18:1 20:1	IL-6	0.250153817	3.44250984	0.942554135	0.018947334	0.003787865	2.3158E-05
DG 18:1 20:1	MCP1	0.850241026	2.95374868	0.775439671	0.003688749	0.000601554	9.64543E-07
DG 18:1 20:1	TNF-α	1.430922041	3.864151966	0.71375557	0.112460666	0.02664019	0.000207076
DG 18:1 20:2	IL-17A	-8.28716E-16	4.929141043	1	3.32575E-17	0.004289538	1
DG 18:1 20:2	IL-1β	-0.841550821	4.082986052	0.838096127	0.068897995	0.03493157	0.057846855
DG 18:1 20:2	IL-6	0.376476524	3.118889339	0.904726748	0.019148073	0.003831705	2.34768E-05
DG 18:1 20:2	MCP1	0.001032966	2.660385976	0.999692771	0.003708463	0.000604949	9.69452E-07
DG 18:1 20:2	TNF-α	-0.130572677	3.617455528	0.971445584	0.108486146	0.027845765	0.000507818
DG 18:1 20:3	IL-17A	0.026560997	1.721568845	0.987792583	0.001938194	0.00408941	0.638963709
DG 18:1 20:3	IL-1β	0.876659836	1.291109184	0.502344656	0.075545415	0.030150893	0.017882131
DG 18:1 20:3	IL-6	0.859299147	0.978383768	0.386769745	0.019650639	0.00328094	1.43649E-06
DG 18:1 20:3	MCP1	0.305759779	0.959914619	0.752291431	0.003619759	0.000595804	1.12948E-06
DG 18:1 20:3	TNF-α	1.066360162	1.203569068	0.382665677	0.112728387	0.025288552	0.00010717
DG 18:1 20:4	IL-17A	-0.018573349	0.370008993	0.960298114	0.001963713	0.00407833	0.633655517
DG 18:1 20:4	IL-1β	-0.235845911	0.316675095	0.462213564	0.066237448	0.034315078	0.063074539

DG 18:1 20:4	IL-6	-0.017460599	0.252236299	0.945271329	0.018763681	0.003924918	4.32536E-05
DG 18:1 20:4	MCP1	-0.111156893	0.21724555	0.612631552	0.003608071	0.000625685	2.68163E-06
DG 18:1 20:4	TNF-α	-0.086766261	0.277961229	0.75708406	0.106241168	0.02710011	0.000475099
DG 18:1 20:5	IL-17A	-0.027129905	2.123453033	0.989900193	0.001942508	0.004308288	0.655678408
DG 18:1 20:5	IL-1β	-1.319462583	1.545385185	0.400723125	0.049782669	0.034183844	0.156835078
DG 18:1 20:5	IL-6	0.831556787	1.29332774	0.525673839	0.019780308	0.003921468	2.7052E-05
DG 18:1 20:5	MCP1	0.047406454	1.176163302	0.96814573	0.003794431	0.000658927	4.00704E-06
DG 18:1 20:5	TNF-α	-0.211180813	1.487636802	0.888167752	0.109115843	0.027236282	0.000435291
DG 18:1 22:4	IL-17A	-0.040008551	8.490755231	0.996281003	0.001946855	0.005224894	0.712849358
DG 18:1 22:4	IL-1β	3.169659306	6.019163221	0.603517081	0.094231494	0.039755478	0.026539
DG 18:1 22:4	IL-6	3.395871665	4.572927147	0.465239183	0.020637458	0.004858991	0.000304025
DG 18:1 22:4	MCP1	2.366087392	3.837734679	0.543598105	0.003723761	0.000698789	2.07074E-05
DG 18:1 22:4	TNF-α	1.155957074	5.333744891	0.830333329	0.110902651	0.033230894	0.002860819
DG 18:1 22:5	IL-17A	0.081487053	2.159318873	0.970147072	0.001934968	0.004078587	0.638634733
DG 18:1 22:5	IL-1β	1.528662235	1.582869469	0.341888611	0.078173295	0.029392669	0.012432534
DG 18:1 22:5	IL-6	1.363037049	1.170107148	0.253246162	0.019783332	0.003120121	5.40366E-07
DG 18:1 22:5	MCP1	0.701113325	1.189271671	0.559918707	0.003701242	0.000586961	5.95063E-07
DG 18:1 22:5	TNF-α	1.911855284	1.495810631	0.211001249	0.114009689	0.024991153	8.00012E-05
DG 18:1 22:6	IL-17A	-0.053510813	1.144282584	0.963011533	0.001961757	0.004069989	0.633300049
DG 18:1 22:6	IL-1β	-0.969932581	1.005541506	0.342463204	0.079350533	0.035160974	0.031464186
DG 18:1 22:6	IL-6	0.115411614	0.785250104	0.884135554	0.019228529	0.003942946	3.2994E-05
DG 18:1 22:6	MCP1	-0.342863055	0.686477609	0.621103887	0.003654056	0.000638001	2.99417E-06
DG 18:1 22:6	TNF-α	-0.28681533	0.887363842	0.748769568	0.104682665	0.027917619	0.000755943
DG 18:2 18:3	IL-1β	-1.109054289	1.623920188	0.499879713	0.080724993	0.037333686	0.038699219
DG 18:2 18:3	IL-6	0.151906626	1.175584908	0.898047684	0.019100977	0.003880986	2.90607E-05
DG 18:2 18:3	MCP1	0.182514768	1.0088127	0.857646817	0.003687048	0.000616426	1.46896E-06
DG 18:2 18:3	TNF-α	0.070757863	1.415342969	0.960458993	0.109873324	0.029276127	0.000749216
DG 18:2 20:0	IL-17A	-5.667138187	4.077297995	0.182485504	-0.088045139	0.036344346	0.026871977
DG 18:2 20:0	IL-1β	-4.963259087	4.921689313	0.327379361	0.13676715	0.056682009	0.027402358
DG 18:2 20:0	IL-6	-0.899068717	3.149283453	0.778722839	0.015162418	0.006892501	0.041937625
DG 18:2 20:0	MCP1	-0.862717964	4.157578486	0.838080955	0.002714157	0.002084626	0.210286701
DG 18:2 20:3	IL-17A	-0.007753835	1.810979295	0.996612157	0.001941491	0.004111712	0.640211064
DG 18:2 20:3	IL-1β	-0.552175342	1.585959288	0.7301477	0.073362883	0.035399912	0.046917323
DG 18:2 20:3	IL-6	0.14697776	1.190869292	0.902597259	0.018987698	0.003817035	2.50384E-05
DG 18:2 20:3	MCP1	0.472640216	1.018058728	0.645815632	0.003757388	0.000603972	7.52718E-07
DG 18:2 20:3	TNF-α	1.308111905	1.330637013	0.333434369	0.12029459	0.026723017	9.47851E-05
DG 18:2 20:4	IL-17A	-0.80420245	0.584493738	0.179760541	0.002163692	0.003630188	0.555944984
DG 18:2 20:4	IL-1β	-0.484432172	0.620479308	0.441507307	0.063819141	0.03982416	0.120262828
DG 18:2 20:4	IL-6	-7.35041E-07	0.48183353	0.999998794	0.019138751	0.004380437	0.000155099
DG 18:2 20:4	MCP1	-0.191177932	0.390148806	0.627942451	0.00365303	0.000653995	5.61128E-06
DG 18:2 20:4	TNF-α	-0.176321481	0.450674728	0.698582103	0.111234001	0.025894452	0.000189311
DG 18:2 20:5	IL-17A	-0.05374469	2.354151207	0.981953964	0.001940978	0.004354422	0.65933279
DG 18:2 20:5	IL-1β	-2.286801527	1.97320687	0.256633798	0.083263025	0.036522666	0.030741373
DG 18:2 20:5	IL-6	-0.023923133	1.837695731	0.989709145	0.019641804	0.005700386	0.001877716
DG 18:2 20:5	MCP1	-0.46839226	1.447443656	0.748734391	0.00370252	0.000773548	5.40753E-05
DG 18:2 20:5	TNF-α	-0.131905608	1.996811311	0.947818263	0.098507359	0.036097363	0.011041124
DG 18:2 22:4	IL-17A	-4.771446717	3.01933434	0.126609381	0.002049613	0.004194996	0.629393857
DG 18:2 22:4	IL-1β	-5.262668488	3.139167627	0.106112187	0.082035086	0.045978741	0.086535233
DG 18:2 22:4	IL-6	-0.70676468	2.195576268	0.750201701	0.019381591	0.005088224	0.000807592
DG 18:2 22:4	MCP1	0.516394371	2.042049588	0.802428393	0.003646446	0.000814323	0.000144225

DG 18:2 22:4	TNF-α	-0.373953045	2.92742683	0.899375773	0.103636377	0.040784461	0.017633636
DG 18:2 22:5	IL-17A	-0.00240759	2.341941981	0.999186789	0.001945793	0.004014552	0.6315407
DG 18:2 22:5	IL-1β	-0.33330526	2.08730035	0.87423822	0.067927457	0.035520986	0.065758938
DG 18:2 22:5	IL-6	0.17094709	1.699618976	0.920576295	0.019532144	0.004504092	0.000159369
DG 18:2 22:5	MCP1	0.929260354	1.292501064	0.477917474	0.003652141	0.000596149	1.12723E-06
DG 18:2 22:5	TNF-α	0.756744372	1.839981843	0.683892216	0.10617047	0.02971987	0.001259663
DG 18:2 22:6	IL-17A	-2.262519727	1.537892781	0.15201082	0.002278588	0.004231459	0.594350067
DG 18:2 22:6	IL-1β	-2.10743592	1.325756713	0.122766896	0.075281427	0.036557707	0.048549409
DG 18:2 22:6	IL-6	0.010534055	1.183279934	0.992957951	0.018787294	0.004681938	0.00038648
DG 18:2 22:6	MCP1	-0.662588901	0.9700205	0.499985652	0.003450377	0.000709	3.67568E-05
DG 18:2 22:6	TNF-α	-0.889050943	1.128736507	0.437291414	0.096216968	0.027458538	0.001508175
DG 20:1 20:0	IL-17A	2.307166191	10.29621924	0.826465596	-0.106375524	0.048327758	0.048039585
DG 20:1 20:0	IL-1β	-2.33906492	8.926278692	0.797732648	0.103763262	0.049759655	0.059066663
DG 20:1 20:0	IL-6	-6.346025871	11.68386738	0.596975306	0.032292177	0.016554029	0.074834581
DG 20:1 20:0	MCP1	-1.405654392	10.56238877	0.896334875	0.004359039	0.003085545	0.183143171
DG 20:5 20:0	IL-17A	0.042967153	2.50629093	0.986463686	0.001939956	0.002926088	0.513657165
DG 20:5 20:0	IL-1β	-1.36321E-14	2.771232818	1	1.94902E-15	0.036758122	1
DG 20:5 20:0	IL-6	-1.564944804	1.843625835	0.404357364	0.020028863	0.003265581	2.45713E-06
DG 20:5 20:0	MCP1	-0.718569982	2.101333401	0.735358058	0.003845572	0.000697848	1.14793E-05
DG 20:5 20:0	TNF-α	0.699070588	2.360150444	0.769627204	0.109152054	0.025324542	0.000240041
DG 22:1 20:0	IL-17A	-2.601309093	4.775721493	0.592300069	-0.095675498	0.037214144	0.018708043
DG 22:1 20:0	IL-1β	-3.262080896	4.72154401	0.497987656	0.120663081	0.044903287	0.014587
DG 22:1 20:0	IL-6	0.514395319	3.570300263	0.886957492	0.018292917	0.006568719	0.011806617
DG 22:1 20:0	MCP1	1.778683234	3.478831777	0.615040189	0.003298853	0.001176509	0.011326175
DG 22:1 20:0	TNF-α	-4.07076E-15	6.406022813	1	3.17575E-17	0.068247194	1
DG 22:6 20:0	IL-17A	0.007477191	1.914222632	0.996910119	0.00193942	0.00406468	0.636838858
DG 22:6 20:0	IL-1β	-0.438230456	1.58823766	0.784564387	0.074426244	0.033530449	0.034420124
DG 22:6 20:0	IL-6	-0.107232323	1.29653075	0.934652669	0.018999175	0.003939279	4.14809E-05
DG 22:6 20:0	MCP1	1.033511465	1.012805634	0.315955791	0.003783509	0.000566113	2.49055E-07
DG 22:6 20:0	TNF-α	0.894057121	1.433888431	0.53781704	0.111563989	0.027022896	0.000281927
FA 12:0	IL-1β	-0.000721987	0.005810217	0.901936954	0.073688755	0.034350921	0.040154868
FA 12:0	IL-6	0.000202665	0.004466606	0.964110355	0.018896692	0.003792063	2.44268E-05
FA 12:0	MCP1	-0.001597774	0.004202483	0.706477388	0.003715718	0.000660368	3.97354E-06
FA 12:0	TNF-α	-0.001575885	0.005123666	0.760534892	0.107784975	0.027254776	0.000432383
FA 14:0	IL-17A	2.44502E-17	0.005624342	1	5.36829E-17	0.004288513	1
FA 14:0	IL-1β	-0.004187536	0.003951533	0.297727943	0.071621251	0.029621131	0.021892168
FA 14:0	IL-6	-0.005681856	0.003638418	0.128864174	0.020242108	0.003916522	1.44795E-05
FA 14:0	MCP1	-0.007162944	0.003903998	0.076473761	0.003950329	0.000777821	1.86533E-05
FA 14:0	TNF-α	-0.00727668	0.004663375	0.129156279	0.115723624	0.031452278	0.000914114
FA 14:1	IL-17A	0.000206112	0.001174916	0.861885018	0.001961847	0.00377601	0.607063475
FA 14:1	IL-1β	0.001647978	0.001057683	0.129360843	0.088120876	0.033395936	0.01290374
FA 14:1	IL-6	-0.000976842	0.000814812	0.239668323	0.02032009	0.003599557	3.37859E-06
FA 14:1	MCP1	-0.000776129	0.000914965	0.402794765	0.004122399	0.000761582	6.57154E-06
FA 14:1	TNF-α	-9.72455E-05	0.001191507	0.935477121	0.109826638	0.033900272	0.002853944
FA 15:0	IL-17A	0.000313431	0.004053407	0.938861621	0.00200448	0.003925192	0.613199252
FA 15:0	IL-1β	0.002929256	0.003497577	0.408716629	0.08390791	0.033275109	0.017034239
FA 15:0	IL-6	-0.000608529	0.002770857	0.827608594	0.019090857	0.003688246	1.29616E-05
FA 15:0	MCP1	-0.001349391	0.002677775	0.617878465	0.003951379	0.000671584	1.70922E-06
FA 15:0	TNF-α	0.000608668	0.003393583	0.858823299	0.105158296	0.029092369	0.001052311
FA 16:0	IL-1β	-7.81963E-05	0.000173344	0.655053965	0.071456898	0.033654021	0.041816758

FA 16:0	IL-6	-7.02752E-05	0.000125185	0.578579505	0.018939838	0.003400421	4.19156E-06
FA 16:0	MCP1	-0.000297309	0.000127059	0.025901989	0.00415647	0.000650289	4.04545E-07
FA 16:0	TNF-α	-8.83328E-05	0.000166069	0.598583922	0.109269268	0.029052635	0.000706292
FA 16:1	IL-1β	-0.000936284	0.001061288	0.384450522	0.073191314	0.027870625	0.013297867
FA 16:1	IL-6	-0.000712712	0.000898951	0.433905495	0.019225929	0.003302959	2.04493E-06
FA 16:1	MCP1	-0.002218505	0.000866447	0.015545256	0.004320333	0.000599832	4.23939E-08
FA 16:1	TNF-α	-0.000878513	0.001211865	0.473933857	0.114372223	0.028677153	0.000377409
FA 17:0	IL-17A	4.0188E-05	0.001334607	0.976170569	0.001966745	0.003958978	0.622848252
FA 17:0	IL-1β	0.000684504	0.001237731	0.584208727	0.080999449	0.036071785	0.032007432
FA 17:0	IL-6	-0.000209159	0.000899476	0.817651128	0.018856661	0.003667622	1.432E-05
FA 17:0	MCP1	-0.000964613	0.000820928	0.248930436	0.004031113	0.000630697	4.04779E-07
FA 17:0	TNF-α	-1.42277E-05	0.001087761	0.989647938	0.109820118	0.028565603	0.00056173
FA 18:0	IL-17A	-9.74224E-18	0.000407556	1	3.13292E-16	0.004050915	1
FA 18:0	IL-1β	-0.000101755	0.000331286	0.760781454	0.073132825	0.03235054	0.030949359
FA 18:0	IL-6	-0.000226106	0.000264778	0.399686036	0.019303813	0.003617546	8.19028E-06
FA 18:0	MCP1	-0.000525178	0.000233882	0.032009272	0.004232752	0.000602073	6.80919E-08
FA 18:0	TNF-α	-0.000259225	0.00035349	0.468866128	0.110319457	0.031104516	0.001263991
FA 18:1	IL-17A	-1.4756E-05	0.000161229	0.927666905	0.001899377	0.003843273	0.624643186
FA 18:1	IL-1β	-0.000141941	0.000134996	0.301180724	0.071929506	0.031614665	0.029961146
FA 18:1	IL-6	-8.24157E-05	0.00010269	0.42833599	0.018933982	0.003364738	3.55706E-06
FA 18:1	MCP1	-0.000220887	0.000100771	0.036016651	0.003982733	0.000622124	3.9319E-07
FA 18:1	TNF-α	-0.000143385	0.000153192	0.356517847	0.106742528	0.032327564	0.002424793
FA 18:2	IL-17A	2.28391E-16	0.000170476	1	2.67833E-14	0.004057415	1
FA 18:2	IL-1β	-0.000103652	0.000137064	0.455218181	0.074546249	0.032049559	0.026733221
FA 18:2	IL-6	-7.88319E-05	0.000101965	0.445303589	0.018654517	0.003335831	3.9321E-06
FA 18:2	MCP1	-0.000265609	9.73565E-05	0.010393503	0.003817496	0.00060012	4.40881E-07
FA 18:2	TNF-α	-0.000151595	0.00013852	0.282214319	0.105141992	0.029186356	0.001087622
FA 18:3	IL-17A	-0.003701794	0.003712387	0.326668826	0.001349815	0.003497668	0.702280394
FA 18:3	IL-1β	-0.004467265	0.003327241	0.189462381	0.068618164	0.030818436	0.033634891
FA 18:3	IL-6	-0.003631217	0.002939134	0.226242963	0.018424042	0.003909288	5.23488E-05
FA 18:3	MCP1	-0.005824267	0.002453037	0.024178762	0.003781017	0.000603899	6.73774E-07
FA 18:3	TNF-α	-0.00337616	0.00356929	0.351760859	0.101276229	0.029745681	0.001899765
FA 18:4	IL-1β	-0.024338186	0.034416056	0.484740063	0.075293811	0.031891127	0.024695259
FA 18:4	IL-6	-0.012746598	0.027439333	0.645508932	0.018999412	0.003557426	8.08265E-06
FA 18:4	MCP1	-0.036979308	0.024582972	0.142634206	0.003811796	0.000600506	4.58105E-07
FA 18:4	TNF-α	-0.03301636	0.031355907	0.300500147	0.111678642	0.026181627	0.000173744
FA 20:0	IL-17A	-0.000138496	0.001621603	0.932486972	0.001911482	0.003862676	0.624188668
FA 20:0	IL-1β	-0.000258466	0.001421398	0.856892621	0.075683708	0.033263735	0.029956628
FA 20:0	IL-6	-0.000153595	0.001055356	0.885227607	0.018768992	0.003455478	6.22813E-06
FA 20:0	MCP1	-0.000363595	0.000931337	0.69890946	0.003719742	0.000574561	3.20852E-07
FA 20:0	TNF-α	-0.001499279	0.001198545	0.22032403	0.110141257	0.025274246	0.000133911
FA 20:1	IL-17A	-0.006968805	0.00696509	0.324798766	0.001224314	0.003074943	0.693242377
FA 20:1	IL-1β	-0.006674071	0.007555893	0.383876649	0.067318638	0.032772352	0.048477893
FA 20:1	IL-6	-0.005179506	0.005244876	0.331025402	0.018936583	0.003182809	1.41624E-06
FA 20:1	MCP1	-0.012618238	0.004922378	0.015436051	0.003876897	0.000562822	1.00858E-07
FA 20:1	TNF-α	-0.009478441	0.007685186	0.226719644	0.105327941	0.030036182	0.001407522
FA 20:2	IL-17A	-0.000320446	0.006352638	0.960093051	0.001886132	0.003907443	0.632698346
FA 20:2	IL-1β	-0.004297771	0.005318743	0.425223695	0.070150963	0.032140983	0.036763415
FA 20:2	IL-6	-0.002676757	0.003969742	0.505124571	0.019012909	0.003356341	3.19424E-06
FA 20:2	MCP1	-0.008473129	0.003683941	0.028341481	0.003908835	0.000586863	1.90196E-07

FA 20:2	TNF-α	-0.007463181	0.005050221	0.149552116	0.112949439	0.027499766	0.000270868
FA 20:3	IL-17A	1.26873E-05	0.001281402	0.992163608	0.001941641	0.00387759	0.6200952
FA 20:3	IL-1β	0.000451647	0.001109489	0.686748776	0.078607732	0.032984582	0.023476606
FA 20:3	IL-6	-0.000394677	0.000823539	0.635127792	0.018530092	0.003425517	6.63811E-06
FA 20:3	MCP1	-0.001111034	0.000840536	0.195901507	0.003933051	0.000658747	1.33457E-06
FA 20:3	TNF-α	-0.000214645	0.001103347	0.847022616	0.109262831	0.029557566	0.000842273
FA 20:4	IL-17A	-4.74063E-05	0.000320665	0.883428584	0.001891247	0.003826295	0.624595675
FA 20:4	IL-1β	-0.000292829	0.000276137	0.297132824	0.073959795	0.032371468	0.029329887
FA 20:4	IL-6	-0.000112051	0.000201055	0.581314758	0.018318403	0.003297658	4.37403E-06
FA 20:4	MCP1	-0.000366532	0.000193352	0.067359064	0.003758624	0.00059753	5.38684E-07
FA 20:4	TNF-α	-0.000320551	0.000242888	0.196584107	0.105563346	0.025657423	0.00026558
FA 20:5	IL-17A	5.67089E-18	0.003203182	1	6.0947E-17	0.004060379	1
FA 20:5	IL-1β	-0.00204044	0.002637186	0.44495788	0.0788643	0.032842579	0.022523031
FA 20:5	IL-6	-0.00048785	0.001947853	0.803887671	0.018749205	0.003393957	4.77592E-06
FA 20:5	MCP1	-0.001443006	0.001857196	0.44305885	0.003863537	0.000609717	4.72596E-07
FA 20:5	TNF-α	-0.001541555	0.002634386	0.562671165	0.109056563	0.02956272	0.000859941
FA 22:0	IL-17A	-0.001293115	0.001659816	0.441844608	0.001741229	0.003293491	0.60078692
FA 22:0	IL-1β	-0.000758956	0.001692772	0.657018268	0.069698515	0.032999445	0.042834317
FA 22:0	IL-6	-0.000180229	0.001277517	0.888721183	0.018701087	0.003484403	7.49516E-06
FA 22:0	MCP1	-0.000932563	0.00112403	0.413074348	0.003626043	0.000577644	5.58794E-07
FA 22:0	TNF-α	-0.002036838	0.001417573	0.160777964	0.108316886	0.024901306	0.000136971
FA 22:1	IL-17A	-0.000519139	0.00797534	0.948517865	0.001941867	0.003865059	0.618925425
FA 22:1	IL-1β	-0.006337337	0.006429637	0.331938398	0.075381152	0.03061293	0.01956095
FA 22:1	IL-6	-0.002779953	0.005066076	0.587115479	0.018932217	0.003374766	3.73705E-06
FA 22:1	MCP1	-0.007052369	0.005094573	0.176154434	0.003764521	0.000639441	1.69206E-06
FA 22:1	TNF-α	-0.004999926	0.006025613	0.413008365	0.107416285	0.025851646	0.000236938
FA 22:2	IL-17A	-0.008565393	0.010100934	0.40294904	0.001239933	0.003521395	0.72713524
FA 22:2	IL-1β	-0.012418164	0.008758288	0.166201346	0.072928991	0.029997425	0.021026492
FA 22:2	IL-6	-0.005452289	0.006908888	0.436007004	0.019121605	0.003310751	2.32674E-06
FA 22:2	MCP1	-0.013803381	0.006490937	0.041521795	0.003833313	0.000586066	2.66032E-07
FA 22:2	TNF-α	-0.011715488	0.009425047	0.22318147	0.109260897	0.029088194	0.000715801
FA 22:4	IL-17A	-2.90042E-16	0.007461428	1	5.62591E-15	0.004076815	1
FA 22:4	IL-1β	-0.00483565	0.005445486	0.381373471	0.070133981	0.029231239	0.02262656
FA 22:4	IL-6	-0.004959451	0.004363358	0.26441011	0.018805767	0.003277065	2.58629E-06
FA 22:4	MCP1	-0.011480233	0.004012574	0.007495817	0.004252339	0.000567816	1.94244E-08
FA 22:4	TNF-α	-0.00699157	0.005946153	0.24862056	0.116469034	0.028761742	0.000318336
FA 22:5	IL-17A	2.43425E-05	0.003309495	0.994178472	0.001942999	0.003884727	0.620492194
FA 22:5	IL-1β	-0.000349987	0.002740835	0.899216246	0.073675436	0.03160773	0.026435227
FA 22:5	IL-6	-0.001445803	0.002129199	0.502153757	0.019052421	0.003435424	4.48958E-06
FA 22:5	MCP1	-0.004694671	0.002233564	0.043786278	0.00426314	0.000679021	5.57117E-07
FA 22:5	TNF-α	-0.001233741	0.002917766	0.675332588	0.11210154	0.030320013	0.000840742
FA 22:6	IL-17A	5.56596E-17	0.00131172	1	1.1619E-15	0.004063043	1
FA 22:6	IL-1β	-0.001050309	0.001063603	0.33104271	0.076771904	0.032366925	0.024086641
FA 22:6	IL-6	-0.000331895	0.000783199	0.674660443	0.018228581	0.003334629	5.63702E-06
FA 22:6	MCP1	-0.001128758	0.000738642	0.136614896	0.003782354	0.000592556	4.1449E-07
FA 22:6	TNF-α	-0.00118093	0.000933375	0.215218195	0.111062769	0.025594479	0.000141103
FA 24:0	IL-1β	-0.006899531	0.012505201	0.585087055	0.083040883	0.032497517	0.015736145
FA 24:0	IL-6	-0.002661688	0.009371798	0.778290845	0.018844131	0.003407501	4.69587E-06
FA 24:0	MCP1	0.005584141	0.008135257	0.497555602	0.00384755	0.00055732	9.66578E-08
FA 24:0	TNF-α	-0.000859954	0.011803991	0.942391283	0.107842585	0.0276412	0.000479962

FA 24:1	IL-1β	-0.004299543	0.013398678	0.750444788	0.074281369	0.034007883	0.036631961
FA 24:1	IL-6	-0.003516182	0.009676237	0.718785028	0.018937708	0.003436195	4.95784E-06
FA 24:1	MCP1	-0.00644856	0.010118024	0.528586705	0.003836467	0.000676998	3.17506E-06
FA 24:1	TNF-α	-0.005733527	0.01269559	0.654689792	0.112262215	0.029036151	0.000528976
HexCer 16:0	IL-17A	0.000199012	0.001852343	0.915965926	0.001646829	0.00473042	0.732917434
HexCer 16:0	IL-1β	0.000888766	0.001603183	0.588068628	0.069244855	0.049421339	0.182956458
HexCer 16:0	IL-6	0.000126677	0.00206263	0.951896616	0.020054376	0.014474304	0.187572372
HexCer 16:0	MCP1	0.000503999	0.001542621	0.748716073	0.004508245	0.001845952	0.028465865
HexCer 16:0	TNF-α	0.00082706	0.001743088	0.642473591	0.011097237	0.06425357	0.865351339
HexCer 18:0	IL-17A	0.012580591	0.016671537	0.467878823	-0.00072129	0.005849053	0.904299053
HexCer 18:0	IL-1β	0.00825034	0.014557653	0.583389539	0.043987539	0.080554853	0.596997475
HexCer 18:0	IL-6	0.009844949	0.013969966	0.497067547	0.028064053	0.015044425	0.091696514
HexCer 18:0	MCP1	0.009487812	0.011165944	0.41536606	0.004679179	0.001971576	0.03905537
HexCer 18:0	TNF-α	0.013328505	0.016857894	0.447499542	0.031548627	0.093492436	0.74275693
HexCer 22:0	IL-17A	0.000827884	0.001285876	0.530093144	0.000847472	0.004464364	0.852166863
HexCer 22:0	IL-1β	0.000616531	0.001111801	0.587963464	0.066858294	0.046595154	0.173283729
HexCer 22:0	IL-6	0.000540445	0.001612508	0.742475233	0.019582526	0.015383691	0.223772009
HexCer 22:0	MCP1	0.000542654	0.001137429	0.640661047	0.004553509	0.001850408	0.027466168
HexCer 22:0	TNF-α	0.000848849	0.001297875	0.523691466	0.00882056	0.065041883	0.894057407
HexCer 24:0	IL-17A	0.000138789	0.001132466	0.90420142	0.001681804	0.004536472	0.716388241
HexCer 24:0	IL-1β	0.00052195	0.000918299	0.578775244	0.069026151	0.044404836	0.142381475
HexCer 24:0	IL-6	9.56849E-05	0.001293178	0.942063504	0.020163993	0.014234737	0.178484567
HexCer 24:0	MCP1	0.000383141	0.000965893	0.697591576	0.00458977	0.001813029	0.023959069
HexCer 24:0	TNF-α	0.000528349	0.001203899	0.667456773	0.014908669	0.069611769	0.833503374
HexCer 24:1	IL-17A	0.00106022	0.001354998	0.446982424	0.000387445	0.004552487	0.933382091
HexCer 24:1	IL-1β	0.000713552	0.001127361	0.536974803	0.071156837	0.045722106	0.141950856
HexCer 24:1	IL-6	0.000984013	0.001828119	0.598847982	0.019205128	0.016877682	0.274261484
HexCer 24:1	MCP1	0.00069689	0.001165609	0.559470393	0.00446221	0.001835041	0.029049716
HexCer 24:1	TNF-α	0.001108279	0.001293498	0.405979039	0.007281019	0.062730062	0.909246309
LPC 14:0	IL-17A	-1.738286424	0.807282162	0.039460125	0.002773708	0.004269721	0.520879284
LPC 14:0	IL-1β	-1.631417572	0.671548887	0.021326607	0.075808542	0.034918274	0.037970138
LPC 14:0	IL-6	-0.482747616	0.484008054	0.326549779	0.01777221	0.003613929	2.93893E-05
LPC 14:0	MCP1	-0.537655173	0.486961699	0.27832654	0.003382485	0.000672983	2.16408E-05
LPC 14:0	TNF-α	-1.240371035	0.526504359	0.02521009	0.10268389	0.024631613	0.000239656
LPC 15:0	IL-17A	1.88229E-15	1.223001901	1	3.31651E-17	0.004288473	1
LPC 15:0	IL-1β	-0.14648533	0.960918288	0.879858151	0.071718495	0.033125545	0.038465571
LPC 15:0	IL-6	-0.133772677	0.781723253	0.865274205	0.018843288	0.003869738	3.36799E-05
LPC 15:0	MCP1	-0.684698346	0.681461907	0.323053399	0.003807186	0.000624384	1.06199E-06
LPC 15:0	TNF-α	-0.049212318	0.8884688	0.956194985	0.108720364	0.027557163	0.000443731
LPC 16:0	IL-17A	7.61388E-05	0.009609168	0.993730436	0.001936119	0.004157531	0.644800963
LPC 16:0	IL-1β	0.004404138	0.007615086	0.567348701	0.078432583	0.032391083	0.021716753
LPC 16:0	IL-6	-0.000688469	0.006564923	0.917176364	0.019002952	0.004009887	4.86353E-05
LPC 16:0	MCP1	-0.005925701	0.006074433	0.337105866	0.003949825	0.000686737	2.79709E-06
LPC 16:0	TNF-α	0.001282925	0.007347752	0.862566196	0.108363594	0.028120362	0.000570113
LPC 16:1	IL-17A	0.003219715	0.331022925	0.992303844	0.001938629	0.00410012	0.639761884
LPC 16:1	IL-1β	0.056655601	0.279471047	0.840719097	0.075922613	0.034031125	0.033307626
LPC 16:1	IL-6	-0.036507545	0.230381264	0.875151712	0.018951809	0.004028455	5.36017E-05
LPC 16:1	MCP1	-0.198730672	0.213634157	0.359673289	0.003940977	0.000691423	3.23527E-06
LPC 16:1	TNF-α	0.007233272	0.264201257	0.978339649	0.107488112	0.028946104	0.000833918
LPC 17:0	IL-17A	0.023568268	0.686388091	0.972836148	0.001946063	0.00408316	0.637095242

LPC 17:0	IL-1β	0.328386974	0.55797697	0.560580973	0.072672883	0.032632015	0.033597004
LPC 17:0	IL-6	0.069817864	0.460399496	0.880481149	0.018802844	0.003866467	3.42878E-05
LPC 17:0	MCP1	-0.226181474	0.436435647	0.60808738	0.00376941	0.000678394	4.84436E-06
LPC 17:0	TNF-α	0.365715173	0.492095542	0.463153802	0.105719504	0.025893622	0.000303951
LPC 18:0	IL-17A	0.001169153	0.029211448	0.968339324	0.001916644	0.004122664	0.645356919
LPC 18:0	IL-1β	0.018208005	0.023188948	0.438492634	0.076045798	0.032174133	0.02477437
LPC 18:0	IL-6	0.003620974	0.018884587	0.849235992	0.018777874	0.003762575	2.39167E-05
LPC 18:0	MCP1	-0.009305839	0.019149559	0.630529273	0.003803948	0.000706185	7.81875E-06
LPC 18:0	TNF-α	0.018409524	0.020345284	0.372752099	0.106269653	0.025398338	0.000229685
LPC 18:1	IL-17A	7.71966E-05	0.051415473	0.998811975	0.001941409	0.004087916	0.638287547
LPC 18:1	IL-1β	-2.27071E-08	0.042229781	0.999999575	0.072463759	0.033008664	0.036016602
LPC 18:1	IL-6	-0.008906061	0.035089316	0.801372911	0.019119296	0.003938546	3.51345E-05
LPC 18:1	MCP1	-0.033467997	0.032611106	0.312965772	0.003877298	0.000677499	3.03139E-06
LPC 18:1	TNF-α	-7.95132E-12	0.040335945	1	0.109287962	0.028367241	0.000571594
LPC 18:2	IL-17A	-8.80847E-17	0.030662326	1	3.23239E-17	0.004319652	1
LPC 18:2	IL-1β	-0.015019275	0.023100073	0.520522375	0.076780009	0.031993224	0.0228125
LPC 18:2	IL-6	-0.015545187	0.018076462	0.396626881	0.019536399	0.003595092	6.83669E-06
LPC 18:2	MCP1	-0.021212581	0.017751381	0.241455844	0.003688929	0.000653447	3.77097E-06
LPC 18:2	TNF-α	-0.007627283	0.022282437	0.734511593	0.108740101	0.027766625	0.000480467
LPC 18:3	IL-17A	4.61878E-15	1.495930676	1	7.97705E-17	0.004331893	1
LPC 18:3	IL-1β	-1.318983419	1.15004041	0.260487357	0.081520452	0.032740125	0.018544552
LPC 18:3	IL-6	-0.513711233	0.877582723	0.562676244	0.018869275	0.003587626	1.11929E-05
LPC 18:3	MCP1	-1.101772461	0.857857333	0.208856163	0.003754121	0.000649107	2.55716E-06
LPC 18:3	TNF-α	-0.545223881	1.079315472	0.617139664	0.106572992	0.027645942	0.000568004
LPC 19:0	IL-17A	-0.027556205	5.537294743	0.996063441	0.001947054	0.004334093	0.656597033
LPC 19:0	IL-1β	-0.503354802	4.460165399	0.910922623	0.070641014	0.034384812	0.04904432
LPC 19:0	IL-6	0.675727401	3.630489424	0.853642037	0.018772698	0.00411162	8.46503E-05
LPC 19:0	MCP1	-1.539592913	3.421298282	0.656054553	0.0036949	0.000711461	1.48181E-05
LPC 19:0	TNF-α	-0.378971289	3.834136304	0.92194429	0.109250927	0.026529232	0.00029002
LPC 20:0	IL-1β	-4.693968914	5.484365505	0.398847744	0.07652883	0.036261428	0.043263135
LPC 20:0	IL-6	-1.26425875	3.990080669	0.753551578	0.018823504	0.003788367	2.54445E-05
LPC 20:0	MCP1	-3.804119172	3.678954943	0.309389966	0.003683455	0.000646512	3.257E-06
LPC 20:0	TNF-α	-1.735154547	4.664109406	0.712490975	0.108220218	0.027746167	0.000501747
LPC 20:1	IL-17A	6.05374E-16	2.94829288	1	6.0547E-18	0.004295989	1
LPC 20:1	IL-1β	-0.537576125	2.403873175	0.824561768	0.07067309	0.034435393	0.048950816
LPC 20:1	IL-6	-0.205430974	1.889902067	0.914164822	0.019050137	0.003887628	3.08771E-05
LPC 20:1	MCP1	-1.408884501	1.673041257	0.406389892	0.003751125	0.000636992	1.90325E-06
LPC 20:1	TNF-α	-0.179893064	2.159190561	0.934154491	0.108535193	0.027829167	0.000502179
LPC 20:2	IL-17A	3.93456E-15	1.804400616	1	1.63451E-17	0.004299163	1
LPC 20:2	IL-1β	-0.731553652	1.467442029	0.621751004	0.075540061	0.034372641	0.035829567
LPC 20:2	IL-6	-0.668103396	1.060683735	0.533541571	0.019025559	0.003567712	9.10382E-06
LPC 20:2	MCP1	-1.251763629	1.030846542	0.234103217	0.003760347	0.000641771	2.06718E-06
LPC 20:2	TNF-α	-0.433451747	1.292697441	0.739726307	0.110670768	0.027243605	0.000321699
LPC 20:3	IL-17A	3.55361E-16	0.486916512	1	3.34226E-17	0.004295517	1
LPC 20:3	IL-1β	-0.071164212	0.377260222	0.851649756	0.06984555	0.03271922	0.041072019
LPC 20:3	IL-6	-0.132172947	0.3099776	0.672864081	0.019044111	0.003860505	2.81422E-05
LPC 20:3	MCP1	-0.303518433	0.299857721	0.319533153	0.003808393	0.000691212	5.52485E-06
LPC 20:3	TNF-α	0.005717543	0.35941002	0.987413008	0.108909102	0.028045801	0.000525752
LPC 20:4	IL-17A	-1.27648E-15	0.15936305	1	3.34061E-17	0.004290008	1
LPC 20:4	IL-1β	-0.095730478	0.125290543	0.450794866	0.072622605	0.033158112	0.036419835

LPC 20:4	IL-6	-0.053807436	0.095748973	0.578315699	0.018747212	0.003638794	1.51639E-05
LPC 20:4	MCP1	-0.121044299	0.089197219	0.184891354	0.003755463	0.000627418	1.4516E-06
LPC 20:4	TNF-α	-0.033124309	0.117033389	0.779095435	0.107648685	0.027867437	0.000555809
LPC 20:5	IL-17A	-0.089177525	1.721634151	0.959032872	0.001933588	0.004083911	0.639309027
LPC 20:5	IL-1β	-2.296876141	1.474969485	0.129902315	0.084707571	0.034396909	0.019752868
LPC 20:5	IL-6	-0.235555012	1.151154575	0.839247084	0.01880958	0.003854978	3.27524E-05
LPC 20:5	MCP1	-0.616522699	0.974294694	0.531665766	0.003644047	0.000603894	1.26703E-06
LPC 20:5	TNF-α	-0.550915918	1.333656185	0.6824807	0.10535761	0.027983112	0.000725172
LPC 22:0	IL-1β	-7.157659289	3.920538826	0.077867838	0.091658388	0.039652787	0.027854162
LPC 22:0	IL-6	-0.160118985	2.696228742	0.95303828	0.018881523	0.003915945	3.85283E-05
LPC 22:0	MCP1	-0.292260804	2.325605371	0.900830898	0.003649946	0.00062517	2.19258E-06
LPC 22:0	TNF-α	-1.345235325	3.102044615	0.667637333	0.104712839	0.028228757	0.000842857
LPC 22:1	IL-17A	-8.092945285	12.85398814	0.533718541	0.001969991	0.003651496	0.593522291
LPC 22:1	IL-1β	-22.39131398	14.45165248	0.131773026	0.08391507	0.040360029	0.04624923
LPC 22:1	IL-6	-7.696730912	8.380564824	0.365733576	0.018417152	0.003360928	6.01167E-06
LPC 22:1	MCP1	-9.913302561	8.520947808	0.253835983	0.003614212	0.000632493	3.10667E-06
LPC 22:1	TNF-α	-9.338498766	10.51587205	0.381584962	0.104352912	0.026423783	0.000438969
LPC 22:2	IL-17A	-0.783298406	20.5024814	0.969840215	0.001940527	0.003411102	0.574719244
LPC 22:2	IL-1β	-24.94981283	20.2666013	0.230221234	0.040302392	0.039600147	0.318955182
LPC 22:2	IL-6	1.493926067	14.29732622	0.917648925	0.01961704	0.003696769	1.91612E-05
LPC 22:2	MCP1	8.148717243	12.37321163	0.516436028	0.003959434	0.000580924	4.75461E-07
LPC 22:2	TNF-α	-6.63569E-05	14.24873793	0.999996323	0.121211841	0.022255565	1.34844E-05
LPC 22:4	IL-17A	-0.062824103	1.167817472	0.957454216	0.00194987	0.004077954	0.63601012
LPC 22:4	IL-1β	-1.420350855	1.062507465	0.191340548	0.08364633	0.036475422	0.029016919
LPC 22:4	IL-6	-0.830626921	0.670211881	0.224824379	0.018431327	0.003303942	4.55053E-06
LPC 22:4	MCP1	-0.807028543	0.671570146	0.238879085	0.003647873	0.000612765	1.58952E-06
LPC 22:4	TNF-α	-0.649104875	0.880313771	0.466634054	0.105136979	0.027190775	0.000550143
LPC 22:5	IL-17A	0.009895486	1.700983507	0.99539684	0.001941841	0.004088263	0.638241449
LPC 22:5	IL-1β	-0.245974913	1.35295607	0.856958208	0.072347273	0.031968585	0.031028599
LPC 22:5	IL-6	-0.362701614	1.168793366	0.758462226	0.019151209	0.003965787	3.77324E-05
LPC 22:5	MCP1	-0.882483395	1.066623298	0.41456501	0.00377559	0.000669861	3.8672E-06
LPC 22:5	TNF-α	0.24616752	1.31324668	0.852570221	0.107868205	0.027919123	0.000554732
LPC 22:6	IL-17A	-1.27655E-15	0.765085397	1	3.15344E-17	0.004294697	1
LPC 22:6	IL-1β	-0.244850149	0.60927915	0.690629507	0.07193067	0.033623269	0.040665687
LPC 22:6	IL-6	-0.023077781	0.480652558	0.962023756	0.018996254	0.003808963	2.41506E-05
LPC 22:6	MCP1	-0.249911524	0.42187365	0.558031904	0.003702532	0.000618785	1.4599E-06
LPC 22:6	TNF-α	-0.123005123	0.550563441	0.824725517	0.107514798	0.027336741	0.000458922
LPC 24:0	IL-17A	-0.274626111	4.434643094	0.951031364	0.00197335	0.004073189	0.631568673
LPC 24:0	IL-1β	-8.488899406	4.223421863	0.053503037	0.087890816	0.038136547	0.028286873
LPC 24:0	IL-6	-0.86857034	2.946258064	0.770173689	0.018478009	0.003820319	3.69257E-05
LPC 24:0	MCP1	-0.623392608	2.573532369	0.810248527	0.003643132	0.000617647	1.85277E-06
LPC 24:0	TNF-α	-1.785941948	3.469966196	0.610544046	0.104370722	0.028191481	0.000859465
LPC 24:1	IL-17A	-9.294341279	8.816531574	0.300806043	0.002356832	0.00449861	0.604469081
LPC 24:1	IL-1β	-15.43167692	7.318212764	0.044047837	0.090497144	0.038588301	0.026333535
LPC 24:1	IL-6	-0.995293325	5.454033813	0.85651497	0.018700878	0.004073517	8.48256E-05
LPC 24:1	MCP1	-1.594356465	4.596992667	0.731315207	0.003629811	0.000633066	3.75581E-06
LPC 24:1	TNF-α	-2.985007418	5.630998652	0.60021974	0.11174393	0.026580323	0.000242614
LPE 14:1	IL-17A	3.280541562	2.172253012	0.153228856	-0.142161175	0.045287328	0.007247041
LPE 14:1	IL-1β	-3.064943671	1.52776044	0.064557654	0.108959501	0.038529631	0.013421109
LPE 14:1	IL-6	-0.563980535	1.42266839	0.697768432	0.017913391	0.005764609	0.007716918

LPE 14:1	MCP1	-1.106700354	2.081932296	0.603354282	0.002322555	0.001541771	0.154187185
LPE 14:1	TNF-α	-2.983575925	2.650875126	0.279300039	-0.008084239	0.062894701	0.899552967
LPE 16:0	IL-17A	0.000389095	0.539881295	0.999429896	0.001940595	0.00423082	0.649882712
LPE 16:0	IL-1β	0.167830884	0.463040374	0.719640905	0.08224791	0.036681516	0.032761859
LPE 16:0	IL-6	-0.048062009	0.337566035	0.887765958	0.019082833	0.003794372	2.33856E-05
LPE 16:0	MCP1	-0.381809896	0.308077738	0.22515989	0.004050075	0.000639926	6.48612E-07
LPE 16:0	TNF-α	-0.192261917	0.381947668	0.618504705	0.117847933	0.027704131	0.00020005
LPE 18:0	IL-17A	0.004603929	0.755312467	0.995178345	0.001937831	0.00433057	0.65785538
LPE 18:0	IL-1β	0.285729433	0.611434857	0.643770737	0.07233878	0.034528977	0.045015747
LPE 18:0	IL-6	-0.029186414	0.520526452	0.955669824	0.019041431	0.004318256	0.000130344
LPE 18:0	MCP1	-0.406176982	0.485878745	0.410010615	0.003739102	0.000740127	2.19537E-05
LPE 18:0	TNF-α	-0.091437898	0.554664492	0.870203731	0.111879665	0.028112879	0.000422772
LPE 18:1	IL-1β	-0.505213894	0.506634799	0.326645437	0.077649162	0.032109652	0.021875187
LPE 18:1	IL-6	-0.279010354	0.394412433	0.484772757	0.018978786	0.003589575	1.03521E-05
LPE 18:1	MCP1	-0.403998636	0.393041675	0.312224188	0.003736515	0.000662084	3.78976E-06
LPE 18:1	TNF-α	-0.220525371	0.485296249	0.652801895	0.108204695	0.027673447	0.000488632
LPE 18:2	IL-17A	-0.002626462	0.363338245	0.994280241	0.001941509	0.00411607	0.640560734
LPE 18:2	IL-1β	-0.12021077	0.301743071	0.693165597	0.072293933	0.033605463	0.039631232
LPE 18:2	IL-6	-0.122630067	0.241624774	0.615499129	0.019020198	0.003864256	2.90277E-05
LPE 18:2	MCP1	-0.271163255	0.23271651	0.253116149	0.003766537	0.000688864	6.21917E-06
LPE 18:2	TNF-α	-0.109807369	0.281487787	0.699221313	0.111091716	0.028206415	0.000452001
LPE 18:3	IL-17A	-6.664538121	10.77315231	0.546850776	-0.150320083	0.06982047	0.050682378
LPE 18:3	IL-1β	-8.147278469	9.271547073	0.39549439	0.091001378	0.046538127	0.072376658
LPE 18:3	IL-6	0.611652939	5.77037654	0.917201505	0.019363814	0.00381059	0.000210436
LPE 18:3	MCP1	2.054899327	3.065741482	0.514411846	0.004305144	0.000396168	6.80105E-08
LPE 18:3	TNF-α	-2.711420616	6.322813601	0.675066557	0.125910796	0.027317638	0.000489499
LPE 20:3	IL-17A	0.009272404	4.187881544	0.998248563	0.001946444	0.004327076	0.656178315
LPE 20:3	IL-1β	-1.629098353	3.484143697	0.643583243	0.074164172	0.035457753	0.045341162
LPE 20:3	IL-6	-0.695222638	2.743719417	0.801756971	0.018983778	0.004101918	7.12464E-05
LPE 20:3	MCP1	-1.082423569	2.439538553	0.660552376	0.003669282	0.00066968	6.70318E-06
LPE 20:3	TNF-α	-0.393766581	2.892054188	0.892639815	0.109445883	0.026415755	0.000270824
LPE 20:4	IL-1β	-0.536711941	0.500507752	0.292120421	0.082670613	0.035381995	0.02633436
LPE 20:4	IL-6	-0.210852282	0.351494012	0.553094327	0.018666644	0.003568136	1.2116E-05
LPE 20:4	MCP1	-0.378801587	0.353237638	0.29210475	0.003607957	0.000663701	6.79952E-06
LPE 20:4	TNF-α	-0.226061442	0.439151645	0.610486857	0.108479036	0.027931983	0.00052514
LPE 20:5	IL-17A	-0.564626397	4.925563836	0.909500743	0.00195477	0.004059392	0.633625152
LPE 20:5	IL-1β	-8.073560758	4.917184283	0.111052499	0.090187743	0.039840295	0.030983401
LPE 20:5	IL-6	0.3101406	3.507277725	0.930124395	0.01904152	0.004080638	5.96795E-05
LPE 20:5	MCP1	-0.40570385	3.009743513	0.893672854	0.0036318	0.000648141	4.24327E-06
LPE 20:5	TNF-α	-2.127522274	3.823992928	0.582090381	0.102860048	0.027876541	0.000888608
LPE 22:4	IL-17A	-3.674920731	3.879784686	0.354843301	0.001652794	0.004341317	0.707430265
LPE 22:4	IL-1β	-4.901799006	3.043906793	0.122989019	0.051125944	0.046053017	0.280102391
LPE 22:4	IL-6	-0.53382823	1.724241954	0.760066467	0.018894774	0.003186677	8.4621E-06
LPE 22:4	MCP1	-1.211341983	1.800454135	0.508775571	0.003610332	0.000592582	5.91532E-06
LPE 22:4	TNF-α	-1.247581067	1.798850873	0.495945234	0.114702251	0.020123715	1.40726E-05
LPE 22:5	IL-17A	-0.117302094	4.230880236	0.978101446	0.001956117	0.004781081	0.685923169
LPE 22:5	IL-1β	-4.018495022	3.271978682	0.230826605	0.059574566	0.042639625	0.174642784
LPE 22:5	IL-6	-0.705261779	2.216693527	0.753006623	0.019035859	0.0037993	3.62854E-05
LPE 22:5	MCP1	-1.411334272	2.050112302	0.4975286	0.003620117	0.000648662	8.35957E-06
LPE 22:5	TNF-α	-0.715277079	2.256848819	0.753923591	0.111016692	0.023751495	8.67174E-05

LPE 22:6	IL-17A	-2.017224667	1.036725554	0.062161612	0.000962582	0.004420201	0.829246607
LPE 22:6	IL-1β	-1.85944356	0.82253112	0.032051839	0.067303706	0.036372901	0.075230556
LPE 22:6	IL-6	-0.110661013	0.766798588	0.886323069	0.018718493	0.00493145	0.000757835
LPE 22:6	MCP1	-0.679191924	0.609910901	0.27527007	0.003397557	0.000716384	6.08253E-05
LPE 22:6	TNF-α	-0.809700885	0.61224137	0.197089118	0.10473124	0.024131053	0.000179051
TG 36:0 FA12:0	IL-17A	0.441504661	0.458399352	0.343174964	0.000972108	0.004150202	0.816396491
TG 36:0 FA12:0	IL-1β	0.195443025	0.364578608	0.595856627	0.068378855	0.032450201	0.043567821
TG 36:0 FA12:0	IL-6	0.205568557	0.275267585	0.461000166	0.018728238	0.003518303	9.35438E-06
TG 36:0 FA12:0	MCP1	0.0123041	0.265133781	0.9632933	0.003703214	0.000627228	1.82344E-06
TG 36:0 FA12:0	TNF-α	0.052794612	0.366927599	0.886555113	0.105496803	0.029384773	0.00116128
TG 38:0 FA12:0	IL-17A	0.021651199	0.554668958	0.969121532	0.001918926	0.004143464	0.646619716
TG 38:0 FA12:0	IL-1β	0.319632846	0.401718168	0.432478033	0.066309891	0.029502048	0.032106518
TG 38:0 FA12:0	IL-6	0.277054541	0.312992852	0.383100509	0.018642893	0.003300783	3.74256E-06
TG 38:0 FA12:0	MCP1	0.025632834	0.319434633	0.936575821	0.003698143	0.000623515	1.69053E-06
TG 38:0 FA12:0	TNF-α	0.061955006	0.43392151	0.887419153	0.106361961	0.028671976	0.000842458
TG 40:0 FA12:0	IL-17A	0.134415643	0.314024527	0.671678016	0.001165072	0.004119472	0.77925658
TG 40:0 FA12:0	IL-1β	0.223734336	0.226127592	0.330372956	0.06520084	0.029163022	0.032960346
TG 40:0 FA12:0	IL-6	0.19378567	0.173722285	0.273496081	0.018398281	0.003217262	3.06862E-06
TG 40:0 FA12:0	MCP1	0.066060334	0.1826605	0.720143702	0.003556145	0.00062612	3.42388E-06
TG 40:0 FA12:0	TNF-α	0.052002601	0.24415029	0.83277225	0.105324213	0.028330352	0.000824227
TG 40:0 FA14:0	IL-17A	0.008472291	0.611603751	0.989039307	0.001941928	0.004091614	0.638500585
TG 40:0 FA14:0	IL-1β	0.286796204	0.449466579	0.528261779	0.074417911	0.02956126	0.017395806
TG 40:0 FA14:0	IL-6	0.274556841	0.342806807	0.429482411	0.018811468	0.003237628	2.37221E-06
TG 40:0 FA14:0	MCP1	0.099510533	0.344554796	0.774713841	0.003642769	0.000602307	1.2192E-06
TG 40:0 FA14:0	TNF-α	0.007396696	0.473137978	0.987630467	0.108577361	0.027998135	0.000533323
TG 40:0 FA16:0	IL-17A	0.006537505	0.369467519	0.98599981	0.001931303	0.004200388	0.648980808
TG 40:0 FA16:0	IL-1β	0.19814601	0.267158296	0.464048291	0.072655721	0.02985952	0.021135851
TG 40:0 FA16:0	IL-6	0.176388591	0.201446158	0.388198291	0.018898421	0.003233144	2.15065E-06
TG 40:0 FA16:0	MCP1	0.069105031	0.204396291	0.737647473	0.003654763	0.000607185	1.32147E-06
TG 40:0 FA16:0	TNF-α	0.040757796	0.275857486	0.883529065	0.106061217	0.027740525	0.000619029
TG 42:0 FA12:0	IL-17A	0.003381589	0.228491593	0.98829003	0.001934733	0.004157184	0.64500946
TG 42:0 FA12:0	IL-1β	0.110418637	0.163524583	0.504693764	0.072649433	0.029249139	0.018809204
TG 42:0 FA12:0	IL-6	0.118705053	0.127593179	0.359621436	0.018756927	0.003277249	3.02783E-06
TG 42:0 FA12:0	MCP1	0.036469957	0.130604898	0.781977651	0.003597307	0.000620902	2.48528E-06
TG 42:0 FA12:0	TNF-α	0.01646862	0.172855961	0.92473102	0.10628458	0.027818272	0.000623517
TG 42:0 FA14:0	IL-17A	0.002915218	0.250680085	0.990798414	0.001938199	0.004130353	0.642278341
TG 42:0 FA14:0	IL-1β	0.095010243	0.187151982	0.615401531	0.072643295	0.030315363	0.023002032
TG 42:0 FA14:0	IL-6	0.101061391	0.145631458	0.493050639	0.018846481	0.00338747	4.74695E-06
TG 42:0 FA14:0	MCP1	0.04435697	0.141121112	0.755454709	0.003646143	0.000607567	1.38954E-06
TG 42:0 FA14:0	TNF-α	0.002846181	0.192422488	0.988296603	0.108807196	0.028043997	0.000530632
TG 42:0 FA16:0	IL-17A	0.001546523	0.1385639	0.991168825	0.001936333	0.004097746	0.639963864
TG 42:0 FA16:0	IL-1β	0.058061802	0.104561087	0.582813203	0.076875069	0.030399469	0.016938278
TG 42:0 FA16:0	IL-6	0.050590488	0.08230153	0.543389494	0.018862965	0.00343602	5.84472E-06
TG 42:0 FA16:0	MCP1	0.024968292	0.079336427	0.755157227	0.003675156	0.000613059	1.41477E-06
TG 42:0 FA16:0	TNF-α	1.11434E-07	0.105066385	0.999999161	0.109289596	0.027483716	0.00040728
TG 42:1 FA12:0	IL-17A	0.003572947	0.262228923	0.98922223	0.001923609	0.004550426	0.675609206
TG 42:1 FA12:0	IL-1β	0.121822941	0.18246382	0.509632387	0.066921613	0.031617054	0.042992734
TG 42:1 FA12:0	IL-6	0.119839621	0.139194469	0.396327695	0.018493069	0.003470305	1.01686E-05
TG 42:1 FA12:0	MCP1	0.058347964	0.14142501	0.682954688	0.003590114	0.000651929	6.20693E-06
TG 42:1 FA12:0	TNF-α	0.022794163	0.18489181	0.902732857	0.103629064	0.028640026	0.001115311

TG 42:1 FA14:0	IL-17A	0.00757023	0.691136541	0.991333219	0.001937665	0.004088763	0.639002061
TG 42:1 FA14:0	IL-1β	0.262863293	0.535195812	0.626891915	0.075767067	0.031127321	0.021094639
TG 42:1 FA14:0	IL-6	0.246338932	0.418196168	0.560236813	0.018832771	0.003492698	7.69997E-06
TG 42:1 FA14:0	MCP1	0.125471736	0.395613801	0.753321154	0.003690448	0.000611553	1.26597E-06
TG 42:1 FA14:0	TNF-α	-0.000911726	0.524162577	0.99862368	0.109286825	0.027429052	0.000398625
TG 42:1 FA16:0	IL-17A	-0.000316012	0.327323965	0.999236082	0.001943818	0.004091469	0.638163289
TG 42:1 FA16:0	IL-1β	0.121598889	0.25262291	0.633763505	0.076197567	0.031043824	0.020126984
TG 42:1 FA16:0	IL-6	0.113371076	0.199769555	0.574590993	0.018970148	0.003525199	7.937E-06
TG 42:1 FA16:0	MCP1	0.056798441	0.180230881	0.754835027	0.003705648	0.000588661	6.13048E-07
TG 42:1 FA16:0	TNF-α	-0.010726764	0.246240352	0.96554207	0.109251471	0.027225599	0.00036862
TG 42:1 FA16:1	IL-17A	-0.000812868	0.849807472	0.999243131	0.001940851	0.004089849	0.638541785
TG 42:1 FA16:1	IL-1β	0.244834628	0.659417066	0.713031258	0.073072676	0.031199517	0.026004727
TG 42:1 FA16:1	IL-6	0.290372008	0.51908884	0.580049689	0.018892107	0.003526804	8.50709E-06
TG 42:1 FA16:1	MCP1	0.144609396	0.476454174	0.763594966	0.003696318	0.00059916	8.69769E-07
TG 42:1 FA16:1	TNF-α	-0.027979977	0.639332734	0.965382253	0.109244394	0.027216381	0.000367508
TG 42:1 FA18:1	IL-17A	0.00499787	0.285383737	0.986143471	0.001934953	0.004159345	0.645143167
TG 42:1 FA18:1	IL-1β	0.13297252	0.214956723	0.540847909	0.072270542	0.03079979	0.025750768
TG 42:1 FA18:1	IL-6	0.120379516	0.165970927	0.473885171	0.018892388	0.003414921	5.18432E-06
TG 42:1 FA18:1	MCP1	0.089778834	0.157736794	0.57347883	0.003625408	0.000600708	1.26359E-06
TG 42:1 FA18:1	TNF-α	0.033254103	0.208699509	0.874469218	0.107579367	0.026905056	0.000383443
TG 42:2 FA12:0	IL-17A	0.010787227	1.167620261	0.992715939	0.001912379	0.00660497	0.775010338
TG 42:2 FA12:0	IL-1β	0.442698726	0.659317567	0.509253373	0.090990895	0.039219326	0.030492226
TG 42:2 FA12:0	IL-6	0.425355746	0.562692396	0.458087819	0.018198691	0.005397715	0.002884233
TG 42:2 FA12:0	MCP1	0.190644374	0.547850179	0.731314435	0.003667237	0.00099403	0.001362406
TG 42:2 FA12:0	TNF-α	0.216146728	0.718164806	0.766396392	0.098121769	0.041200219	0.026776402
TG 42:2 FA18:2	IL-17A	0.011773192	0.696921367	0.986633715	0.001919809	0.004303832	0.6587499
TG 42:2 FA18:2	IL-1β	0.346734719	0.506726073	0.499060352	0.072849771	0.030764213	0.02452684
TG 42:2 FA18:2	IL-6	0.306890088	0.395677652	0.444052956	0.019176241	0.003449578	4.80853E-06
TG 42:2 FA18:2	MCP1	0.220216234	0.378401291	0.564938519	0.003635435	0.000610603	1.5864E-06
TG 42:2 FA18:2	TNF-α	0.074456027	0.497282832	0.881983095	0.106738145	0.027163878	0.000463422
TG 44:0 FA12:0	IL-17A	0.00294575	0.176987822	0.986830983	0.001927705	0.004181278	0.64810044
TG 44:0 FA12:0	IL-1β	0.07574772	0.132184254	0.570883932	0.072948189	0.030700566	0.024079666
TG 44:0 FA12:0	IL-6	0.065987122	0.103116966	0.52707871	0.018882233	0.003439131	5.83422E-06
TG 44:0 FA12:0	MCP1	0.051069163	0.098708107	0.608688097	0.003650419	0.000609331	1.43035E-06
TG 44:0 FA12:0	TNF-α	0.001968079	0.134411454	0.98841458	0.108909752	0.028087862	0.000534131
TG 44:0 FA14:0	IL-17A	-0.000160492	0.151841561	0.99916366	0.001941288	0.004092233	0.638661273
TG 44:0 FA14:0	IL-1β	0.047766455	0.118044977	0.688608312	0.075372673	0.031276501	0.022297787
TG 44:0 FA14:0	IL-6	0.029171165	0.091159896	0.751186434	0.018877228	0.003468378	6.67506E-06
TG 44:0 FA14:0	MCP1	0.000336318	0.086002753	0.996905726	0.003708848	0.000605643	9.86874E-07
TG 44:0 FA14:0	TNF-α	-0.005588121	0.113708705	0.961130132	0.109219968	0.027106916	0.00035235
TG 44:0 FA16:0	IL-17A	0.001220045	0.072475592	0.986680607	0.001925606	0.004181385	0.648464795
TG 44:0 FA16:0	IL-1β	0.031163444	0.054859707	0.574223723	0.073381497	0.031115935	0.025070041
TG 44:0 FA16:0	IL-6	0.025703493	0.042050624	0.545637547	0.018799984	0.003424946	5.85525E-06
TG 44:0 FA16:0	MCP1	0.02101172	0.04020568	0.605086961	0.003639767	0.000606109	1.3744E-06
TG 44:0 FA16:0	TNF-α	0.000810551	0.05500925	0.988341296	0.109004116	0.028072484	0.000526192
TG 44:0 FA18:0	IL-17A	0.013968736	0.61622429	0.982064996	0.001939753	0.004097296	0.639338533
TG 44:0 FA18:0	IL-1β	0.374900008	0.433344434	0.393832151	0.069739113	0.028326487	0.019783682
TG 44:0 FA18:0	IL-6	0.332884565	0.344222812	0.341245341	0.018767611	0.003231105	2.38452E-06
TG 44:0 FA18:0	MCP1	0.112006307	0.34583688	0.748283369	0.003618166	0.000600848	1.312E-06
TG 44:0 FA18:0	TNF-α	0.087357088	0.476006532	0.85562419	0.106958078	0.027995496	0.000623736

TG 44:1 FA12:0	IL-17A	0.00163927	0.150407662	0.991376306	0.001934135	0.004107164	0.641106033
TG 44:1 FA12:0	IL-1β	0.053665289	0.114127007	0.641595403	0.072816125	0.030638076	0.024050397
TG 44:1 FA12:0	IL-6	0.053301961	0.091833276	0.565965465	0.018794613	0.003540177	9.73546E-06
TG 44:1 FA12:0	MCP1	0.037544333	0.083841628	0.657514177	0.003678235	0.000598228	9.21158E-07
TG 44:1 FA12:0	TNF-α	3.187E-06	0.113816136	0.999977844	0.10928912	0.027491133	0.000408501
TG 44:1 FA14:0	IL-17A	0.002043803	0.182271718	0.991127789	0.001936464	0.004106681	0.640666579
TG 44:1 FA14:0	IL-1β	0.067502062	0.138378901	0.629233395	0.073550477	0.03065085	0.022826063
TG 44:1 FA14:0	IL-6	0.064269274	0.107689394	0.555116949	0.018783843	0.003425291	5.94282E-06
TG 44:1 FA14:0	MCP1	0.032751242	0.101229686	0.748535522	0.003672323	0.000595956	8.87133E-07
TG 44:1 FA14:0	TNF-α	4.95589E-05	0.138165687	0.99971618	0.109281791	0.027535222	0.000416003
TG 44:1 FA14:1	IL-17A	-0.001268488	1.156885714	0.999132404	0.001941627	0.004089179	0.638353277
TG 44:1 FA14:1	IL-1β	0.088700157	0.95762575	0.926817217	0.07289404	0.033276869	0.036392801
TG 44:1 FA14:1	IL-6	0.01890844	0.767099344	0.980497951	0.018932417	0.003827808	2.71316E-05
TG 44:1 FA14:1	MCP1	-0.020408552	0.65665743	0.975412029	0.003714661	0.000606484	9.83868E-07
TG 44:1 FA14:1	TNF-α	-0.110216528	0.874609934	0.900558508	0.10808278	0.027344929	0.000434936
TG 44:1 FA16:0	IL-17A	-9.85227E-05	0.103277644	0.999245166	0.001940971	0.004088148	0.638381923
TG 44:1 FA16:0	IL-1β	0.039854757	0.080794636	0.62540324	0.077861932	0.031441536	0.019134828
TG 44:1 FA16:0	IL-6	0.035863918	0.062894233	0.572770793	0.018882113	0.003514665	8.13933E-06
TG 44:1 FA16:0	MCP1	0.018227297	0.057111274	0.751821401	0.003699555	0.000590714	6.70325E-07
TG 44:1 FA16:0	TNF-α	-0.0019584	0.078287637	0.980208301	0.109257636	0.027411333	0.000396975
TG 44:1 FA16:1	IL-17A	0.00399766	0.364924233	0.991332062	0.001933454	0.004100116	0.640652033
TG 44:1 FA16:1	IL-1β	0.135983356	0.281091789	0.63206277	0.075633029	0.031048642	0.021005172
TG 44:1 FA16:1	IL-6	0.117881974	0.217881538	0.592477249	0.018858531	0.003455945	6.41353E-06
TG 44:1 FA16:1	MCP1	0.06546563	0.20678843	0.753752772	0.003678441	0.000607092	1.18205E-06
TG 44:1 FA16:1	TNF-α	-0.00703856	0.276302239	0.979845479	0.109314936	0.027459625	0.000402378
TG 44:1 FA18:1	IL-17A	0.001077599	0.09706541	0.99121574	0.00193474	0.004090956	0.639685268
TG 44:1 FA18:1	IL-1β	0.039885579	0.074941072	0.598489568	0.073565577	0.031051398	0.024462941
TG 44:1 FA18:1	IL-6	0.03678053	0.058790605	0.536295546	0.01883137	0.00349801	7.8889E-06
TG 44:1 FA18:1	MCP1	0.028571027	0.054175502	0.601809175	0.003671878	0.00059662	9.06115E-07
TG 44:1 FA18:1	TNF-α	0.001039587	0.073636577	0.988829462	0.109098239	0.027451786	0.000409911
TG 44:2 FA12:0	IL-17A	0.002153029	0.399418565	0.995734784	0.001935742	0.004125396	0.642300669
TG 44:2 FA12:0	IL-1β	0.142360599	0.305978259	0.645102378	0.072665237	0.031069118	0.026197825
TG 44:2 FA12:0	IL-6	0.141789885	0.246077779	0.56877841	0.018816931	0.003588084	1.16858E-05
TG 44:2 FA12:0	MCP1	0.079685707	0.222681762	0.72296434	0.003686669	0.000600976	9.57999E-07
TG 44:2 FA12:0	TNF-α	0.004224536	0.30618443	0.989083001	0.107816661	0.027972881	0.000568926
TG 44:2 FA14:0	IL-17A	-0.001051125	0.521058813	0.998404253	0.001943081	0.004382938	0.660818058
TG 44:2 FA14:0	IL-1β	0.136764425	0.384946262	0.724949567	0.069761798	0.032333437	0.039380948
TG 44:2 FA14:0	IL-6	0.176396089	0.314054922	0.578654386	0.018817504	0.003795407	2.851E-05
TG 44:2 FA14:0	MCP1	0.082901595	0.284575598	0.772882761	0.003649379	0.000635885	3.26623E-06
TG 44:2 FA14:0	TNF-α	5.4087E-06	0.382053204	0.999988801	0.105260817	0.028687104	0.000974075
TG 44:2 FA16:0	IL-17A	-0.00046906	0.246961437	0.998497132	0.001941061	0.004090965	0.638596829
TG 44:2 FA16:0	IL-1β	0.07616823	0.193816785	0.697104989	0.07621248	0.031563775	0.022060861
TG 44:2 FA16:0	IL-6	0.088422621	0.151513839	0.563855568	0.019112169	0.00354325	7.65826E-06
TG 44:2 FA16:0	MCP1	0.041443019	0.137106503	0.764532927	0.003707443	0.000593457	7.00128E-07
TG 44:2 FA16:0	TNF-α	-0.008401613	0.185854106	0.964243147	0.109192003	0.027232371	0.00037186
TG 44:2 FA16:1	IL-17A	0.00998819	0.887782407	0.991097902	0.001942909	0.004088678	0.638091387
TG 44:2 FA16:1	IL-1β	0.374700316	0.68012648	0.58576333	0.075983588	0.030794063	0.019534003
TG 44:2 FA16:1	IL-6	0.329895278	0.519260868	0.530038181	0.018943662	0.003376096	4.15224E-06
TG 44:2 FA16:1	MCP1	0.15701729	0.498844075	0.755121014	0.003671204	0.000600311	1.01E-06
TG 44:2 FA16:1	TNF-α	0.00972713	0.683543884	0.988740353	0.108797343	0.027845785	0.000492544

TG 44:2 FA18:1	IL-17A	0.006964708	0.423326951	0.986982477	0.001944404	0.004092187	0.638121166
TG 44:2 FA18:1	IL-1β	0.196190029	0.328590503	0.554943543	0.074078897	0.031227382	0.024292376
TG 44:2 FA18:1	IL-6	0.164931681	0.260879929	0.532036761	0.019011261	0.00356019	8.91936E-06
TG 44:2 FA18:1	MCP1	0.126116816	0.239044094	0.601665853	0.003677387	0.000603799	1.08319E-06
TG 44:2 FA18:1	TNF-α	0.017238946	0.319210766	0.957289299	0.108731427	0.027294419	0.000399387
TG 44:2 FA18:2	IL-17A	-0.000182654	0.191633763	0.999245814	0.00194121	0.004096202	0.638998786
TG 44:2 FA18:2	IL-1β	0.069785901	0.149325927	0.643633877	0.07442391	0.031379467	0.024320069
TG 44:2 FA18:2	IL-6	0.073003206	0.117833976	0.540235948	0.018989034	0.003555763	8.90936E-06
TG 44:2 FA18:2	MCP1	0.035327013	0.108211332	0.746340646	0.003705816	0.00060439	9.65963E-07
TG 44:2 FA18:2	TNF-α	-0.003545852	0.144957345	0.980646604	0.109258446	0.027407304	0.000396304
TG 44:3 FA18:2	IL-17A	0.016989076	0.746878928	0.982008072	0.001945183	0.003050895	0.528750656
TG 44:3 FA18:2	IL-1β	0.436630082	0.753478214	0.566736156	0.054886394	0.033511139	0.112258662
TG 44:3 FA18:2	IL-6	0.395609683	0.545549225	0.474165992	0.019316263	0.003153401	1.12937E-06
TG 44:3 FA18:2	MCP1	0.29690958	0.54430013	0.589588952	0.003719146	0.000581685	5.44017E-07
TG 44:3 FA18:2	TNF-α	0.010911274	0.72107898	0.9880306	0.109202203	0.026052146	0.000237191
TG 45:0 FA14:0	IL-17A	-0.006684902	0.837959837	0.993687685	0.001943241	0.004090623	0.638193423
TG 45:0 FA14:0	IL-1β	1.92093E-07	0.703521714	0.999999784	0.072463768	0.033763287	0.040063642
TG 45:0 FA14:0	IL-6	0.013831128	0.558325969	0.980400478	0.018931508	0.003847751	2.91867E-05
TG 45:0 FA14:0	MCP1	-0.018884002	0.480397931	0.968904399	0.003703844	0.000612777	1.23175E-06
TG 45:0 FA14:0	TNF-α	-0.07953361	0.629200028	0.900255262	0.108106226	0.027168878	0.000404468
TG 45:0 FA15:0	IL-17A	-0.007760956	1.068484976	0.994252692	0.001943306	0.004089971	0.638128896
TG 45:0 FA15:0	IL-1β	0.064766454	0.890363281	0.942494566	0.072518188	0.033505801	0.038524287
TG 45:0 FA15:0	IL-6	0.0183068	0.711188448	0.979634278	0.018931751	0.003843168	2.87023E-05
TG 45:0 FA15:0	MCP1	-0.022706367	0.612364236	0.970666991	0.003707483	0.000612486	1.20184E-06
TG 45:0 FA15:0	TNF-α	-0.103347203	0.808154255	0.899096904	0.108041448	0.027362941	0.000439867
TG 45:0 FA16:0	IL-17A	-0.005150556	0.657787026	0.993804364	0.001944414	0.004091425	0.638056927
TG 45:0 FA16:0	IL-1β	0.046164305	0.547456741	0.933358225	0.072844188	0.033476502	0.037563983
TG 45:0 FA16:0	IL-6	0.056384816	0.416389915	0.893189475	0.018866542	0.003656305	1.48266E-05
TG 45:0 FA16:0	MCP1	-6.1853E-07	0.371316213	0.999998682	0.003708511	0.000603487	9.29962E-07
TG 45:0 FA16:0	TNF-α	-0.047622632	0.497447915	0.924368557	0.108046712	0.027368666	0.000440631
TG 45:1 FA15:0	IL-17A	-0.010955101	1.300204034	0.993333143	0.001943727	0.004090738	0.638119078
TG 45:1 FA15:0	IL-1β	0.004426306	1.07793573	0.996750857	0.072467459	0.033341414	0.037767133
TG 45:1 FA15:0	IL-6	0.411063884	0.788255876	0.605857558	0.018783371	0.003501146	8.3125E-06
TG 45:1 FA15:0	MCP1	0.228409479	0.733476746	0.757645335	0.003699699	0.000602992	9.55071E-07
TG 45:1 FA15:0	TNF-α	-0.025466951	0.985156224	0.979547638	0.109346537	0.027416499	0.000394268
TG 45:1 FA16:0	IL-17A	-0.00143301	1.297017637	0.999125772	0.00194117	0.00408888	0.638407644
TG 45:1 FA16:0	IL-1β	0.241748288	1.062619432	0.821576591	0.074540426	0.032933452	0.031008591
TG 45:1 FA16:0	IL-6	0.425124925	0.770024896	0.584974914	0.018835824	0.003427016	5.73852E-06
TG 45:1 FA16:0	MCP1	0.218796451	0.728864978	0.766104172	0.003667708	0.0006004	1.02912E-06
TG 45:1 FA16:0	TNF-α	-0.02625869	0.982946631	0.978864557	0.109191747	0.027409757	0.000399353
TG 45:1 FA18:1	IL-17A	0.009897673	0.901205544	0.991309959	0.001937257	0.004087898	0.639001862
TG 45:1 FA18:1	IL-1β	0.329857131	0.712297493	0.646642527	0.074246796	0.031764251	0.026280101
TG 45:1 FA18:1	IL-6	0.31170276	0.549224954	0.574575726	0.018780651	0.003517057	8.92137E-06
TG 45:1 FA18:1	MCP1	0.26282853	0.497738106	0.601352006	0.003684282	0.000589946	7.04168E-07
TG 45:1 FA18:1	TNF-α	-1.14809E-09	0.683650507	0.999999999	0.109289433	0.027430081	0.000398685
TG 46:0 FA12:0	IL-17A	0.089937202	0.465000174	0.847938903	0.001576733	0.004277281	0.714992411
TG 46:0 FA12:0	IL-1β	0.287983357	0.322555435	0.379060738	0.072980836	0.029168898	0.018031235
TG 46:0 FA12:0	IL-6	0.238493467	0.249080637	0.345970664	0.018665989	0.003234503	2.6494E-06
TG 46:0 FA12:0	MCP1	0.165217401	0.252002052	0.517065827	0.003583155	0.000605694	1.76476E-06
TG 46:0 FA12:0	TNF-α	0.111705706	0.337366616	0.742862606	0.106163086	0.027449441	0.000548726

TG 46:0 FA14:0	IL-17A	0.000566032	0.067271019	0.993342218	0.00193336	0.004107899	0.641298621
TG 46:0 FA14:0	IL-1β	0.033076565	0.054279336	0.546862498	0.080102652	0.032585712	0.019956119
TG 46:0 FA14:0	IL-6	0.006655257	0.042400877	0.876327687	0.018960416	0.00365527	1.37324E-05
TG 46:0 FA14:0	MCP1	0.010426996	0.038090961	0.786160835	0.003658051	0.000607783	1.32336E-06
TG 46:0 FA14:0	TNF-α	-3.66566E-11	0.051024877	0.999999999	0.109289587	0.02756071	0.000419887
TG 46:0 FA16:0	IL-17A	0.000322365	0.032197732	0.992077971	0.001930699	0.004120822	0.642797409
TG 46:0 FA16:0	IL-1β	0.016022352	0.026214067	0.545663127	0.08025118	0.032983293	0.021143893
TG 46:0 FA16:0	IL-6	0.00481403	0.019596052	0.807615848	0.018878292	0.003540626	9.12438E-06
TG 46:0 FA16:0	MCP1	0.005091911	0.018217055	0.781768957	0.003657558	0.000609217	1.3799E-06
TG 46:0 FA16:0	TNF-α	1.1386E-12	0.024381453	1	0.109252385	0.027601643	0.000428295
TG 46:0 FA18:0	IL-17A	0.005505098	0.27861845	0.98436681	0.001924172	0.004258832	0.65465745
TG 46:0 FA18:0	IL-1β	0.167799642	0.193210172	0.392025204	0.072997483	0.029034319	0.017527475
TG 46:0 FA18:0	IL-6	0.134728966	0.149466322	0.374555665	0.018794729	0.00322535	2.26218E-06
TG 46:0 FA18:0	MCP1	0.074992728	0.154890203	0.631783254	0.003616563	0.000618642	2.1461E-06
TG 46:0 FA18:0	TNF-α	0.054803283	0.201102545	0.787093733	0.106381702	0.027190361	0.000485404
TG 46:1 FA12:0	IL-17A	0.000952604	0.05898791	0.987222333	0.001926294	0.004155081	0.646280846
TG 46:1 FA12:0	IL-1β	0.024382672	0.044142035	0.584788142	0.073240652	0.030568197	0.023016607
TG 46:1 FA12:0	IL-6	0.021318786	0.03576091	0.55554835	0.018849473	0.003556133	9.96891E-06
TG 46:1 FA12:0	MCP1	0.017809219	0.032849173	0.591719021	0.003648589	0.000604611	1.26576E-06
TG 46:1 FA12:0	TNF-α	0.002286176	0.043958305	0.958867211	0.108580228	0.027388873	0.000421064
TG 46:1 FA14:0	IL-17A	0.000688013	0.057775517	0.990577548	0.001937716	0.004091355	0.639204948
TG 46:1 FA14:0	IL-1β	0.023596724	0.044144737	0.596913956	0.076426312	0.030732876	0.018679753
TG 46:1 FA14:0	IL-6	0.019607472	0.034255554	0.571324058	0.018875965	0.003424579	5.4911E-06
TG 46:1 FA14:0	MCP1	0.008260293	0.032269026	0.799711797	0.003655904	0.000597096	9.89647E-07
TG 46:1 FA14:0	TNF-α	-1.20243E-11	0.043902501	1	0.109289543	0.027499784	0.000409888
TG 46:1 FA14:1	IL-17A	-0.000504479	0.47437476	0.999158521	0.001941209	0.004088936	0.638405424
TG 46:1 FA14:1	IL-1β	0.157405315	0.382101354	0.683307324	0.078365969	0.032379362	0.021775545
TG 46:1 FA14:1	IL-6	0.008070956	0.314558995	0.979700038	0.018935252	0.003827751	2.70693E-05
TG 46:1 FA14:1	MCP1	-0.009005037	0.271472739	0.973757899	0.00371453	0.000611434	1.13044E-06
TG 46:1 FA14:1	TNF-α	-0.047065186	0.361165343	0.897187278	0.107738435	0.027536677	0.000485318
TG 46:1 FA16:0	IL-17A	0.000483233	0.033520533	0.988593525	0.0019305	0.004136163	0.64406013
TG 46:1 FA16:0	IL-1β	0.014331541	0.025275178	0.574918871	0.073878305	0.030660694	0.022314983
TG 46:1 FA16:0	IL-6	0.010980343	0.019543665	0.578401239	0.018871435	0.00340444	5.02782E-06
TG 46:1 FA16:0	MCP1	0.006096004	0.018534614	0.744517565	0.003662562	0.000597593	9.73098E-07
TG 46:1 FA16:0	TNF-α	0.000373949	0.025641374	0.988460778	0.10894898	0.027986256	0.000512025
TG 46:1 FA16:1	IL-17A	0.001805635	0.142236227	0.989955475	0.001938466	0.00409865	0.639670293
TG 46:1 FA16:1	IL-1β	0.071961688	0.109995352	0.517951279	0.08129591	0.031160607	0.014027324
TG 46:1 FA16:1	IL-6	0.037955908	0.084349626	0.655957454	0.0188846	0.003431363	5.62238E-06
TG 46:1 FA16:1	MCP1	-0.001280475	0.081356943	0.987546844	0.003712591	0.000612576	1.1771E-06
TG 46:1 FA16:1	TNF-α	-5.45227E-12	0.108365901	1	0.109282905	0.027621036	0.000430265
TG 46:1 FA18:0	IL-17A	0.032563614	0.8147535	0.968383885	0.001941735	0.004074227	0.63710746
TG 46:1 FA18:0	IL-1β	0.445080088	0.618869469	0.477593664	0.073218062	0.03042419	0.022466895
TG 46:1 FA18:0	IL-6	0.325499656	0.488207924	0.510044424	0.018858915	0.003446487	6.14639E-06
TG 46:1 FA18:0	MCP1	0.260833934	0.457660127	0.572969328	0.003647564	0.000597994	1.05561E-06
TG 46:1 FA18:0	TNF-α	0.179951462	0.604011988	0.7678125	0.107426045	0.026716606	0.000360473
TG 46:1 FA18:1	IL-17A	0.000598913	0.036537234	0.987030304	0.00192924	0.00413152	0.64390478
TG 46:1 FA18:1	IL-1β	0.01518752	0.02774326	0.588132564	0.073504645	0.030841297	0.023689135
TG 46:1 FA18:1	IL-6	0.013289236	0.021987376	0.550119863	0.01885221	0.003509946	8.16917E-06
TG 46:1 FA18:1	MCP1	0.010880838	0.020369988	0.597167141	0.003653094	0.000601866	1.14792E-06
TG 46:1 FA18:1	TNF-α	0.001021364	0.027759279	0.970893323	0.10878476	0.02776509	0.000478074

TG 46:2 FA12:0	IL-17A	-9.12446E-05	0.094553348	0.999236425	0.001941909	0.004140433	0.64245232
TG 46:2 FA12:0	IL-1β	0.027480845	0.072176483	0.706075243	0.073107446	0.031071737	0.025381591
TG 46:2 FA12:0	IL-6	0.03263561	0.057317435	0.573334795	0.018894625	0.003543304	9.10946E-06
TG 46:2 FA12:0	MCP1	0.016779664	0.052211249	0.750150701	0.003684398	0.000597404	8.74177E-07
TG 46:2 FA12:0	TNF-α	-0.001769616	0.07080422	0.980225985	0.109278815	0.027424878	0.000398281
TG 46:2 FA14:0	IL-17A	-0.000201491	0.151108778	0.998944914	0.001941142	0.004088484	0.638380013
TG 46:2 FA14:0	IL-1β	0.015772659	0.122655502	0.898537797	0.073370247	0.032625762	0.032021035
TG 46:2 FA14:0	IL-6	0.049845484	0.091021252	0.588001199	0.018972955	0.003476709	6.40772E-06
TG 46:2 FA14:0	MCP1	0.003333506	0.085033862	0.968988986	0.003711789	0.000601173	8.57563E-07
TG 46:2 FA14:0	TNF-α	-0.003233643	0.114411927	0.97763944	0.109150378	0.027381717	0.000396546
TG 46:2 FA14:1	IL-17A	0.002560843	0.479075912	0.995770412	0.001938404	0.004088875	0.638883768
TG 46:2 FA14:1	IL-1β	0.179490247	0.365143084	0.62660686	0.079166015	0.030638221	0.014883155
TG 46:2 FA14:1	IL-6	0.163299721	0.280758233	0.565155681	0.018869301	0.003382867	4.55932E-06
TG 46:2 FA14:1	MCP1	-0.00819767	0.270167819	0.975994565	0.003716523	0.000602515	8.71754E-07
TG 46:2 FA14:1	TNF-α	-0.010117947	0.363707384	0.977990813	0.109273932	0.027457964	0.000403764
TG 46:2 FA16:0	IL-17A	-8.78361E-05	0.089057424	0.999219587	0.001941875	0.00416396	0.644331564
TG 46:2 FA16:0	IL-1β	0.027037458	0.068369248	0.695299986	0.073355455	0.031426657	0.026473222
TG 46:2 FA16:0	IL-6	0.031204046	0.05306562	0.56091474	0.018969529	0.003502697	7.20287E-06
TG 46:2 FA16:0	MCP1	0.015197925	0.048667877	0.756989266	0.003681919	0.000594585	8.15268E-07
TG 46:2 FA16:0	TNF-α	-0.001681953	0.066351105	0.979944229	0.109273874	0.027441086	0.000401062
TG 46:2 FA16:1	IL-17A	0.002410067	0.20109411	0.99051711	0.001941392	0.004091457	0.638580066
TG 46:2 FA16:1	IL-1β	0.082541327	0.150570675	0.587617903	0.076463346	0.030117579	0.016546698
TG 46:2 FA16:1	IL-6	0.077904129	0.116120323	0.507422486	0.01892295	0.003335338	3.48393E-06
TG 46:2 FA16:1	MCP1	0.028576236	0.114060826	0.803881502	0.003637675	0.000606387	1.39845E-06
TG 46:2 FA16:1	TNF-α	0.002316429	0.155590242	0.9882201	0.108773927	0.028001272	0.000523821
TG 46:2 FA18:1	IL-17A	0.001853231	0.11222999	0.986934628	0.001940418	0.004086659	0.638355393
TG 46:2 FA18:1	IL-1β	0.052005798	0.086994037	0.554453887	0.074683564	0.03114225	0.022903203
TG 46:2 FA18:1	IL-6	0.04053313	0.068120315	0.556290324	0.018848004	0.003501782	7.91226E-06
TG 46:2 FA18:1	MCP1	0.032234955	0.063321251	0.614427157	0.003658087	0.000602482	1.14126E-06
TG 46:2 FA18:1	TNF-α	0.001219117	0.085255927	0.988685715	0.109085133	0.027460019	0.000411792
TG 46:2 FA18:2	IL-17A	-7.81455E-05	0.079834036	0.999225472	0.001941738	0.004151012	0.64332624
TG 46:2 FA18:2	IL-1β	0.029568855	0.062051703	0.637156779	0.07462596	0.031719082	0.025389902
TG 46:2 FA18:2	IL-6	0.028104134	0.048257829	0.564666085	0.018923183	0.00354231	8.86693E-06
TG 46:2 FA18:2	MCP1	0.014123638	0.043832674	0.749522946	0.003678326	0.000595523	8.51896E-07
TG 46:2 FA18:2	TNF-α	7.47429E-13	0.059705973	1	0.109289617	0.027459993	0.000403457
TG 46:3 FA12:0	IL-17A	0.004927599	0.525006393	0.992580342	0.001933768	0.00431782	0.657829015
TG 46:3 FA12:0	IL-1β	0.136532729	0.395129379	0.732366729	0.056446394	0.037182962	0.140619736
TG 46:3 FA12:0	IL-6	0.19302297	0.280997816	0.497991258	0.019277724	0.003391207	4.87583E-06
TG 46:3 FA12:0	MCP1	0.09986119	0.257009914	0.700656172	0.003763014	0.000571635	4.62528E-07
TG 46:3 FA12:0	TNF-α	0.005534549	0.333233632	0.986870975	0.114341553	0.025664989	0.000131593
TG 46:3 FA14:0	IL-17A	-0.015682685	0.951138144	0.986957725	0.001941927	0.004309795	0.655639817
TG 46:3 FA14:0	IL-1β	-0.408043536	0.777280864	0.603599641	0.069313445	0.03516933	0.058352625
TG 46:3 FA14:0	IL-6	0.127698907	0.603680649	0.83395029	0.018815383	0.003930016	4.57653E-05
TG 46:3 FA14:0	MCP1	-0.018274336	0.540170713	0.973243964	0.003646087	0.000650199	4.69622E-06
TG 46:3 FA14:0	TNF-α	-0.118622023	0.680490082	0.862826393	0.102901794	0.027524473	0.00080962
TG 46:3 FA14:1	IL-17A	-0.007355146	1.560974096	0.99627166	0.001941635	0.004095455	0.638864568
TG 46:3 FA14:1	IL-1β	-0.162410626	1.294559368	0.900999288	0.072151214	0.033390998	0.038821591
TG 46:3 FA14:1	IL-6	0.504526799	0.935478085	0.593643104	0.019017841	0.003464918	5.86266E-06
TG 46:3 FA14:1	MCP1	0.032294617	0.867589624	0.970553494	0.003714244	0.000594779	7.04904E-07
TG 46:3 FA14:1	TNF-α	-0.060603332	1.17699381	0.959276446	0.108888877	0.027314758	0.00039633

TG 46:3 FA16:0	IL-17A	-0.011089514	0.750534245	0.988309118	0.001947694	0.004103437	0.638473924
TG 46:3 FA16:0	IL-1β	-0.04214504	0.621432083	0.946379543	0.072165639	0.033401955	0.038844879
TG 46:3 FA16:0	IL-6	0.253941771	0.456838461	0.582423611	0.0192154	0.003526085	6.54765E-06
TG 46:3 FA16:0	MCP1	0.09608469	0.420075345	0.820629278	0.003684596	0.000600122	9.44023E-07
TG 46:3 FA16:0	TNF-α	-0.065765094	0.566637267	0.908376797	0.107467598	0.027403062	0.000473264
TG 46:3 FA16:1	IL-17A	0.00267017	0.669068619	0.99684217	0.001938335	0.004090757	0.639049524
TG 46:3 FA16:1	IL-1β	0.241891909	0.519650471	0.644942514	0.073520734	0.031235277	0.025329334
TG 46:3 FA16:1	IL-6	0.256000436	0.39377915	0.520567802	0.019012442	0.003398902	4.3609E-06
TG 46:3 FA16:1	MCP1	0.101137992	0.375198962	0.789345572	0.003652357	0.000599418	1.07489E-06
TG 46:3 FA16:1	TNF-α	-0.003518583	0.507647207	0.994515667	0.109271655	0.027454389	0.000403282
TG 46:3 FA18:1	IL-17A	0.008590571	0.454405353	0.985042001	0.001942226	0.004090926	0.638392967
TG 46:3 FA18:1	IL-1β	0.227908743	0.351991115	0.522242908	0.074082575	0.031153787	0.023979038
TG 46:3 FA18:1	IL-6	0.184212527	0.280932691	0.51700338	0.019172681	0.003570535	8.20125E-06
TG 46:3 FA18:1	MCP1	0.14267714	0.256507522	0.582179203	0.003697611	0.000603411	9.75844E-07
TG 46:3 FA18:1	TNF-α	0.061257165	0.336571465	0.856804272	0.108932429	0.026802307	0.000319906
TG 46:3 FA18:2	IL-17A	0.002902697	0.266426067	0.991379397	0.001936661	0.004088209	0.639129985
TG 46:3 FA18:2	IL-1β	0.110435779	0.207431826	0.598373534	0.076167139	0.031291956	0.021095671
TG 46:3 FA18:2	IL-6	0.101735208	0.161217733	0.532791571	0.019091544	0.003492384	6.23898E-06
TG 46:3 FA18:2	MCP1	0.046108612	0.148835105	0.758857392	0.003691537	0.000596756	8.29943E-07
TG 46:3 FA18:2	TNF-α	2.41421E-07	0.202091845	0.999999055	0.109289605	0.027429751	0.000398625
TG 46:3 FA18:3	IL-17A	0.001521115	0.601548369	0.99800332	0.001945394	0.003635433	0.597490465
TG 46:3 FA18:3	IL-1β	3.13774E-15	0.834898733	1	1.37233E-15	0.059512339	1
TG 46:3 FA18:3	IL-6	0.257107412	0.368051934	0.491539508	0.019992381	0.00347041	6.1526E-06
TG 46:3 FA18:3	MCP1	0.18268225	0.331195611	0.586332878	0.004032031	0.000584493	3.91226E-07
TG 46:3 FA18:3	TNF-α	0.007933328	0.389765935	0.983929191	0.121225905	0.023424906	2.6692E-05
TG 46:4 FA18:2	IL-17A	0.037285558	0.797778382	0.963043423	0.001938145	0.00304942	0.530035277
TG 46:4 FA18:2	IL-1β	0.528451188	0.794824721	0.511389829	0.056404652	0.033078649	0.098854591
TG 46:4 FA18:2	IL-6	0.49473959	0.57009358	0.392616758	0.01943329	0.003083538	6.97447E-07
TG 46:4 FA18:2	MCP1	0.348183852	0.567070042	0.54399905	0.003738079	0.000567079	3.18523E-07
TG 46:4 FA18:2	TNF-α	0.416534543	0.775977603	0.595509479	0.111409393	0.026234197	0.000203974
TG 47:0 FA14:0	IL-17A	-0.00407676	1.01430167	0.996819698	0.001942311	0.00408967	0.638275573
TG 47:0 FA14:0	IL-1β	0.285676246	0.808787414	0.72639689	0.074985667	0.032059483	0.026190208
TG 47:0 FA14:0	IL-6	0.091904047	0.647296809	0.888043992	0.018946656	0.003684491	1.5588E-05
TG 47:0 FA14:0	MCP1	0.141851456	0.571008048	0.80549997	0.003664498	0.000601587	1.08026E-06
TG 47:0 FA14:0	TNF-α	-0.021823251	0.768690063	0.977538979	0.109239772	0.027415056	0.000398278
TG 47:0 FA15:0	IL-17A	-0.003211406	0.506556435	0.994983671	0.001944332	0.004092877	0.638189919
TG 47:0 FA15:0	IL-1β	0.088773158	0.427155789	0.836770931	0.078913416	0.033930334	0.026979721
TG 47:0 FA15:0	IL-6	0.009000589	0.336154389	0.978816382	0.018924615	0.003834357	2.79462E-05
TG 47:0 FA15:0	MCP1	-0.012636848	0.290493935	0.96559023	0.003705933	0.0006133	1.23778E-06
TG 47:0 FA15:0	TNF-α	-0.021429455	0.378009545	0.955167842	0.109186077	0.027015957	0.000340606
TG 47:0 FA16:0	IL-17A	-0.001205733	0.191506318	0.995018194	0.001944436	0.004092864	0.638171008
TG 47:0 FA16:0	IL-1β	0.018886478	0.160061859	0.906858222	0.07298101	0.033630478	0.038047872
TG 47:0 FA16:0	IL-6	-0.000598968	0.127754772	0.996290234	0.018962008	0.003854555	2.92503E-05
TG 47:0 FA16:0	MCP1	-0.004746037	0.109798005	0.965808472	0.003705553	0.00061316	1.23515E-06
TG 47:0 FA16:0	TNF-α	-0.008149568	0.142841602	0.954881142	0.109155403	0.027003226	0.000339885
TG 47:0 FA17:0	IL-17A	-0.005753516	0.934524969	0.995128517	0.001943555	0.004091279	0.638192925
TG 47:0 FA17:0	IL-1β	0.099571314	0.776253536	0.898790052	0.072803567	0.033409716	0.037308949
TG 47:0 FA17:0	IL-6	-0.002873553	0.622874022	0.996349612	0.018964424	0.003849651	2.86867E-05
TG 47:0 FA17:0	MCP1	-0.020902331	0.539189136	0.969333599	0.003708528	0.0006168	1.34629E-06
TG 47:0 FA17:0	TNF-α	-0.039142213	0.697292532	0.955606811	0.109163809	0.02700225	0.000339457

TG 47:1 FA14:0	IL-17A	-0.000471477	0.431366186	0.999135159	0.001942857	0.004088356	0.638073988
TG 47:1 FA14:0	IL-1β	0.138052843	0.337856167	0.685724979	0.07704812	0.031480052	0.020453837
TG 47:1 FA14:0	IL-6	0.143969606	0.263730991	0.589174394	0.018835634	0.003528716	8.97345E-06
TG 47:1 FA14:0	MCP1	0.059592893	0.240793554	0.806218573	0.003665226	0.000596324	9.26781E-07
TG 47:1 FA14:0	TNF-α	-0.008679334	0.326813396	0.97898858	0.109190383	0.027398021	0.00039754
TG 47:1 FA15:0	IL-17A	-0.003361029	0.415245912	0.993595525	0.001943942	0.004089541	0.637984066
TG 47:1 FA15:0	IL-1β	0.032677393	0.343055929	0.924746634	0.073092111	0.033215066	0.035603987
TG 47:1 FA15:0	IL-6	0.140253689	0.254465941	0.585599924	0.018930566	0.003537951	8.65256E-06
TG 47:1 FA15:0	MCP1	-0.007611834	0.235563888	0.974436277	0.003714132	0.000606196	9.78283E-07
TG 47:1 FA15:0	TNF-α	-0.015739786	0.309902229	0.959829907	0.109150787	0.026996697	0.000339131
TG 47:1 FA16:0	IL-17A	-0.000428206	0.367073407	0.999076959	0.001941371	0.004088991	0.638382046
TG 47:1 FA16:0	IL-1β	0.109983199	0.292353959	0.709416593	0.078553505	0.032016453	0.020172521
TG 47:1 FA16:0	IL-6	0.075306726	0.218364611	0.732600615	0.018971305	0.003433985	5.29853E-06
TG 47:1 FA16:0	MCP1	-0.006969041	0.209632416	0.973700135	0.003714337	0.000610178	1.09264E-06
TG 47:1 FA16:0	TNF-α	-0.007944932	0.278141571	0.977401226	0.109244205	0.027406015	0.000396666
TG 47:1 FA16:1	IL-17A	8.31854E-15	0.897985605	1	7.05927E-17	0.004292083	1
TG 47:1 FA16:1	IL-1β	0.058609038	0.703210189	0.934130893	0.072510071	0.03304341	0.036087954
TG 47:1 FA16:1	IL-6	0.227509275	0.524569075	0.6676042	0.018921842	0.003539601	8.77455E-06
TG 47:1 FA16:1	MCP1	-0.016277055	0.487892543	0.973606923	0.003716387	0.000609337	1.05738E-06
TG 47:1 FA16:1	TNF-α	-0.019392413	0.643712148	0.976166168	0.109222621	0.027214926	0.0003681
TG 47:1 FA17:0	IL-17A	0.002564724	1.129491078	0.998203286	0.001939412	0.004089702	0.638777686
TG 47:1 FA17:0	IL-1β	0.342021634	0.883244958	0.70131728	0.073345879	0.031440611	0.026553174
TG 47:1 FA17:0	IL-6	0.360730079	0.687137007	0.603458655	0.018772461	0.003512409	8.80293E-06
TG 47:1 FA17:0	MCP1	0.203569252	0.623785641	0.74642923	0.003695162	0.000590173	6.735E-07
TG 47:1 FA17:0	TNF-α	-5.08188E-10	0.856877646	1	0.109289617	0.027443808	0.000400865
TG 47:1 FA18:1	IL-17A	-0.001724983	0.357228897	0.996179167	0.001942473	0.004089964	0.638271658
TG 47:1 FA18:1	IL-1β	0.057364208	0.291395221	0.845264339	0.073022706	0.032798673	0.033645072
TG 47:1 FA18:1	IL-6	0.115189045	0.217796482	0.600777612	0.018788139	0.003520275	8.99103E-06
TG 47:1 FA18:1	MCP1	0.05822286	0.199710015	0.772647047	0.003663985	0.000597458	9.62957E-07
TG 47:1 FA18:1	TNF-α	-0.006977976	0.270518567	0.979591823	0.109186257	0.02739597	0.000397379
TG 47:2 FA14:0	IL-17A	-0.009457843	1.072301878	0.993021038	0.001943247	0.004092035	0.638307807
TG 47:2 FA14:0	IL-1β	-0.064673956	0.899119706	0.943134784	0.072238586	0.033731894	0.040469923
TG 47:2 FA14:0	IL-6	0.148817241	0.665044516	0.824452898	0.018962395	0.003582827	1.01959E-05
TG 47:2 FA14:0	MCP1	-0.023543141	0.614596027	0.969696938	0.003704961	0.00061284	1.22762E-06
TG 47:2 FA14:0	TNF-α	-0.103621718	0.806660846	0.898644154	0.107482463	0.02722889	0.000441183
TG 47:2 FA15:0	IL-17A	-0.011368578	1.130254911	0.99204127	0.001943302	0.004093468	0.638415545
TG 47:2 FA15:0	IL-1β	-0.141278569	0.937425738	0.881214009	0.071972312	0.033377425	0.039199932
TG 47:2 FA15:0	IL-6	0.094693594	0.734352791	0.898259136	0.018894048	0.003754677	2.12757E-05
TG 47:2 FA15:0	MCP1	-0.198220753	0.66030365	0.766097786	0.003714101	0.000624876	1.63186E-06
TG 47:2 FA15:0	TNF-α	-0.115845561	0.851446378	0.892684823	0.107874787	0.027276523	0.000432218
TG 47:2 FA16:1	IL-1β	-0.000247486	1.536807837	0.999872576	0.072463486	0.033073573	0.036357376
TG 47:2 FA16:1	IL-6	0.494689299	1.117356185	0.661135551	0.018970989	0.003453068	5.77622E-06
TG 47:2 FA16:1	MCP1	0.386788849	1.040040559	0.712582959	0.003647023	0.000594902	9.68782E-07
TG 47:2 FA16:1	TNF-α	0.137263282	1.413459915	0.9232837	0.108525426	0.027369117	0.000420073
TG 47:2 FA18:1	IL-17A	0.001484261	1.097722603	0.99893011	0.001940187	0.004088204	0.638521642
TG 47:2 FA18:1	IL-1β	0.359415318	0.843865014	0.67320961	0.075766778	0.030896827	0.020232309
TG 47:2 FA18:1	IL-6	0.353337679	0.652101304	0.591927522	0.018843094	0.003428529	5.74346E-06
TG 47:2 FA18:1	MCP1	0.180399418	0.609344678	0.769227982	0.003660197	0.000592977	8.61549E-07
TG 47:2 FA18:1	TNF-α	3.85912E-10	0.832850964	1	0.109263401	0.027436199	0.000400701
TG 47:2 FA18:2	IL-17A	-0.006924125	0.781746005	0.992991663	0.00194327	0.004091273	0.638241515

TG 47:2 FA18:2	IL-1β	-0.043703202	0.656464189	0.947362841	0.072230869	0.033775733	0.0407331
TG 47:2 FA18:2	IL-6	0.165586505	0.469699083	0.726900057	0.018947527	0.003470287	6.35782E-06
TG 47:2 FA18:2	MCP1	-0.014282453	0.44392885	0.974547314	0.003713445	0.000607073	1.0059E-06
TG 47:2 FA18:2	TNF-α	-0.042351572	0.590489097	0.943298486	0.107346585	0.027335145	0.000466426
TG 48:0 FA14:0	IL-17A	0.003807292	0.197851658	0.98477454	0.001927826	0.004121572	0.643350358
TG 48:0 FA14:0	IL-1β	0.114236721	0.15575478	0.468983526	0.081036796	0.031898152	0.016482587
TG 48:0 FA14:0	IL-6	0.073371388	0.120336066	0.546637631	0.018685676	0.003538929	1.05635E-05
TG 48:0 FA14:0	MCP1	0.046017119	0.111455406	0.682634484	0.003671038	0.000606679	1.20901E-06
TG 48:0 FA14:0	TNF-α	0.030651377	0.14834476	0.837700606	0.106564051	0.027334561	0.0005043
TG 48:0 FA16:0	IL-17A	0.00032705	0.01245511	0.9792252	0.001924062	0.004149117	0.646189791
TG 48:0 FA16:0	IL-1β	0.009746555	0.009741897	0.325081111	0.085302924	0.031904547	0.012020625
TG 48:0 FA16:0	IL-6	0.005978414	0.007312252	0.420038355	0.018885584	0.003438841	5.81063E-06
TG 48:0 FA16:0	MCP1	0.00299348	0.00699974	0.671955911	0.003650454	0.000609291	1.42855E-06
TG 48:0 FA16:0	TNF-α	0.004030255	0.008897223	0.653823113	0.10704859	0.026216776	0.00030364
TG 48:0 FA18:0	IL-17A	0.001566138	0.117298851	0.98943561	0.001934715	0.004133526	0.643128401
TG 48:0 FA18:0	IL-1β	0.069146855	0.089279776	0.444698914	0.08076354	0.030930077	0.013953246
TG 48:0 FA18:0	IL-6	0.045153983	0.069984697	0.52370302	0.018698333	0.003481632	8.1811E-06
TG 48:0 FA18:0	MCP1	0.02119986	0.064876588	0.74610927	0.003643879	0.000597379	1.05527E-06
TG 48:0 FA18:0	TNF-α	0.013180709	0.087631945	0.881448014	0.106053376	0.027315308	0.000526753
TG 48:1 FA12:0	IL-17A	0.006187085	0.329492668	0.985142848	0.0019242	0.00424669	0.653732839
TG 48:1 FA12:0	IL-1β	0.178689081	0.232651569	0.448458631	0.07007663	0.029478935	0.024021929
TG 48:1 FA12:0	IL-6	0.151179121	0.180120494	0.407927367	0.01863731	0.003277335	3.35645E-06
TG 48:1 FA12:0	MCP1	0.109643093	0.180464185	0.548050151	0.00359398	0.000607758	1.77604E-06
TG 48:1 FA12:0	TNF-α	0.072965041	0.240119889	0.763325191	0.106389128	0.02737472	0.000521261
TG 48:1 FA14:0	IL-17A	0.000227084	0.018165139	0.990108588	0.001934746	0.00410046	0.640457492
TG 48:1 FA14:0	IL-1β	0.009002822	0.013845441	0.5204878	0.080126442	0.030725688	0.01406503
TG 48:1 FA14:0	IL-6	0.006418788	0.010807999	0.557036231	0.018926093	0.003444229	5.75893E-06
TG 48:1 FA14:0	MCP1	0.003183127	0.010121732	0.755329911	0.003655637	0.000597013	9.8853E-07
TG 48:1 FA14:0	TNF-α	0.000222544	0.013808773	0.987248474	0.109023416	0.027571836	0.000433048
TG 48:1 FA14:1	IL-17A	0.020112467	1.546584017	0.989710346	0.001933378	0.004088513	0.639720851
TG 48:1 FA14:1	IL-1β	0.852794993	1.237160982	0.49592182	0.080548565	0.032152818	0.017898742
TG 48:1 FA14:1	IL-6	0.567567313	0.945533991	0.552840022	0.018867757	0.003528758	8.74736E-06
TG 48:1 FA14:1	MCP1	0.322133325	0.871237603	0.714171915	0.003676652	0.000601815	1.02772E-06
TG 48:1 FA14:1	TNF-α	0.019751117	1.178441042	0.986738752	0.10917195	0.027556016	0.000424046
TG 48:1 FA16:0	IL-17A	0.00013464	0.009444127	0.988719712	0.001933956	0.004116179	0.64186493
TG 48:1 FA16:0	IL-1β	0.005555717	0.007470132	0.462828923	0.079510226	0.032008236	0.018798707
TG 48:1 FA16:0	IL-6	0.003653149	0.005513333	0.512643916	0.019062357	0.003392342	4.05842E-06
TG 48:1 FA16:0	MCP1	0.001540157	0.005371844	0.776305843	0.003652086	0.000611774	1.51769E-06
TG 48:1 FA16:0	TNF-α	0.000131829	0.007276133	0.985664671	0.108917576	0.028051096	0.000526371
TG 48:1 FA16:1	IL-17A	0.001213924	0.050425661	0.980953369	0.001928802	0.004154538	0.645810242
TG 48:1 FA16:1	IL-1β	0.037353925	0.037381814	0.325663864	0.085550788	0.03027837	0.008317401
TG 48:1 FA16:1	IL-6	0.0238858	0.028575485	0.409828431	0.018976057	0.003323668	3.1494E-06
TG 48:1 FA16:1	MCP1	0.004032471	0.029053318	0.890539248	0.003666004	0.000625463	2.05602E-06
TG 48:1 FA16:1	TNF-α	0.008605301	0.037621628	0.820628671	0.105798803	0.027417348	0.000561992
TG 48:1 FA18:0	IL-17A	0.002920353	0.155330931	0.985124467	0.001927754	0.004193636	0.649055979
TG 48:1 FA18:0	IL-1β	0.083581553	0.114476159	0.470979039	0.073537681	0.030384268	0.021775009
TG 48:1 FA18:0	IL-6	0.066933797	0.086168149	0.443369887	0.018827368	0.003284221	2.94974E-06
TG 48:1 FA18:0	MCP1	0.048518383	0.085962339	0.576668158	0.003577691	0.000606423	1.84627E-06
TG 48:1 FA18:0	TNF-α	0.020285585	0.114262157	0.860281112	0.107215608	0.027286755	0.000463671
TG 48:1 FA18:1	IL-17A	0.00015325	0.012102312	0.989980581	0.001936958	0.004101802	0.640185842

TG 48:1 FA18:1	IL-1β	0.005603854	0.009241737	0.548834529	0.079981383	0.030793632	0.014417941
TG 48:1 FA18:1	IL-6	0.004400093	0.007238988	0.547872671	0.018877028	0.003463675	6.53842E-06
TG 48:1 FA18:1	MCP1	0.002147458	0.006778488	0.753585749	0.003655766	0.000600308	1.08499E-06
TG 48:1 FA18:1	TNF-α	0.000156255	0.009334591	0.986755377	0.108967809	0.027984552	0.000510754
TG 48:2 FA12:0	IL-17A	0.00141841	0.079368501	0.985859942	0.001928986	0.004139861	0.644613638
TG 48:2 FA12:0	IL-1β	0.033282314	0.060386109	0.58560693	0.070511406	0.030965322	0.030077031
TG 48:2 FA12:0	IL-6	0.030770596	0.047628018	0.523151732	0.018875048	0.003507139	7.92367E-06
TG 48:2 FA12:0	MCP1	0.024520201	0.043363766	0.575970221	0.003635947	0.000591016	9.1229E-07
TG 48:2 FA12:0	TNF-α	0.009980815	0.058449753	0.86555984	0.10830007	0.02696727	0.00036543
TG 48:2 FA14:0	IL-17A	3.42186E-05	0.028317939	0.999043858	0.001939912	0.004089361	0.638663653
TG 48:2 FA14:0	IL-1β	0.011009961	0.021596008	0.613911666	0.080129537	0.030659724	0.013875803
TG 48:2 FA14:0	IL-6	0.009650554	0.016997456	0.574419778	0.019022862	0.003465218	5.84659E-06
TG 48:2 FA14:0	MCP1	-0.000515671	0.016226755	0.97485874	0.003716335	0.000612294	1.14821E-06
TG 48:2 FA14:0	TNF-α	-0.000593939	0.021454566	0.978097885	0.109166339	0.027405028	0.000399616
TG 48:2 FA14:1	IL-17A	0.000759648	0.125371059	0.995205605	0.001937467	0.00408883	0.639041699
TG 48:2 FA14:1	IL-1β	0.048575536	0.095021468	0.612948916	0.079979994	0.030466633	0.013496958
TG 48:2 FA14:1	IL-6	0.042921784	0.074456721	0.568600455	0.018934964	0.00342814	5.3162E-06
TG 48:2 FA14:1	MCP1	-0.002336027	0.071572966	0.974179139	0.0037151	0.000609938	1.08158E-06
TG 48:2 FA14:1	TNF-α	-0.002594383	0.095106248	0.978418037	0.10915618	0.027436425	0.00040506
TG 48:2 FA16:0	IL-17A	-2.59859E-05	0.021812619	0.999057349	0.001941481	0.004090379	0.63847669
TG 48:2 FA16:0	IL-1β	0.007280612	0.016976825	0.671092013	0.075952777	0.03129776	0.021451941
TG 48:2 FA16:0	IL-6	0.007530005	0.013279162	0.574896149	0.018999213	0.003515437	7.43377E-06
TG 48:2 FA16:0	MCP1	-0.000476205	0.012637591	0.97019119	0.003710004	0.000619234	1.4287E-06
TG 48:2 FA16:0	TNF-α	-0.000478075	0.016530375	0.977119137	0.109294795	0.027419228	0.000396749
TG 48:2 FA16:1	IL-17A	0.000850366	0.042988955	0.984349021	0.001944528	0.004106749	0.639287069
TG 48:2 FA16:1	IL-1β	0.025535807	0.031637671	0.425943093	0.08077876	0.029713015	0.010788763
TG 48:2 FA16:1	IL-6	0.023637616	0.023319452	0.318858673	0.019180642	0.003144941	1.05789E-06
TG 48:2 FA16:1	MCP1	0.000508623	0.025141586	0.983993561	0.003707848	0.00062758	1.80276E-06
TG 48:2 FA16:1	TNF-α	0.003561767	0.032680316	0.913937636	0.107514087	0.027614947	0.000511488
TG 48:2 FA18:0	IL-17A	0.00804382	0.460447251	0.98617767	0.001914712	0.004393632	0.666107137
TG 48:2 FA18:0	IL-1β	0.229304737	0.330069878	0.492579266	0.073235681	0.030963548	0.024681827
TG 48:2 FA18:0	IL-6	0.19456339	0.25139763	0.44503046	0.018871234	0.003386553	4.63052E-06
TG 48:2 FA18:0	MCP1	0.150626498	0.241366156	0.537306458	0.003590421	0.000601804	1.53293E-06
TG 48:2 FA18:0	TNF-α	0.092460729	0.319395032	0.774199983	0.107733921	0.026958066	0.000385693
TG 48:2 FA18:1	IL-17A	0.000306355	0.018875857	0.987158371	0.001935028	0.004101394	0.640484897
TG 48:2 FA18:1	IL-1β	0.00809217	0.014430027	0.579107515	0.074148743	0.030824281	0.022520486
TG 48:2 FA18:1	IL-6	0.007206665	0.011157753	0.523261751	0.018874396	0.003422587	5.44871E-06
TG 48:2 FA18:1	MCP1	0.003300407	0.010650268	0.758787404	0.003651396	0.000604673	1.25161E-06
TG 48:2 FA18:1	TNF-α	0.00046372	0.014563831	0.974810119	0.108531013	0.027990923	0.00053428
TG 48:2 FA18:2	IL-17A	-0.000124659	0.027171258	0.996369759	0.001944307	0.004091152	0.638053138
TG 48:2 FA18:2	IL-1β	0.002755637	0.022584776	0.903702356	0.074483407	0.033431254	0.033529494
TG 48:2 FA18:2	IL-6	0.003538309	0.017047045	0.836974008	0.019021195	0.00362358	1.1522E-05
TG 48:2 FA18:2	MCP1	-0.000596256	0.015680471	0.96991937	0.003708144	0.000616921	1.35311E-06
TG 48:2 FA18:2	TNF-α	-0.001070033	0.02023291	0.958173516	0.109066147	0.026947035	0.000335128
TG 48:3 FA12:0	IL-17A	0.000908267	0.081061226	0.991134307	0.001928783	0.004142621	0.644868056
TG 48:3 FA12:0	IL-1β	0.031978625	0.062058178	0.610119448	0.072365917	0.03117899	0.027269843
TG 48:3 FA12:0	IL-6	0.031679946	0.04799196	0.514220275	0.018869836	0.003462449	6.54115E-06
TG 48:3 FA12:0	MCP1	0.02419081	0.04403181	0.586801845	0.003652258	0.000587981	7.73091E-07
TG 48:3 FA12:0	TNF-α	0.009700546	0.059543543	0.871677706	0.107685054	0.026916181	0.000381054
TG 48:3 FA14:0	IL-17A	-0.000167465	0.13915196	0.999047737	0.001941524	0.004088653	0.638327966

TG 48:3 FA14:0	IL-1β	0.016623146	0.114059875	0.885101087	0.073963656	0.03294768	0.032302382
TG 48:3 FA14:0	IL-6	0.047194487	0.081257758	0.565713415	0.019147668	0.003370611	3.41316E-06
TG 48:3 FA14:0	MCP1	-0.003051589	0.080556442	0.970033223	0.003708994	0.000618481	1.40624E-06
TG 48:3 FA14:0	TNF-α	-0.005691843	0.104361349	0.956866725	0.108962319	0.027123603	0.000364157
TG 48:3 FA14:1	IL-17A	-5.62923E-16	0.250211641	1	3.15568E-17	0.004292254	1
TG 48:3 FA14:1	IL-1β	-0.020948237	0.198986234	0.916858502	0.072062184	0.033558449	0.039965729
TG 48:3 FA14:1	IL-6	0.032550841	0.148734191	0.828247096	0.019031345	0.003601982	1.04585E-05
TG 48:3 FA14:1	MCP1	-0.041763194	0.13816738	0.764535632	0.003723318	0.000619324	1.34866E-06
TG 48:3 FA14:1	TNF-α	-0.025296491	0.184183211	0.891676407	0.10833767	0.027947607	0.000535606
TG 48:3 FA16:0	IL-17A	-0.000158266	0.142882037	0.999124355	0.001941038	0.004314035	0.656348616
TG 48:3 FA16:0	IL-1β	-0.008188041	0.114100195	0.943320376	0.054328714	0.03960295	0.181407117
TG 48:3 FA16:0	IL-6	0.050787288	0.073456833	0.495223155	0.019512901	0.003257829	2.17478E-06
TG 48:3 FA16:0	MCP1	0.000839112	0.070741629	0.990623218	0.003768774	0.00057763	5.37748E-07
TG 48:3 FA16:0	TNF-α	-0.003815922	0.086705361	0.965220136	0.117010052	0.024219803	4.79454E-05
TG 48:3 FA16:1	IL-17A	0.001094013	0.152629747	0.994328458	0.001944413	0.004090197	0.637956772
TG 48:3 FA16:1	IL-1β	0.073344719	0.112269568	0.518544574	0.078653624	0.029577954	0.012445062
TG 48:3 FA16:1	IL-6	0.069325391	0.08410242	0.416276521	0.019069737	0.003181753	1.41997E-06
TG 48:3 FA16:1	MCP1	-0.003997708	0.090571131	0.965086195	0.003707922	0.000634205	2.14252E-06
TG 48:3 FA16:1	TNF-α	1.89843E-11	0.116486966	1	0.10923877	0.027612082	0.000430637
TG 48:3 FA18:1	IL-17A	0.000780067	0.069219036	0.991083006	0.001932272	0.004127743	0.643083349
TG 48:3 FA18:1	IL-1β	0.025893263	0.053066218	0.629137868	0.073759609	0.031110446	0.024366641
TG 48:3 FA18:1	IL-6	0.027307342	0.041297194	0.513505372	0.018972721	0.003476647	6.40716E-06
TG 48:3 FA18:1	MCP1	0.012205164	0.038011469	0.750367242	0.00366897	0.000592293	8.10527E-07
TG 48:3 FA18:1	TNF-α	0.00187593	0.052966908	0.971981828	0.108778468	0.027938846	0.000511322
TG 48:3 FA18:2	IL-17A	-4.36051E-05	0.044366909	0.999222321	0.001941382	0.004100137	0.639289853
TG 48:3 FA18:2	IL-1β	0.014411021	0.034281187	0.677206042	0.074113091	0.031145555	0.023891637
TG 48:3 FA18:2	IL-6	0.017350516	0.02625252	0.513714734	0.019044633	0.00342502	4.78915E-06
TG 48:3 FA18:2	MCP1	0.006950563	0.024639	0.779806932	0.003654184	0.000594973	9.38732E-07
TG 48:3 FA18:2	TNF-α	1.03878E-12	0.033593246	1	0.109288319	0.027460468	0.000403585
TG 48:3 FA18:3	IL-17A	-0.002207286	0.227042459	0.992307527	0.001944563	0.004089378	0.637863824
TG 48:3 FA18:3	IL-1β	-0.122470769	0.187567424	0.518766155	0.071657401	0.033213036	0.039099043
TG 48:3 FA18:3	IL-6	-0.000654532	0.152095056	0.996594854	0.018960092	0.003867392	3.06724E-05
TG 48:3 FA18:3	MCP1	-0.039566579	0.133672735	0.769272542	0.003682047	0.000629112	2.10557E-06
TG 48:3 FA18:3	TNF-α	-0.029726842	0.172578807	0.864397119	0.107398921	0.027495041	0.000493918
TG 48:4 FA12:0	IL-17A	0.002669996	0.167891589	0.987420607	0.001934986	0.003091574	0.536282415
TG 48:4 FA12:0	IL-1β	0.101239774	0.161783335	0.536357609	0.053355144	0.032435905	0.110780062
TG 48:4 FA12:0	IL-6	0.093856441	0.11434738	0.418456	0.019251165	0.002979513	4.53199E-07
TG 48:4 FA12:0	MCP1	0.068592083	0.118792372	0.568117737	0.003687685	0.000572284	4.75019E-07
TG 48:4 FA12:0	TNF-α	0.033503079	0.152169409	0.827281654	0.109863563	0.024783451	0.00012219
TG 48:4 FA14:0	IL-17A	4.40013E-15	1.564770057	1	4.54487E-17	0.005001849	1
TG 48:4 FA14:0	IL-1β	-0.842326052	1.166081785	0.476286229	0.069295426	0.039308199	0.089238901
TG 48:4 FA14:0	IL-6	0.433388453	0.813426008	0.598534446	0.019213901	0.003888995	3.57289E-05
TG 48:4 FA14:0	MCP1	-0.218417175	0.731753172	0.76761834	0.003686743	0.000644102	4.39314E-06
TG 48:4 FA14:0	TNF-α	-0.138451993	0.89645638	0.878408635	0.113943672	0.02681643	0.000228355
TG 48:4 FA14:1	IL-1β	-0.589893992	1.211609676	0.629890223	0.069678208	0.034173524	0.050350297
TG 48:4 FA14:1	IL-6	0.099563822	0.927188046	0.915200427	0.018898949	0.003755318	2.1249E-05
TG 48:4 FA14:1	MCP1	-0.256686652	0.827489183	0.758554094	0.003708779	0.000620331	1.47978E-06
TG 48:4 FA14:1	TNF-α	-0.15687944	1.090650003	0.886588461	0.108122454	0.027677584	0.000493406
TG 48:4 FA16:0	IL-17A	-0.014421393	1.103157295	0.989656236	0.001942262	0.004090898	0.638384476
TG 48:4 FA16:0	IL-1β	-0.936748231	0.885580822	0.298599966	0.071332278	0.03228571	0.03492242

TG 48:4 FA16:0	IL-6	0.046714506	0.730061254	0.949404962	0.018926528	0.003822023	2.66801E-05
TG 48:4 FA16:0	MCP1	-0.239406957	0.648046386	0.714403098	0.003702474	0.000627946	1.86441E-06
TG 48:4 FA16:0	TNF-α	-0.157474988	0.838800597	0.852345879	0.107313771	0.027514183	0.000501828
TG 48:4 FA16:1	IL-17A	-1.60035E-15	1.176520003	1	3.13568E-17	0.004292824	1
TG 48:4 FA16:1	IL-1β	-0.2569789	0.922983292	0.782598515	0.070935495	0.033108433	0.04038547
TG 48:4 FA16:1	IL-6	0.400328654	0.625225139	0.526841319	0.019170375	0.003220573	1.59248E-06
TG 48:4 FA16:1	MCP1	-0.157368381	0.654598917	0.811652126	0.003706874	0.0006241	1.65111E-06
TG 48:4 FA16:1	TNF-α	-0.05514541	0.858404998	0.949203924	0.108575283	0.027704657	0.000476786
TG 48:4 FA18:1	IL-17A	0.007456309	0.448186732	0.98684045	0.00194029	0.003050571	0.529737536
TG 48:4 FA18:1	IL-1β	0.225630144	0.439751696	0.611778347	0.053112317	0.032589006	0.113967562
TG 48:4 FA18:1	IL-6	0.2383013	0.33174971	0.478309857	0.019305565	0.003195221	1.41948E-06
TG 48:4 FA18:1	MCP1	0.175179773	0.326063709	0.595189343	0.003665457	0.000580626	6.77505E-07
TG 48:4 FA18:1	TNF-α	0.088187388	0.410512283	0.831409044	0.110293309	0.024713367	0.000112492
TG 48:4 FA18:2	IL-17A	-0.000159308	0.150972606	0.999165051	0.00194211	0.004136915	0.642136191
TG 48:4 FA18:2	IL-1β	0.054283118	0.117020657	0.646084731	0.07384354	0.031524049	0.025984705
TG 48:4 FA18:2	IL-6	0.063395767	0.088068923	0.477192989	0.019155725	0.003406858	4.01905E-06
TG 48:4 FA18:2	MCP1	0.024896075	0.082539144	0.765017068	0.003645794	0.000590981	8.70037E-07
TG 48:4 FA18:2	TNF-α	0.001759643	0.11509026	0.987902653	0.108997642	0.027895476	0.000492247
TG 48:4 FA18:3	IL-17A	0.003675321	0.242725733	0.988022693	0.001942881	0.003046796	0.528685144
TG 48:4 FA18:3	IL-1β	0.050068556	0.253445778	0.844774283	0.050876425	0.034638102	0.152652047
TG 48:4 FA18:3	IL-6	0.125419084	0.171397771	0.470197918	0.019417616	0.003044395	5.67526E-07
TG 48:4 FA18:3	MCP1	0.052158794	0.186375835	0.781572873	0.003677038	0.000612053	1.55947E-06
TG 48:4 FA18:3	TNF-α	-2.21972E-10	0.233117867	0.999999999	0.109289618	0.02588134	0.000217854
TG 48:4 FA20:4	IL-17A	-0.016517367	1.716605294	0.992413609	0.001971132	0.005534729	0.725289503
TG 48:4 FA20:4	IL-6	0.476196254	0.878472685	0.593474294	0.020198928	0.004870164	0.00045667
TG 48:4 FA20:4	MCP1	0.057268536	0.729583824	0.938177616	0.003870626	0.000726351	2.76871E-05
TG 48:4 FA20:4	TNF-α	-0.070807164	0.670470268	0.916895493	0.11713731	0.021628763	2.26085E-05
TG 48:5 FA18:2	IL-17A	-0.001350004	1.425899651	0.999251545	0.001941326	0.004316736	0.656501651
TG 48:5 FA18:2	IL-1β	0.400018871	1.086321135	0.715571151	0.05807008	0.037805925	0.13617416
TG 48:5 FA18:2	IL-6	0.590136313	0.709000096	0.412513264	0.019796981	0.003152847	1.01751E-06
TG 48:5 FA18:2	MCP1	0.345713757	0.705420557	0.628034864	0.003780728	0.000577542	5.08425E-07
TG 48:5 FA18:2	TNF-α	0.046297355	0.862168271	0.957570542	0.116864005	0.024147779	4.68797E-05
TG 48:5 FA18:3	IL-17A	-0.001119907	1.179962556	0.999249702	0.001941341	0.004316502	0.65648168
TG 48:5 FA18:3	IL-1β	0.256758398	0.882694848	0.773365764	0.055602644	0.036946982	0.143951569
TG 48:5 FA18:3	IL-6	0.47025565	0.591611794	0.433619996	0.01945606	0.003175791	1.51748E-06
TG 48:5 FA18:3	MCP1	0.228247865	0.588054782	0.700955591	0.003783679	0.000581769	5.67471E-07
TG 48:5 FA18:3	TNF-α	0.013179023	0.749021246	0.986091331	0.114290348	0.025659613	0.000131968
TG 49:0 FA15:0	IL-17A	-0.000942668	1.00624484	0.99925873	0.001944197	0.004094499	0.63834569
TG 49:0 FA15:0	IL-1β	0.440547175	0.80526634	0.588368918	0.073708364	0.032213476	0.029348852
TG 49:0 FA15:0	IL-6	0.1687073	0.614847089	0.785666334	0.018800756	0.003531971	9.35616E-06
TG 49:0 FA15:0	MCP1	0.147367606	0.56107825	0.794615151	0.003665229	0.000596562	9.33108E-07
TG 49:0 FA15:0	TNF-α	0.009955369	0.76492373	0.989702112	0.109174711	0.02753163	0.000419869
TG 49:0 FA16:0	IL-17A	0.002206606	0.154005742	0.988663102	0.001929219	0.00409798	0.641207857
TG 49:0 FA16:0	IL-1β	0.081006593	0.123597803	0.517201959	0.080790692	0.032332898	0.018169847
TG 49:0 FA16:0	IL-6	0.042375088	0.09321511	0.652673336	0.018826995	0.003501645	8.0426E-06
TG 49:0 FA16:0	MCP1	0.031031751	0.086716501	0.722959	0.003650571	0.000602934	1.19677E-06
TG 49:0 FA16:0	TNF-α	0.002170494	0.117241005	0.985352108	0.109114677	0.027594921	0.000433049
TG 49:0 FA17:0	IL-17A	0.00476463	0.309405322	0.987815568	0.001929351	0.004095301	0.640967687
TG 49:0 FA17:0	IL-1β	0.182231133	0.252309225	0.47573159	0.080415773	0.03283161	0.020368415
TG 49:0 FA17:0	IL-6	0.075613814	0.186137544	0.687460186	0.01883063	0.003478123	7.23692E-06

TG 49:0 FA17:0	MCP1	0.064136047	0.173021097	0.713478001	0.003641756	0.000598401	1.09717E-06
TG 49:0 FA17:0	TNF-α	0.048896969	0.228157991	0.831753915	0.1074158	0.02671225	0.000360203
TG 49:0 FA18:0	IL-17A	-0.000697956	0.590232667	0.999064322	0.001941355	0.004091024	0.638550994
TG 49:0 FA18:0	IL-1β	0.218146657	0.473561112	0.648370593	0.075846478	0.032269023	0.025520529
TG 49:0 FA18:0	IL-6	0.04243659	0.380362769	0.911908505	0.018871182	0.003721857	1.90983E-05
TG 49:0 FA18:0	MCP1	0.088233652	0.328736951	0.790226982	0.003662995	0.000595377	9.11354E-07
TG 49:0 FA18:0	TNF-α	-4.55061E-12	0.447754724	1	0.109289615	0.027451477	0.000402091
TG 49:1 FA14:0	IL-17A	0.005093956	0.4308259	0.990644553	0.001936321	0.004087167	0.639103433
TG 49:1 FA14:0	IL-1β	0.182878657	0.337931595	0.592386284	0.079705612	0.031517399	0.016933896
TG 49:1 FA14:0	IL-6	0.144046463	0.260075214	0.583776614	0.018837184	0.003483152	7.35914E-06
TG 49:1 FA14:0	MCP1	0.080568789	0.24022335	0.739664185	0.003670901	0.000595485	8.80999E-07
TG 49:1 FA14:0	TNF-α	0.005007535	0.327714681	0.987909835	0.109123899	0.027500033	0.00041676
TG 49:1 FA15:0	IL-17A	-0.00011803	0.107494904	0.999131191	0.001941269	0.004089891	0.638473291
TG 49:1 FA15:0	IL-1β	0.040435476	0.083121569	0.630174689	0.07693484	0.031091279	0.01922006
TG 49:1 FA15:0	IL-6	0.037761688	0.065668512	0.569555615	0.018920074	0.003527231	8.33408E-06
TG 49:1 FA15:0	MCP1	0.015924151	0.059742466	0.791641874	0.00366231	0.000593939	8.77135E-07
TG 49:1 FA15:0	TNF-α	-1.59779E-13	0.081504591	1	0.109288717	0.027429803	0.000398669
TG 49:1 FA16:0	IL-17A	0.000373085	0.058318065	0.994937992	0.001936208	0.004093577	0.639645885
TG 49:1 FA16:0	IL-1β	0.028002051	0.047200138	0.55745238	0.07887342	0.032571973	0.021712441
TG 49:1 FA16:0	IL-6	0.021469182	0.035445767	0.549275366	0.018880231	0.003512506	8.07602E-06
TG 49:1 FA16:0	MCP1	0.008282332	0.032826436	0.802524605	0.003661459	0.000602086	1.11112E-06
TG 49:1 FA16:0	TNF-α	0.000646996	0.044321796	0.988449797	0.109113442	0.02751908	0.00042035
TG 49:1 FA16:1	IL-17A	0.009337371	0.746655053	0.990105029	0.00193409	0.004106119	0.641029208
TG 49:1 FA16:1	IL-1β	0.383527149	0.58476877	0.516912287	0.080217584	0.031615254	0.016605743
TG 49:1 FA16:1	IL-6	0.339665561	0.44554076	0.451793823	0.018958968	0.00345901	5.99043E-06
TG 49:1 FA16:1	MCP1	0.00790535	0.425228633	0.985290621	0.003709782	0.000611039	1.14262E-06
TG 49:1 FA16:1	TNF-α	0.010254718	0.574448288	0.985875582	0.108923518	0.027943397	0.000504995
TG 49:1 FA17:0	IL-17A	0.003898073	0.29330113	0.989484144	0.001936521	0.004089112	0.639227799
TG 49:1 FA17:0	IL-1β	0.142794402	0.233405369	0.545287536	0.080277231	0.031990949	0.017724838
TG 49:1 FA17:0	IL-6	0.109049209	0.17746423	0.543527168	0.018871213	0.003492843	7.46922E-06
TG 49:1 FA17:0	MCP1	0.046205005	0.163715582	0.779707209	0.00365435	0.000596404	9.7731E-07
TG 49:1 FA17:0	TNF-α	0.003816324	0.223393944	0.98648321	0.109014768	0.027548847	0.000429517
TG 49:1 FA18:1	IL-17A	-7.61544E-05	0.065270134	0.999076787	0.001941704	0.004089563	0.638371364
TG 49:1 FA18:1	IL-1β	0.024073766	0.051525439	0.643717678	0.076725918	0.031738406	0.021915407
TG 49:1 FA18:1	IL-6	0.022759348	0.039766817	0.571369555	0.018881295	0.003517516	8.2454E-06
TG 49:1 FA18:1	MCP1	0.009629805	0.036379091	0.79304533	0.003664635	0.000595592	9.10006E-07
TG 49:1 FA18:1	TNF-α	5.93142E-12	0.049492847	1	0.109288263	0.027429734	0.000398676
TG 49:2 FA14:0	IL-17A	0.005614009	0.457528846	0.990291213	0.001941908	0.004088471	0.638246901
TG 49:2 FA14:0	IL-1β	0.198720368	0.349373395	0.573731979	0.079048093	0.030692571	0.015181602
TG 49:2 FA14:0	IL-6	0.167460019	0.274970186	0.54710133	0.018972157	0.003468813	6.19115E-06
TG 49:2 FA14:0	MCP1	0.067678397	0.254065916	0.791767999	0.003652186	0.000593231	9.0116E-07
TG 49:2 FA14:0	TNF-α	0.005480872	0.348059099	0.987540524	0.1090117	0.027511368	0.000423344
TG 49:2 FA15:0	IL-17A	-0.00084399	0.166908851	0.995998915	0.001942964	0.00408845	0.638063181
TG 49:2 FA15:0	IL-1β	0.014520152	0.140123832	0.918157608	0.074817765	0.033743672	0.034328161
TG 49:2 FA15:0	IL-6	0.059384861	0.100073753	0.557354064	0.019212549	0.003460608	4.90744E-06
TG 49:2 FA15:0	MCP1	-0.020216042	0.095683819	0.834097782	0.003738553	0.000612425	1.04144E-06
TG 49:2 FA15:0	TNF-α	-0.007205545	0.124408597	0.954197706	0.109046887	0.026955443	0.000336958
TG 49:2 FA16:0	IL-17A	-3.5388E-15	0.13910282	1	8.27011E-16	0.004288615	1
TG 49:2 FA16:0	IL-1β	0.011166147	0.110322287	0.920054115	0.074482083	0.033438392	0.033567493
TG 49:2 FA16:0	IL-6	0.035594124	0.080580397	0.661858223	0.01910417	0.003507223	6.59227E-06

TG 49:2 FA16:0	MCP1	-0.014855072	0.076862672	0.84805188	0.003736875	0.000619201	1.26439E-06
TG 49:2 FA16:0	TNF-α	-0.005688832	0.098831114	0.954479896	0.109082692	0.026952035	0.000335255
TG 49:2 FA16:1	IL-17A	0.005050896	0.291678018	0.986298633	0.00194391	0.004093875	0.638344121
TG 49:2 FA16:1	IL-1β	0.183910352	0.213189142	0.395166913	0.082491521	0.029416902	0.008762146
TG 49:2 FA16:1	IL-6	0.161335928	0.160986432	0.324276387	0.019238685	0.00318987	1.27794E-06
TG 49:2 FA16:1	MCP1	2.68451E-07	0.168935825	0.999998743	0.003708511	0.000619566	1.45131E-06
TG 49:2 FA16:1	TNF-α	0.053512468	0.213848794	0.804111317	0.107236966	0.026549381	0.000342855
TG 49:2 FA17:0	IL-17A	-0.000837466	0.732867948	0.999095803	0.001941109	0.004090033	0.638512403
TG 49:2 FA17:0	IL-1β	0.111941143	0.596691411	0.85245094	0.073482439	0.032737975	0.032325159
TG 49:2 FA17:0	IL-6	0.262724275	0.44584906	0.560093706	0.019045372	0.003512706	7.07877E-06
TG 49:2 FA17:0	MCP1	-0.013980466	0.415895481	0.973406559	0.003714856	0.000606485	9.83014E-07
TG 49:2 FA17:0	TNF-α	-0.0153576	0.554714353	0.978096262	0.109139716	0.027383388	0.000397235
TG 49:2 FA18:1	IL-17A	0.002508887	0.192854687	0.989706577	0.001940118	0.004087567	0.638481545
TG 49:2 FA18:1	IL-1β	0.091772194	0.145265406	0.532331778	0.077286787	0.030268987	0.015993467
TG 49:2 FA18:1	IL-6	0.088282923	0.111382138	0.434227022	0.019147075	0.003332753	2.84834E-06
TG 49:2 FA18:1	MCP1	0.019999137	0.108570515	0.855092831	0.003664556	0.000601287	1.07084E-06
TG 49:2 FA18:1	TNF-α	0.006804756	0.14670051	0.963310515	0.108140995	0.02750319	0.000460224
TG 49:2 FA18:2	IL-17A	-0.001084836	0.141297071	0.993924976	0.001943348	0.004090269	0.638145974
TG 49:2 FA18:2	IL-1β	-0.008978252	0.12009268	0.940901157	0.072081638	0.03417718	0.043394505
TG 49:2 FA18:2	IL-6	0.011504259	0.092628601	0.901987192	0.018874453	0.003785443	2.42317E-05
TG 49:2 FA18:2	MCP1	-0.022198774	0.081467259	0.787114341	0.003727309	0.000616221	1.21708E-06
TG 49:2 FA18:2	TNF-α	-0.01474698	0.106451429	0.890745343	0.107773754	0.027257557	0.000433351
TG 49:3 FA15:0	IL-17A	1.05327E-14	0.843317261	1	3.42976E-16	0.004545121	1
TG 49:3 FA15:0	IL-1β	-0.158890628	0.683919101	0.817918353	0.066553404	0.036941137	0.082013404
TG 49:3 FA15:0	IL-6	0.057376713	0.524680345	0.913673693	0.018789289	0.004066525	7.27515E-05
TG 49:3 FA15:0	MCP1	-0.220242809	0.465489856	0.639653963	0.003604392	0.000666448	8.15717E-06
TG 49:3 FA15:0	TNF-α	-0.117907802	0.564415321	0.83598476	0.106811555	0.026895155	0.000432343
TG 49:3 FA16:0	IL-17A	-0.010583989	0.979511887	0.991450267	0.001944127	0.004091881	0.638143612
TG 49:3 FA16:0	IL-1β	-0.332948832	0.838239987	0.69403197	0.069207201	0.034425686	0.053460798
TG 49:3 FA16:0	IL-6	0.016396528	0.655728371	0.980216517	0.018942674	0.003867142	3.1037E-05
TG 49:3 FA16:0	MCP1	-0.173530025	0.566346845	0.761414779	0.003705189	0.000618202	1.41988E-06
TG 49:3 FA16:0	TNF-α	-0.13015423	0.748191891	0.863066761	0.106990355	0.027646679	0.000545249
TG 49:3 FA16:1	IL-17A	1.24942E-15	0.845301213	1	3.31683E-17	0.004289345	1
TG 49:3 FA16:1	IL-1β	0.239276774	0.639930673	0.711100608	0.078110485	0.031923714	0.020488529
TG 49:3 FA16:1	IL-6	0.348894769	0.452099246	0.446315997	0.019266937	0.003238666	1.60788E-06
TG 49:3 FA16:1	MCP1	-0.12074154	0.472504032	0.800053472	0.003720799	0.000626498	1.65349E-06
TG 49:3 FA16:1	TNF-α	-5.18019E-11	0.611599649	1	0.109289265	0.027451335	0.000402083
TG 49:3 FA18:2	IL-17A	-0.001307978	0.421253897	0.997543157	0.001941783	0.004090745	0.638454362
TG 49:3 FA18:2	IL-1β	-1.2033E-08	0.349098887	0.999999973	0.072463768	0.033327894	0.037703948
TG 49:3 FA18:2	IL-6	0.1443276	0.25586986	0.576901935	0.019151522	0.003507765	6.36105E-06
TG 49:3 FA18:2	MCP1	-0.056379158	0.244803752	0.819419204	0.003720908	0.000621171	1.43321E-06
TG 49:3 FA18:2	TNF-α	-0.008814863	0.31853483	0.978106109	0.109092031	0.027361008	0.000395583
TG 49:3 FA18:3	IL-17A	-0.00925486	1.227358374	0.994039071	0.001942544	0.004313925	0.656091877
TG 49:3 FA18:3	IL-1β	-0.291143887	0.99436708	0.771918683	0.051413981	0.040177514	0.211548252
TG 49:3 FA18:3	IL-6	0.450438217	0.658574638	0.499829508	0.01962386	0.003400135	3.87123E-06
TG 49:3 FA18:3	MCP1	0.007991678	0.611675868	0.989671826	0.003770066	0.000581422	5.96894E-07
TG 49:3 FA18:3	TNF-α	-0.031238053	0.747177248	0.966959328	0.11703099	0.024296487	4.98358E-05
TG 50:0 FA14:0	IL-17A	0.02681172	1.239399411	0.98288409	0.001932162	0.004131052	0.643367252
TG 50:0 FA14:0	IL-1β	0.801839716	0.872081405	0.365191858	0.074733594	0.028576392	0.013819607
TG 50:0 FA14:0	IL-6	0.592047381	0.69280959	0.399567771	0.018875139	0.003259989	2.51147E-06

TG 50:0 FA14:0	MCP1	0.252570118	0.695067806	0.718873051	0.003629105	0.000605357	1.41396E-06
TG 50:0 FA14:0	TNF-α	0.312847594	0.919492676	0.736047232	0.106410972	0.027109056	0.000468681
TG 50:0 FA16:0	IL-17A	0.001637779	0.023593921	0.945119687	0.001867575	0.004131952	0.654531861
TG 50:0 FA16:0	IL-1β	0.021132117	0.017844964	0.245629335	0.089582555	0.030723576	0.00665423
TG 50:0 FA16:0	IL-6	0.014871663	0.014542229	0.314647874	0.018889253	0.003595332	1.1375E-05
TG 50:0 FA16:0	MCP1	0.010139006	0.013070539	0.443990024	0.003625034	0.000598113	1.17656E-06
TG 50:0 FA16:0	TNF-α	0.015304588	0.01753564	0.389720553	0.105274347	0.027163984	0.000536988
TG 50:0 FA18:0	IL-17A	0.002361725	0.034341479	0.945627625	0.00188475	0.004095225	0.648668907
TG 50:0 FA18:0	IL-1β	0.02957197	0.026150008	0.267074292	0.086463721	0.030657135	0.008422757
TG 50:0 FA18:0	IL-6	0.020964551	0.021074134	0.327790136	0.018848663	0.003547818	9.63154E-06
TG 50:0 FA18:0	MCP1	0.011134292	0.019262637	0.567560899	0.003657687	0.000600218	1.07264E-06
TG 50:0 FA18:0	TNF-α	0.019639339	0.025705182	0.450821457	0.105935702	0.02711416	0.000492697
TG 50:1 FA14:0	IL-17A	0.003571837	0.085057464	0.966782345	0.001922829	0.004100514	0.642514032
TG 50:1 FA14:0	IL-1β	0.056942921	0.064951095	0.387613714	0.08014294	0.030783172	0.014210204
TG 50:1 FA14:0	IL-6	0.036989038	0.050552067	0.470028166	0.018889844	0.00344047	5.83298E-06
TG 50:1 FA14:0	MCP1	0.02870099	0.047299457	0.54855321	0.003648107	0.000595823	9.89653E-07
TG 50:1 FA14:0	TNF-α	0.027699855	0.063291127	0.664769226	0.108052081	0.026988918	0.000378115
TG 50:1 FA16:0	IL-17A	5.98954E-05	0.002198754	0.978448246	0.001920283	0.004160486	0.647731638
TG 50:1 FA16:0	IL-1β	0.001677809	0.001602294	0.303403557	0.087317205	0.029806471	0.006430926
TG 50:1 FA16:0	IL-6	0.001268135	0.001197953	0.298238386	0.019261564	0.003200075	1.32181E-06
TG 50:1 FA16:0	MCP1	0.000527536	0.001208532	0.665592453	0.003645958	0.000597531	1.04961E-06
TG 50:1 FA16:0	TNF-α	0.001178729	0.001610851	0.470003394	0.106516077	0.026961215	0.000437155
TG 50:1 FA16:1	IL-17A	0.005809052	0.120605039	0.961903252	0.001890807	0.004199159	0.655744258
TG 50:1 FA16:1	IL-1β	0.110928837	0.089281635	0.223691619	0.089056766	0.03056047	0.006681471
TG 50:1 FA16:1	IL-6	0.079418557	0.068378922	0.254617759	0.019066187	0.003361027	3.49126E-06
TG 50:1 FA16:1	MCP1	0.031269861	0.066234559	0.640265886	0.003643315	0.000602582	1.22555E-06
TG 50:1 FA16:1	TNF-α	0.063508118	0.088570052	0.478896409	0.1068131	0.027277247	0.000480972
TG 50:1 FA18:0	IL-17A	0.001702559	0.044505187	0.96973758	0.001916051	0.004131968	0.646199855
TG 50:1 FA18:0	IL-1β	0.033912701	0.03342157	0.318365347	0.082336532	0.030505216	0.011309028
TG 50:1 FA18:0	IL-6	0.021660997	0.026082784	0.412833045	0.018871192	0.003418638	5.36591E-06
TG 50:1 FA18:0	MCP1	0.01600258	0.024415281	0.517184073	0.003606255	0.000592302	1.08885E-06
TG 50:1 FA18:0	TNF-α	0.017927931	0.032770762	0.588376823	0.10786648	0.026912222	0.000373452
TG 50:1 FA18:1	IL-17A	8.81541E-05	0.003504837	0.980100186	0.001923736	0.004144329	0.645866062
TG 50:1 FA18:1	IL-1β	0.002665027	0.00257067	0.308157734	0.086913789	0.029883654	0.006776982
TG 50:1 FA18:1	IL-6	0.001993858	0.001912209	0.305418103	0.019272704	0.003192087	1.25506E-06
TG 50:1 FA18:1	MCP1	0.000812788	0.001942029	0.678544176	0.003651294	0.000600036	1.09928E-06
TG 50:1 FA18:1	TNF-α	0.001795136	0.002567111	0.489762173	0.106398608	0.026850214	0.000423055
TG 50:1 FA20:1	IL-17A	0.012564417	0.953065983	0.989568951	0.001933399	0.004093982	0.640162888
TG 50:1 FA20:1	IL-1β	0.528522119	0.736039514	0.47827409	0.080511332	0.031083157	0.014663545
TG 50:1 FA20:1	IL-6	0.353872324	0.572231629	0.540973955	0.01892525	0.003470146	6.46994E-06
TG 50:1 FA20:1	MCP1	0.181710439	0.533571694	0.735812055	0.003664807	0.000598895	9.99428E-07
TG 50:1 FA20:1	TNF-α	0.013159704	0.726156096	0.985661188	0.109140417	0.027591145	0.000431302
TG 50:2 FA14:0	IL-17A	0.000337649	0.014547246	0.98163603	0.001939477	0.004092516	0.638996399
TG 50:2 FA14:0	IL-1β	0.009454611	0.010721621	0.384881634	0.078998271	0.029653165	0.012300688
TG 50:2 FA14:0	IL-6	0.006699612	0.008315465	0.426767061	0.019157539	0.003302547	2.43581E-06
TG 50:2 FA14:0	MCP1	0.002883423	0.008032985	0.722148053	0.003643924	0.000590502	8.65609E-07
TG 50:2 FA14:0	TNF-α	0.004189901	0.010678546	0.697561458	0.107207169	0.02657287	0.000347313
TG 50:2 FA14:1	IL-17A	0.065120181	1.035160614	0.950256818	0.001928281	0.004056351	0.637963961
TG 50:2 FA14:1	IL-1β	0.699129224	0.814318783	0.39739963	0.079216556	0.03137063	0.01708323
TG 50:2 FA14:1	IL-6	0.421559384	0.626286073	0.506029935	0.018883639	0.003464603	6.53016E-06

TG 50:2 FA14:1	MCP1	0.349148789	0.582448972	0.553373831	0.003618517	0.000596377	1.15469E-06
TG 50:2 FA14:1	TNF-α	0.309063337	0.765657247	0.689327466	0.106934928	0.026538657	0.000352182
TG 50:2 FA16:0	IL-17A	4.90618E-05	0.003118875	0.987553467	0.00192877	0.004125713	0.643520528
TG 50:2 FA16:0	IL-1β	0.002097387	0.002295898	0.368244379	0.08529004	0.029857584	0.007704936
TG 50:2 FA16:0	IL-6	0.00161056	0.001704451	0.352251481	0.019282199	0.003183013	1.18625E-06
TG 50:2 FA16:0	MCP1	-7.46266E-05	0.001818135	0.967531406	0.003721504	0.000628437	1.73507E-06
TG 50:2 FA16:0	TNF-α	5.98218E-05	0.002397024	0.98025477	0.108633399	0.028047246	0.000540344
TG 50:2 FA16:1	IL-17A	0.000190977	0.008495326	0.982213743	0.001935169	0.004148443	0.6442411
TG 50:2 FA16:1	IL-1β	0.005609732	0.006154471	0.369304312	0.081831018	0.02954587	0.009534099
TG 50:2 FA16:1	IL-6	0.005343767	0.004495254	0.243860099	0.019221359	0.003098933	7.9257E-07
TG 50:2 FA16:1	MCP1	0.000445792	0.00497749	0.929230877	0.003674752	0.000635112	2.53947E-06
TG 50:2 FA16:1	TNF-α	0.001999599	0.006216175	0.749928292	0.105332338	0.026850045	0.000471649
TG 50:2 FA18:0	IL-17A	0.00275671	0.15311295	0.985754537	0.001931367	0.004102004	0.641164178
TG 50:2 FA18:0	IL-1β	0.079977214	0.120691394	0.512608481	0.078650551	0.03178787	0.019231242
TG 50:2 FA18:0	IL-6	0.060490119	0.092375717	0.517568763	0.01896426	0.003493774	6.95676E-06
TG 50:2 FA18:0	MCP1	0.029198517	0.085039124	0.733726865	0.003673245	0.000595303	8.66833E-07
TG 50:2 FA18:0	TNF-α	0.020418878	0.113489394	0.8584261	0.107107058	0.026894047	0.000400589
TG 50:2 FA18:1	IL-17A	9.30784E-05	0.004041841	0.981779868	0.001937707	0.004116509	0.641248219
TG 50:2 FA18:1	IL-1β	0.002805945	0.002909655	0.342576983	0.08103008	0.029133476	0.009265877
TG 50:2 FA18:1	IL-6	0.002496676	0.002147049	0.254060889	0.019115727	0.003087058	8.15736E-07
TG 50:2 FA18:1	MCP1	0.000756682	0.002313462	0.745880472	0.003601386	0.000615669	2.12465E-06
TG 50:2 FA18:1	TNF-α	0.001114257	0.002992602	0.712259337	0.105932596	0.026959726	0.000463579
TG 50:2 FA18:2	IL-17A	3.94987E-05	0.008970318	0.996515866	0.001936514	0.004105589	0.640569627
TG 50:2 FA18:2	IL-1β	0.0051989	0.006775063	0.448866217	0.084076344	0.030484704	0.009807882
TG 50:2 FA18:2	IL-6	0.003754072	0.005156959	0.472280615	0.019214252	0.003332071	2.68264E-06
TG 50:2 FA18:2	MCP1	-0.000227078	0.005234925	0.965688049	0.003719168	0.000626056	1.64613E-06
TG 50:2 FA18:2	TNF-α	-4.09176E-10	0.006781177	0.999999952	0.109289617	0.027452976	0.000402331
TG 50:2 FA20:2	IL-17A	-0.001471944	1.144486001	0.998982341	0.001942807	0.004088047	0.638057239
TG 50:2 FA20:2	IL-1β	0.479143884	0.922007007	0.607103717	0.079052263	0.032377299	0.020734594
TG 50:2 FA20:2	IL-6	0.026020653	0.758778291	0.972870802	0.018938417	0.003826249	2.68585E-05
TG 50:2 FA20:2	MCP1	-0.104418178	0.671944981	0.877549299	0.003729273	0.000627154	1.62003E-06
TG 50:2 FA20:2	TNF-α	-0.055511809	0.871011927	0.94960596	0.108840388	0.027519861	0.000432079
TG 50:3 FA14:0	IL-17A	0.000198001	0.017027267	0.990799032	0.001940413	0.004089325	0.638574376
TG 50:3 FA14:0	IL-1β	0.007751786	0.0128456	0.550739585	0.078685292	0.030329275	0.014519499
TG 50:3 FA14:0	IL-6	0.00769108	0.009691175	0.433651085	0.019074304	0.003285761	2.40656E-06
TG 50:3 FA14:0	MCP1	0.002980963	0.009364908	0.752453684	0.003640695	0.000587686	8.09508E-07
TG 50:3 FA14:0	TNF-α	0.002946539	0.012312856	0.812495808	0.108945952	0.026156652	0.000242093
TG 50:3 FA14:1	IL-17A	0.006517127	0.260901039	0.98023689	0.001949423	0.004084678	0.636640979
TG 50:3 FA14:1	IL-1β	0.158799079	0.195252165	0.422452772	0.078721433	0.030052377	0.013680768
TG 50:3 FA14:1	IL-6	0.124035217	0.145616036	0.40107582	0.01909022	0.00321843	1.68862E-06
TG 50:3 FA14:1	MCP1	0.049421582	0.146602758	0.738379504	0.003632942	0.000599734	1.18711E-06
TG 50:3 FA14:1	TNF-α	0.0689656	0.193511589	0.724042851	0.107283467	0.026798205	0.000378312
TG 50:3 FA16:0	IL-17A	2.02991E-17	0.011537755	1	3.23439E-17	0.004299725	1
TG 50:3 FA16:0	IL-1β	0.00577607	0.008442474	0.499120566	0.082695866	0.030930722	0.012023747
TG 50:3 FA16:0	IL-6	0.00479992	0.006027488	0.432092361	0.019223169	0.00317109	1.17255E-06
TG 50:3 FA16:0	MCP1	-0.001132321	0.006531735	0.863535392	0.003741332	0.000636038	1.93855E-06
TG 50:3 FA16:0	TNF-α	-0.000294806	0.008338185	0.972029956	0.109269328	0.027485749	0.000408435
TG 50:3 FA16:1	IL-17A	0.000158784	0.012363477	0.989838113	0.001935266	0.004100582	0.64037787
TG 50:3 FA16:1	IL-1β	0.007475414	0.009217214	0.423737755	0.080573383	0.030054223	0.011813635
TG 50:3 FA16:1	IL-6	0.007196308	0.006620183	0.28568246	0.019276546	0.003099758	7.5776E-07

TG 50:3 FA16:1	MCP1	-0.001179513	0.007449381	0.875252277	0.003748924	0.000645595	2.3945E-06
TG 50:3 FA16:1	TNF-α	1.84344E-05	0.009452984	0.998456949	0.109209217	0.027732594	0.000452732
TG 50:3 FA18:0	IL-17A	0.014615288	0.725803625	0.984077139	0.001937537	0.003206148	0.550497073
TG 50:3 FA18:0	IL-1β	0.342579297	0.724063142	0.639784992	0.044704438	0.035546811	0.218916805
TG 50:3 FA18:0	IL-6	0.369966864	0.526264474	0.487856757	0.019169978	0.003352695	3.9213E-06
TG 50:3 FA18:0	MCP1	0.168683719	0.521144807	0.748586243	0.003669226	0.000612517	1.87791E-06
TG 50:3 FA18:0	TNF-α	0.010298901	0.650348102	0.987477546	0.109396524	0.025397422	0.000183393
TG 50:3 FA18:1	IL-17A	0.000183015	0.014352334	0.989910396	0.001939756	0.004090027	0.638744882
TG 50:3 FA18:1	IL-1β	0.006542628	0.010687983	0.545051904	0.077985626	0.029943344	0.014177474
TG 50:3 FA18:1	IL-6	0.006560023	0.008041458	0.421058809	0.019213702	0.003235127	1.65327E-06
TG 50:3 FA18:1	MCP1	0.002246943	0.008120599	0.783910544	0.003630068	0.000604681	1.38157E-06
TG 50:3 FA18:1	TNF-α	0.001064858	0.010713619	0.921487468	0.107266721	0.027005767	0.000412374
TG 50:3 FA18:2	IL-17A	5.40798E-05	0.008945986	0.995216719	0.001936952	0.004093828	0.639538183
TG 50:3 FA18:2	IL-1β	0.004226883	0.006721417	0.534195079	0.08008962	0.030238707	0.012764796
TG 50:3 FA18:2	IL-6	0.003946216	0.00497628	0.434001895	0.01925363	0.003214838	1.43786E-06
TG 50:3 FA18:2	MCP1	-0.000200042	0.005230688	0.969746525	0.00371768	0.000625453	1.63082E-06
TG 50:3 FA18:2	TNF-α	-8.00176E-13	0.006788657	1	0.109289496	0.027479065	0.000406533
TG 50:3 FA18:3	IL-1β	-0.010763852	0.067938125	0.875174885	0.07087381	0.032884112	0.039289661
TG 50:3 FA18:3	IL-6	0.001572893	0.055341829	0.977514274	0.018939657	0.003846608	2.88933E-05
TG 50:3 FA18:3	MCP1	-0.015747713	0.048358406	0.74695287	0.003740343	0.000622126	1.34756E-06
TG 50:3 FA18:3	TNF-α	-0.012527743	0.063865278	0.845808528	0.106985029	0.027813338	0.000581134
TG 50:3 FA20:3	IL-1β	0.005681595	0.687355468	0.993461462	0.069445762	0.035743561	0.061794658
TG 50:3 FA20:3	IL-6	-0.039991151	0.554086055	0.942958141	0.018652084	0.00413444	9.83876E-05
TG 50:3 FA20:3	MCP1	-0.192083636	0.491064893	0.698540135	0.003615099	0.000676871	9.83672E-06
TG 50:3 FA20:3	TNF-α	-0.132487748	0.597321286	0.826021661	0.108210269	0.02740275	0.000459553
TG 50:4 FA14:0	IL-17A	1.75699E-11	0.088526668	1	0.001940934	0.004091541	0.638665814
TG 50:4 FA14:0	IL-1β	0.031435543	0.068392614	0.649092876	0.077480199	0.031075847	0.018401508
TG 50:4 FA14:0	IL-6	0.031736072	0.052976213	0.553628411	0.019155477	0.00345658	5.04827E-06
TG 50:4 FA14:0	MCP1	0.012402738	0.049173174	0.802586682	0.003653024	0.000593849	9.13784E-07
TG 50:4 FA14:0	TNF-α	-1.16705E-10	0.067095046	0.999999999	0.109285558	0.027429632	0.000398768
TG 50:4 FA14:1	IL-17A	0.008501268	0.646795167	0.989603171	0.001948148	0.004244317	0.649654949
TG 50:4 FA14:1	IL-1β	0.342233958	0.464604039	0.467274539	0.088065115	0.030819541	0.007822448
TG 50:4 FA14:1	IL-6	0.320999187	0.330003036	0.338738652	0.019930089	0.003106089	5.11539E-07
TG 50:4 FA14:1	MCP1	0.107795008	0.324030569	0.741776876	0.003701451	0.000563599	3.40064E-07
TG 50:4 FA14:1	TNF-α	0.192028771	0.449951143	0.672693534	0.116362706	0.027328814	0.000197826
TG 50:4 FA16:0	IL-17A	-4.2926E-16	0.1672932	1	3.22775E-17	0.004288609	1
TG 50:4 FA16:0	IL-1β	-0.01537833	0.131393509	0.907608202	0.071087328	0.033114108	0.040019678
TG 50:4 FA16:0	IL-6	0.021267892	0.10056883	0.833945966	0.019124138	0.003639607	1.1355E-05
TG 50:4 FA16:0	MCP1	-0.041246174	0.094070642	0.664194508	0.003735289	0.000630126	1.70612E-06
TG 50:4 FA16:0	TNF-α	-0.019137641	0.124409738	0.878775872	0.109206689	0.028210402	0.000543418
TG 50:4 FA16:1	IL-17A	0.000354814	0.09275358	0.996973148	0.00193967	0.004091019	0.638840856
TG 50:4 FA16:1	IL-1β	0.017782858	0.075055481	0.814321929	0.075466636	0.032544995	0.027401269
TG 50:4 FA16:1	IL-6	0.032377449	0.052648218	0.543206833	0.019151385	0.003278215	2.17003E-06
TG 50:4 FA16:1	MCP1	-0.024226613	0.054101869	0.657517317	0.00370665	0.000623517	1.62728E-06
TG 50:4 FA16:1	TNF-α	-0.006167583	0.07135183	0.931691779	0.109442147	0.027837015	0.000460742
TG 50:4 FA18:1	IL-17A	0.00186781	0.191012414	0.99226277	0.001944315	0.004095114	0.638375568
TG 50:4 FA18:1	IL-1β	0.079344582	0.145740908	0.590175114	0.076883815	0.030717569	0.01799279
TG 50:4 FA18:1	IL-6	0.081614394	0.1089864	0.459779528	0.019288583	0.003298599	2.13687E-06
TG 50:4 FA18:1	MCP1	0.027790374	0.106355595	0.795649199	0.003636076	0.000595799	1.04625E-06
TG 50:4 FA18:1	TNF-α	3.26386E-06	0.144716144	0.999982154	0.109289178	0.027443411	0.000400819

TG 50:4 FA18:2	IL-17A	1.96651E-05	0.049524281	0.999685804	0.001940475	0.004091729	0.63876018
TG 50:4 FA18:2	IL-1β	0.016382439	0.039182204	0.678845116	0.077643461	0.03182574	0.02082783
TG 50:4 FA18:2	IL-6	0.019534468	0.029038808	0.506285768	0.019124485	0.00338704	3.75997E-06
TG 50:4 FA18:2	MCP1	0.001609351	0.027713705	0.9540775	0.003702468	0.000598299	8.24611E-07
TG 50:4 FA18:2	TNF-α	2.98999E-11	0.037533224	0.999999999	0.109288377	0.027429714	0.000398668
TG 50:4 FA18:3	IL-17A	-7.11802E-17	0.112379849	1	3.1382E-17	0.004291767	1
TG 50:4 FA18:3	IL-1β	-0.012592955	0.087084985	0.885989581	0.072100798	0.032695797	0.035245396
TG 50:4 FA18:3	IL-6	0.043221383	0.059636458	0.474221989	0.019317947	0.003215232	1.36246E-06
TG 50:4 FA18:3	MCP1	-0.015247938	0.062333727	0.808417826	0.003718667	0.000622022	1.48126E-06
TG 50:4 FA18:3	TNF-α	-0.004809995	0.081575271	0.953371899	0.109014359	0.027556427	0.000430819
TG 50:4 FA20:3	IL-17A	-0.00234936	2.463159918	0.999246259	0.00194534	0.004959033	0.698047094
TG 50:4 FA20:3	IL-1β	0.933137923	1.749532316	0.598311834	0.081921096	0.036982021	0.035715581
TG 50:4 FA20:3	IL-6	0.899308112	1.372044831	0.517935548	0.019256377	0.004269779	0.00012255
TG 50:4 FA20:3	MCP1	-0.045306678	1.208338612	0.970376724	0.003707379	0.000683349	1.09772E-05
TG 50:4 FA20:3	TNF-α	-0.085998005	1.419353884	0.952149442	0.115223537	0.026572533	0.000193699
TG 50:4 FA20:4	IL-17A	-0.009193375	0.390243557	0.981378386	0.001942408	0.004287538	0.654142775
TG 50:4 FA20:4	IL-1β	-0.225118206	0.308417955	0.471729319	0.04542258	0.038953541	0.253783274
TG 50:4 FA20:4	IL-6	-8.25104E-06	0.238373636	0.999972637	0.019230761	0.003846984	3.05443E-05
TG 50:4 FA20:4	MCP1	-0.069924271	0.198183657	0.72695879	0.003782236	0.000588856	6.99522E-07
TG 50:4 FA20:4	TNF-α	-0.058434412	0.233012408	0.803884284	0.114457119	0.023684807	4.7773E-05
TG 50:5 FA14:0	IL-17A	-0.003601996	0.559811675	0.994908804	0.001942109	0.004097099	0.638917184
TG 50:5 FA14:0	IL-1β	-0.210635791	0.463133993	0.652523476	0.072636076	0.033322848	0.037255893
TG 50:5 FA14:0	IL-6	0.182725946	0.338300058	0.593094929	0.019319161	0.00349534	5.26066E-06
TG 50:5 FA14:0	MCP1	-0.010892979	0.316963202	0.972812387	0.00371526	0.000606147	9.71904E-07
TG 50:5 FA14:0	TNF-α	-0.064160345	0.422581171	0.88033777	0.107027579	0.027356551	0.000485614
TG 50:5 FA16:1	IL-17A	0.004744606	0.926500173	0.995947955	0.001946544	0.004097911	0.638220823
TG 50:5 FA16:1	IL-1β	-0.166335918	0.758122897	0.827820619	0.070681543	0.032965348	0.040246713
TG 50:5 FA16:1	IL-6	0.247766989	0.526111632	0.641090117	0.019109201	0.003285094	2.32817E-06
TG 50:5 FA16:1	MCP1	-0.268329319	0.541897862	0.624090551	0.003745269	0.000626281	1.47376E-06
TG 50:5 FA16:1	TNF-α	-0.076359264	0.719590721	0.916197627	0.10892914	0.028152654	0.000546269
TG 50:5 FA18:1	IL-17A	0.008133509	1.786777664	0.996398135	0.001944018	0.004100465	0.638863252
TG 50:5 FA18:1	IL-1β	-0.415879918	1.476702033	0.780162724	0.069463653	0.033316277	0.045681392
TG 50:5 FA18:1	IL-6	0.683900204	1.037295965	0.514725905	0.019415791	0.003360611	2.60104E-06
TG 50:5 FA18:1	MCP1	0.207081565	0.990668537	0.835836054	0.003659624	0.000594054	8.91199E-07
TG 50:5 FA18:1	TNF-α	-0.035323184	1.347092208	0.97925408	0.108960576	0.027344921	0.000398267
TG 50:5 FA18:2	IL-17A	-0.000261457	0.443930711	0.999533978	0.001941008	0.004100074	0.639348992
TG 50:5 FA18:2	IL-1β	-0.051520742	0.368715157	0.889806842	0.070899864	0.03347869	0.042593302
TG 50:5 FA18:2	IL-6	0.157025951	0.265534974	0.558711248	0.019374172	0.003462192	4.33365E-06
TG 50:5 FA18:2	MCP1	0.015176837	0.24793334	0.951595275	0.00370828	0.000598338	8.03522E-07
TG 50:5 FA18:2	TNF-α	-0.022193359	0.33520982	0.947651991	0.108389175	0.027384823	0.000428509
TG 50:5 FA18:3	IL-17A	0.000295742	0.376562725	0.999378563	0.001940479	0.00410343	0.63971283
TG 50:5 FA18:3	IL-1β	-0.092922383	0.306699471	0.763999657	0.070215582	0.032856685	0.040866446
TG 50:5 FA18:3	IL-6	0.150038762	0.215511833	0.491664332	0.019305949	0.003315383	2.28801E-06
TG 50:5 FA18:3	MCP1	-0.009537187	0.215088413	0.9649266	0.003706595	0.000612437	1.20507E-06
TG 50:5 FA18:3	TNF-α	-0.017833101	0.287167288	0.950895062	0.108645628	0.027679703	0.000468926
TG 50:5 FA20:4	IL-17A	-0.006964822	0.827567262	0.993349312	0.001944643	0.004512965	0.670091488
TG 50:5 FA20:4	IL-1β	-0.315957927	0.633199978	0.621985058	0.054263575	0.040163998	0.188320188
TG 50:5 FA20:4	IL-6	0.239229986	0.440525697	0.591717136	0.01933089	0.003615088	1.34702E-05
TG 50:5 FA20:4	MCP1	-0.017035047	0.412678345	0.96738865	0.003760612	0.000620601	2.11148E-06
TG 50:5 FA20:4	TNF-α	-0.036184696	0.508388588	0.943802928	0.114847374	0.025793279	0.000142546

TG 50:5 FA20:5	IL-17A	-0.085675237	1.847328292	0.963326945	0.001944725	0.004231626	0.649252154
TG 50:5 FA20:5	IL-1β	-1.53984119	1.417244808	0.28620522	0.074658524	0.031921736	0.026446296
TG 50:5 FA20:5	IL-6	0.028459548	1.275086136	0.982345799	0.018953928	0.004248344	0.000112932
TG 50:5 FA20:5	MCP1	-0.303041061	1.094677842	0.783873302	0.003789015	0.000680281	5.21507E-06
TG 50:5 FA20:5	TNF-α	-0.328010766	1.42380422	0.819416799	0.104761122	0.02944312	0.001308128
TG 50:6 FA20:4	IL-17A	-0.023173499	2.532338217	0.992771272	0.001948074	0.004765873	0.686199885
TG 50:6 FA20:4	IL-1β	-0.710924451	1.923304057	0.714765649	0.05590925	0.042266248	0.197879285
TG 50:6 FA20:4	IL-6	0.857425376	1.409855405	0.548563642	0.019500151	0.004117861	7.39517E-05
TG 50:6 FA20:4	MCP1	0.370752177	1.230712968	0.765715375	0.003727425	0.00065206	5.91739E-06
TG 50:6 FA20:4	TNF-α	-0.101379431	1.410328328	0.943266565	0.114971903	0.024680849	9.03321E-05
TG 51:0 FA16:0	IL-17A	0.025374574	0.482659221	0.958421091	0.001938784	0.004050216	0.635632164
TG 51:0 FA16:0	IL-1β	0.338703006	0.371106586	0.368685463	0.082404545	0.030615208	0.011514232
TG 51:0 FA16:0	IL-6	0.221331327	0.291235365	0.453201429	0.018806332	0.003450123	6.52154E-06
TG 51:0 FA16:0	MCP1	0.116275674	0.269813907	0.669587164	0.003687122	0.000591612	7.29636E-07
TG 51:0 FA16:0	TNF-α	0.135838166	0.356499601	0.705863772	0.107159876	0.026461432	0.00033307
TG 51:0 FA17:0	IL-17A	0.249121156	0.760329306	0.745452675	0.001771651	0.003506266	0.61705334
TG 51:0 FA17:0	IL-1β	0.722522595	0.70663731	0.314728585	0.082448158	0.032036172	0.015249147
TG 51:0 FA17:0	IL-6	0.39243671	0.540551561	0.473469305	0.018693308	0.003519112	9.65355E-06
TG 51:0 FA17:0	MCP1	0.335853152	0.483873905	0.492964542	0.003662501	0.000583056	6.3641E-07
TG 51:0 FA17:0	TNF-α	0.278682328	0.65800633	0.674933177	0.106099343	0.026840455	0.000434469
TG 51:0 FA18:0	IL-17A	0.026713052	0.630483371	0.966485153	0.001921639	0.004067361	0.640022035
TG 51:0 FA18:0	IL-1β	0.419130737	0.499550989	0.408097032	0.081172373	0.031682533	0.015669068
TG 51:0 FA18:0	IL-6	0.241686613	0.387188361	0.537208421	0.018738309	0.003526259	9.59934E-06
TG 51:0 FA18:0	MCP1	0.215651098	0.351040186	0.543634929	0.003676101	0.00059174	7.71292E-07
TG 51:0 FA18:0	TNF-α	0.162414132	0.463875312	0.728691561	0.10681708	0.026470191	0.000346441
TG 51:1 FA15:0	IL-17A	0.012690685	0.656632268	0.984708274	0.001929738	0.004087853	0.640295176
TG 51:1 FA15:0	IL-1β	0.357541308	0.518813343	0.496023542	0.077370109	0.031753028	0.020972943
TG 51:1 FA15:0	IL-6	0.256596798	0.397214872	0.523197022	0.018876254	0.003491013	7.37963E-06
TG 51:1 FA15:0	MCP1	0.204497988	0.367522239	0.582048552	0.003658037	0.000597849	1.00115E-06
TG 51:1 FA15:0	TNF-α	0.147196794	0.472709252	0.757658031	0.107738233	0.026030581	0.000260322
TG 51:1 FA16:0	IL-17A	0.001476377	0.064073443	0.981769394	0.001930444	0.004093542	0.640636434
TG 51:1 FA16:0	IL-1β	0.047934442	0.048533415	0.331219238	0.084939952	0.030483373	0.009151394
TG 51:1 FA16:0	IL-6	0.032350223	0.038266126	0.404579395	0.018989442	0.003451347	5.64603E-06
TG 51:1 FA16:0	MCP1	0.014964249	0.036062507	0.681128865	0.003662984	0.000602021	1.10129E-06
TG 51:1 FA16:0	TNF-α	0.019912883	0.045746894	0.666472105	0.108785903	0.02585232	0.000214979
TG 51:1 FA17:0	IL-17A	0.001992949	0.086742746	0.981822044	0.001932074	0.004094268	0.640414443
TG 51:1 FA17:0	IL-1β	0.05393983	0.06462789	0.41052809	0.081348655	0.029989141	0.010946882
TG 51:1 FA17:0	IL-6	0.040465406	0.051282712	0.436265814	0.018978528	0.003417173	4.8787E-06
TG 51:1 FA17:0	MCP1	0.019349804	0.048914564	0.695211338	0.003667838	0.000603276	1.11554E-06
TG 51:1 FA17:0	TNF-α	0.024944689	0.061510668	0.687962554	0.108606838	0.0256809	0.000202739
TG 51:1 FA18:0	IL-17A	0.01104939	0.365500918	0.976083199	0.00192603	0.004081824	0.640444267
TG 51:1 FA18:0	IL-1β	0.229241772	0.287203601	0.431036226	0.078769006	0.031532376	0.018198539
TG 51:1 FA18:0	IL-6	0.151502269	0.22309603	0.502286879	0.018866437	0.003517309	8.33685E-06
TG 51:1 FA18:0	MCP1	0.123766356	0.198810304	0.538293765	0.003660188	0.000580149	5.8969E-07
TG 51:1 FA18:0	TNF-α	0.093503008	0.265355323	0.727025005	0.107932748	0.026212616	0.000276135
TG 51:1 FA18:1	IL-17A	0.001570465	0.074667759	0.983358841	0.001932421	0.004089426	0.639960491
TG 51:1 FA18:1	IL-1β	0.048285003	0.057442805	0.407235785	0.080194149	0.030928996	0.014571808
TG 51:1 FA18:1	IL-6	0.033986078	0.044403169	0.450014823	0.018944512	0.003433176	5.39653E-06
TG 51:1 FA18:1	MCP1	0.01559494	0.042044839	0.7133094	0.00367306	0.000601695	1.04142E-06
TG 51:1 FA18:1	TNF-α	0.019626504	0.053446318	0.716033473	0.108053308	0.025891888	0.00023671

TG 51:2 FA15:0	IL-17A	0.002265966	0.110980548	0.983845371	0.001940329	0.004085529	0.638278226
TG 51:2 FA15:0	IL-1β	0.064662824	0.082544935	0.43955368	0.078373085	0.029873961	0.01355189
TG 51:2 FA15:0	IL-6	0.052021813	0.063932068	0.422225959	0.019149068	0.003322561	2.7062E-06
TG 51:2 FA15:0	MCP1	0.02156657	0.060377747	0.723447097	0.003648612	0.000580782	6.35213E-07
TG 51:2 FA15:0	TNF-α	0.031459363	0.079095127	0.693637398	0.10823468	0.025755373	0.000218323
TG 51:2 FA16:0	IL-17A	0.001061255	0.047796064	0.982432409	0.001940028	0.004092229	0.638878092
TG 51:2 FA16:0	IL-1β	0.035339371	0.034333147	0.311562818	0.086512689	0.028898972	0.005477055
TG 51:2 FA16:0	IL-6	0.025452989	0.026396211	0.342621225	0.019164303	0.003190526	1.36868E-06
TG 51:2 FA16:0	MCP1	0.008682369	0.027114297	0.751026033	0.003622949	0.000606598	1.50543E-06
TG 51:2 FA16:0	TNF-α	0.014065063	0.034568127	0.686984922	0.10702815	0.026179441	0.000299444
TG 51:2 FA16:1	IL-17A	0.008999881	0.349024169	0.979598922	0.001942619	0.00408458	0.63780559
TG 51:2 FA16:1	IL-1β	0.248770828	0.250479516	0.328566716	0.082427383	0.028818085	0.007634862
TG 51:2 FA16:1	IL-6	0.223563461	0.189799665	0.248099479	0.019185598	0.003135741	1.00199E-06
TG 51:2 FA16:1	MCP1	0.069882756	0.201031779	0.73055244	0.003610297	0.00061474	1.99012E-06
TG 51:2 FA16:1	TNF-α	0.101321568	0.257593187	0.696850285	0.106282699	0.026665088	0.000396996
TG 51:2 FA17:0	IL-17A	0.001934956	0.1405297	0.989105423	0.001938338	0.004086682	0.638715989
TG 51:2 FA17:0	IL-1β	0.073223572	0.104647934	0.48949682	0.080820864	0.029918122	0.011246451
TG 51:2 FA17:0	IL-6	0.066718092	0.083452674	0.430301567	0.019246853	0.003426067	4.07517E-06
TG 51:2 FA17:0	MCP1	0.022989022	0.077972262	0.770149988	0.003646432	0.000592485	9.06106E-07
TG 51:2 FA17:0	TNF-α	0.020554164	0.103204083	0.843480996	0.106804972	0.026547052	0.000358213
TG 51:2 FA18:1	IL-17A	0.00084475	0.034841933	0.980817565	0.001940555	0.004086878	0.638349749
TG 51:2 FA18:1	IL-1β	0.024445372	0.024753868	0.331277585	0.081652761	0.028545224	0.007631012
TG 51:2 FA18:1	IL-6	0.016758657	0.019522972	0.397474504	0.0191904	0.00323287	1.6675E-06
TG 51:2 FA18:1	MCP1	0.006636548	0.019361057	0.734155578	0.003637175	0.000593408	9.71908E-07
TG 51:2 FA18:1	TNF-α	0.009289072	0.025166731	0.714644643	0.107403105	0.026111567	0.000279474
TG 51:2 FA18:2	IL-17A	0.000369562	0.182031777	0.99839357	0.001939594	0.004088758	0.63866924
TG 51:2 FA18:2	IL-1β	0.08473374	0.142359476	0.55616693	0.078334476	0.031436308	0.018462005
TG 51:2 FA18:2	IL-6	0.072064794	0.110304167	0.518521207	0.019233384	0.003497752	5.69806E-06
TG 51:2 FA18:2	MCP1	0.026930942	0.101263959	0.792098553	0.003656061	0.000594339	9.13678E-07
TG 51:2 FA18:2	TNF-α	0.002177127	0.138525975	0.987564716	0.10917305	0.027522769	0.00041847
TG 51:3 FA15:0	IL-17A	0.000890901	0.126240572	0.994415959	0.001937997	0.004095728	0.639513075
TG 51:3 FA15:0	IL-1β	0.053128986	0.094743013	0.579119297	0.075452759	0.030219017	0.018248184
TG 51:3 FA15:0	IL-6	0.061112835	0.075390213	0.423968044	0.019281489	0.003453027	4.48247E-06
TG 51:3 FA15:0	MCP1	0.02146983	0.06967759	0.760111961	0.003640721	0.000590691	8.83606E-07
TG 51:3 FA15:0	TNF-α	0.022251137	0.091272317	0.809054147	0.109030405	0.026193164	0.00024383
TG 51:3 FA16:1	IL-17A	0.006800409	0.344089426	0.984362903	0.001947989	0.004086188	0.637011961
TG 51:3 FA16:1	IL-1β	0.221435243	0.244263955	0.371873821	0.0815431	0.028517234	0.007650578
TG 51:3 FA16:1	IL-6	0.20199451	0.182424808	0.276974366	0.019191024	0.003058326	6.4809E-07
TG 51:3 FA16:1	MCP1	-0.008807115	0.20234419	0.965571121	0.003724558	0.000627874	1.68634E-06
TG 51:3 FA16:1	TNF-α	0.065312716	0.259189094	0.802768183	0.106688034	0.027225789	0.000477292
TG 51:3 FA17:0	IL-17A	0.005339826	0.573218319	0.992629073	0.001940027	0.004092257	0.63888047
TG 51:3 FA17:0	IL-1β	0.260161821	0.441429866	0.560031273	0.079012615	0.030981718	0.016107772
TG 51:3 FA17:0	IL-6	0.242273057	0.327021697	0.464545859	0.019216839	0.003295887	2.24094E-06
TG 51:3 FA17:0	MCP1	-0.048478279	0.33453076	0.885747783	0.003728457	0.000624042	1.49652E-06
TG 51:3 FA17:0	TNF-α	0.002974295	0.434949068	0.994589171	0.109154622	0.02746616	0.000409941
TG 51:3 FA18:2	IL-17A	0.001026248	0.079922344	0.989840004	0.001940295	0.004091134	0.638742499
TG 51:3 FA18:2	IL-1β	0.037474877	0.059974923	0.536798403	0.07734968	0.030181872	0.015642134
TG 51:3 FA18:2	IL-6	0.037515441	0.046074702	0.421929149	0.019223049	0.003329591	2.63088E-06
TG 51:3 FA18:2	MCP1	0.012544494	0.044980651	0.782245482	0.003633587	0.000601639	1.24866E-06
TG 51:3 FA18:2	TNF-α	0.013847777	0.058888927	0.815689465	0.109232255	0.026664034	0.000292609

TG 51:3 FA18:3	IL-17A	0.001726814	1.37843282	0.999009343	0.001940301	0.003215265	0.551057019
TG 51:3 FA18:3	IL-1β	1.47032E-15	1.891256355	1	1.85254E-15	0.049027682	1
TG 51:3 FA18:3	IL-6	0.672805571	1.030475809	0.519141176	0.019232717	0.003466527	6.21495E-06
TG 51:3 FA18:3	MCP1	0.244324296	1.014297872	0.811403078	0.003654854	0.000629495	3.0882E-06
TG 51:3 FA18:3	TNF-α	-0.055066559	1.226860396	0.964518249	0.109921799	0.025299135	0.000165657
TG 51:4 FA15:0	IL-17A	-0.00042117	0.309710674	0.998923975	0.001940893	0.004106536	0.639894081
TG 51:4 FA15:0	IL-1β	-2.09578E-06	0.255170956	0.999993501	0.0724636	0.033262254	0.037354413
TG 51:4 FA15:0	IL-6	0.115248827	0.181102289	0.529358422	0.019252397	0.003389973	3.42809E-06
TG 51:4 FA15:0	MCP1	-0.00883293	0.176957725	0.960520598	0.003718506	0.000613089	1.16216E-06
TG 51:4 FA15:0	TNF-α	-4.65971E-06	0.235225237	0.999984325	0.109289384	0.027587992	0.000424436
TG 51:4 FA16:1	IL-17A	0.007128085	1.068374506	0.994720806	0.001945048	0.004095661	0.638294251
TG 51:4 FA16:1	IL-1β	0.493878165	0.847545264	0.564439183	0.076494169	0.031942167	0.023079862
TG 51:4 FA16:1	IL-6	0.57251055	0.57091475	0.323980014	0.019252448	0.003089759	7.32242E-07
TG 51:4 FA16:1	MCP1	-0.104470657	0.636025636	0.870631769	0.003745693	0.000637103	1.9549E-06
TG 51:4 FA16:1	TNF-α	1.6685E-08	0.813440302	0.999999984	0.109289617	0.027583089	0.000423608
TG 51:4 FA18:2	IL-17A	-1.85904E-05	0.215089993	0.99993161	0.001940637	0.004103512	0.639692409
TG 51:4 FA18:2	IL-1β	0.048241154	0.174486617	0.784079297	0.077113684	0.032726431	0.025184397
TG 51:4 FA18:2	IL-6	0.08550284	0.126986003	0.505895108	0.019248841	0.003420143	3.95859E-06
TG 51:4 FA18:2	MCP1	-0.00607801	0.123542074	0.961087677	0.003723484	0.000615865	1.22632E-06
TG 51:4 FA18:2	TNF-α	0.003463975	0.163842463	0.983272291	0.109279065	0.027648956	0.00043517
TG 51:4 FA18:3	IL-17A	0.008398132	0.729914518	0.990904628	0.001947762	0.004336202	0.656882354
TG 51:4 FA18:3	IL-1β	0.181227917	0.560065359	0.748746474	0.05788743	0.038248231	0.141780032
TG 51:4 FA18:3	IL-6	0.325990298	0.353306467	0.364344137	0.019961667	0.00308304	6.11841E-07
TG 51:4 FA18:3	MCP1	0.142849846	0.352875853	0.688802049	0.003764514	0.000566928	3.99049E-07
TG 51:4 FA18:3	TNF-α	0.008767537	0.434555684	0.984051444	0.116922425	0.023883699	4.03328E-05
TG 51:4 FA20:4	IL-17A	-0.177293514	1.760153738	0.920540573	0.001961269	0.004465823	0.664164547
TG 51:4 FA20:4	IL-1β	-0.858553528	1.402263341	0.545681836	0.047334292	0.041382607	0.263120104
TG 51:4 FA20:4	IL-6	-0.049468767	1.118162005	0.965050221	0.019138348	0.004269181	0.000131601
TG 51:4 FA20:4	MCP1	-0.369623885	0.897687	0.683896437	0.003761215	0.000628085	2.53681E-06
TG 51:4 FA20:4	TNF-α	-0.18622424	1.070541441	0.863249494	0.114144669	0.025270071	0.000120287
TG 51:5 FA18:2	IL-17A	0.025627983	1.878623185	0.989215984	0.00194234	0.004354538	0.659118042
TG 51:5 FA18:2	IL-1β	0.709888031	1.364375709	0.607093304	0.055623407	0.036355616	0.137654435
TG 51:5 FA18:2	IL-6	1.211232626	0.933426571	0.20539558	0.020601052	0.003178137	6.00153E-07
TG 51:5 FA18:2	MCP1	0.691489945	0.918223018	0.457921974	0.003857101	0.000575598	3.4129E-07
TG 51:5 FA18:2	TNF-α	0.785513403	1.223949691	0.526423962	0.12109496	0.026247299	8.60256E-05
TG 51:5 FA18:3	IL-17A	-0.010379013	2.086663673	0.996069327	0.001943242	0.004603967	0.67643657
TG 51:5 FA18:3	IL-1β	-0.973551963	1.648449086	0.559896519	0.046472813	0.04194306	0.278007867
TG 51:5 FA18:3	IL-6	1.028577426	1.01650412	0.320921377	0.020550012	0.003398464	2.18204E-06
TG 51:5 FA18:3	MCP1	0.264255391	1.007702028	0.795206347	0.003701862	0.000614129	2.29164E-06
TG 51:5 FA18:3	TNF-α	-0.060465778	1.128738749	0.957687787	0.116945922	0.023023824	2.72602E-05
TG 52:0 FA16:0	IL-17A	0.059863854	0.120442321	0.62278862	0.001447809	0.003182672	0.652453609
TG 52:0 FA16:0	IL-1β	0.148383242	0.113940606	0.202728585	0.086462808	0.029600037	0.006567427
TG 52:0 FA16:0	IL-6	0.109248965	0.089011985	0.229234992	0.01900685	0.003320581	3.02293E-06
TG 52:0 FA16:0	MCP1	0.07104932	0.085468474	0.412376103	0.003588678	0.000590138	1.11176E-06
TG 52:0 FA16:0	TNF-α	0.10236577	0.114038746	0.37652511	0.103684216	0.026655198	0.0005164
TG 52:0 FA18:0	IL-17A	0.05050888	0.059424406	0.402075185	0.001227951	0.003117428	0.696441638
TG 52:0 FA18:0	IL-1β	0.079493623	0.056825388	0.172094034	0.088551307	0.029307245	0.005106054
TG 52:0 FA18:0	IL-6	0.053466589	0.044908099	0.243155182	0.018867868	0.0033259	3.48846E-06
TG 52:0 FA18:0	MCP1	0.034743514	0.043372793	0.429405126	0.003588399	0.000594544	1.26244E-06
TG 52:0 FA18:0	TNF-α	0.059501999	0.056367795	0.299576995	0.102347445	0.026156484	0.000484858

TG 52:0 FA20:0	IL-17A	0.024952217	1.077803336	0.981683136	0.001925546	0.004102412	0.642199227
TG 52:0 FA20:0	IL-1β	0.639732366	0.862404211	0.46397611	0.080771677	0.032270937	0.017992925
TG 52:0 FA20:0	IL-6	0.429060561	0.650461149	0.51452674	0.018824371	0.003495216	7.83782E-06
TG 52:0 FA20:0	MCP1	0.374829766	0.582022373	0.52446082	0.003638666	0.000578861	6.28783E-07
TG 52:0 FA20:0	TNF-α	0.281194773	0.776763462	0.719883455	0.107981605	0.02615202	0.00026756
TG 52:1 FA16:0	IL-17A	0.007012925	0.00882368	0.432981872	0.001186438	0.003205094	0.713854569
TG 52:1 FA16:0	IL-1β	0.011383587	0.008446131	0.187817332	0.089532763	0.030161278	0.005833942
TG 52:1 FA16:0	IL-6	0.008297138	0.006619248	0.219709906	0.019021239	0.003394318	4.23808E-06
TG 52:1 FA16:0	MCP1	0.005151097	0.006203946	0.41293174	0.00360217	0.000588835	1.00455E-06
TG 52:1 FA16:0	TNF-α	0.009066454	0.007795064	0.253956518	0.105103243	0.025045366	0.000221924
TG 52:1 FA16:1	IL-17A	0.927240224	1.151114765	0.426861342	0.001086829	0.003157171	0.733066086
TG 52:1 FA16:1	IL-1β	1.512986353	1.121119098	0.187262841	0.082532755	0.030229589	0.010491372
TG 52:1 FA16:1	IL-6	1.138250513	0.807739502	0.169063817	0.018871602	0.003127546	1.26793E-06
TG 52:1 FA16:1	MCP1	0.678701639	0.827511669	0.418587445	0.003574909	0.000593046	1.28918E-06
TG 52:1 FA16:1	TNF-α	1.25897392	1.063933166	0.245974703	0.102175535	0.025811342	0.000427863
TG 52:1 FA18:0	IL-17A	0.00713293	0.008683038	0.417854531	0.001249033	0.003138282	0.693449136
TG 52:1 FA18:0	IL-1β	0.01144745	0.008483569	0.187315228	0.089675114	0.030143925	0.005740671
TG 52:1 FA18:0	IL-6	0.007842155	0.006605232	0.244439617	0.019068787	0.003370243	3.63907E-06
TG 52:1 FA18:0	MCP1	0.00499838	0.006113097	0.420003283	0.003617469	0.00057732	6.64553E-07
TG 52:1 FA18:0	TNF-α	0.008525466	0.008084371	0.300045409	0.106196086	0.025845394	0.000282847
TG 52:1 FA18:1	IL-17A	0.007183515	0.008797753	0.420641244	0.001244497	0.003086109	0.68961836
TG 52:1 FA18:1	IL-1β	0.012078235	0.008784871	0.179347952	0.08972155	0.030295343	0.005935676
TG 52:1 FA18:1	IL-6	0.008325576	0.006775444	0.228703879	0.019059684	0.00335529	3.41587E-06
TG 52:1 FA18:1	MCP1	0.005218367	0.006257579	0.410913024	0.003621963	0.000573562	5.80275E-07
TG 52:1 FA18:1	TNF-α	0.009829752	0.008141548	0.236728089	0.105690382	0.025261736	0.00022988
TG 52:1 FA20:0	IL-17A	0.030750417	1.596257276	0.984757999	0.00193554	0.004116649	0.641631126
TG 52:1 FA20:0	IL-1β	0.870272362	1.220301468	0.481256813	0.077910729	0.030939196	0.017364381
TG 52:1 FA20:0	IL-6	0.654239509	0.922040829	0.483458386	0.018935707	0.003356943	3.81982E-06
TG 52:1 FA20:0	MCP1	0.320517623	0.889298753	0.721061275	0.003644941	0.000599271	1.10803E-06
TG 52:1 FA20:0	TNF-α	0.233218691	1.195530974	0.846649085	0.106906009	0.027272093	0.000475547
TG 52:1 FA20:1	IL-17A	0.021354172	0.319964106	0.94723207	0.001911663	0.004028571	0.638560773
TG 52:1 FA20:1	IL-1β	0.307175271	0.243781641	0.21736791	0.093422114	0.030175308	0.004227558
TG 52:1 FA20:1	IL-6	0.227506883	0.182238634	0.221539568	0.019142644	0.00323924	1.79552E-06
TG 52:1 FA20:1	MCP1	0.095876583	0.179548385	0.597285239	0.003654963	0.000590699	8.26452E-07
TG 52:1 FA20:1	TNF-α	0.247237279	0.236771151	0.304732746	0.108270706	0.026369097	0.000285145
TG 52:2 FA14:0	IL-17A	0.019453585	0.925385966	0.983367203	0.001939577	0.004085675	0.638419883
TG 52:2 FA14:0	IL-1β	0.515537244	0.710863126	0.473932121	0.079227685	0.030855167	0.015461313
TG 52:2 FA14:0	IL-6	0.409307137	0.530371312	0.446307064	0.018983298	0.003305779	2.86975E-06
TG 52:2 FA14:0	MCP1	0.203422887	0.517370385	0.696962284	0.003656245	0.000596867	9.81623E-07
TG 52:2 FA14:0	TNF-α	0.265865654	0.669586467	0.694134401	0.109095604	0.026149557	0.000237539
TG 52:2 FA16:0	IL-17A	6.49741E-05	0.001533661	0.966488149	0.001911086	0.004143934	0.647998059
TG 52:2 FA16:0	IL-1β	0.001292159	0.001036016	0.221959209	0.085182336	0.027520155	0.004235183
TG 52:2 FA16:0	IL-6	0.00124007	0.000800281	0.131737505	0.019534232	0.003052665	4.59573E-07
TG 52:2 FA16:0	MCP1	0.000357483	0.000858941	0.680233288	0.003626455	0.000606431	1.47448E-06
TG 52:2 FA16:0	TNF-α	0.001061974	0.001104648	0.344051804	0.106696573	0.02640122	0.000340785
TG 52:2 FA16:1	IL-17A	0.058516758	0.072851377	0.42815753	0.001248326	0.003088926	0.688984125
TG 52:2 FA16:1	IL-1β	0.103721304	0.069228496	0.14451895	0.087739462	0.028857296	0.00486709
TG 52:2 FA16:1	IL-6	0.081268548	0.05098196	0.121404308	0.019270017	0.003051681	5.80751E-07
TG 52:2 FA16:1	MCP1	0.04224642	0.054419487	0.443643461	0.003654808	0.000602919	1.17305E-06
TG 52:2 FA16:1	TNF-α	0.080824365	0.068241802	0.245560789	0.106075859	0.02559393	0.000256276

TG 52:2 FA18:0	IL-17A	0.000475788	0.01886646	0.980047497	0.001920272	0.004135735	0.645776125
TG 52:2 FA18:0	IL-1β	0.014814443	0.013783014	0.291018338	0.08723645	0.029703491	0.006312783
TG 52:2 FA18:0	IL-6	0.010865577	0.010637522	0.315213433	0.019340999	0.003291969	1.9772E-06
TG 52:2 FA18:0	MCP1	0.00482889	0.010361373	0.644547895	0.003643297	0.000593491	9.46647E-07
TG 52:2 FA18:0	TNF-α	0.010932504	0.014068954	0.443204458	0.10819111	0.027279779	0.000419232
TG 52:2 FA18:1	IL-17A	3.14277E-05	0.001052804	0.976383255	0.001924839	0.004128939	0.644452165
TG 52:2 FA18:1	IL-1β	0.000890782	0.000698252	0.211840222	0.085601917	0.026921788	0.003412762
TG 52:2 FA18:1	IL-6	0.000780788	0.000538474	0.157432794	0.019514752	0.002981316	3.06679E-07
TG 52:2 FA18:1	MCP1	0.000233519	0.00059365	0.696834598	0.003632582	0.000608353	1.51132E-06
TG 52:2 FA18:1	TNF-α	0.000613496	0.000772981	0.433618135	0.107420388	0.026814923	0.000375607
TG 52:2 FA18:2	IL-17A	0.000631493	0.02958056	0.98310921	0.00192226	0.004130182	0.64499337
TG 52:2 FA18:2	IL-1β	0.022147645	0.021808535	0.31796481	0.085904496	0.029935762	0.007460399
TG 52:2 FA18:2	IL-6	0.015955516	0.017220208	0.361550698	0.019421462	0.00339433	3.04182E-06
TG 52:2 FA18:2	MCP1	0.007567142	0.016216728	0.644138668	0.003679643	0.000591645	7.56456E-07
TG 52:2 FA18:2	TNF-α	0.015607149	0.02173652	0.478302692	0.109637387	0.026845382	0.000302952
TG 52:2 FA20:0	IL-17A	0.054089682	2.730034826	0.984323883	0.001935265	0.004100375	0.640361265
TG 52:2 FA20:0	IL-1β	1.50239035	2.041841287	0.467565741	0.075643724	0.030149381	0.017741719
TG 52:2 FA20:0	IL-6	1.214014651	1.572157424	0.446038922	0.019194459	0.00333353	2.74716E-06
TG 52:2 FA20:0	MCP1	0.865799812	1.481332107	0.563271549	0.003660493	0.000581357	6.10632E-07
TG 52:2 FA20:0	TNF-α	0.785374372	1.959078516	0.691342371	0.109142315	0.026026994	0.000223832
TG 52:2 FA20:1	IL-17A	0.006871654	0.399557193	0.986392392	0.001942839	0.00409409	0.638546023
TG 52:2 FA20:1	IL-1β	0.271932119	0.296399711	0.366223773	0.085126275	0.029857757	0.007810139
TG 52:2 FA20:1	IL-6	0.218770826	0.217835104	0.323268381	0.019134869	0.003151078	1.13876E-06
TG 52:2 FA20:1	MCP1	0.054951055	0.225806777	0.809387301	0.00364558	0.000604575	1.28221E-06
TG 52:2 FA20:1	TNF-α	0.080251843	0.294657681	0.787214004	0.106831028	0.02670625	0.000381609
TG 52:2 FA20:2	IL-17A	0.006007641	0.429162868	0.988923851	0.001935457	0.004096594	0.640020881
TG 52:2 FA20:2	IL-1β	0.321946255	0.34776375	0.361957744	0.089342746	0.032635185	0.010304561
TG 52:2 FA20:2	IL-6	0.176687464	0.245450326	0.477188411	0.019176647	0.003307633	2.45738E-06
TG 52:2 FA20:2	MCP1	-0.014706733	0.256771183	0.954705421	0.003716004	0.000640445	2.42637E-06
TG 52:2 FA20:2	TNF-α	0.006465399	0.330113554	0.984503812	0.108962089	0.027872818	0.000489695
TG 52:3 FA14:0	IL-17A	0.008807762	0.775588417	0.991014417	0.001939351	0.004088314	0.638674797
TG 52:3 FA14:0	IL-1β	0.436476988	0.596764041	0.470205918	0.079372127	0.030925487	0.015503871
TG 52:3 FA14:0	IL-6	0.322864117	0.442917946	0.471686356	0.019064488	0.003296014	2.55296E-06
TG 52:3 FA14:0	MCP1	0.102904463	0.440868681	0.817026131	0.003645011	0.000607235	1.38405E-06
TG 52:3 FA14:0	TNF-α	0.170161797	0.558153159	0.762573442	0.108003096	0.026024539	0.00025242
TG 52:3 FA16:0	IL-17A	2.19495E-05	0.001754616	0.990101888	0.001933342	0.004102398	0.640856356
TG 52:3 FA16:0	IL-1β	0.001121664	0.001298168	0.394424279	0.082538798	0.029839232	0.009615874
TG 52:3 FA16:0	IL-6	0.000988045	0.000961909	0.312551256	0.019478955	0.003174992	9.56275E-07
TG 52:3 FA16:0	MCP1	0.000172887	0.000980298	0.861195042	0.003642343	0.000598892	1.10954E-06
TG 52:3 FA16:0	TNF-α	0.000433009	0.001249983	0.731451117	0.108665523	0.025850945	0.000217639
TG 52:3 FA16:1	IL-17A	0.000598714	0.013907274	0.965946602	0.001927937	0.00408603	0.640457566
TG 52:3 FA16:1	IL-1β	0.011604074	0.009909864	0.25083132	0.077611134	0.028623857	0.010978333
TG 52:3 FA16:1	IL-6	0.010520023	0.007495413	0.170726284	0.018995201	0.003108909	1.02583E-06
TG 52:3 FA16:1	MCP1	0.001473767	0.007972348	0.854582996	0.003637524	0.000612041	1.63405E-06
TG 52:3 FA16:1	TNF-α	0.006727497	0.010417305	0.523318248	0.106710588	0.027072701	0.000448174
TG 52:3 FA18:0	IL-17A	0.002906017	0.124600984	0.981547396	0.001933192	0.004107878	0.641325846
TG 52:3 FA18:0	IL-1β	0.10255493	0.090805197	0.267681308	0.088248977	0.029431186	0.005410539
TG 52:3 FA18:0	IL-6	0.065925597	0.067602481	0.337261998	0.019294587	0.003146383	9.63799E-07
TG 52:3 FA18:0	MCP1	0.02533843	0.069720426	0.718832929	0.003645617	0.000600608	1.14707E-06
TG 52:3 FA18:0	TNF-α	0.044881688	0.092214821	0.630002323	0.108184799	0.026891408	0.000358418

TG 52:3 FA18:1	IL-17A	2.87561E-05	0.0015344	0.985171828	0.001939576	0.004095447	0.639218348
TG 52:3 FA18:1	IL-1β	0.001115438	0.001098118	0.317860252	0.083609798	0.028814608	0.006891696
TG 52:3 FA18:1	IL-6	0.00103796	0.000846232	0.229525499	0.019636809	0.003188638	8.96316E-07
TG 52:3 FA18:1	MCP1	0.000153923	0.000858461	0.858906478	0.003642421	0.000598712	1.1035E-06
TG 52:3 FA18:1	TNF-α	0.000413699	0.001110602	0.712139385	0.108407103	0.026220328	0.000263541
TG 52:3 FA18:2	IL-17A	3.07498E-05	0.001910545	0.987265329	0.00192916	0.004098737	0.641279397
TG 52:3 FA18:2	IL-1β	0.00128553	0.001415193	0.370918782	0.081049596	0.029847603	0.01087203
TG 52:3 FA18:2	IL-6	0.001069998	0.001040145	0.311843198	0.019456431	0.003150208	8.52825E-07
TG 52:3 FA18:2	MCP1	0.000195739	0.001068167	0.855836587	0.003644062	0.000598779	1.09717E-06
TG 52:3 FA18:2	TNF-α	0.000513883	0.001371154	0.710459209	0.108836211	0.026019298	0.00023046
TG 52:3 FA18:3	IL-17A	0.004435348	0.355674907	0.990133015	0.001929917	0.004105161	0.641669185
TG 52:3 FA18:3	IL-1β	0.161277918	0.276485018	0.564041002	0.079768491	0.031372531	0.016400298
TG 52:3 FA18:3	IL-6	0.142193098	0.214494385	0.512442082	0.019261067	0.003494989	5.50437E-06
TG 52:3 FA18:3	MCP1	0.067549015	0.198287878	0.735732699	0.003679273	0.000598009	9.10999E-07
TG 52:3 FA18:3	TNF-α	0.004333661	0.272591512	0.987421024	0.108426085	0.02782954	0.000507661
TG 52:3 FA20:0	IL-17A	0.008009762	0.616760359	0.989724283	0.001933969	0.004099792	0.640537
TG 52:3 FA20:0	IL-1β	0.361734289	0.462765659	0.440528998	0.08292638	0.030241787	0.01019302
TG 52:3 FA20:0	IL-6	0.344055553	0.339532209	0.319009613	0.019492757	0.003186245	1.00361E-06
TG 52:3 FA20:0	MCP1	0.09891762	0.344441228	0.775946654	0.003625165	0.000598267	1.18098E-06
TG 52:3 FA20:0	TNF-α	0.169451459	0.439367388	0.702461372	0.108666528	0.025833895	0.000215947
TG 52:3 FA20:1	IL-17A	0.003734606	1.15191781	0.997434665	0.001938926	0.004089869	0.63887519
TG 52:3 FA20:1	IL-1β	0.51815777	0.891169054	0.565290174	0.078544533	0.031106348	0.017089211
TG 52:3 FA20:1	IL-6	0.512552913	0.657085643	0.441478677	0.01920758	0.003293539	2.23251E-06
TG 52:3 FA20:1	MCP1	0.136525211	0.631051812	0.830182858	0.003654938	0.000585447	7.08362E-07
TG 52:3 FA20:1	TNF-α	0.016598765	0.875678891	0.985002266	0.109179885	0.027501068	0.000414609
TG 52:3 FA20:2	IL-17A	0.003938257	0.396838373	0.99214756	0.001939313	0.004092847	0.639051527
TG 52:3 FA20:2	IL-1β	0.252774072	0.301082461	0.407801033	0.08466955	0.030527993	0.009444477
TG 52:3 FA20:2	IL-6	0.208036833	0.210605087	0.331148504	0.019275581	0.003066434	6.28632E-07
TG 52:3 FA20:2	MCP1	-0.011747957	0.238207334	0.960992604	0.003716826	0.000641951	2.51181E-06
TG 52:3 FA20:2	TNF-α	0.006495019	0.308442983	0.983339271	0.108779026	0.028138662	0.000551375
TG 52:3 FA20:3	IL-17A	0.003642541	0.330771625	0.991286577	0.001937255	0.004098525	0.639868432
TG 52:3 FA20:3	IL-1β	0.201218288	0.258178094	0.441862432	0.084362016	0.031449931	0.011771488
TG 52:3 FA20:3	IL-6	0.027493009	0.212703093	0.898018329	0.018947544	0.003720712	1.79434E-05
TG 52:3 FA20:3	MCP1	-0.043508179	0.19701998	0.826719878	0.003732736	0.000637888	2.11183E-06
TG 52:3 FA20:3	TNF-α	-0.010646157	0.252549745	0.9666547	0.10921045	0.027679802	0.000443457
TG 52:3 FA22:1	IL-17A	0.0250223	3.293756545	0.993992488	0.001935118	0.004493542	0.670024068
TG 52:3 FA22:1	IL-1β	2.239887945	2.304893204	0.339472424	0.091384198	0.032495124	0.008888823
TG 52:3 FA22:1	IL-6	2.159789828	1.651720633	0.201644393	0.020088221	0.003298413	1.43596E-06
TG 52:3 FA22:1	MCP1	0.713315821	1.624143067	0.663890952	0.00372861	0.000598021	9.74941E-07
TG 52:3 FA22:1	TNF-α	0.600250729	2.098803643	0.776986033	0.115167766	0.026488868	0.00016436
TG 52:4 FA14:0	IL-17A	0.009441312	0.473899766	0.984241568	0.001948238	0.003046067	0.52745909
TG 52:4 FA14:0	IL-1β	0.295624656	0.497446933	0.55693053	0.053048202	0.034812985	0.138389352
TG 52:4 FA14:0	IL-6	0.320638586	0.313347514	0.314641568	0.019248988	0.002850018	2.0607E-07
TG 52:4 FA14:0	MCP1	0.071082623	0.358180627	0.84407405	0.003651188	0.00060232	1.3443E-06
TG 52:4 FA14:0	TNF-α	0.140429527	0.448878373	0.756636918	0.107946706	0.0255191	0.000213527
TG 52:4 FA16:0	IL-17A	5.28119E-06	0.004439591	0.999058739	0.001939961	0.004089671	0.63868051
TG 52:4 FA16:0	IL-1β	0.001379918	0.003652932	0.708271337	0.075894773	0.033081715	0.028956376
TG 52:4 FA16:0	IL-6	0.001440284	0.002638562	0.589199004	0.019368655	0.003431354	3.77866E-06
TG 52:4 FA16:0	MCP1	-0.000169389	0.002585287	0.948194253	0.003733049	0.000622285	1.3984E-06
TG 52:4 FA16:0	TNF-α	-0.000291682	0.003435228	0.932897736	0.108819572	0.02799096	0.000519459

TG 52:4 FA16:1	IL-17A	0.000127847	0.015761644	0.993581923	0.001942458	0.004088885	0.63818607
TG 52:4 FA16:1	IL-1β	0.009445324	0.012006934	0.437653772	0.077127517	0.030622208	0.017344519
TG 52:4 FA16:1	IL-6	0.01007257	0.008767845	0.259712643	0.019422909	0.003211066	1.21678E-06
TG 52:4 FA16:1	MCP1	-0.000631237	0.009251953	0.946057324	0.003732831	0.00062715	1.59437E-06
TG 52:4 FA16:1	TNF-α	0.001103432	0.011960943	0.927110358	0.107700498	0.027446411	0.000470305
TG 52:4 FA18:0	IL-17A	0.028173536	0.896623617	0.975148412	0.001947955	0.003043221	0.52713513
TG 52:4 FA18:0	IL-1β	0.688737485	0.930867743	0.465316176	0.058737861	0.034399545	0.098411883
TG 52:4 FA18:0	IL-6	0.573386067	0.595025726	0.343197426	0.019622577	0.00285777	1.52623E-07
TG 52:4 FA18:0	MCP1	0.226533303	0.67927916	0.741163231	0.003662775	0.000603175	1.30579E-06
TG 52:4 FA18:0	TNF-α	0.331442423	0.840615762	0.69625505	0.10856135	0.025235065	0.000175245
TG 52:4 FA18:1	IL-17A	8.23387E-05	0.009797006	0.993349915	0.001941815	0.004091446	0.638506255
TG 52:4 FA18:1	IL-1β	0.005917021	0.007300411	0.424033136	0.076504074	0.029973104	0.016027603
TG 52:4 FA18:1	IL-6	0.005899119	0.005433926	0.286298789	0.019444896	0.003203687	1.14816E-06
TG 52:4 FA18:1	MCP1	-0.000329173	0.005745392	0.954691366	0.003726248	0.000626957	1.63351E-06
TG 52:4 FA18:1	TNF-α	0.000407891	0.00751666	0.957083897	0.107988439	0.027766751	0.000517393
TG 52:4 FA18:2	IL-17A	3.18277E-06	0.002852715	0.999117187	0.001940178	0.004089803	0.638654042
TG 52:4 FA18:2	IL-1β	0.000844679	0.002354563	0.722301013	0.076294125	0.033186032	0.028646379
TG 52:4 FA18:2	IL-6	0.000832173	0.001725498	0.633107308	0.019255919	0.003492298	5.46153E-06
TG 52:4 FA18:2	MCP1	-0.000112475	0.001643613	0.945895984	0.003734015	0.000615713	1.1643E-06
TG 52:4 FA18:2	TNF-α	-0.000178515	0.002208098	0.936101624	0.108820463	0.028001352	0.000521465
TG 52:4 FA18:3	IL-17A	0.000153839	0.01863384	0.993467484	0.001942191	0.004092824	0.638554092
TG 52:4 FA18:3	IL-1β	0.007671728	0.014068946	0.589582156	0.075429276	0.030379682	0.018849731
TG 52:4 FA18:3	IL-6	0.009113886	0.010184703	0.377985593	0.019337829	0.003158071	9.88285E-07
TG 52:4 FA18:3	MCP1	-0.000512875	0.010850278	0.962612622	0.003718331	0.000622725	1.51178E-06
TG 52:4 FA18:3	TNF-α	-1.08559E-12	0.014133232	1	0.109288938	0.027458632	0.000403266
TG 52:4 FA20:0	IL-17A	0.004766904	0.398294203	0.990532865	0.001941866	0.003047357	0.528974462
TG 52:4 FA20:0	IL-1β	0.188247104	0.406196867	0.646509894	0.049386833	0.033837432	0.155167018
TG 52:4 FA20:0	IL-6	0.324205718	0.285589863	0.265581276	0.019913702	0.003091935	4.79249E-07
TG 52:4 FA20:0	MCP1	0.056877007	0.290611689	0.846198322	0.003686517	0.000581707	6.33912E-07
TG 52:4 FA20:0	TNF-α	0.134619529	0.36863136	0.717621526	0.110169633	0.024945676	0.0001279
TG 52:4 FA20:2	IL-17A	0.005632607	1.398758708	0.996814612	0.001938688	0.004232747	0.65035083
TG 52:4 FA20:2	IL-1β	0.819203913	1.0909633	0.458763272	0.087889938	0.033372729	0.013409098
TG 52:4 FA20:2	IL-6	0.738916517	0.708325259	0.305479777	0.019724703	0.003074447	5.12603E-07
TG 52:4 FA20:2	MCP1	0.011578685	0.722124051	0.98731691	0.003764585	0.000579208	4.0855E-07
TG 52:4 FA20:2	TNF-α	0.253933655	0.928323596	0.786376927	0.115125029	0.026001189	0.000123975
TG 52:4 FA20:3	IL-17A	0.003442805	0.288032036	0.990542364	0.001938552	0.004089665	0.638922845
TG 52:4 FA20:3	IL-1β	0.170160326	0.210363361	0.424948614	0.080613477	0.029364186	0.010114577
TG 52:4 FA20:3	IL-6	0.155691082	0.1549704	0.323100211	0.01923064	0.003106337	8.19006E-07
TG 52:4 FA20:3	MCP1	-0.039020647	0.175041	0.825106406	0.003732648	0.000649414	2.82765E-06
TG 52:4 FA20:3	TNF-α	0.004890329	0.225521252	0.982843169	0.108417139	0.028323772	0.000611583
TG 52:4 FA20:4	IL-17A	-0.00350167	0.110685083	0.974971614	0.001955476	0.004081018	0.635296565
TG 52:4 FA20:4	IL-1β	-0.060112874	0.095793472	0.535062259	0.066231747	0.034722927	0.066078483
TG 52:4 FA20:4	IL-6	-0.02042017	0.072242116	0.779376385	0.018535297	0.003760302	2.84503E-05
TG 52:4 FA20:4	MCP1	-0.041345609	0.063629704	0.520776129	0.003693942	0.000613019	1.29724E-06
TG 52:4 FA20:4	TNF-α	-0.052118338	0.084035361	0.539812141	0.107194064	0.027406794	0.000487074
TG 52:4 FA22:1	IL-17A	0.011303073	2.094723253	0.995735846	0.001938696	0.004571436	0.674989243
TG 52:4 FA22:1	IL-1β	0.663306169	1.509862318	0.664062515	0.060091155	0.037874957	0.124699389
TG 52:4 FA22:1	IL-6	1.141551212	0.884385791	0.208138818	0.020536651	0.002838653	1.10484E-07
TG 52:4 FA22:1	MCP1	0.16977592	0.983354465	0.864263699	0.003670281	0.000587231	1.2955E-06
TG 52:4 FA22:1	TNF-α	2.98139E-11	1.227671697	1	0.114503817	0.02497114	0.000100407

TG 52:4 FA22:4	IL-17A	-0.068277829	2.145177441	0.974834602	0.001957392	0.00449994	0.666910041
TG 52:4 FA22:4	IL-1β	-0.831915535	1.831008516	0.653080889	0.063459141	0.038592015	0.111284786
TG 52:4 FA22:4	IL-6	-0.099740738	1.466857327	0.946271901	0.018620748	0.004547021	0.000325415
TG 52:4 FA22:4	MCP1	-0.649728845	1.230025247	0.601505775	0.003474459	0.000698213	2.95971E-05
TG 52:4 FA22:4	TNF-α	-0.725145851	1.348752808	0.595072142	0.107354717	0.02500105	0.000190165
TG 52:5 FA14:0	IL-17A	0.002412545	0.696867455	0.997263194	0.001939411	0.004322891	0.657273123
TG 52:5 FA14:0	IL-1β	0.169862273	0.542753258	0.756712665	0.05786878	0.038704515	0.146474533
TG 52:5 FA14:0	IL-6	0.303772464	0.33114607	0.36708816	0.019738215	0.003017406	5.14754E-07
TG 52:5 FA14:0	MCP1	0.004297074	0.343843196	0.990120831	0.00376742	0.000576837	5.28621E-07
TG 52:5 FA14:0	TNF-α	-9.92473E-12	0.41582849	1	0.117015133	0.023864753	3.9499E-05
TG 52:5 FA16:0	IL-17A	6.08265E-18	0.037927017	1	1.65271E-16	0.00429588	1
TG 52:5 FA16:0	IL-1β	-0.003199358	0.030192785	0.916316052	0.070170073	0.033620763	0.045475008
TG 52:5 FA16:0	IL-6	0.007859313	0.022371195	0.727808238	0.019264839	0.003577218	7.84511E-06
TG 52:5 FA16:0	MCP1	-0.003352852	0.021236594	0.8756086	0.003712064	0.000628526	1.81385E-06
TG 52:5 FA16:0	TNF-α	-0.004872205	0.028087004	0.863448512	0.107681157	0.028140095	0.000613532
TG 52:5 FA16:1	IL-17A	3.31091E-05	0.03681933	0.99928847	0.001940489	0.004097618	0.639238229
TG 52:5 FA16:1	IL-1β	0.00485079	0.029606103	0.870952626	0.076068248	0.032392001	0.025640447
TG 52:5 FA16:1	IL-6	0.00918799	0.023081723	0.693402144	0.019238238	0.003626408	9.84345E-06
TG 52:5 FA16:1	MCP1	-0.001979877	0.02087032	0.925052343	0.003736533	0.000606903	9.00443E-07
TG 52:5 FA16:1	TNF-α	-0.002081856	0.028685816	0.94262651	0.108726829	0.028238361	0.000575169
TG 52:5 FA18:1	IL-17A	0.000643757	0.099071553	0.994858485	0.001945437	0.004100975	0.638660925
TG 52:5 FA18:1	IL-1β	0.027749521	0.078447963	0.726014011	0.076916783	0.031924257	0.022324781
TG 52:5 FA18:1	IL-6	0.049565044	0.053209897	0.359030951	0.019315395	0.003109449	7.72411E-07
TG 52:5 FA18:1	MCP1	-0.007554745	0.058092116	0.897396794	0.003713154	0.000628333	1.79593E-06
TG 52:5 FA18:1	TNF-α	-0.002789087	0.075395893	0.970736005	0.108961988	0.027605942	0.00044157
TG 52:5 FA18:2	IL-17A	6.59729E-12	0.017973327	1	0.001940925	0.004096419	0.639065412
TG 52:5 FA18:2	IL-1β	0.000903249	0.015005483	0.952399862	0.07374657	0.033622259	0.036167012
TG 52:5 FA18:2	IL-6	0.002668374	0.011570215	0.819172175	0.019211315	0.003722805	1.48082E-05
TG 52:5 FA18:2	MCP1	-0.000925924	0.010146874	0.927898867	0.003726022	0.000604287	8.77513E-07
TG 52:5 FA18:2	TNF-α	-0.001491876	0.013788787	0.9145615	0.108523514	0.027798331	0.000496846
TG 52:5 FA18:3	IL-17A	2.44469E-05	0.029141767	0.999336213	0.001940395	0.004099723	0.639425787
TG 52:5 FA18:3	IL-1β	-0.001613733	0.023964903	0.946759922	0.071130393	0.033144794	0.040079153
TG 52:5 FA18:3	IL-6	0.009839967	0.017111556	0.56954683	0.019252489	0.003398451	3.56719E-06
TG 52:5 FA18:3	MCP1	-0.003748788	0.017156078	0.828510847	0.003693826	0.000630655	2.07998E-06
TG 52:5 FA18:3	TNF-α	-0.002589923	0.022397005	0.908710787	0.10793187	0.027870538	0.00054125
TG 52:5 FA20:3	IL-17A	0.012108632	1.10165993	0.991305685	0.001950433	0.004239876	0.648931521
TG 52:5 FA20:3	IL-1β	0.599562657	0.794767573	0.456699189	0.083492259	0.030920596	0.011444158
TG 52:5 FA20:3	IL-6	0.567146207	0.563458167	0.32247844	0.019683996	0.003110442	6.49691E-07
TG 52:5 FA20:3	MCP1	-0.033076362	0.573355622	0.95439196	0.003782924	0.000584889	4.45191E-07
TG 52:5 FA20:3	TNF-α	0.058179072	0.726713415	0.936741218	0.116848535	0.025887085	9.77412E-05
TG 52:5 FA20:4	IL-17A	-3.44105E-16	0.120166509	1	7.13416E-17	0.00429336	1
TG 52:5 FA20:4	IL-1β	-0.079646769	0.094038538	0.403724238	0.066226894	0.033030922	0.054059463
TG 52:5 FA20:4	IL-6	-0.008755006	0.077122597	0.910374102	0.018795346	0.003889989	3.74552E-05
TG 52:5 FA20:4	MCP1	-0.034141488	0.066501995	0.61143868	0.003739092	0.000620845	1.30901E-06
TG 52:5 FA20:4	TNF-α	-0.033225488	0.086495276	0.703590918	0.106670153	0.027335244	0.000499111
TG 52:5 FA20:5	IL-17A	-0.145006795	0.554470947	0.795476549	0.001995828	0.004045396	0.625352132
TG 52:5 FA20:5	IL-1β	-0.48714703	0.474108355	0.312398156	0.074789823	0.034006418	0.035704129
TG 52:5 FA20:5	IL-6	-0.017618133	0.378765345	0.963208333	0.018891198	0.003901265	3.63518E-05
TG 52:5 FA20:5	MCP1	-0.123084489	0.328122739	0.710212345	0.003691736	0.000625538	1.83574E-06
TG 52:5 FA20:5	TNF-α	-0.17741936	0.435475189	0.686595507	0.106956757	0.028103632	0.00064925

TG 52:5 FA22:5	IL-17A	8.46249E-16	0.725432988	1	1.65589E-16	0.004290456	1
TG 52:5 FA22:5	IL-1β	0.166363057	0.555067488	0.766460026	0.079498722	0.03227401	0.019725885
TG 52:5 FA22:5	IL-6	0.145630758	0.422842946	0.732941686	0.019144307	0.003530516	7.06536E-06
TG 52:5 FA22:5	MCP1	-0.048894307	0.402324177	0.904081943	0.003723279	0.000621752	1.44037E-06
TG 52:5 FA22:5	TNF-α	-0.075642408	0.533347575	0.888165823	0.107920194	0.027901863	0.000548334
TG 52:6 FA14:0	IL-17A	-0.001100284	1.145830741	0.999241162	0.001945552	0.004523302	0.670654184
TG 52:6 FA14:0	IL-1β	-4.44687E-09	0.907196606	0.999999996	0.060606061	0.041655656	0.15765656
TG 52:6 FA14:0	IL-6	0.421368287	0.604545032	0.491987967	0.019590566	0.003591309	1.01557E-05
TG 52:6 FA14:0	MCP1	0.006580559	0.56289665	0.990761765	0.003769275	0.000612782	1.66941E-06
TG 52:6 FA14:0	TNF-α	-0.041673674	0.694233752	0.952592127	0.11511431	0.02549723	0.000120991
TG 52:6 FA16:1	IL-17A	4.14253E-11	0.279023733	1	0.001941878	0.004110341	0.640033682
TG 52:6 FA16:1	IL-1β	-0.020448521	0.227812497	0.929074284	0.071104629	0.032992501	0.039296837
TG 52:6 FA16:1	IL-6	0.036670454	0.177869381	0.83805474	0.019146533	0.003699055	1.41691E-05
TG 52:6 FA16:1	MCP1	-0.067696435	0.15983352	0.674920108	0.003651656	0.000615233	1.6704E-06
TG 52:6 FA16:1	TNF-α	-0.023064378	0.213822058	0.914819263	0.108732928	0.027861593	0.00049868
TG 52:6 FA18:1	IL-17A	0.008736867	1.27210804	0.994565623	0.001945807	0.004102481	0.638720156
TG 52:6 FA18:1	IL-1β	-0.000145002	1.040485182	0.999889729	0.072461916	0.032988262	0.035914392
TG 52:6 FA18:1	IL-6	0.65099257	0.692372694	0.354606961	0.019483828	0.003152212	8.41587E-07
TG 52:6 FA18:1	MCP1	-0.099007478	0.74705298	0.895449457	0.003712015	0.000629519	1.86221E-06
TG 52:6 FA18:1	TNF-α	-0.031775537	0.959753309	0.973807744	0.109017321	0.027377822	0.000401248
TG 52:6 FA18:2	IL-17A	-5.79869E-05	0.195840273	0.999765712	0.001941669	0.004110491	0.640081735
TG 52:6 FA18:2	IL-1β	-0.014910933	0.160432219	0.926567353	0.071109817	0.033104293	0.039906171
TG 52:6 FA18:2	IL-6	0.024386678	0.126287191	0.848178222	0.019151991	0.003742001	1.66888E-05
TG 52:6 FA18:2	MCP1	-0.035754248	0.114467602	0.756933444	0.003669418	0.000627782	2.15166E-06
TG 52:6 FA18:2	TNF-α	-0.037526302	0.151201352	0.805680289	0.107672596	0.028071405	0.000598591
TG 52:6 FA18:3	IL-17A	5.89392E-15	0.18023151	1	8.39601E-16	0.004313683	1
TG 52:6 FA18:3	IL-1β	-0.025631582	0.140011309	0.855976341	0.07015668	0.032944399	0.041529204
TG 52:6 FA18:3	IL-6	0.040283862	0.103611999	0.700173386	0.019300226	0.003500906	5.47542E-06
TG 52:6 FA18:3	MCP1	-0.036698417	0.09914665	0.71387655	0.003670322	0.000620055	1.74722E-06
TG 52:6 FA18:3	TNF-α	-0.025259785	0.130809108	0.848178806	0.107999426	0.02769314	0.000502439
TG 52:6 FA20:4	IL-17A	-0.005018725	0.3477763	0.988588543	0.00194067	0.003223351	0.551974769
TG 52:6 FA20:4	IL-1β	-0.365918369	0.345194924	0.298187965	0.033934472	0.035557546	0.348071874
TG 52:6 FA20:4	IL-6	-2.06589E-10	0.293051767	0.999999999	0.018879747	0.003917216	4.54064E-05
TG 52:6 FA20:4	MCP1	-0.101770858	0.259640302	0.69804896	0.003696349	0.000640287	3.37711E-06
TG 52:6 FA20:4	TNF-α	-0.094641508	0.305337998	0.758889245	0.10757994	0.025018876	0.000187127
TG 52:6 FA20:5	IL-17A	-6.28305E-16	0.60937032	1	1.32545E-17	0.004297232	1
TG 52:6 FA20:5	IL-1β	-0.485187639	0.47890802	0.319108208	0.075302467	0.033201719	0.030689578
TG 52:6 FA20:5	IL-6	0.112148035	0.354264259	0.753765252	0.019142	0.003526856	6.96693E-06
TG 52:6 FA20:5	MCP1	-0.123276086	0.343354114	0.722083716	0.003709089	0.000632681	2.04891E-06
TG 52:6 FA20:5	TNF-α	-0.09969753	0.445043437	0.824262308	0.106890789	0.027760457	0.00057494
TG 52:6 FA22:6	IL-17A	-0.013243524	0.566799417	0.9815188	0.001943406	0.004306739	0.655167445
TG 52:6 FA22:6	IL-1β	-0.408636334	0.450888032	0.372250187	0.065983963	0.034335146	0.06451151
TG 52:6 FA22:6	IL-6	0.171677549	0.349286808	0.626766251	0.018952201	0.00381659	2.79005E-05
TG 52:6 FA22:6	MCP1	-0.02336544	0.325230503	0.943220278	0.003646942	0.000656465	5.42653E-06
TG 52:6 FA22:6	TNF-α	-0.073755781	0.397827803	0.854208864	0.106152449	0.026726067	0.000431799
TG 52:7 FA16:0	IL-17A	0.03192987	0.553286487	0.954362729	0.001935342	0.004045141	0.635808251
TG 52:7 FA16:0	IL-1β	0.432374535	0.428437832	0.3209551	0.084285923	0.030794448	0.010318823
TG 52:7 FA16:0	IL-6	0.252493432	0.327534579	0.44680083	0.018813943	0.003380599	4.7245E-06
TG 52:7 FA16:0	MCP1	0.134317243	0.321597345	0.679172426	0.003681636	0.000614371	1.42372E-06
TG 52:7 FA16:0	TNF-α	0.157185603	0.406473761	0.701703768	0.107793311	0.026286507	0.000289319

TG 52:7 FA18:1	IL-17A	-143.3310411	114.3789802	0.241737391	-0.079588335	0.043032759	0.097431159
TG 52:7 FA18:1	IL-1β	-139.2005675	134.212783	0.326721508	0.130260343	0.063438421	0.070233714
TG 52:7 FA18:1	IL-6	-11.72432214	157.361461	0.942237767	0.018365662	0.014419736	0.234696408
TG 52:7 FA18:1	MCP1	-40.40109904	125.4562918	0.754781679	0.003412206	0.002204996	0.156150765
TG 52:7 FA18:1	TNF-α	-76.27776287	133.61768	0.582061974	-0.102526356	0.083135158	0.248721081
TG 52:7 FA20:5	IL-17A	0.00496412	2.614814239	0.998500317	0.00194683	0.00447978	0.66759426
TG 52:7 FA20:5	IL-1β	-1.062290613	2.159370291	0.627050547	0.058142086	0.042099652	0.179482393
TG 52:7 FA20:5	IL-6	1.060803552	1.437925831	0.467546104	0.019679125	0.003740721	1.90082E-05
TG 52:7 FA20:5	MCP1	0.296634841	1.267171977	0.816820002	0.003886403	0.000610276	1.14858E-06
TG 52:7 FA20:5	TNF-α	-0.090879632	1.656211561	0.956676611	0.115439627	0.026529052	0.00020011
TG 52:7 FA22:6	IL-17A	-0.001296012	1.499018179	0.999316526	0.001944728	0.004658252	0.67962838
TG 52:7 FA22:6	IL-1β	0.111422039	1.170918793	0.924892232	0.080345795	0.038036964	0.044051927
TG 52:7 FA22:6	IL-6	0.524558252	0.878751043	0.55552307	0.019387317	0.004096976	6.25736E-05
TG 52:7 FA22:6	MCP1	0.166508507	0.749094541	0.825768993	0.003729164	0.000640897	3.41621E-06
TG 52:7 FA22:6	TNF-α	-0.057169889	0.944036081	0.952156463	0.11446672	0.027294874	0.000264634
TG 52:8 FA16:1	IL-17A	0.081199565	3.179994725	0.979797731	0.001936412	0.004083448	0.638783513
TG 52:8 FA16:1	IL-1β	2.087785137	2.376556806	0.386659124	0.080970145	0.03000197	0.011316357
TG 52:8 FA16:1	IL-6	1.594593657	1.838269148	0.39258519	0.019013592	0.003332442	3.18294E-06
TG 52:8 FA16:1	MCP1	0.670423538	1.860088803	0.721054308	0.003620333	0.000624121	2.43684E-06
TG 52:8 FA16:1	TNF-α	0.964813814	2.3183262	0.680249191	0.107918608	0.026332486	0.000291245
TG 52:8 FA18:2	IL-17A	8.775760653	13.13676301	0.510487881	0.001695893	0.003827618	0.661684999
TG 52:8 FA18:2	IL-1β	13.58366746	9.596023727	0.169753413	0.060224659	0.032859626	0.079270033
TG 52:8 FA18:2	IL-6	13.14471612	7.392236892	0.088048002	0.019362515	0.003281105	4.34624E-06
TG 52:8 FA18:2	MCP1	8.977574866	7.175703001	0.222954275	0.003659666	0.000582146	1.69162E-06
TG 52:8 FA18:2	TNF-α	6.813913853	9.023141456	0.457500479	0.115134009	0.024973597	0.000111887
TG 53:0 FA16:0	IL-17A	-4.46342E-16	0.489237548	1	7.1239E-17	0.004296124	1
TG 53:0 FA16:0	IL-1β	-0.340795936	0.408170867	0.410354631	0.074744831	0.035237091	0.042280587
TG 53:0 FA16:0	IL-6	0.041991392	0.307264806	0.892211058	0.019188738	0.003809103	2.095E-05
TG 53:0 FA16:0	MCP1	-0.106204107	0.27522703	0.702309871	0.003740195	0.000631514	1.73145E-06
TG 53:0 FA16:0	TNF-α	-0.103204864	0.360892334	0.77686552	0.106732616	0.02803183	0.000646166
TG 53:1 FA16:0	IL-17A	0.015414836	0.438570365	0.97219462	0.00194357	0.004068544	0.636321567
TG 53:1 FA16:0	IL-1β	0.31503526	0.318517622	0.330541045	0.083880885	0.029049202	0.007136925
TG 53:1 FA16:0	IL-6	0.224586805	0.249437962	0.375093868	0.019053719	0.003266745	2.22791E-06
TG 53:1 FA16:0	MCP1	0.102824161	0.250047784	0.683835552	0.003665922	0.000606119	1.21865E-06
TG 53:1 FA16:0	TNF-α	0.124833373	0.319169684	0.698473241	0.108215892	0.026190158	0.000265398
TG 53:1 FA17:0	IL-17A	0.314797561	0.380216617	0.414241112	0.001577644	0.003024645	0.605781135
TG 53:1 FA17:0	IL-1β	0.438043584	0.380119756	0.258264114	0.086758459	0.029727933	0.006610411
TG 53:1 FA17:0	IL-6	0.258694971	0.303467544	0.400711537	0.018865326	0.003408069	5.13804E-06
TG 53:1 FA17:0	MCP1	0.196420452	0.278830718	0.486589668	0.003662434	0.000579587	5.73582E-07
TG 53:1 FA17:0	TNF-α	0.259187749	0.383565594	0.504386484	0.104291982	0.026989799	0.00055393
TG 53:1 FA18:0	IL-17A	0.228257005	0.270356162	0.405194081	0.001680134	0.0030094	0.580785997
TG 53:1 FA18:0	IL-1β	0.332337746	0.283464245	0.250256674	0.089385492	0.031020077	0.007243898
TG 53:1 FA18:0	IL-6	0.187407062	0.216724005	0.394050845	0.018895023	0.003405677	4.9587E-06
TG 53:1 FA18:0	MCP1	0.142270722	0.197318993	0.476479551	0.003654299	0.000573915	5.01777E-07
TG 53:1 FA18:0	TNF-α	0.195822384	0.273295515	0.479210092	0.103639212	0.026908707	0.000573309
TG 53:1 FA18:1	IL-17A	0.183650945	0.217482089	0.405106607	0.001755504	0.003008057	0.563851426
TG 53:1 FA18:1	IL-1β	0.255750185	0.219389984	0.252907155	0.087634707	0.029831863	0.006301582
TG 53:1 FA18:1	IL-6	0.157652695	0.168747689	0.357633804	0.018981827	0.003294984	2.72543E-06
TG 53:1 FA18:1	MCP1	0.115780329	0.158802725	0.471605087	0.003631231	0.000573925	5.6105E-07
TG 53:1 FA18:1	TNF-α	0.155171893	0.222933807	0.491758542	0.105720352	0.027274403	0.000536018

TG 53:2 FA16:0	IL-17A	0.00458796	0.186812588	0.980569233	0.001944267	0.004080484	0.637185102
TG 53:2 FA16:0	IL-1β	0.146136322	0.13410553	0.284516404	0.086293251	0.028797397	0.005436737
TG 53:2 FA16:0	IL-6	0.100224679	0.108089534	0.361203424	0.019274653	0.003333052	2.56172E-06
TG 53:2 FA16:0	MCP1	0.044707674	0.102283376	0.665171914	0.00365646	0.000583775	6.69175E-07
TG 53:2 FA16:0	TNF-α	0.05698378	0.135502206	0.677089946	0.108371808	0.02617994	0.000259899
TG 53:2 FA17:0	IL-17A	0.013108655	0.082127422	0.874255254	0.001869091	0.00397277	0.641417993
TG 53:2 FA17:0	IL-1β	0.070049357	0.059154102	0.245637863	0.084118789	0.028131384	0.005524238
TG 53:2 FA17:0	IL-6	0.054214368	0.047812457	0.26581803	0.019073427	0.00326512	2.17275E-06
TG 53:2 FA17:0	MCP1	0.032190039	0.046970705	0.498402723	0.003608008	0.0005937	1.12401E-06
TG 53:2 FA17:0	TNF-α	0.057602898	0.062542849	0.364390169	0.105756615	0.026760834	0.000435716
TG 53:2 FA18:1	IL-17A	0.009126226	0.038447632	0.813985126	0.001851442	0.003804421	0.630039145
TG 53:2 FA18:1	IL-1β	0.03508458	0.028144295	0.222191533	0.085488822	0.02737854	0.003951335
TG 53:2 FA18:1	IL-6	0.027402629	0.023323923	0.249287141	0.019195252	0.003258168	1.88935E-06
TG 53:2 FA18:1	MCP1	0.015612242	0.02283636	0.499438468	0.003626385	0.000590447	9.38735E-07
TG 53:2 FA18:1	TNF-α	0.031415341	0.030581105	0.312500927	0.105290774	0.02676633	0.000458013
TG 53:2 FA18:2	IL-17A	0.014868333	0.707916158	0.98338237	0.001936325	0.004085593	0.638974157
TG 53:2 FA18:2	IL-1β	0.487482439	0.523277733	0.35898334	0.083285195	0.029689745	0.008741543
TG 53:2 FA18:2	IL-6	0.343523579	0.421650619	0.421658184	0.019206013	0.003435412	4.39916E-06
TG 53:2 FA18:2	MCP1	0.157187728	0.398679784	0.696170087	0.003684823	0.000601218	9.72925E-07
TG 53:2 FA18:2	TNF-α	0.198890635	0.509365191	0.698949717	0.108805124	0.026002747	0.000229525
TG 53:3 FA16:0	IL-17A	0.00439661	0.305089268	0.988597579	0.001939462	0.004090743	0.638854145
TG 53:3 FA16:0	IL-1β	0.167014687	0.22980238	0.472996968	0.081781861	0.030292196	0.011290807
TG 53:3 FA16:0	IL-6	0.148746114	0.177268947	0.408050853	0.019340681	0.003355528	2.70254E-06
TG 53:3 FA16:0	MCP1	0.049396005	0.17014529	0.773571436	0.003659489	0.000596115	9.46262E-07
TG 53:3 FA16:0	TNF-α	0.00430263	0.235057146	0.985517049	0.109135282	0.027878246	0.000482448
TG 53:3 FA17:0	IL-17A	0.003116061	0.100276876	0.975415883	0.001947215	0.004085242	0.637067721
TG 53:3 FA17:0	IL-1β	0.066339894	0.075506802	0.386603955	0.074911236	0.030241552	0.019104176
TG 53:3 FA17:0	IL-6	0.056760378	0.056395774	0.322241263	0.019358433	0.003243519	1.52333E-06
TG 53:3 FA17:0	MCP1	0.025357588	0.054612316	0.645770586	0.003622105	0.000581356	7.33492E-07
TG 53:3 FA17:0	TNF-α	0.055783046	0.077378769	0.476546223	0.109121231	0.027884032	0.000484189
TG 53:3 FA18:2	IL-17A	0.003071101	0.096320486	0.974775591	0.001947938	0.004085917	0.636998539
TG 53:3 FA18:2	IL-1β	0.067808774	0.070850326	0.346181239	0.078522614	0.02954702	0.012494251
TG 53:3 FA18:2	IL-6	0.059586466	0.054232091	0.280625415	0.019395009	0.003247731	1.5084E-06
TG 53:3 FA18:2	MCP1	0.020339071	0.053470864	0.706345813	0.003633289	0.000592684	9.69383E-07
TG 53:3 FA18:2	TNF-α	0.053443371	0.07318672	0.470912046	0.107403593	0.027461222	0.000487237
TG 53:4 FA16:0	IL-17A	-1.34939E-15	1.039063998	1	3.17496E-17	0.004291183	1
TG 53:4 FA16:0	IL-1β	0.202833524	0.805582484	0.802924211	0.079641913	0.032707382	0.021052012
TG 53:4 FA16:0	IL-6	0.243117123	0.595537077	0.686002734	0.019326417	0.003472134	4.71279E-06
TG 53:4 FA16:0	MCP1	-0.08864436	0.573422095	0.878181222	0.003717442	0.000618791	1.36502E-06
TG 53:4 FA16:0	TNF-α	-0.128405274	0.766270308	0.868044696	0.107828309	0.027991973	0.000572367
TG 53:4 FA17:0	IL-17A	0.003898926	0.258692537	0.988074798	0.001941445	0.004111874	0.640232128
TG 53:4 FA17:0	IL-1β	0.144612926	0.196556831	0.467608243	0.074093845	0.030714613	0.022173283
TG 53:4 FA17:0	IL-6	0.147451182	0.146831966	0.32330553	0.019605571	0.003294803	1.60151E-06
TG 53:4 FA17:0	MCP1	0.058889145	0.141876212	0.681041233	0.003627956	0.000589251	9.00005E-07
TG 53:4 FA17:0	TNF-α	0.094378278	0.190286883	0.623523593	0.109080944	0.026753555	0.000308675
TG 53:4 FA18:2	IL-17A	0.00294189	0.113870585	0.979559734	0.001951492	0.004096243	0.637233847
TG 53:4 FA18:2	IL-1β	0.074729026	0.08488712	0.385676473	0.077515892	0.030020433	0.014946263
TG 53:4 FA18:2	IL-6	0.070230319	0.065189261	0.289920186	0.019571117	0.003310573	1.78503E-06
TG 53:4 FA18:2	MCP1	0.026048148	0.062250158	0.67860367	0.003612293	0.000585126	8.59275E-07
TG 53:4 FA18:2	TNF-α	0.043817314	0.084848804	0.609349591	0.108712128	0.026998313	0.000354883

TG 53:4 FA18:3	IL-17A	0.063690035	0.899818502	0.944075206	0.001962629	0.004104038	0.636207164
TG 53:4 FA18:3	IL-1β	0.57941398	0.647492119	0.378490942	0.061750188	0.033152121	0.073037493
TG 53:4 FA18:3	IL-6	0.603654652	0.414062713	0.15599898	0.020179076	0.002716491	4.32945E-08
TG 53:4 FA18:3	MCP1	0.303420757	0.44011707	0.496239894	0.003835787	0.000532903	7.80204E-08
TG 53:4 FA18:3	TNF-α	0.461365658	0.596859006	0.446005459	0.119488915	0.025198484	5.61596E-05
TG 53:4 FA20:4	IL-17A	-0.014023212	2.278257201	0.995134117	0.001942526	0.004318096	0.656404452
TG 53:4 FA20:4	IL-1β	-0.775304274	1.853619993	0.679061577	0.042139503	0.040387347	0.306023077
TG 53:4 FA20:4	IL-6	0.092864333	1.385948973	0.947072113	0.019280611	0.003858573	3.07167E-05
TG 53:4 FA20:4	MCP1	-0.290674136	1.153913851	0.803023796	0.003780483	0.000591469	7.58849E-07
TG 53:4 FA20:4	TNF-α	-0.245763722	1.362833795	0.858237971	0.114290865	0.023897421	5.46382E-05
TG 53:5 FA20:4	IL-17A	-0.009221362	1.096369228	0.993351056	0.001942443	0.004323691	0.65683274
TG 53:5 FA20:4	IL-1β	-0.616769942	0.875572666	0.487203884	0.047449201	0.039694026	0.242334986
TG 53:5 FA20:4	IL-6	0.261946662	0.599232935	0.665488751	0.019781287	0.003471231	4.69749E-06
TG 53:5 FA20:4	MCP1	-0.19919761	0.565046678	0.727175685	0.003805568	0.00060263	9.26675E-07
TG 53:5 FA20:4	TNF-α	-0.114065662	0.657318883	0.863527985	0.114809201	0.023982396	5.3957E-05
TG 53:6 FA20:4	IL-17A	-1.987148315	2.595765866	0.451120714	0.002068186	0.0045921	0.656318735
TG 53:6 FA20:4	IL-1β	-2.108475048	2.140482523	0.334039191	0.044977079	0.044216314	0.318799421
TG 53:6 FA20:4	IL-6	0.051800951	1.818128035	0.977496481	0.019244743	0.00499168	0.000717653
TG 53:6 FA20:4	MCP1	-0.672011203	1.331124423	0.618089247	0.003690996	0.00066294	8.64823E-06
TG 53:6 FA20:4	TNF-α	-1.030181013	1.410823531	0.472056599	0.112540022	0.023207998	5.50764E-05
TG 54:0 FA16:0	IL-17A	0.036559828	1.684453318	0.982827579	0.001917389	0.004171581	0.649094481
TG 54:0 FA16:0	IL-1β	0.898493339	1.307104291	0.497115831	0.076942258	0.031823862	0.021900042
TG 54:0 FA16:0	IL-6	0.771217791	0.985242543	0.439898119	0.019196816	0.003444591	4.62231E-06
TG 54:0 FA16:0	MCP1	0.588150585	0.889857403	0.513689006	0.003644569	0.000575834	5.57644E-07
TG 54:0 FA16:0	TNF-α	0.460227461	1.190849823	0.701876399	0.110008179	0.026086474	0.000209626
TG 54:0 FA18:0	IL-17A	0.007732023	0.524788316	0.98834226	0.00192451	0.004234951	0.652786984
TG 54:0 FA18:0	IL-1β	0.311235872	0.375023355	0.413144888	0.080317002	0.029752508	0.011297959
TG 54:0 FA18:0	IL-6	0.178005028	0.294066069	0.549516413	0.018692239	0.003350133	4.5382E-06
TG 54:0 FA18:0	MCP1	0.09134426	0.294662083	0.758706758	0.003574886	0.000621332	2.78139E-06
TG 54:0 FA18:0	TNF-α	0.102959066	0.381479373	0.789089582	0.106519935	0.027230278	0.000486279
TG 54:1 FA16:0	IL-17A	0.06336361	0.351906287	0.858317648	0.001831853	0.003889213	0.641041641
TG 54:1 FA16:0	IL-1β	0.28841414	0.281686682	0.3140764	0.083050959	0.030605679	0.010921128
TG 54:1 FA16:0	IL-6	0.217728022	0.22307133	0.336846705	0.018896291	0.003480418	6.93125E-06
TG 54:1 FA16:0	MCP1	0.159912349	0.195967706	0.420924509	0.003641756	0.000565919	4.15996E-07
TG 54:1 FA16:0	TNF-α	0.23088281	0.271389434	0.401651291	0.10913348	0.026530436	0.000279257
TG 54:1 FA18:0	IL-17A	0.03982483	0.04355449	0.367814544	0.001147666	0.003087129	0.712685737
TG 54:1 FA18:0	IL-1β	0.053584368	0.040564721	0.196502668	0.08463609	0.028266448	0.005468812
TG 54:1 FA18:0	IL-6	0.037167225	0.032807213	0.266230457	0.018939556	0.003282797	2.66105E-06
TG 54:1 FA18:0	MCP1	0.028213498	0.032005984	0.385051788	0.003581757	0.000592772	1.23858E-06
TG 54:1 FA18:0	TNF-α	0.037902583	0.04402807	0.396136291	0.102033744	0.027603754	0.000873102
TG 54:1 FA18:1	IL-17A	0.073574989	0.085952484	0.398785563	0.00117196	0.003053099	0.703789931
TG 54:1 FA18:1	IL-1β	0.101602755	0.084276785	0.237401024	0.082423223	0.029430077	0.008839138
TG 54:1 FA18:1	IL-6	0.07121144	0.066293631	0.291305275	0.018997675	0.003324353	3.10259E-06
TG 54:1 FA18:1	MCP1	0.05493412	0.062264763	0.384647557	0.00358649	0.000577909	7.85109E-07
TG 54:1 FA18:1	TNF-α	0.075937634	0.087825522	0.394096679	0.105236103	0.027594335	0.000635482
TG 54:1 FA20:0	IL-17A	0.007401362	0.31876318	0.981629338	0.001927583	0.004116736	0.643003676
TG 54:1 FA20:0	IL-1β	0.207830755	0.23976344	0.392923272	0.082491668	0.030441693	0.011020482
TG 54:1 FA20:0	IL-6	0.153958683	0.185753059	0.413743695	0.01892386	0.003386677	4.43476E-06
TG 54:1 FA20:0	MCP1	0.107329679	0.172657929	0.538879539	0.003633381	0.000582648	7.22284E-07
TG 54:1 FA20:0	TNF-α	0.11390352	0.230589694	0.62492752	0.10860039	0.02634156	0.000272201

TG 54:1 FA20:1	IL-17A	0.44990962	0.571590996	0.437387264	0.001645331	0.002848056	0.567775668
TG 54:1 FA20:1	IL-1β	1.027097219	0.631733167	0.114444272	0.094789442	0.030945526	0.00459584
TG 54:1 FA20:1	IL-6	0.748147513	0.464236661	0.117529246	0.019041319	0.003265544	2.23826E-06
TG 54:1 FA20:1	MCP1	0.399053977	0.45486664	0.387296089	0.003643043	0.000592219	9.13584E-07
TG 54:1 FA20:1	TNF-α	0.809149387	0.566576731	0.163580952	0.108358804	0.024971157	0.00014916
TG 54:2 FA16:0	IL-17A	0.002621542	0.094715056	0.978102119	0.001928877	0.004102342	0.641620239
TG 54:2 FA16:0	IL-1β	0.080831641	0.07287125	0.276140132	0.08842407	0.031029165	0.007836205
TG 54:2 FA16:0	IL-6	0.066348596	0.052785696	0.218472901	0.019283684	0.003227617	1.49695E-06
TG 54:2 FA16:0	MCP1	0.024339408	0.051748669	0.64151452	0.003614022	0.000585662	8.65744E-07
TG 54:2 FA16:0	TNF-α	0.047047362	0.070361902	0.508832666	0.108487791	0.026956729	0.00035695
TG 54:2 FA18:0	IL-17A	0.009512236	0.011017383	0.39477654	0.001326065	0.003023841	0.664139284
TG 54:2 FA18:0	IL-1β	0.014603702	0.010628536	0.179618656	0.087490979	0.028678399	0.004741861
TG 54:2 FA18:0	IL-6	0.010930668	0.00837574	0.201801298	0.019093666	0.003245313	1.932E-06
TG 54:2 FA18:0	MCP1	0.006992619	0.00795309	0.38626351	0.003652091	0.000570363	4.54487E-07
TG 54:2 FA18:0	TNF-α	0.011530777	0.010846614	0.29623017	0.107225568	0.026332457	0.000313185
TG 54:2 FA18:1	IL-17A	0.005999572	0.00698609	0.397267437	0.001340867	0.002991724	0.657234821
TG 54:2 FA18:1	IL-1β	0.009694747	0.006682798	0.157239366	0.087323561	0.028134988	0.004144847
TG 54:2 FA18:1	IL-6	0.006993309	0.005310279	0.19783092	0.019144742	0.003210389	1.54466E-06
TG 54:2 FA18:1	MCP1	0.004405742	0.005061386	0.390960378	0.003650993	0.000566359	4.03203E-07
TG 54:2 FA18:1	TNF-α	0.008264161	0.006820085	0.235070528	0.10671479	0.025834137	0.000266254
TG 54:2 FA18:2	IL-17A	0.006365873	0.300479312	0.983237785	0.001916776	0.004182263	0.650031998
TG 54:2 FA18:2	IL-1β	0.197099967	0.236230162	0.41067553	0.080122803	0.032324595	0.019033608
TG 54:2 FA18:2	IL-6	0.147579774	0.172981625	0.400335167	0.019200436	0.003398988	3.73364E-06
TG 54:2 FA18:2	MCP1	0.098978019	0.162241477	0.546411858	0.003677086	0.000590056	7.30814E-07
TG 54:2 FA18:2	TNF-α	0.148296693	0.223217564	0.511534825	0.110199982	0.02748159	0.000371536
TG 54:2 FA20:0	IL-17A	0.008203838	0.656779012	0.990116568	0.001930625	0.00411219	0.642115232
TG 54:2 FA20:0	IL-1β	0.321888053	0.505968	0.529481577	0.078176289	0.031144254	0.017693408
TG 54:2 FA20:0	IL-6	0.293614376	0.379391605	0.445041199	0.01939451	0.003353471	2.5579E-06
TG 54:2 FA20:0	MCP1	0.141531639	0.364096944	0.700228781	0.003647903	0.00059567	9.86278E-07
TG 54:2 FA20:0	TNF-α	0.153874277	0.472008713	0.746688446	0.109941173	0.026140907	0.000216332
TG 54:2 FA20:1	IL-17A	0.003237334	0.086526321	0.970402422	0.001931228	0.004082939	0.639637058
TG 54:2 FA20:1	IL-1β	0.077705644	0.064764989	0.23960327	0.089180207	0.030044567	0.005836912
TG 54:2 FA20:1	IL-6	0.068430362	0.046612505	0.152493065	0.019381894	0.003105131	7.10515E-07
TG 54:2 FA20:1	MCP1	0.024757623	0.047515019	0.606158221	0.003609	0.000585855	8.91701E-07
TG 54:2 FA20:1	TNF-α	0.06408606	0.063326159	0.319631132	0.106834342	0.026431667	0.000340262
TG 54:2 FA20:2	IL-17A	0.916277036	1.433085148	0.527432267	0.001616998	0.003294512	0.627127925
TG 54:2 FA20:2	IL-1β	2.064565282	1.429836822	0.159125368	0.094341471	0.032315174	0.006593957
TG 54:2 FA20:2	IL-6	1.584750756	1.008333935	0.126519686	0.01901289	0.003272475	2.37435E-06
TG 54:2 FA20:2	MCP1	0.665053091	1.005408607	0.513356301	0.003585615	0.000603944	1.66295E-06
TG 54:2 FA20:2	TNF-α	1.790338807	1.250771324	0.162655137	0.110444731	0.025433943	0.000147894
TG 54:3 FA16:0	IL-17A	0.000504056	0.074369073	0.994637038	0.001936059	0.004098268	0.640053458
TG 54:3 FA16:0	IL-1β	0.049338971	0.058475878	0.405487484	0.08428341	0.03168004	0.012407612
TG 54:3 FA16:0	IL-6	0.037602739	0.041997467	0.377724202	0.01931154	0.003267262	1.79045E-06
TG 54:3 FA16:0	MCP1	0.004712512	0.042218967	0.911867626	0.003656003	0.000607926	1.34116E-06
TG 54:3 FA16:0	TNF-α	0.018420853	0.053585028	0.733418081	0.108641185	0.026119699	0.000245996
TG 54:3 FA16:1	IL-17A	0.015869807	0.799527541	0.984295274	0.001942622	0.004090635	0.638301079
TG 54:3 FA16:1	IL-1β	0.596412666	0.602004935	0.329750791	0.084988594	0.030280175	0.0087083
TG 54:3 FA16:1	IL-6	0.6115548	0.446574838	0.181028562	0.019319795	0.003225555	1.4354E-06
TG 54:3 FA16:1	MCP1	0.116331392	0.45136465	0.79837305	0.003629698	0.00060342	1.33625E-06
TG 54:3 FA16:1	TNF-α	0.31468521	0.595218205	0.600911274	0.10656839	0.026937066	0.000430632

TG 54:3 FA18:0	IL-17A	0.001061341	0.017993862	0.953356412	0.001909326	0.004081187	0.6432828
TG 54:3 FA18:0	IL-1β	0.015109901	0.012837312	0.248438028	0.087611846	0.028624451	0.004623562
TG 54:3 FA18:0	IL-6	0.012530413	0.009887319	0.214789923	0.019486314	0.003165873	9.04471E-07
TG 54:3 FA18:0	MCP1	0.006837371	0.009688898	0.485824305	0.003634697	0.000574211	5.5657E-07
TG 54:3 FA18:0	TNF-α	0.011656725	0.013470583	0.393716016	0.108565622	0.027024968	0.000364174
TG 54:3 FA18:1	IL-17A	0.001427355	0.001754202	0.422241009	0.001576398	0.003018238	0.605303565
TG 54:3 FA18:1	IL-1β	0.002329499	0.001565954	0.147295094	0.086155518	0.026488279	0.002826991
TG 54:3 FA18:1	IL-6	0.00207461	0.001218966	0.099108951	0.019435055	0.002960862	2.91617E-07
TG 54:3 FA18:1	MCP1	0.001035116	0.001295798	0.430671148	0.0036555	0.000582567	6.48446E-07
TG 54:3 FA18:1	TNF-α	0.001995748	0.001699914	0.249619454	0.109093322	0.025871252	0.00020979
TG 54:3 FA18:2	IL-17A	0.001304382	0.021139019	0.951207075	0.001907802	0.004075326	0.643071578
TG 54:3 FA18:2	IL-1β	0.017835897	0.01510468	0.246951984	0.087575172	0.028627938	0.004642986
TG 54:3 FA18:2	IL-6	0.014669516	0.011645136	0.217485613	0.019477703	0.003169385	9.28818E-07
TG 54:3 FA18:2	MCP1	0.008347907	0.011464473	0.47216475	0.003634119	0.000577519	6.17154E-07
TG 54:3 FA18:2	TNF-α	0.014380634	0.01586529	0.371936099	0.108018937	0.027054674	0.000389672
TG 54:3 FA18:3	IL-17A	0.074571829	2.660660291	0.977825786	0.001925644	0.004109363	0.642743315
TG 54:3 FA18:3	IL-1β	1.809386496	1.967858128	0.365184212	0.079528828	0.029879947	0.012373403
TG 54:3 FA18:3	IL-6	1.314929658	1.568876768	0.408583522	0.019388915	0.003420793	3.53839E-06
TG 54:3 FA18:3	MCP1	0.975279153	1.443868989	0.504555652	0.003688606	0.000582704	5.56175E-07
TG 54:3 FA18:3	TNF-α	1.181200619	1.991768219	0.55759825	0.109677057	0.027210754	0.000350973
TG 54:3 FA20:1	IL-17A	0.000413076	0.141959035	0.99769756	0.001939093	0.004095697	0.639321841
TG 54:3 FA20:1	IL-1β	0.074984	0.116087242	0.523236565	0.080581188	0.032926854	0.020465292
TG 54:3 FA20:1	IL-6	0.051487754	0.082999863	0.539721164	0.019230445	0.00338061	3.34018E-06
TG 54:3 FA20:1	MCP1	-0.002451301	0.081634246	0.976243715	0.003721016	0.000615421	1.22511E-06
TG 54:3 FA20:1	TNF-α	1.53616E-10	0.107565907	0.999999999	0.109289539	0.027450871	0.000401998
TG 54:3 FA20:2	IL-17A	0.001906634	0.101219625	0.985096174	0.001940142	0.00409264	0.638891924
TG 54:3 FA20:2	IL-1β	0.07714841	0.078057238	0.330882849	0.087365041	0.031027946	0.008519492
TG 54:3 FA20:2	IL-6	0.07209438	0.054405403	0.195131748	0.019447123	0.003105516	6.71694E-07
TG 54:3 FA20:2	MCP1	0.009579236	0.057682768	0.869217955	0.003646122	0.000609424	1.46257E-06
TG 54:3 FA20:2	TNF-α	0.032222564	0.073385056	0.663743547	0.107289453	0.026246006	0.000299785
TG 54:3 FA20:3	IL-17A	0.017067382	1.050256559	0.987142002	0.001927827	0.00413222	0.644202611
TG 54:3 FA20:3	IL-1β	0.827438792	0.822722563	0.322587858	0.088528994	0.031823097	0.009252987
TG 54:3 FA20:3	IL-6	0.611912837	0.598690993	0.314912657	0.019456464	0.003325398	2.11683E-06
TG 54:3 FA20:3	MCP1	0.210838054	0.587055288	0.722002047	0.003620065	0.000603534	1.40166E-06
TG 54:3 FA20:3	TNF-α	0.342715642	0.757225416	0.654097267	0.10882554	0.026352994	0.000267164
TG 54:4 FA16:0	IL-17A	0.000537644	0.0623796	0.993180259	0.001937174	0.004092841	0.639419648
TG 54:4 FA16:0	IL-1β	0.040008886	0.048768058	0.418464287	0.086931276	0.031457108	0.009677599
TG 54:4 FA16:0	IL-6	0.034440456	0.034457397	0.325542702	0.019337603	0.003191668	1.18316E-06
TG 54:4 FA16:0	MCP1	-0.002362069	0.036831648	0.949290951	0.003728928	0.000631449	1.81707E-06
TG 54:4 FA16:0	TNF-α	0.001327819	0.048338387	0.978267384	0.108869891	0.02805377	0.000529354
TG 54:4 FA16:1	IL-17A	0.00319931	0.591269623	0.995718557	0.001939157	0.004089253	0.638784911
TG 54:4 FA16:1	IL-1β	0.262110234	0.485532967	0.593292155	0.078658213	0.033012474	0.023723465
TG 54:4 FA16:1	IL-6	0.275885852	0.34450393	0.429532433	0.019206342	0.003363608	3.14348E-06
TG 54:4 FA16:1	MCP1	-0.034322298	0.34638125	0.921727394	0.00373141	0.000625961	1.55457E-06
TG 54:4 FA16:1	TNF-α	5.04004E-13	0.450927663	1	0.109289156	0.027585571	0.000424041
TG 54:4 FA18:0	IL-17A	0.001668437	0.048829354	0.972968932	0.001935699	0.004084087	0.638958907
TG 54:4 FA18:0	IL-1β	0.043746642	0.035207594	0.223664522	0.083867286	0.028950182	0.006972438
TG 54:4 FA18:0	IL-6	0.043010608	0.027369817	0.126564113	0.019927659	0.003231757	8.76986E-07
TG 54:4 FA18:0	MCP1	0.01281844	0.027221572	0.641123458	0.0036142	0.000594925	1.13062E-06
TG 54:4 FA18:0	TNF-α	0.028894102	0.038078938	0.453896055	0.110285558	0.028171861	0.000482408

TG 54:4 FA18:1	IL-17A	0.000204296	0.003575213	0.954810691	0.001940879	0.004055499	0.635707265
TG 54:4 FA18:1	IL-1β	0.003220218	0.002530698	0.212983215	0.086545657	0.028221737	0.004554937
TG 54:4 FA18:1	IL-6	0.00294177	0.0019775	0.147287535	0.019724984	0.003166735	7.36824E-07
TG 54:4 FA18:1	MCP1	0.000980109	0.00200099	0.627829697	0.00362528	0.000593092	1.01847E-06
TG 54:4 FA18:1	TNF-α	0.002492174	0.00263661	0.352098399	0.107863514	0.026454835	0.000308658
TG 54:4 FA18:2	IL-17A	0.000284958	0.006206571	0.96368447	0.001942346	0.004066839	0.63639228
TG 54:4 FA18:2	IL-1β	0.005855876	0.004404676	0.19371525	0.086683626	0.028373973	0.004690765
TG 54:4 FA18:2	IL-6	0.005383055	0.003422447	0.126238014	0.019746191	0.003165883	7.19871E-07
TG 54:4 FA18:2	MCP1	0.001721659	0.003440862	0.620474674	0.003628303	0.000589125	8.95238E-07
TG 54:4 FA18:2	TNF-α	0.004304985	0.004752789	0.372269546	0.109363127	0.027546756	0.000414532
TG 54:4 FA18:3	IL-17A	0.006703334	0.259841879	0.979589504	0.001938583	0.004093555	0.639235059
TG 54:4 FA18:3	IL-1β	0.183558623	0.190324532	0.342530553	0.078567531	0.029477265	0.012262923
TG 54:4 FA18:3	IL-6	0.134284098	0.14664806	0.367132007	0.019417268	0.003261524	1.58821E-06
TG 54:4 FA18:3	MCP1	0.080362662	0.144792828	0.582997219	0.003674721	0.000596037	8.79295E-07
TG 54:4 FA18:3	TNF-α	0.130872129	0.195644006	0.508655468	0.109056979	0.027263067	0.000381664
TG 54:4 FA20:1	IL-17A	1.53965E-07	0.852440292	0.999999857	0.001940617	0.004232609	0.65001669
TG 54:4 FA20:1	IL-1β	0.26138575	0.682721838	0.704616772	0.084107278	0.034268108	0.020353049
TG 54:4 FA20:1	IL-6	0.365956664	0.459963892	0.432717139	0.019572723	0.003275841	1.70652E-06
TG 54:4 FA20:1	MCP1	-0.028429162	0.4347849	0.948314769	0.003774815	0.000572219	3.14268E-07
TG 54:4 FA20:1	TNF-α	0.020124515	0.585313551	0.972807745	0.11465414	0.026899683	0.000195446
TG 54:4 FA20:2	IL-17A	0.000146147	0.148834014	0.999223022	0.001940204	0.004089523	0.638626561
TG 54:4 FA20:2	IL-1β	0.044834688	0.125807466	0.724053678	0.077659129	0.033984264	0.029543794
TG 54:4 FA20:2	IL-6	0.035896864	0.09208439	0.699419341	0.019196098	0.003571983	8.1005E-06
TG 54:4 FA20:2	MCP1	-0.007231108	0.085985734	0.933538307	0.003733584	0.00061735	1.22013E-06
TG 54:4 FA20:2	TNF-α	-0.008163802	0.113675234	0.943224287	0.108380321	0.02762823	0.000471875
TG 54:4 FA20:3	IL-17A	0.001624151	0.076028611	0.983098049	0.001943907	0.004097625	0.638650805
TG 54:4 FA20:3	IL-1β	0.059464546	0.054554107	0.284386776	0.084807886	0.028905704	0.00635954
TG 54:4 FA20:3	IL-6	0.055668142	0.039056024	0.164380831	0.019302606	0.002971639	3.5204E-07
TG 54:4 FA20:3	MCP1	3.22844E-06	0.044792174	0.999942969	0.003708488	0.000630801	1.9562E-06
TG 54:4 FA20:3	TNF-α	0.023292547	0.056774277	0.684526264	0.104283385	0.027065961	0.000571091
TG 54:4 FA20:4	IL-1β	0.156555675	0.300473653	0.606171119	0.078121221	0.032761939	0.023625006
TG 54:4 FA20:4	IL-6	0.117882327	0.214099501	0.585987701	0.019349764	0.003352206	2.63938E-06
TG 54:4 FA20:4	MCP1	-0.023591564	0.215693294	0.913633012	0.00372855	0.000625077	1.53789E-06
TG 54:4 FA20:4	TNF-α	-0.031681574	0.28760494	0.913018822	0.107377617	0.028214691	0.000649363
TG 54:4 FA22:1	IL-17A	0.839930309	0.830180749	0.320324117	0.00205111	0.003038826	0.505232048
TG 54:4 FA22:1	IL-1β	1.196622796	0.769217149	0.131026288	0.072327728	0.031608401	0.029877063
TG 54:4 FA22:1	IL-6	1.243051671	0.500489525	0.019258435	0.020446799	0.002635202	1.88066E-08
TG 54:4 FA22:1	MCP1	0.612720046	0.534018641	0.260936014	0.003727747	0.000518935	8.09735E-08
TG 54:4 FA22:1	TNF-α	1.300280579	0.706524791	0.076333173	0.122661086	0.023939043	1.97655E-05
TG 54:4 FA22:4	IL-17A	-5.23684E-16	1.092841256	1	1.35175E-17	0.004543629	1
TG 54:4 FA22:4	IL-1β	-0.086453116	0.856259955	0.920272307	0.067708386	0.035678161	0.067724048
TG 54:4 FA22:4	IL-6	-0.18077631	0.661474795	0.786565066	0.018498747	0.003954879	6.2126E-05
TG 54:4 FA22:4	MCP1	-0.324295632	0.597156222	0.591233984	0.003581875	0.000659531	7.66192E-06
TG 54:4 FA22:4	TNF-α	-0.435854669	0.704745048	0.541101555	0.109097268	0.025905894	0.000224785
TG 54:5 FA16:0	IL-17A	-3.15977E-17	0.071452154	1	3.1885E-17	0.004288468	1
TG 54:5 FA16:0	IL-1β	0.018481314	0.055486654	0.74139298	0.078576776	0.032739824	0.022804534
TG 54:5 FA16:0	IL-6	0.014130659	0.041582702	0.736359394	0.019333841	0.003523323	5.88411E-06
TG 54:5 FA16:0	MCP1	-0.015016872	0.039972308	0.709796838	0.003784853	0.000626875	1.25505E-06
TG 54:5 FA16:0	TNF-α	-0.009346083	0.052815983	0.860732564	0.108304695	0.028039413	0.000556258
TG 54:5 FA16:1	IL-17A	0.003145747	0.44405057	0.994394566	0.001943329	0.004090192	0.638142883

TG 54:5 FA16:1	IL-1β	0.252377391	0.353239463	0.480462267	0.081510768	0.031987586	0.016187242
TG 54:5 FA16:1	IL-6	0.256620937	0.244697134	0.302678648	0.0191854	0.003181948	1.28413E-06
TG 54:5 FA16:1	MCP1	-0.03928113	0.2655965	0.883413032	0.003718552	0.000639247	2.32723E-06
TG 54:5 FA16:1	TNF-α	6.60387E-11	0.340918164	1	0.109084639	0.027776539	0.000466212
TG 54:5 FA18:0	IL-17A	0.006108325	0.372687064	0.987031811	0.001941187	0.004093778	0.638804858
TG 54:5 FA18:0	IL-1β	0.188200803	0.27215209	0.494551017	0.074357378	0.029389526	0.016889472
TG 54:5 FA18:0	IL-6	0.191885802	0.21682896	0.383214382	0.019485388	0.003362406	2.47553E-06
TG 54:5 FA18:0	MCP1	0.075870621	0.21053544	0.721095543	0.003628818	0.000604282	1.37428E-06
TG 54:5 FA18:0	TNF-α	0.088926474	0.270092004	0.74425809	0.110265862	0.026242692	0.000218709
TG 54:5 FA18:1	IL-17A	0.000189508	0.009582959	0.984353372	0.001948967	0.004095374	0.637596808
TG 54:5 FA18:1	IL-1β	0.006261167	0.007243643	0.394245226	0.076229105	0.030433502	0.017915536
TG 54:5 FA18:1	IL-6	0.006222402	0.005580273	0.27367309	0.019763692	0.003366683	2.00416E-06
TG 54:5 FA18:1	MCP1	0.002036267	0.005374928	0.707470762	0.003601032	0.000600208	1.39566E-06
TG 54:5 FA18:1	TNF-α	0.003084353	0.007179929	0.670571028	0.10953844	0.027141311	0.000345979
TG 54:5 FA18:2	IL-17A	0.000162649	0.008208163	0.984321768	0.00194086	0.004094851	0.63894873
TG 54:5 FA18:2	IL-1β	0.006207577	0.006245985	0.328244524	0.080149636	0.030633315	0.01378033
TG 54:5 FA18:2	IL-6	0.006047402	0.004850829	0.222163567	0.019881775	0.003416339	2.31078E-06
TG 54:5 FA18:2	MCP1	0.001987662	0.004646991	0.671901847	0.003607421	0.000605757	1.58028E-06
TG 54:5 FA18:2	TNF-α	0.003481742	0.006247912	0.581480896	0.109899535	0.027570444	0.00039667
TG 54:5 FA18:3	IL-17A	0.001236357	0.044116435	0.977827927	0.001955551	0.004091463	0.636143949
TG 54:5 FA18:3	IL-1β	0.025092673	0.034116517	0.46774715	0.073570788	0.031105979	0.024685016
TG 54:5 FA18:3	IL-6	0.028349777	0.02417655	0.250177821	0.019483746	0.003165377	9.04085E-07
TG 54:5 FA18:3	MCP1	0.00949932	0.024295623	0.69856845	0.003602681	0.000588764	1.00004E-06
TG 54:5 FA18:3	TNF-α	0.01669078	0.032479534	0.611096266	0.109073027	0.026644325	0.000294999
TG 54:5 FA20:2	IL-17A	0.000683354	0.768168378	0.9992961	0.001940505	0.00409818	0.639281171
TG 54:5 FA20:2	IL-1β	0.183306517	0.632093144	0.77381191	0.077869067	0.033152526	0.02561458
TG 54:5 FA20:2	IL-6	0.075450749	0.4944715	0.879744315	0.018991921	0.003724163	1.75824E-05
TG 54:5 FA20:2	MCP1	-0.040848562	0.438973125	0.926478692	0.00372818	0.000611938	1.07716E-06
TG 54:5 FA20:2	TNF-α	-0.0459827	0.593318617	0.938739707	0.108523933	0.02799879	0.000536241
TG 54:5 FA20:3	IL-17A	0.00131619	0.105341687	0.990113824	0.001937064	0.004088564	0.639089389
TG 54:5 FA20:3	IL-1β	0.074095758	0.081888609	0.37276184	0.080548171	0.031246039	0.015096356
TG 54:5 FA20:3	IL-6	0.062592572	0.055577777	0.269002928	0.019402949	0.003045261	4.95976E-07
TG 54:5 FA20:3	MCP1	-0.007071093	0.062582959	0.910793149	0.003735476	0.00063469	1.9209E-06
TG 54:5 FA20:3	TNF-α	0.015770402	0.078730839	0.842591586	0.106205583	0.027029132	0.000463566
TG 54:5 FA20:4	IL-17A	-0.000365054	0.02996121	0.990359302	0.001944839	0.004086673	0.63759447
TG 54:5 FA20:4	IL-1β	-0.011495575	0.026001748	0.661582098	0.066836537	0.034866956	0.064818561
TG 54:5 FA20:4	IL-6	-0.001289327	0.020074955	0.94921661	0.018870675	0.003865609	3.25328E-05
TG 54:5 FA20:4	MCP1	-0.008497779	0.017812426	0.636769208	0.003715536	0.000634846	2.10623E-06
TG 54:5 FA20:4	TNF-α	-0.008063761	0.022986993	0.728192336	0.106409404	0.027733801	0.000596729
TG 54:5 FA20:5	IL-17A	-0.046202092	2.32374408	0.984268672	0.001945282	0.00408624	0.637482702
TG 54:5 FA20:5	IL-1β	-1.322519822	1.981851607	0.509671664	0.075712121	0.034261606	0.034890549
TG 54:5 FA20:5	IL-6	0.602830045	1.420733904	0.674367961	0.019236419	0.003526966	6.46327E-06
TG 54:5 FA20:5	MCP1	-0.041091566	1.321278771	0.975395898	0.003715201	0.000607107	9.98763E-07
TG 54:5 FA20:5	TNF-α	-0.290346988	1.757124339	0.869863654	0.107068379	0.027330956	0.000478813
TG 54:5 FA22:1	IL-17A	0.022316347	1.063880599	0.983403408	0.001945167	0.004095518	0.638262164
TG 54:5 FA22:1	IL-1β	0.722829513	0.794266582	0.370045593	0.077823126	0.030059603	0.014706258
TG 54:5 FA22:1	IL-6	0.789966318	0.609869972	0.205095135	0.019802748	0.003314409	1.49629E-06
TG 54:5 FA22:1	MCP1	0.22866549	0.595890007	0.703879074	0.003600424	0.0005994	1.36837E-06
TG 54:5 FA22:1	TNF-α	0.341024255	0.803048059	0.6741131	0.109387619	0.027344767	0.000381518
TG 54:5 FA22:4	IL-17A	0.002821993	0.827044001	0.997300873	0.001938097	0.004307803	0.656124921

TG 54:5 FA22:4	IL-1β	0.229797027	0.648554281	0.725661117	0.075548529	0.033855144	0.033538886
TG 54:5 FA22:4	IL-6	0.150660634	0.506354304	0.76817541	0.018956861	0.003792765	2.54961E-05
TG 54:5 FA22:4	MCP1	-0.181812131	0.4720333	0.702923579	0.003635118	0.000653133	5.27477E-06
TG 54:5 FA22:4	TNF-α	-0.112432089	0.5970145	0.85193442	0.108735287	0.027493684	0.000452101
TG 54:5 FA22:5	IL-17A	1.17401E-15	0.454167835	1	1.34032E-17	0.004294369	1
TG 54:5 FA22:5	IL-1β	0.211136776	0.343236386	0.543103833	0.084057265	0.03190636	0.01320058
TG 54:5 FA22:5	IL-6	0.040908056	0.281142122	0.885284105	0.018918988	0.003752855	2.07366E-05
TG 54:5 FA22:5	MCP1	-0.028466953	0.254170475	0.911569884	0.003735444	0.000627976	1.61079E-06
TG 54:5 FA22:5	TNF-α	-0.052298597	0.333029522	0.876266313	0.10837	0.02785367	0.00051519
TG 54:6 FA16:0	IL-17A	-1.80505E-16	0.118003864	1	7.13509E-17	0.004295827	1
TG 54:6 FA16:0	IL-1β	-0.049684808	0.095859172	0.608043688	0.070399687	0.034307191	0.048981799
TG 54:6 FA16:0	IL-6	-3.86799E-10	0.076262311	0.999999996	0.018957346	0.003919344	3.69158E-05
TG 54:6 FA16:0	MCP1	-0.030543664	0.065980799	0.646764069	0.003697264	0.000627629	1.89243E-06
TG 54:6 FA16:0	TNF-α	-0.027868978	0.085913006	0.747896847	0.106166726	0.027664712	0.000595415
TG 54:6 FA16:1	IL-17A	0.006972888	0.369605435	0.985077397	0.001956907	0.003046471	0.525690596
TG 54:6 FA16:1	IL-1β	0.180417736	0.382341202	0.640546417	0.050618522	0.034312419	0.150927569
TG 54:6 FA16:1	IL-6	0.286929237	0.237728127	0.237199263	0.019817407	0.00277273	7.25114E-08
TG 54:6 FA16:1	MCP1	-0.029066464	0.288371478	0.920406674	0.003744128	0.000621847	1.50371E-06
TG 54:6 FA16:1	TNF-α	0.008385971	0.358971757	0.98152225	0.108771506	0.026169938	0.000261262
TG 54:6 FA18:1	IL-17A	0.000346823	0.049175875	0.99441949	0.001940959	0.004112681	0.64038083
TG 54:6 FA18:1	IL-1β	0.012510918	0.039145191	0.751484468	0.078175013	0.032184916	0.021346292
TG 54:6 FA18:1	IL-6	0.023228142	0.028640316	0.423736612	0.019355883	0.003381455	3.02144E-06
TG 54:6 FA18:1	MCP1	0.006378029	0.027027178	0.815047438	0.003658113	0.000590621	8.12426E-07
TG 54:6 FA18:1	TNF-α	0.001139814	0.037701922	0.976082063	0.109053802	0.027890301	0.000488575
TG 54:6 FA18:2	IL-17A	0.000103114	0.018621218	0.99561843	0.001939647	0.00410814	0.640238111
TG 54:6 FA18:2	IL-1β	0.008449732	0.015096141	0.579820594	0.075004697	0.032741911	0.029175153
TG 54:6 FA18:2	IL-6	0.006693287	0.011360706	0.560163989	0.019385535	0.003538305	6.02899E-06
TG 54:6 FA18:2	MCP1	0.001467088	0.010540026	0.890228585	0.003664214	0.000607594	1.27968E-06
TG 54:6 FA18:2	TNF-α	0.000544654	0.014116045	0.969477644	0.109379358	0.027546527	0.000413825
TG 54:6 FA18:3	IL-17A	0.000447943	0.048820655	0.992740035	0.001943121	0.004114141	0.640128245
TG 54:6 FA18:3	IL-1β	0.017304738	0.03919228	0.661992417	0.076045645	0.032469613	0.026007859
TG 54:6 FA18:3	IL-6	0.025707476	0.027943559	0.364922694	0.019429854	0.003324377	2.15399E-06
TG 54:6 FA18:3	MCP1	0.007623346	0.026923293	0.779006144	0.003650134	0.000592842	8.99701E-07
TG 54:6 FA18:3	TNF-α	0.011274165	0.035802576	0.755018846	0.109216256	0.026687421	0.000296014
TG 54:6 FA20:3	IL-17A	0.004846728	0.605572464	0.993667148	0.001943992	0.0041046	0.639204391
TG 54:6 FA20:3	IL-1β	0.282649901	0.47529519	0.556514497	0.075958171	0.031671532	0.022894447
TG 54:6 FA20:3	IL-6	0.296707766	0.327579196	0.372280939	0.019511325	0.00313454	7.45446E-07
TG 54:6 FA20:3	MCP1	-0.071592045	0.354970353	0.841524687	0.003706224	0.000628683	1.86937E-06
TG 54:6 FA20:3	TNF-α	-7.06199E-11	0.460653729	1	0.10928898	0.027618239	0.000429534
TG 54:6 FA20:4	IL-17A	-0.001189671	0.040208478	0.976591945	0.001951926	0.00408622	0.636336682
TG 54:6 FA20:4	IL-1β	-0.024035568	0.03448991	0.491238796	0.067594591	0.034458562	0.059144619
TG 54:6 FA20:4	IL-6	-0.005265474	0.027415542	0.84898807	0.018520316	0.003933271	5.29801E-05
TG 54:6 FA20:4	MCP1	-0.018630034	0.023817629	0.440230373	0.003622012	0.000632466	2.99879E-06
TG 54:6 FA20:4	TNF-α	-0.023024611	0.02927051	0.437677514	0.10469627	0.026311834	0.000404456
TG 54:6 FA20:5	IL-17A	1.9387E-16	0.152938772	1	7.14614E-17	0.004297643	1
TG 54:6 FA20:5	IL-1β	-0.115035177	0.121049734	0.349546737	0.07668291	0.033440872	0.02902566
TG 54:6 FA20:5	IL-6	0.039513688	0.086587588	0.651429668	0.019393198	0.003434957	3.76565E-06
TG 54:6 FA20:5	MCP1	-0.027541422	0.086392361	0.752091286	0.003681017	0.000634341	2.42176E-06
TG 54:6 FA20:5	TNF-α	-0.026175917	0.112576524	0.817715747	0.106671467	0.027981909	0.000638125
TG 54:6 FA22:5	IL-17A	0.005098753	0.358943883	0.988760579	0.001949095	0.004091794	0.637281504

TG 54:6 FA22:5	IL-1β	0.206890786	0.256680122	0.426571353	0.07993444	0.028766062	0.009323963
TG 54:6 FA22:5	IL-6	0.179793208	0.193070919	0.359169121	0.019468231	0.003107112	6.65056E-07
TG 54:6 FA22:5	MCP1	-0.010983264	0.211219033	0.958873876	0.003718921	0.000629151	1.78848E-06
TG 54:6 FA22:5	TNF-α	2.28308E-12	0.276022053	1	0.108623375	0.027832211	0.000498429
TG 54:6 FA22:6	IL-17A	-0.006623031	0.243256466	0.97845936	0.001955398	0.004082353	0.635420201
TG 54:6 FA22:6	IL-1β	-0.185274421	0.208345746	0.380936582	0.073092627	0.034374137	0.041812464
TG 54:6 FA22:6	IL-6	0.055161266	0.1523203	0.719786047	0.019432136	0.003608751	7.86056E-06
TG 54:6 FA22:6	MCP1	-0.047082601	0.145435266	0.748384228	0.00368278	0.00063775	2.62171E-06
TG 54:6 FA22:6	TNF-α	-0.050557228	0.192656822	0.79479044	0.106117046	0.028598767	0.000840341
TG 54:7 FA16:1	IL-17A	1.60724E-14	0.938034126	1	2.59651E-16	0.004560387	1
TG 54:7 FA16:1	IL-1β	-0.843639916	0.677771686	0.223198113	0.057050939	0.033023076	0.094695795
TG 54:7 FA16:1	IL-6	0.035523108	0.592173879	0.952576979	0.018824369	0.004140058	8.91901E-05
TG 54:7 FA16:1	MCP1	-0.351682843	0.515822851	0.500781051	0.003576882	0.00066617	9.09048E-06
TG 54:7 FA16:1	TNF-α	-0.365157146	0.604920211	0.550772548	0.106008567	0.026001711	0.00032447
TG 54:7 FA18:2	IL-17A	5.30588E-05	0.088218646	0.999524096	0.001940581	0.004113622	0.64052191
TG 54:7 FA18:2	IL-1β	-0.002621445	0.072298013	0.971316134	0.071848333	0.033142961	0.038234428
TG 54:7 FA18:2	IL-6	0.02065967	0.055867998	0.714133474	0.01926565	0.003677731	1.18796E-05
TG 54:7 FA18:2	MCP1	0.006242895	0.049625146	0.900728724	0.003695991	0.000604645	1.01804E-06
TG 54:7 FA18:2	TNF-α	-0.004633971	0.06711446	0.945411351	0.108303921	0.027681957	0.000485459
TG 54:7 FA18:3	IL-17A	0.000419806	0.112975601	0.997059745	0.001941184	0.004120353	0.640962532
TG 54:7 FA18:3	IL-1β	0.011077071	0.093917298	0.906897439	0.074398376	0.033674127	0.034925778
TG 54:7 FA18:3	IL-6	0.031556859	0.070662578	0.658381808	0.019220154	0.003638247	1.04812E-05
TG 54:7 FA18:3	MCP1	0.012185327	0.063530877	0.849189922	0.003685986	0.000605438	1.09011E-06
TG 54:7 FA18:3	TNF-α	-7.59264E-07	0.085097325	0.99999294	0.109289484	0.027452532	0.000402266
TG 54:7 FA20:4	IL-1β	-0.195375325	0.246809227	0.434805893	0.067939633	0.036291301	0.070975507
TG 54:7 FA20:4	IL-6	-0.032548788	0.183970961	0.860757537	0.018519896	0.003884564	4.48792E-05
TG 54:7 FA20:4	MCP1	-0.129957754	0.161117212	0.426243288	0.003634382	0.000629676	2.64247E-06
TG 54:7 FA20:4	TNF-α	-0.146278447	0.203349215	0.4774944	0.106674238	0.026902918	0.000420184
TG 54:7 FA20:5	IL-17A	-5.27398E-17	0.220414678	1	7.13679E-17	0.004306323	1
TG 54:7 FA20:5	IL-1β	-0.162272218	0.175975241	0.36381727	0.079486947	0.033800154	0.025449655
TG 54:7 FA20:5	IL-6	0.015631084	0.13729849	0.91011722	0.019138641	0.003786908	2.00077E-05
TG 54:7 FA20:5	MCP1	-0.047105087	0.124219576	0.707204124	0.003706462	0.000634149	2.15324E-06
TG 54:7 FA20:5	TNF-α	-0.049510385	0.161935312	0.76191286	0.106409236	0.027984943	0.000655318
TG 54:7 FA22:5	IL-17A	0.034654396	1.049544227	0.973894073	0.001956599	0.003209813	0.547063162
TG 54:7 FA22:5	IL-1β	0.87257913	1.017240937	0.398291609	0.051499354	0.034575033	0.147535756
TG 54:7 FA22:5	IL-6	0.798264377	0.685253496	0.253881085	0.01949325	0.003022427	5.50753E-07
TG 54:7 FA22:5	MCP1	0.301820671	0.739892315	0.686431152	0.003648159	0.000602064	1.55993E-06
TG 54:7 FA22:5	TNF-α	0.197625208	0.923744941	0.832144817	0.110259861	0.024975283	0.000137019
TG 54:7 FA22:6	IL-17A	2.68639E-16	0.224314849	1	7.13064E-17	0.004291583	1
TG 54:7 FA22:6	IL-1β	-0.143479611	0.175049192	0.418878495	0.075232808	0.032924603	0.029553459
TG 54:7 FA22:6	IL-6	0.076953481	0.124165774	0.540092296	0.019512611	0.003353623	2.31887E-06
TG 54:7 FA22:6	MCP1	-0.042145246	0.12506521	0.738473567	0.003724497	0.000625217	1.57189E-06
TG 54:7 FA22:6	TNF-α	-0.039004503	0.16769915	0.817662258	0.106330597	0.028379651	0.000762063
TG 54:8 FA18:2	IL-17A	0.001322124	0.988775802	0.998941975	0.001940538	0.004117666	0.640856165
TG 54:8 FA18:2	IL-1β	-0.177567715	0.809378565	0.827833922	0.071733918	0.033136448	0.038486165
TG 54:8 FA18:2	IL-6	0.273250913	0.611198087	0.658032644	0.019330337	0.003593256	7.97477E-06
TG 54:8 FA18:2	MCP1	0.100491848	0.565418712	0.860129697	0.003691053	0.000615259	1.39747E-06
TG 54:8 FA18:2	TNF-α	-0.098667661	0.747278757	0.895837437	0.1069662	0.027526605	0.000521945
TG 54:8 FA18:3	IL-17A	-0.001340938	0.590461768	0.998203041	0.001940641	0.004125732	0.641489081
TG 54:8 FA18:3	IL-1β	-0.250725745	0.497257686	0.61779222	0.073730232	0.034157946	0.039014471

TG 54:8 FA18:3	IL-6	0.04599719	0.382963056	0.905197836	0.019085575	0.003777627	2.01E-05
TG 54:8 FA18:3	MCP1	-0.029906529	0.338668926	0.930220254	0.003710509	0.000618329	1.3909E-06
TG 54:8 FA18:3	TNF-α	-0.129413984	0.445403754	0.773391364	0.106405633	0.02752833	0.000552138
TG 54:8 FA20:4	IL-17A	-2.43752102	1.704231488	0.164546511	0.001949867	0.004055083	0.634648157
TG 54:8 FA20:4	IL-1β	-2.874495035	1.475566941	0.062279578	0.050732711	0.040488794	0.221355632
TG 54:8 FA20:4	IL-6	-0.172285181	1.203810413	0.887301543	0.018998506	0.004340332	0.000173877
TG 54:8 FA20:4	MCP1	-0.932688084	0.935157928	0.327782625	0.003632985	0.000614616	3.09823E-06
TG 54:8 FA20:4	TNF-α	-1.345018704	0.92461832	0.157726732	0.11286255	0.020339387	7.94212E-06
TG 54:8 FA20:5	IL-17A	-0.086077246	1.400700755	0.951487118	0.00195654	0.004727223	0.682485605
TG 54:8 FA20:5	IL-1β	-1.264801476	1.026509073	0.229360581	0.04177156	0.039951313	0.305768142
TG 54:8 FA20:5	IL-6	0.557806661	0.637709901	0.390061677	0.020187976	0.003264273	1.81551E-06
TG 54:8 FA20:5	MCP1	-0.225616173	0.693578304	0.747666118	0.003601945	0.000655393	1.03914E-05
TG 54:8 FA20:5	TNF-α	-0.251443071	0.754747648	0.741800037	0.113598609	0.023722236	6.44253E-05
TG 54:8 FA22:6	IL-17A	0.004749119	0.797388779	0.995291745	0.001939354	0.004339921	0.658537345
TG 54:8 FA22:6	IL-1β	-0.006691517	0.626043904	0.991550501	0.055635015	0.03916982	0.166950052
TG 54:8 FA22:6	IL-6	0.41781157	0.390932223	0.294641661	0.020513162	0.003125382	4.86377E-07
TG 54:8 FA22:6	MCP1	0.159162316	0.398032635	0.692396398	0.003809403	0.000585867	5.698E-07
TG 54:8 FA22:6	TNF-α	-0.021267167	0.474627639	0.964590021	0.116961397	0.023899198	4.05006E-05
TG 55:1 FA16:0	IL-17A	4.17792E-16	0.292074283	1	7.17514E-17	0.004295051	1
TG 55:1 FA16:0	IL-1β	-0.149915859	0.247380007	0.549063395	0.075860201	0.035763544	0.042283984
TG 55:1 FA16:0	IL-6	0.063141188	0.178488889	0.725997254	0.019509777	0.003705434	1.10161E-05
TG 55:1 FA16:0	MCP1	-0.052534273	0.166639022	0.754748379	0.003656986	0.000640304	3.13217E-06
TG 55:1 FA16:0	TNF-α	-0.07720194	0.215572485	0.722757308	0.105188541	0.028040453	0.000752645
TG 55:1 FA18:1	IL-17A	6.64849E-16	0.584147947	1	3.21865E-17	0.004316563	1
TG 55:1 FA18:1	IL-1β	-0.070564724	0.454494905	0.877656562	0.071075307	0.033017579	0.039511933
TG 55:1 FA18:1	IL-6	0.168454477	0.328796479	0.612167002	0.019116298	0.003430009	4.61946E-06
TG 55:1 FA18:1	MCP1	-0.053930337	0.327125923	0.870159175	0.003712546	0.000631633	1.96348E-06
TG 55:1 FA18:1	TNF-α	1.29352E-06	0.4196754	0.999997561	0.109289549	0.027431241	0.000398865
TG 55:2 FA18:1	IL-17A	0.292512669	0.343660389	0.401419324	0.001860897	0.002959824	0.534291404
TG 55:2 FA18:1	IL-1β	0.398927364	0.340094815	0.250030089	0.08501238	0.028796345	0.006076406
TG 55:2 FA18:1	IL-6	0.30458824	0.268569375	0.265728232	0.019044913	0.00326547	2.23054E-06
TG 55:2 FA18:1	MCP1	0.20309847	0.254553983	0.431222186	0.003631531	0.000572863	5.42322E-07
TG 55:2 FA18:1	TNF-α	0.29609248	0.347932897	0.401508584	0.107391212	0.026506269	0.000331351
TG 55:2 FA18:2	IL-17A	1.41429E-15	1.291841497	1	7.19067E-17	0.004311532	1
TG 55:2 FA18:2	IL-1β	-0.957462354	1.096259916	0.389388311	0.078878722	0.035969705	0.036203667
TG 55:2 FA18:2	IL-6	0.187592237	0.820448512	0.820695575	0.019384749	0.003865686	2.23578E-05
TG 55:2 FA18:2	MCP1	-0.20760602	0.728051838	0.777486516	0.00364591	0.000634921	2.87096E-06
TG 55:2 FA18:2	TNF-α	-0.324920048	0.964203747	0.738476108	0.104312513	0.028464785	0.000951074
TG 55:3 FA18:1	IL-17A	0.161824092	0.193882277	0.410511612	0.001872041	0.00291924	0.526212192
TG 55:3 FA18:1	IL-1β	0.243641783	0.193964282	0.218768032	0.085395713	0.028711408	0.005749604
TG 55:3 FA18:1	IL-6	0.196661265	0.151766459	0.204920832	0.019254047	0.003225971	1.52287E-06
TG 55:3 FA18:1	MCP1	0.117094381	0.143965098	0.42242454	0.003624252	0.000566401	4.59998E-07
TG 55:3 FA18:1	TNF-α	0.211303164	0.196537443	0.290887997	0.107276637	0.026175427	0.000291192
TG 55:3 FA18:2	IL-17A	0.054484028	1.162404134	0.962926006	0.001944898	0.004058484	0.63525846
TG 55:3 FA18:2	IL-1β	0.865608012	0.900621496	0.34417348	0.082821439	0.030913629	0.011865056
TG 55:3 FA18:2	IL-6	0.686667108	0.70814536	0.339964473	0.019482069	0.003490455	4.513E-06
TG 55:3 FA18:2	MCP1	0.444875574	0.628098761	0.484233127	0.00367478	0.00057302	4.42206E-07
TG 55:3 FA18:2	TNF-α	0.675924574	0.875447442	0.44610087	0.108958047	0.027036689	0.000351596
TG 55:4 FA18:1	IL-17A	0.050463462	0.49588719	0.919621143	0.001922207	0.004002304	0.634515342
TG 55:4 FA18:1	IL-1β	0.437977527	0.354531527	0.22628029	0.086950804	0.028130858	0.004282123

TG 55:4 FA18:1	IL-6	0.357243823	0.282940621	0.216455772	0.01935024	0.003223854	1.3857E-06
TG 55:4 FA18:1	MCP1	0.172489603	0.275025872	0.535288463	0.003658411	0.00058001	5.92235E-07
TG 55:4 FA18:1	TNF-α	0.319170384	0.383873125	0.412290014	0.1084744	0.027405112	0.000428298
TG 55:4 FA18:2	IL-17A	0.083092577	0.690542734	0.905024509	0.001935031	0.003970833	0.629579965
TG 55:4 FA18:2	IL-1β	0.650984243	0.504492004	0.206777594	0.088569225	0.028519788	0.004125871
TG 55:4 FA18:2	IL-6	0.540028015	0.39854085	0.185529599	0.019651076	0.003235316	1.13418E-06
TG 55:4 FA18:2	MCP1	0.296638452	0.385912663	0.448100138	0.003649015	0.000579849	6.16455E-07
TG 55:4 FA18:2	TNF-α	0.496183493	0.532547699	0.358921071	0.109802919	0.027087334	0.000329419
TG 55:5 FA18:1	IL-17A	0.037515742	1.088704026	0.972747165	0.001953793	0.003050605	0.526901613
TG 55:5 FA18:1	IL-1β	0.876848872	1.084956398	0.425562653	0.057491216	0.033100145	0.093019782
TG 55:5 FA18:1	IL-6	1.052036162	0.754238597	0.173659304	0.020103693	0.002990564	2.24281E-07
TG 55:5 FA18:1	MCP1	0.342597966	0.763489879	0.656964332	0.003692198	0.000559695	3.1427E-07
TG 55:5 FA18:1	TNF-α	0.540681702	1.020189466	0.600159312	0.109575705	0.025283699	0.000160551
TG 55:5 FA18:2	IL-17A	0.033125919	1.213408274	0.978401338	0.001945092	0.004097868	0.638466958
TG 55:5 FA18:2	IL-1β	0.958092967	0.959381729	0.32594845	0.088755066	0.031852496	0.009151295
TG 55:5 FA18:2	IL-6	0.858669091	0.709337084	0.235529366	0.019618172	0.003381868	2.43478E-06
TG 55:5 FA18:2	MCP1	0.323229618	0.671823633	0.633919937	0.003646605	0.000592845	9.14832E-07
TG 55:5 FA18:2	TNF-α	0.643498243	0.921203801	0.490219247	0.111387866	0.02751842	0.000334824
TG 55:5 FA20:4	IL-17A	0.00871532	1.323305237	0.994793573	0.001939243	0.004330403	0.657855457
TG 55:5 FA20:4	IL-1β	0.389575012	1.021072565	0.70579239	0.061766176	0.038411391	0.119463962
TG 55:5 FA20:4	IL-6	0.766888109	0.658873411	0.254631552	0.020716664	0.003167089	5.15048E-07
TG 55:5 FA20:4	MCP1	0.154739827	0.64073181	0.810988623	0.003812706	0.00056704	3.21859E-07
TG 55:5 FA20:4	TNF-α	-1.83971E-11	0.793792233	1	0.11697176	0.024032214	4.35092E-05
TG 55:7 FA15:0	IL-1β	-0.870695065	4.491283064	0.847589616	0.070208678	0.033340355	0.043696239
TG 55:7 FA15:0	IL-6	1.621609055	3.302742563	0.62700714	0.01933201	0.003520679	5.82468E-06
TG 55:7 FA15:0	MCP1	0.609068204	3.093172408	0.845228045	0.003659951	0.000610292	1.40583E-06
TG 55:7 FA15:0	TNF-α	-0.136262406	4.119108908	0.973829468	0.108977608	0.027511788	0.000424855
TG 55:7 FA22:6	IL-17A	-1.694882586	1.395196872	0.234588537	0.002255027	0.003626134	0.539050618
TG 55:7 FA22:6	IL-1β	-1.493360789	1.38625947	0.290558611	0.064782692	0.036200657	0.084349772
TG 55:7 FA22:6	IL-6	0.662924441	1.125327494	0.560521009	0.01983087	0.004321981	8.53985E-05
TG 55:7 FA22:6	MCP1	-0.318549054	0.991239187	0.750320874	0.00341512	0.000697136	3.6576E-05
TG 55:7 FA22:6	TNF-α	-0.436008109	1.133520406	0.703403219	0.105871641	0.026032751	0.00035115
TG 56:1 FA16:0	IL-17A	-0.002118305	1.345640056	0.998754393	0.001943239	0.004241134	0.650118771
TG 56:1 FA16:0	IL-1β	-0.053476856	1.064581193	0.96026987	0.072553935	0.032986244	0.035685769
TG 56:1 FA16:0	IL-6	0.181150913	0.831324754	0.828977527	0.018973413	0.003698935	1.61641E-05
TG 56:1 FA16:0	MCP1	0.649276453	0.665547523	0.337087675	0.003700084	0.000548109	1.74799E-07
TG 56:1 FA16:0	TNF-α	0.234845633	0.95053741	0.806540005	0.110070285	0.026499583	0.000249908
TG 56:1 FA18:1	IL-17A	0.029542998	1.096485086	0.97868334	0.00191203	0.004208246	0.652844994
TG 56:1 FA18:1	IL-1β	0.435075362	0.841828495	0.609069929	0.073504686	0.031763105	0.027690655
TG 56:1 FA18:1	IL-6	0.433962917	0.63969645	0.502724934	0.018897058	0.003465973	6.49861E-06
TG 56:1 FA18:1	MCP1	0.542779504	0.542046456	0.32466534	0.003661373	0.000543588	1.82149E-07
TG 56:1 FA18:1	TNF-α	0.380277671	0.766618051	0.623476944	0.110572815	0.026025202	0.000191997
TG 56:10 FA18:2	IL-17A	0.0694789	1.486027082	0.963018515	0.001949581	0.004064131	0.634916711
TG 56:10 FA18:2	IL-1β	1.027971565	1.111445908	0.362400577	0.081545053	0.029883422	0.010527607
TG 56:10 FA18:2	IL-6	0.874744462	0.897337446	0.337443848	0.01936909	0.003464573	4.39898E-06
TG 56:10 FA18:2	MCP1	0.556631275	0.807695369	0.496018695	0.003663191	0.000577196	5.31517E-07
TG 56:10 FA18:2	TNF-α	0.813468159	1.105063272	0.467370029	0.109015551	0.026732827	0.000308066
TG 56:2 FA16:0	IL-17A	0.016345564	1.027550927	0.987413667	0.001926724	0.004159744	0.646575796
TG 56:2 FA16:0	IL-1β	0.580808457	0.820946594	0.484724582	0.080768796	0.032672259	0.019326697
TG 56:2 FA16:0	IL-6	0.471131931	0.584018793	0.426184841	0.01922691	0.003337667	2.72727E-06

TG 56:2 FA16:0	MCP1	0.263863156	0.564728438	0.643704924	0.003632266	0.000597362	1.11354E-06
TG 56:2 FA16:0	TNF-α	0.350981914	0.741562563	0.639424792	0.10864237	0.026553869	0.000296857
TG 56:2 FA18:0	IL-17A	0.279362456	0.358861367	0.442389693	0.001522862	0.002894683	0.602694026
TG 56:2 FA18:0	IL-1β	0.486931677	0.390298578	0.22183325	0.084609458	0.030950829	0.010403506
TG 56:2 FA18:0	IL-6	0.393099986	0.279761888	0.170255742	0.019191001	0.00318578	1.30399E-06
TG 56:2 FA18:0	MCP1	0.245140387	0.275274633	0.380267669	0.003643618	0.000580197	6.39232E-07
TG 56:2 FA18:0	TNF-α	0.522008289	0.351801153	0.148289007	0.10775038	0.025100844	0.000169869
TG 56:2 FA20:0	IL-17A	0.023431358	0.437969803	0.957688262	0.001915226	0.004074674	0.641731421
TG 56:2 FA20:0	IL-1β	0.235631826	0.33515257	0.487439913	0.077580045	0.030654358	0.016860434
TG 56:2 FA20:0	IL-6	0.198723393	0.259804139	0.450308412	0.019032356	0.003412303	4.56361E-06
TG 56:2 FA20:0	MCP1	0.228662723	0.224542204	0.316654009	0.003685454	0.00054586	1.74318E-07
TG 56:2 FA20:0	TNF-α	0.255011228	0.317019676	0.427493781	0.110897385	0.026088652	0.000190875
TG 56:2 FA20:1	IL-17A	0.275764446	0.354706839	0.442985232	0.001607969	0.002890346	0.582115589
TG 56:2 FA20:1	IL-1β	0.511467465	0.381937348	0.19058386	0.086677241	0.030596615	0.008167037
TG 56:2 FA20:1	IL-6	0.432823081	0.261264932	0.108015181	0.019131314	0.003005483	5.04354E-07
TG 56:2 FA20:1	MCP1	0.242992495	0.272335835	0.379358596	0.003640469	0.000579856	6.42318E-07
TG 56:2 FA20:1	TNF-α	0.50931223	0.347805989	0.153497662	0.107608749	0.02506883	0.000169949
TG 56:3 FA16:0	IL-17A	0.062938087	2.440931162	0.97961889	0.001935224	0.004320832	0.657811265
TG 56:3 FA16:0	IL-1β	1.862318738	1.851686052	0.323468536	0.06493113	0.037680292	0.096286049
TG 56:3 FA16:0	IL-6	2.026906468	1.096074439	0.075399947	0.020666295	0.00284998	8.45667E-08
TG 56:3 FA16:0	MCP1	0.728255592	1.199092821	0.548700321	0.003745406	0.000574028	5.37428E-07
TG 56:3 FA16:0	TNF-α	1.201429273	1.487266689	0.426259924	0.117973346	0.024356746	4.63752E-05
TG 56:3 FA18:0	IL-17A	0.073965372	0.783660386	0.925431343	0.001904457	0.003990408	0.636636556
TG 56:3 FA18:0	IL-1β	0.783500773	0.619007722	0.215349276	0.08929936	0.030987494	0.007239412
TG 56:3 FA18:0	IL-6	0.632654468	0.430920334	0.152471903	0.01925286	0.003097699	7.652E-07
TG 56:3 FA18:0	MCP1	0.324916549	0.432968442	0.458835933	0.003637628	0.000576077	5.80871E-07
TG 56:3 FA18:0	TNF-α	0.641641486	0.577952747	0.275733794	0.108780233	0.026031457	0.000233097
TG 56:3 FA18:1	IL-17A	0.060850561	0.086354547	0.486457002	0.001737164	0.003022725	0.569778353
TG 56:3 FA18:1	IL-1β	0.113546783	0.083487789	0.183950621	0.089318258	0.028730114	0.004090923
TG 56:3 FA18:1	IL-6	0.097960848	0.060004249	0.113014344	0.019374738	0.002965161	3.16594E-07
TG 56:3 FA18:1	MCP1	0.044775418	0.063258296	0.484520962	0.003645842	0.000578583	6.02452E-07
TG 56:3 FA18:1	TNF-α	0.117105137	0.079301078	0.150173014	0.109916874	0.024553264	0.000101622
TG 56:3 FA18:2	IL-17A	0.013034617	0.816004076	0.987361123	0.001927613	0.004100326	0.641674582
TG 56:3 FA18:2	IL-1β	0.384300222	0.633431753	0.548617194	0.077533891	0.031291534	0.019073461
TG 56:3 FA18:2	IL-6	0.378818297	0.48995767	0.445474145	0.019398047	0.003475662	4.51833E-06
TG 56:3 FA18:2	MCP1	0.329267975	0.430449908	0.450283675	0.003665134	0.000565176	3.62546E-07
TG 56:3 FA18:2	TNF-α	0.29469462	0.601810593	0.627922097	0.111418485	0.026748695	0.000241927
TG 56:3 FA20:0	IL-17A	0.014129563	0.864833325	0.987076706	0.001925118	0.004243739	0.653466565
TG 56:3 FA20:0	IL-1β	0.443062202	0.658661149	0.506481704	0.082031685	0.032672355	0.017875874
TG 56:3 FA20:0	IL-6	0.400307448	0.46610204	0.397471914	0.0196029	0.003280591	1.70396E-06
TG 56:3 FA20:0	MCP1	0.486510568	0.396960371	0.230213582	0.003816357	0.000516306	3.82218E-08
TG 56:3 FA20:0	TNF-α	0.496781585	0.557106388	0.379883721	0.124322703	0.025302811	3.22697E-05
TG 56:3 FA20:1	IL-17A	0.097244141	0.137003514	0.483313421	0.00176886	0.003032503	0.564050838
TG 56:3 FA20:1	IL-1β	0.172678202	0.133326283	0.205145471	0.089307902	0.029012579	0.004422569
TG 56:3 FA20:1	IL-6	0.149736061	0.095796113	0.128524664	0.019366737	0.002993439	3.78088E-07
TG 56:3 FA20:1	MCP1	0.071912248	0.100193902	0.478475451	0.003644443	0.00057949	6.23313E-07
TG 56:3 FA20:1	TNF-α	0.159386796	0.129286392	0.22721687	0.109678787	0.025312738	0.00015185
TG 56:3 FA20:2	IL-17A	0.804807447	1.178547935	0.500500239	0.001751159	0.003116932	0.578872191
TG 56:3 FA20:2	IL-1β	1.64703275	1.223249374	0.18935583	0.079202189	0.03719034	0.042467374
TG 56:3 FA20:2	IL-6	1.559237038	0.681059632	0.030097824	0.020527504	0.002645789	2.4153E-08

TG 56:3 FA20:2	MCP1	0.68840053	0.794843369	0.394075722	0.00379564	0.0005685	3.63397E-07
TG 56:3 FA20:2	TNF-α	1.65719784	0.915599489	0.081447229	0.120442593	0.022402898	1.11016E-05
TG 56:4 FA16:0	IL-17A	0.013196695	1.657627924	0.99370247	0.001937092	0.004308511	0.656343816
TG 56:4 FA16:0	IL-1β	1.150655826	1.284285373	0.377656483	0.08656017	0.033454363	0.014950158
TG 56:4 FA16:0	IL-6	0.198066756	1.098665116	0.85818708	0.018859083	0.004106574	7.86315E-05
TG 56:4 FA16:0	MCP1	-0.080059848	0.982090147	0.935588568	0.003642818	0.000678099	9.02056E-06
TG 56:4 FA16:0	TNF-α	-0.282331103	1.199275941	0.815538901	0.109511688	0.027560055	0.000429817
TG 56:4 FA18:0	IL-17A	0.026182734	0.470755458	0.956014461	0.00190901	0.004077373	0.643028267
TG 56:4 FA18:0	IL-1β	0.468314626	0.350596205	0.191671569	0.088468752	0.029853364	0.005907799
TG 56:4 FA18:0	IL-6	0.426423086	0.242303672	0.088624533	0.019621952	0.00296277	2.48068E-07
TG 56:4 FA18:0	MCP1	0.146132058	0.266017016	0.586842812	0.003607001	0.000602046	1.42884E-06
TG 56:4 FA18:0	TNF-α	0.386181264	0.337992934	0.262253355	0.107164633	0.025894613	0.000260624
TG 56:4 FA18:1	IL-17A	0.008341122	0.121366904	0.945663373	0.001928212	0.004027218	0.635555794
TG 56:4 FA18:1	IL-1β	0.118212369	0.091946216	0.208394726	0.088739238	0.029994364	0.005980878
TG 56:4 FA18:1	IL-6	0.112194385	0.064199823	0.090766846	0.019423493	0.003007402	3.89922E-07
TG 56:4 FA18:1	MCP1	0.033305327	0.069321005	0.63439202	0.003594765	0.000601042	1.47079E-06
TG 56:4 FA18:1	TNF-α	0.080411544	0.090568764	0.381684322	0.110009958	0.026582755	0.000260693
TG 56:4 FA18:2	IL-17A	0.005583724	0.284764466	0.984485741	0.001934361	0.004085078	0.639270928
TG 56:4 FA18:2	IL-1β	0.206362657	0.227289086	0.371151064	0.086138301	0.032054879	0.01163685
TG 56:4 FA18:2	IL-6	0.178864882	0.16416768	0.284596973	0.019415866	0.00332472	2.18326E-06
TG 56:4 FA18:2	MCP1	0.085027567	0.158900537	0.596525682	0.00362529	0.000595628	1.09507E-06
TG 56:4 FA18:2	TNF-α	0.141423904	0.202314308	0.489917326	0.109597412	0.025671894	0.00018137
TG 56:4 FA20:1	IL-17A	0.005097071	0.261461057	0.984575652	0.001936897	0.004086126	0.638918963
TG 56:4 FA20:1	IL-1β	0.184142586	0.208520523	0.384209182	0.084930205	0.032037197	0.012691627
TG 56:4 FA20:1	IL-6	0.186065728	0.149165915	0.22191033	0.019390423	0.003290995	1.8865E-06
TG 56:4 FA20:1	MCP1	0.078460821	0.14698791	0.597419498	0.003609831	0.000600235	1.34066E-06
TG 56:4 FA20:1	TNF-α	0.131897367	0.188912341	0.490433148	0.109310089	0.026114504	0.000228617
TG 56:4 FA20:2	IL-17A	0.021518031	0.309522751	0.945037029	0.001929075	0.004027701	0.635445548
TG 56:4 FA20:2	IL-1β	0.300426461	0.227733277	0.197082008	0.08929701	0.029133464	0.004572652
TG 56:4 FA20:2	IL-6	0.281461465	0.161720638	0.092035598	0.019253825	0.002970865	3.66633E-07
TG 56:4 FA20:2	MCP1	0.084729144	0.177558308	0.636684554	0.003592667	0.000603727	1.59989E-06
TG 56:4 FA20:2	TNF-α	0.188217757	0.234339061	0.428185501	0.107477073	0.026972802	0.000398296
TG 56:4 FA20:3	IL-17A	0.527353671	0.476694281	0.27770162	0.001489134	0.002892716	0.610603105
TG 56:4 FA20:3	IL-1β	0.736461477	0.465437624	0.124427727	0.064254237	0.030751678	0.045549817
TG 56:4 FA20:3	IL-6	0.738774406	0.306585296	0.022538271	0.020031096	0.002632603	2.17658E-08
TG 56:4 FA20:3	MCP1	0.331813284	0.355883556	0.358846061	0.003597663	0.000564997	5.84042E-07
TG 56:4 FA20:3	TNF-α	0.757440827	0.45971316	0.110221509	0.109360454	0.024673823	0.000122424
TG 56:4 FA20:4	IL-17A	1.44879E-14	2.292919533	1	1.62789E-16	0.004334725	1
TG 56:4 FA20:4	IL-1β	1.189773423	1.64874134	0.476111999	0.07935866	0.030642647	0.014676743
TG 56:4 FA20:4	IL-6	1.12309927	1.250113693	0.37612782	0.019731999	0.003336377	1.77259E-06
TG 56:4 FA20:4	MCP1	0.245826347	1.226170654	0.842455823	0.00366244	0.000605701	1.22404E-06
TG 56:4 FA20:4	TNF-α	4.98407E-11	1.640480819	1	0.109264991	0.027432201	0.000399999
TG 56:4 FA22:4	IL-17A	0.078405173	3.593108057	0.982751218	0.001927348	0.003432352	0.579071986
TG 56:4 FA22:4	IL-1β	3.051593885	3.724763809	0.419804606	0.054332423	0.039626304	0.181627269
TG 56:4 FA22:4	IL-6	2.775899138	2.55145718	0.286226811	0.01957615	0.003615208	1.00088E-05
TG 56:4 FA22:4	MCP1	1.533119268	2.515267675	0.547271849	0.003595022	0.000651556	7.60827E-06
TG 56:4 FA22:4	TNF-α	0.000408258	2.950414572	0.999890612	0.110294216	0.024902999	0.000141162
TG 56:5 FA16:0	IL-17A	0.005425351	0.303943314	0.985876801	0.001945164	0.004095915	0.63829501
TG 56:5 FA16:0	IL-1β	0.187353013	0.233678913	0.429000509	0.081559694	0.030958424	0.013200816
TG 56:5 FA16:0	IL-6	0.151415866	0.164766798	0.365441053	0.01930791	0.003134591	8.93183E-07

TG 56:5 FA16:0	MCP1	-0.009972342	0.178494427	0.955816439	0.003731687	0.000628518	1.66167E-06
TG 56:5 FA16:0	TNF-α	0.01554844	0.232479055	0.947120158	0.107382787	0.027711427	0.000537686
TG 56:5 FA18:0	IL-17A	0.008886973	0.296063447	0.976252205	0.001939116	0.004085344	0.638472189
TG 56:5 FA18:0	IL-1β	0.257474771	0.221113989	0.253421045	0.07955483	0.029995839	0.012655063
TG 56:5 FA18:0	IL-6	0.258810848	0.153190845	0.101498264	0.019887666	0.002984211	2.21399E-07
TG 56:5 FA18:0	MCP1	0.076203195	0.164358805	0.646252686	0.003603027	0.000592614	1.11546E-06
TG 56:5 FA18:0	TNF-α	0.20221057	0.217983484	0.360997106	0.109641311	0.026606279	0.000273632
TG 56:5 FA18:1	IL-17A	0.003008073	0.092923187	0.974390119	0.001944038	0.004078871	0.637091813
TG 56:5 FA18:1	IL-1β	0.080568006	0.065453028	0.227913219	0.083317976	0.028245325	0.006112906
TG 56:5 FA18:1	IL-6	0.075802823	0.049527225	0.136363711	0.019621538	0.003069112	4.67064E-07
TG 56:5 FA18:1	MCP1	0.016720293	0.053027575	0.754706848	0.003587545	0.000608209	1.85204E-06
TG 56:5 FA18:1	TNF-α	0.043197674	0.06962197	0.539640013	0.107568802	0.027032045	0.000404178
TG 56:5 FA18:2	IL-17A	0.002575956	0.269535524	0.992437992	0.00193843	0.004092148	0.639146687
TG 56:5 FA18:2	IL-1β	0.174735296	0.220118046	0.433531567	0.078895358	0.032854279	0.022735168
TG 56:5 FA18:2	IL-6	0.140337104	0.163036451	0.396191479	0.019316416	0.003494402	5.25033E-06
TG 56:5 FA18:2	MCP1	0.039063202	0.150506716	0.796987962	0.003640983	0.000597071	1.06031E-06
TG 56:5 FA18:2	TNF-α	0.094073387	0.204768633	0.649249388	0.109516837	0.027498909	0.000400553
TG 56:5 FA20:1	IL-17A	0.000338725	0.706377439	0.99962057	0.001940526	0.004093776	0.638918535
TG 56:5 FA20:1	IL-1β	0.203310478	0.590465549	0.733005912	0.07817488	0.033642078	0.027102674
TG 56:5 FA20:1	IL-6	0.110453367	0.455791873	0.810171284	0.019165465	0.003729123	1.57149E-05
TG 56:5 FA20:1	MCP1	0.060000377	0.393855193	0.879937784	0.003685922	0.000596429	8.43975E-07
TG 56:5 FA20:1	TNF-α	-0.021534576	0.540094564	0.968459452	0.108100357	0.027686866	0.000496258
TG 56:5 FA20:2	IL-17A	0.003904398	0.418606519	0.992619884	0.001940069	0.004097883	0.639332036
TG 56:5 FA20:2	IL-1β	0.279413935	0.3377208	0.414570563	0.079875204	0.032502168	0.019987408
TG 56:5 FA20:2	IL-6	0.193993686	0.256960302	0.456161786	0.019274121	0.003551178	6.96631E-06
TG 56:5 FA20:2	MCP1	0.032276855	0.23526229	0.891793149	0.003665302	0.000601784	1.08226E-06
TG 56:5 FA20:2	TNF-α	0.097199117	0.312439232	0.757877845	0.108829942	0.027054271	0.000358785
TG 56:5 FA20:3	IL-17A	0.02330049	0.213796558	0.913940584	0.001908031	0.004016065	0.638156778
TG 56:5 FA20:3	IL-1β	0.225414512	0.163040181	0.177003101	0.079157171	0.03010895	0.013373696
TG 56:5 FA20:3	IL-6	0.205296831	0.113694994	0.081012491	0.019449704	0.003015048	3.98282E-07
TG 56:5 FA20:3	MCP1	0.06140785	0.128900004	0.637241614	0.003566406	0.000632685	3.86116E-06
TG 56:5 FA20:3	TNF-α	0.194965704	0.151851636	0.208997136	0.107907403	0.0252311	0.000177574
TG 56:5 FA20:4	IL-17A	0.003567157	0.128624628	0.978058706	0.001934353	0.004093983	0.639998597
TG 56:5 FA20:4	IL-1β	0.09448926	0.09533183	0.329534116	0.079074388	0.029830516	0.012697657
TG 56:5 FA20:4	IL-6	0.109626886	0.067453872	0.114578479	0.019981305	0.00303097	2.69716E-07
TG 56:5 FA20:4	MCP1	0.029049211	0.071698185	0.688234976	0.003607134	0.0005963	1.21525E-06
TG 56:5 FA20:4	TNF-α	0.062935943	0.092274808	0.500440401	0.109009183	0.025978958	0.000222206
TG 56:5 FA22:4	IL-17A	0.002413294	0.253109835	0.99245575	0.001937055	0.00410116	0.64011707
TG 56:5 FA22:4	IL-1β	0.144824062	0.190561728	0.453194975	0.082486734	0.030355286	0.010821527
TG 56:5 FA22:4	IL-6	0.123535162	0.137997888	0.377808999	0.019372246	0.003156625	9.51246E-07
TG 56:5 FA22:4	MCP1	-0.019616705	0.153501905	0.899164382	0.00371726	0.0006499	3.05889E-06
TG 56:5 FA22:4	TNF-α	0.004670763	0.195796318	0.981126071	0.108564608	0.028062019	0.000547005
TG 56:5 FA22:5	IL-17A	0.025560463	1.830233188	0.988949821	0.001930311	0.004129324	0.643545678
TG 56:5 FA22:5	IL-1β	1.143168586	1.373264572	0.411735278	0.083471658	0.030459848	0.010235822
TG 56:5 FA22:5	IL-6	0.858865415	1.030682367	0.411262324	0.019377389	0.003282844	1.83101E-06
TG 56:5 FA22:5	MCP1	0.146092937	1.031577207	0.888326189	0.003670642	0.000608148	1.26165E-06
TG 56:5 FA22:5	TNF-α	0.313910782	1.312420932	0.812589046	0.107105853	0.026191623	0.000298564
TG 56:6 FA16:0	IL-17A	0.003824198	0.136639941	0.977857548	0.001946814	0.004083941	0.637029987
TG 56:6 FA16:0	IL-1β	0.106098398	0.096823222	0.281887739	0.079699741	0.028450003	0.008822922
TG 56:6 FA16:0	IL-6	0.091389272	0.071102455	0.208515553	0.01952706	0.003000125	3.39517E-07

TG 56:6 FA16:0	MCP1	0.023979301	0.08054119	0.767963521	0.003570345	0.000629006	3.45759E-06
TG 56:6 FA16:0	TNF-α	0.045939212	0.099996893	0.64925413	0.107523432	0.026436566	0.000317331
TG 56:6 FA18:0	IL-17A	0.004407126	0.632666629	0.994497309	0.001937384	0.004802372	0.690067292
TG 56:6 FA18:0	IL-1β	0.271160481	0.432820564	0.536668717	0.065062136	0.037887525	0.098300037
TG 56:6 FA18:0	IL-6	0.663285066	0.251333029	0.014106378	0.022556203	0.002893563	3.75679E-08
TG 56:6 FA18:0	MCP1	0.213276183	0.307266916	0.494014991	0.00370466	0.000653044	6.61008E-06
TG 56:6 FA18:0	TNF-α	0.01643227	0.337990948	0.961610463	0.114555831	0.023893458	6.34728E-05
TG 56:6 FA18:1	IL-17A	0.007673936	0.062815478	0.903609959	0.001916654	0.003048882	0.534504058
TG 56:6 FA18:1	IL-1β	0.067821938	0.0610723	0.275898836	0.060182422	0.032274507	0.072372547
TG 56:6 FA18:1	IL-6	0.069119144	0.039591846	0.091434885	0.020214455	0.002719242	3.42462E-08
TG 56:6 FA18:1	MCP1	0.022404015	0.04447509	0.618247448	0.003688084	0.000564759	3.75907E-07
TG 56:6 FA18:1	TNF-α	0.061995912	0.057016368	0.285843635	0.109387088	0.024476937	0.000110618
TG 56:6 FA18:2	IL-17A	0.001939362	0.166865496	0.990803897	0.001940118	0.00409644	0.639206055
TG 56:6 FA18:2	IL-1β	0.104735092	0.130624263	0.4289719	0.077592984	0.031525709	0.019816345
TG 56:6 FA18:2	IL-6	0.113921875	0.092778987	0.229037605	0.019824373	0.003215456	8.7909E-07
TG 56:6 FA18:2	MCP1	0.014968277	0.094245401	0.874873204	0.003661153	0.000604554	1.19251E-06
TG 56:6 FA18:2	TNF-α	0.05008368	0.124597121	0.690559322	0.109115311	0.027056073	0.000348776
TG 56:6 FA18:3	IL-17A	-0.004598151	2.310713777	0.998426899	0.00194089	0.004776372	0.687686725
TG 56:6 FA18:3	IL-1β	0.672558388	1.748990962	0.703589884	0.079937391	0.037496179	0.042266672
TG 56:6 FA18:3	IL-6	1.782175392	1.202378328	0.149863274	0.020744396	0.003748939	7.29393E-06
TG 56:6 FA18:3	MCP1	0.385637027	1.147313514	0.739377294	0.003666812	0.000653334	5.908E-06
TG 56:6 FA18:3	TNF-α	-0.12669392	1.317040985	0.924075468	0.11590191	0.025166728	8.79532E-05
TG 56:6 FA20:2	IL-17A	0.000951478	0.895380588	0.999159162	0.001940501	0.004102116	0.639602214
TG 56:6 FA20:2	IL-1β	0.201271868	0.726878863	0.783756818	0.078118279	0.032738842	0.023539628
TG 56:6 FA20:2	IL-6	0.165326053	0.567783808	0.772919079	0.019256516	0.003672283	1.17036E-05
TG 56:6 FA20:2	MCP1	0.053383839	0.502644379	0.91612594	0.00368066	0.000601722	1.00615E-06
TG 56:6 FA20:2	TNF-α	-1.19516E-11	0.677040928	1	0.109289539	0.027436704	0.000399735
TG 56:6 FA20:3	IL-17A	0.009515557	0.267257903	0.971833657	0.001948462	0.004080855	0.636492032
TG 56:6 FA20:3	IL-1β	0.22875408	0.199023113	0.259478646	0.07944522	0.029876183	0.012446511
TG 56:6 FA20:3	IL-6	0.211723578	0.14470941	0.153840595	0.019602321	0.003119394	6.32092E-07
TG 56:6 FA20:3	MCP1	0.050489478	0.150939066	0.740327908	0.00359302	0.000602223	1.53221E-06
TG 56:6 FA20:3	TNF-α	0.173784366	0.19924889	0.390028024	0.1055982	0.026911212	0.000470408
TG 56:6 FA20:4	IL-17A	5.00049E-16	0.064308231	1	5.41562E-17	0.004291587	1
TG 56:6 FA20:4	IL-1β	0.015903307	0.048915101	0.747346373	0.076054743	0.032091893	0.024421389
TG 56:6 FA20:4	IL-6	0.021838382	0.036731753	0.556610827	0.019705204	0.003460556	3.28638E-06
TG 56:6 FA20:4	MCP1	-0.002380704	0.034782165	0.945884705	0.003726141	0.000606517	9.34189E-07
TG 56:6 FA20:4	TNF-α	-0.002048387	0.04639048	0.965073181	0.108933207	0.027384007	0.000405646
TG 56:6 FA20:5	IL-17A	2.03285E-15	1.257411996	1	1.43439E-17	0.004289291	1
TG 56:6 FA20:5	IL-1β	-0.066354631	0.992447779	0.947137104	0.071754636	0.033282547	0.039233291
TG 56:6 FA20:5	IL-6	0.5463888	0.693217282	0.43676814	0.01949468	0.003338337	2.18455E-06
TG 56:6 FA20:5	MCP1	0.215629712	0.662957399	0.747246234	0.003655166	0.00059092	8.30994E-07
TG 56:6 FA20:5	TNF-α	0.0163102	0.922328184	0.98600825	0.108884859	0.027829802	0.000485339
TG 56:6 FA22:4	IL-1β	0.086375512	0.29920526	0.774809256	0.078872247	0.032887773	0.022898965
TG 56:6 FA22:4	IL-6	0.062258075	0.233724364	0.791773594	0.019198994	0.003689106	1.30851E-05
TG 56:6 FA22:4	MCP1	-0.06904718	0.218285684	0.753953398	0.003701926	0.000637712	2.40742E-06
TG 56:6 FA22:4	TNF-α	-0.036126866	0.28489944	0.899940312	0.108671866	0.028175561	0.000564867
TG 56:6 FA22:5	IL-17A	0.006778191	0.095561379	0.943924026	0.001908833	0.00406515	0.642064404
TG 56:6 FA22:5	IL-1β	0.087580556	0.068488776	0.210786408	0.080114632	0.028642738	0.008917604
TG 56:6 FA22:5	IL-6	0.075492926	0.048809193	0.132422169	0.019682983	0.002931222	1.92722E-07
TG 56:6 FA22:5	MCP1	0.02079288	0.054953271	0.707817293	0.003586203	0.000610833	2.00064E-06

TG 56:6 FA22:5	TNF-α	0.062369005	0.070842527	0.385647036	0.107491503	0.02665661	0.000349238
TG 56:6 FA22:6	IL-17A	0.018792286	1.101183568	0.986497328	0.001929406	0.004112413	0.642342532
TG 56:6 FA22:6	IL-1β	0.634836095	0.830087581	0.4503758	0.080605355	0.030476277	0.012878725
TG 56:6 FA22:6	IL-6	0.604036671	0.64862825	0.359157091	0.019759133	0.003419682	2.59664E-06
TG 56:6 FA22:6	MCP1	0.241779892	0.617050763	0.697952423	0.003670635	0.000602133	1.06631E-06
TG 56:6 FA22:6	TNF-α	0.193116783	0.804005977	0.811814227	0.107812751	0.02655908	0.00032429
TG 56:7 FA16:0	IL-17A	0.00283364	0.14921029	0.984974101	0.001946457	0.004092349	0.637781034
TG 56:7 FA16:0	IL-1β	0.091674456	0.108346019	0.404180912	0.07548836	0.029213786	0.014879622
TG 56:7 FA16:0	IL-6	0.095788967	0.082767344	0.256270312	0.020218666	0.003204688	5.89623E-07
TG 56:7 FA16:0	MCP1	0.023102374	0.083988035	0.785150038	0.003643569	0.000601903	1.20099E-06
TG 56:7 FA16:0	TNF-α	0.029257634	0.108826668	0.789888356	0.108440013	0.02640134	0.000284045
TG 56:7 FA16:1	IL-17A	0.042060725	1.686120605	0.980263816	0.001954446	0.004091852	0.636365936
TG 56:7 FA16:1	IL-1β	1.198949853	1.231329512	0.337990048	0.070044126	0.029376942	0.023635117
TG 56:7 FA16:1	IL-6	1.075650476	0.892773464	0.237683412	0.019333447	0.00305862	5.70517E-07
TG 56:7 FA16:1	MCP1	-0.047806897	0.985381293	0.961626463	0.003724568	0.000624842	1.55576E-06
TG 56:7 FA16:1	TNF-α	0.348169226	1.259746567	0.78415113	0.105605398	0.027041528	0.00049502
TG 56:7 FA18:0	IL-17A	0.063281097	2.07128709	0.975829507	0.001946473	0.004088541	0.637466147
TG 56:7 FA18:0	IL-1β	1.23933019	1.546498598	0.429214667	0.070583381	0.030010867	0.025435101
TG 56:7 FA18:0	IL-6	1.215884137	1.215742079	0.325253	0.019541265	0.003387836	2.67055E-06
TG 56:7 FA18:0	MCP1	0.841819695	1.13229774	0.46298551	0.003702559	0.000584014	5.4145E-07
TG 56:7 FA18:0	TNF-α	0.855180206	1.505607994	0.574264264	0.112292401	0.026287947	0.000180128
TG 56:7 FA18:1	IL-17A	4.60788E-16	0.147462562	1	1.43053E-17	0.00429944	1
TG 56:7 FA18:1	IL-1β	-0.021276142	0.118147799	0.858300114	0.071233906	0.03386544	0.043918745
TG 56:7 FA18:1	IL-6	0.051675605	0.083115598	0.538814768	0.019827805	0.003421098	2.47089E-06
TG 56:7 FA18:1	MCP1	-0.014105582	0.082764787	0.865816389	0.003707358	0.000630537	1.95256E-06
TG 56:7 FA18:1	TNF-α	-0.019023283	0.108130023	0.861531363	0.107507454	0.027886443	0.000567596
TG 56:7 FA18:2	IL-17A	0.002610133	0.151693486	0.986385703	0.001938533	0.004099689	0.639743266
TG 56:7 FA18:2	IL-1β	0.08305942	0.117909416	0.486595327	0.073511809	0.031328058	0.025747602
TG 56:7 FA18:2	IL-6	0.098687726	0.082561333	0.24132339	0.019901495	0.003150022	5.75409E-07
TG 56:7 FA18:2	MCP1	0.014865199	0.084787432	0.862003726	0.003655405	0.000598757	1.04004E-06
TG 56:7 FA18:2	TNF-α	0.040876441	0.109946732	0.712666817	0.108560078	0.026283491	0.000266559
TG 56:7 FA18:3	IL-17A	0.023225481	1.362498662	0.986529865	0.00196211	0.004594287	0.672839918
TG 56:7 FA18:3	IL-1β	0.661150659	0.996020626	0.51266703	0.05584405	0.038730682	0.161281022
TG 56:7 FA18:3	IL-6	1.45564126	0.561075753	0.015366706	0.021656535	0.002866799	5.09422E-08
TG 56:7 FA18:3	MCP1	0.419186078	0.67280512	0.538683949	0.00373342	0.000626642	2.74503E-06
TG 56:7 FA18:3	TNF-α	0.193300211	0.764790108	0.8024495	0.118141546	0.023841222	3.7807E-05
TG 56:7 FA20:3	IL-17A	0.00599567	0.556157109	0.99146992	0.001943265	0.004118898	0.640488486
TG 56:7 FA20:3	IL-1β	0.234239351	0.439389103	0.597890231	0.075535612	0.031991443	0.024912119
TG 56:7 FA20:3	IL-6	0.246677008	0.319206466	0.445697599	0.019547519	0.003337399	2.08009E-06
TG 56:7 FA20:3	MCP1	-0.009508399	0.312123255	0.975899087	0.003726459	0.000604011	8.68904E-07
TG 56:7 FA20:3	TNF-α	0.139623738	0.413282806	0.737835992	0.108894532	0.027073701	0.00035928
TG 56:7 FA20:4	IL-17A						
TG 56:7 FA20:4	IL-1β	-0.025994525	0.073293281	0.725322051	0.067992834	0.035532772	0.065265656
TG 56:7 FA20:4	IL-6	-0.002447654	0.056521705	0.965745612	0.018885438	0.003934885	4.10179E-05
TG 56:7 FA20:4	MCP1	-0.022715173	0.048131211	0.640382466	0.003614704	0.000620191	2.2547E-06
TG 56:7 FA20:4	TNF-α	-0.026975183	0.062492522	0.66907892	0.105989075	0.027258924	0.000518658
TG 56:7 FA20:5	IL-1β	-0.089793361	0.262054999	0.734250456	0.075662876	0.033860366	0.033046185
TG 56:7 FA20:5	IL-6	0.123983139	0.179663634	0.495445212	0.019411804	0.003333583	2.28826E-06
TG 56:7 FA20:5	MCP1	0.032124581	0.174122577	0.854867414	0.003672226	0.000597982	9.40659E-07
TG 56:7 FA20:5	TNF-α	-0.007418225	0.235529608	0.975082648	0.109125036	0.027381703	0.000397553

TG 56:7 FA22:4	IL-17A	0.047936586	2.479121718	0.984715159	0.00195511	0.004322838	0.654683934
TG 56:7 FA22:4	IL-1β	1.539392975	1.871039464	0.417859099	0.065591815	0.037504997	0.09167334
TG 56:7 FA22:4	IL-6	1.929551915	1.101444172	0.09115439	0.020911063	0.002821133	5.66258E-08
TG 56:7 FA22:4	MCP1	0.242874836	1.218636747	0.843521057	0.003775814	0.000574664	4.77591E-07
TG 56:7 FA22:4	TNF-α	0.318227972	1.508277677	0.834481254	0.117579453	0.024331619	4.77909E-05
TG 56:7 FA22:5	IL-17A	0.003801196	0.130043457	0.976874589	0.001946548	0.00408201	0.636917053
TG 56:7 FA22:5	IL-1β	0.110200291	0.094943025	0.254917512	0.078606446	0.029298782	0.011757301
TG 56:7 FA22:5	IL-6	0.102888689	0.070333385	0.153901356	0.019809973	0.003116735	5.17752E-07
TG 56:7 FA22:5	MCP1	0.032400135	0.072431304	0.657855586	0.003609922	0.000594082	1.12617E-06
TG 56:7 FA22:5	TNF-α	0.056918478	0.09864968	0.568260585	0.108198204	0.027390372	0.00043774
TG 56:7 FA22:6	IL-17A	1.78431E-16	0.060205409	1	7.15326E-17	0.004289356	1
TG 56:7 FA22:6	IL-1β	-0.019956725	0.049507497	0.689729441	0.07282593	0.034675966	0.044225041
TG 56:7 FA22:6	IL-6	0.020763041	0.034314928	0.54968039	0.019755643	0.003451378	3.02264E-06
TG 56:7 FA22:6	MCP1	-0.004187085	0.03351481	0.901410496	0.00370167	0.000623919	1.682E-06
TG 56:7 FA22:6	TNF-α	-0.008732656	0.043879865	0.843595386	0.106264717	0.027652769	0.000587041
TG 56:8 FA16:0	IL-17A	3.49581E-16	0.325664663	1	1.37488E-17	0.00429477	1
TG 56:8 FA16:0	IL-1β	-0.173847598	0.275458779	0.532741328	0.076343779	0.035713045	0.040807148
TG 56:8 FA16:0	IL-6	0.073638196	0.199369683	0.714457455	0.019555576	0.003711772	1.09125E-05
TG 56:8 FA16:0	MCP1	-0.058277534	0.187297821	0.757839188	0.003652331	0.000645412	3.62956E-06
TG 56:8 FA16:0	TNF-α	-0.08645544	0.240247696	0.721473161	0.104614872	0.028024976	0.000791058
TG 56:8 FA16:1	IL-17A	0.006491476	1.328399395	0.996133352	0.001939885	0.004105677	0.639997404
TG 56:8 FA16:1	IL-1β	-0.029394538	1.085535368	0.978576549	0.072063294	0.032983826	0.036849591
TG 56:8 FA16:1	IL-6	0.731445533	0.744462508	0.333704058	0.019620059	0.003248268	1.24629E-06
TG 56:8 FA16:1	MCP1	-0.125777141	0.783070006	0.873468976	0.00369922	0.0006324	2.12497E-06
TG 56:8 FA16:1	TNF-α	-0.050308191	1.002421116	0.960306285	0.109093556	0.027404571	0.000402467
TG 56:8 FA18:2	IL-17A	7.91174E-16	0.25658143	1	7.30948E-17	0.004311498	1
TG 56:8 FA18:2	IL-1β	-0.100606245	0.217240912	0.646628278	0.074684166	0.035887695	0.046064008
TG 56:8 FA18:2	IL-6	0.049010006	0.157893461	0.758403652	0.019476547	0.003745588	1.32478E-05
TG 56:8 FA18:2	MCP1	-0.062784236	0.143686581	0.665274835	0.00365955	0.000630891	2.43737E-06
TG 56:8 FA18:2	TNF-α	-0.067404595	0.188666584	0.723392037	0.105054695	0.028042368	0.000762961
TG 56:8 FA18:3	IL-17A	-0.001760644	0.998807383	0.998605603	0.001940837	0.004338077	0.657913422
TG 56:8 FA18:3	IL-1β	-0.582694646	0.887300549	0.516548532	0.068746644	0.038622231	0.085561668
TG 56:8 FA18:3	IL-6	0.065768764	0.660855926	0.921409738	0.018860503	0.004127589	8.38092E-05
TG 56:8 FA18:3	MCP1	-0.19757426	0.571617054	0.7321074	0.003587264	0.000659511	7.48685E-06
TG 56:8 FA18:3	TNF-α	-0.290545647	0.707865593	0.68449044	0.106581578	0.027182358	0.000495599
TG 56:8 FA20:4	IL-17A	-0.015736704	0.176981153	0.929738506	0.001964216	0.004070879	0.632949953
TG 56:8 FA20:4	IL-1β	-0.143139193	0.165818702	0.394861617	0.068589424	0.037496922	0.077325548
TG 56:8 FA20:4	IL-6	-0.023957062	0.122534382	0.846309601	0.018534886	0.003978982	6.10564E-05
TG 56:8 FA20:4	MCP1	-0.077723412	0.106947239	0.473015011	0.003568938	0.000642785	4.90021E-06
TG 56:8 FA20:4	TNF-α	-0.145760902	0.125419597	0.254322522	0.100571645	0.025517802	0.000448661
TG 56:8 FA20:5	IL-1β	-0.226781085	0.327721062	0.494262381	0.079152872	0.035123992	0.031691301
TG 56:8 FA20:5	IL-6	0.087012307	0.23178726	0.710006255	0.019465483	0.003567311	6.41736E-06
TG 56:8 FA20:5	MCP1	-0.020385886	0.220963658	0.927105605	0.003679076	0.00062944	2.15195E-06
TG 56:8 FA20:5	TNF-α	-0.044703264	0.291559038	0.879168751	0.106828308	0.028115239	0.00066017
TG 56:8 FA22:5	IL-17A	0.006147576	0.839892213	0.994210071	0.001944481	0.004321008	0.656051837
TG 56:8 FA22:5	IL-1β	0.341047904	0.638246611	0.597171632	0.072519632	0.032341865	0.032757058
TG 56:8 FA22:5	IL-6	0.642444183	0.458736832	0.171983754	0.020156564	0.003414855	2.08131E-06
TG 56:8 FA22:5	MCP1	0.093694006	0.455861933	0.838592826	0.003648536	0.000623095	2.36938E-06
TG 56:8 FA22:5	TNF-α	-0.036469557	0.580167146	0.950308917	0.110308061	0.026385852	0.000244511
TG 56:8 FA22:6	IL-17A	-1.73852E-15	0.079275994	1	3.55094E-16	0.004298138	1

TG 56:8 FA22:6	IL-1β	-0.04347935	0.067079347	0.521800448	0.079027496	0.035754313	0.034854761
TG 56:8 FA22:6	IL-6	0.013619572	0.049504593	0.785112075	0.019428605	0.003789108	1.62528E-05
TG 56:8 FA22:6	MCP1	-0.020590454	0.044308647	0.645497884	0.00364512	0.000627714	2.39422E-06
TG 56:8 FA22:6	TNF-α	-0.021685503	0.057907012	0.71067517	0.104762932	0.02777068	0.000710797
TG 56:9 FA18:3	IL-17A	-0.946625526	1.446399951	0.518557033	0.001993573	0.004091788	0.630189917
TG 56:9 FA18:3	IL-1β	-1.561291579	1.301306463	0.241040949	0.053306842	0.042453074	0.220405053
TG 56:9 FA18:3	IL-6	0.288803927	1.016019121	0.778470012	0.019799663	0.004355323	0.000111399
TG 56:9 FA18:3	MCP1	-0.228053712	0.854202501	0.791591931	0.003685246	0.000667473	8.5402E-06
TG 56:9 FA18:3	TNF-α	-0.653178183	0.878443243	0.463807551	0.112675077	0.022974322	4.325E-05
TG 56:9 FA20:4	IL-17A	-0.321646069	0.560151769	0.570104914	0.001976395	0.004017155	0.626308263
TG 56:9 FA20:4	IL-1β	-0.737719547	0.527298743	0.17205128	0.07239949	0.037176678	0.060894585
TG 56:9 FA20:4	IL-6	-0.088207082	0.385032458	0.820353602	0.018490248	0.003898191	4.80546E-05
TG 56:9 FA20:4	MCP1	-0.202685061	0.327468205	0.540624017	0.003566363	0.000613645	2.36219E-06
TG 56:9 FA20:4	TNF-α	-0.467856572	0.414580142	0.268049988	0.102694119	0.02629893	0.000495597
TG 56:9 FA20:5	IL-17A	-0.031151369	0.800007295	0.969205968	0.00194117	0.004327956	0.657112145
TG 56:9 FA20:5	IL-1β	-0.581221291	0.689129353	0.405900282	0.071205622	0.037362911	0.066634523
TG 56:9 FA20:5	IL-6	0.015059565	0.540533789	0.977964224	0.018793682	0.004205193	0.000110564
TG 56:9 FA20:5	MCP1	-0.161639234	0.470609977	0.733724479	0.003562301	0.000676319	1.20711E-05
TG 56:9 FA20:5	TNF-α	-0.229178431	0.57362454	0.692432358	0.106102385	0.02743705	0.000573195
TG 56:9 FA22:6	IL-17A	-1.92954E-16	0.473986347	1	7.12319E-17	0.004304745	1
TG 56:9 FA22:6	IL-1β	-0.257493605	0.392745183	0.517062118	0.079994354	0.03506658	0.029803749
TG 56:9 FA22:6	IL-6	0.064303223	0.304373176	0.834109548	0.019364275	0.003902481	2.59319E-05
TG 56:9 FA22:6	MCP1	-0.121891603	0.263983077	0.647598516	0.00364604	0.000626459	2.30776E-06
TG 56:9 FA22:6	TNF-α	-0.128306491	0.347253774	0.714358565	0.105058318	0.027896214	0.000723211
TG 57:2 FA18:1	IL-17A	1.35791E-15	0.719751333	1	7.18165E-17	0.004292675	1
TG 57:2 FA18:1	IL-1β	-0.300500454	0.606287622	0.623759822	0.0750894	0.035548825	0.043095461
TG 57:2 FA18:1	IL-6	0.12276668	0.456830474	0.789972539	0.019453334	0.003846392	1.98021E-05
TG 57:2 FA18:1	MCP1	-0.050473006	0.407944773	0.902358187	0.003681456	0.000635743	2.50548E-06
TG 57:2 FA18:1	TNF-α	-0.173060687	0.536484696	0.749248774	0.103904763	0.028302163	0.000934206
TG 57:3 FA18:2	IL-1β	-0.620528853	0.969878098	0.527158301	0.075695759	0.035468859	0.041120473
TG 57:3 FA18:2	IL-6	0.11646414	0.750067262	0.877647234	0.019172508	0.003938966	3.38704E-05
TG 57:3 FA18:2	MCP1	-0.128074335	0.653352848	0.845911395	0.00366658	0.000635056	2.62911E-06
TG 57:3 FA18:2	TNF-α	-0.337816443	0.860276386	0.697329532	0.105188693	0.028306347	0.000827874
TG 58:10 FA18:2	IL-17A	-0.06787936	1.461593032	0.963287592	0.001958396	0.004489595	0.666031104
TG 58:10 FA18:2	IL-1β	-0.809635009	1.2704217	0.529108924	0.074403165	0.040327259	0.075641994
TG 58:10 FA18:2	IL-6	0.332227399	0.924473926	0.722015063	0.01997937	0.00415668	4.70636E-05
TG 58:10 FA18:2	MCP1	-0.116056837	0.755543003	0.879021206	0.003693998	0.000626375	2.41261E-06
TG 58:10 FA18:2	TNF-α	-0.403093976	0.935841478	0.669963183	0.111319037	0.026593609	0.000254758
TG 58:10 FA20:4	IL-17A	-0.69687359	0.454885905	0.137163139	0.001975167	0.003961441	0.622100327
TG 58:10 FA20:4	IL-1β	-0.750765776	0.438018888	0.097987927	0.053701194	0.043850919	0.231291991
TG 58:10 FA20:4	IL-6	-0.056571889	0.308710978	0.855969523	0.018869674	0.003949052	5.52705E-05
TG 58:10 FA20:4	MCP1	-0.153925524	0.249555651	0.542534247	0.00364296	0.000587742	1.25697E-06
TG 58:10 FA20:4	TNF-α	-0.376612094	0.259014631	0.157467686	0.108825138	0.020868617	1.71089E-05
TG 58:10 FA20:5	IL-17A	-3.169772579	1.85913847	0.101113198	0.001923928	0.003804213	0.617655545
TG 58:10 FA20:5	IL-1β	-3.354820332	1.746174116	0.066655784	0.045973119	0.041727684	0.281500845
TG 58:10 FA20:5	IL-6	-0.08012446	1.442955218	0.956177484	0.019195976	0.004588748	0.000331272
TG 58:10 FA20:5	MCP1	-0.678715529	1.199289557	0.576694226	0.003521655	0.000699824	3.83118E-05
TG 58:10 FA20:5	TNF-α	-1.381961097	1.232198542	0.273154194	0.10718816	0.023607174	0.00013361
TG 58:10 FA22:5	IL-17A	-0.007849172	1.177689705	0.994727865	0.001944885	0.004343701	0.657661842
TG 58:10 FA22:5	IL-1β	-0.727120259	1.108198586	0.516914252	0.087917377	0.040258878	0.037212246

TG 58:10 FA22:5	IL-6	0.07760452	0.791590744	0.922578054	0.019044425	0.004224515	9.92945E-05
TG 58:10 FA22:5	MCP1	0.985491459	0.611173184	0.11769342	0.003859566	0.000598898	4.74196E-07
TG 58:10 FA22:5	TNF-α	0.151336804	0.817911102	0.854494709	0.110600836	0.026668072	0.0002678
TG 58:10 FA22:6	IL-17A	-0.00653953	0.432690837	0.988041591	0.001943394	0.004109515	0.639706508
TG 58:10 FA22:6	IL-1β	-0.275494912	0.378167409	0.471956457	0.073818269	0.035310019	0.045140331
TG 58:10 FA22:6	IL-6	0.050931028	0.297447038	0.865194824	0.019343062	0.003988186	3.55646E-05
TG 58:10 FA22:6	MCP1	-0.097659333	0.258164271	0.707883549	0.003630355	0.000640684	3.55421E-06
TG 58:10 FA22:6	TNF-α	-0.162390283	0.325250337	0.621225552	0.105430191	0.027324194	0.000562505
TG 58:2 FA18:1	IL-17A	-0.013277673	0.65584014	0.983981755	0.001958263	0.00412556	0.638462377
TG 58:2 FA18:1	IL-1β	-0.14931981	0.564716423	0.793267858	0.080240034	0.034923383	0.02873485
TG 58:2 FA18:1	IL-6	0.035242084	0.423072208	0.934166041	0.019043799	0.003757096	1.91855E-05
TG 58:2 FA18:1	MCP1	0.348678849	0.331365725	0.301088029	0.003777209	0.000544662	1.05804E-07
TG 58:2 FA18:1	TNF-α	0.068361404	0.491894918	0.890398163	0.110208391	0.027369948	0.000354889
TG 58:3 FA18:1	IL-17A	0.013898669	0.932180492	0.988202821	0.001928432	0.004114982	0.642716807
TG 58:3 FA18:1	IL-1β	-0.049264302	0.764901487	0.949074151	0.073582844	0.033195134	0.034370562
TG 58:3 FA18:1	IL-6	0.13345965	0.593954113	0.823738531	0.018926884	0.003701466	1.6915E-05
TG 58:3 FA18:1	MCP1	0.479734213	0.474906796	0.320494807	0.003752669	0.000547786	1.33074E-07
TG 58:3 FA18:1	TNF-α	0.234168644	0.66192831	0.725987448	0.109679047	0.025846136	0.000194762
TG 58:5 FA18:1	IL-17A	0.05104314	1.563269568	0.974176175	0.001943734	0.004222526	0.648716213
TG 58:5 FA18:1	IL-1β	1.532272468	1.177765224	0.203501193	0.092809971	0.032158763	0.007293392
TG 58:5 FA18:1	IL-6	1.26924461	0.758367029	0.10495288	0.020283378	0.002938142	1.38273E-07
TG 58:5 FA18:1	MCP1	0.384694673	0.818886468	0.642027204	0.00377059	0.000586281	4.91282E-07
TG 58:5 FA18:1	TNF-α	1.12311311	1.052887548	0.294908439	0.116979649	0.026322968	0.000118512
TG 58:6 FA16:0	IL-17A	0.021348584	1.728772959	0.990228948	0.001945054	0.004092373	0.638024337
TG 58:6 FA16:0	IL-1β	1.184549217	1.375942687	0.396121671	0.082620669	0.032021272	0.015014247
TG 58:6 FA16:0	IL-6	0.129150635	1.129303658	0.909711967	0.018937038	0.003773991	2.21563E-05
TG 58:6 FA16:0	MCP1	-0.044207161	1.013379849	0.9654936	0.003725501	0.000626822	1.6331E-06
TG 58:6 FA16:0	TNF-α	-0.083938284	1.347142129	0.950730519	0.108905135	0.028207682	0.000558927
TG 58:6 FA18:0	IL-17A	1.410200018	1.833596709	0.449045708	0.001709452	0.003449844	0.624563847
TG 58:6 FA18:0	IL-1β	2.500747953	1.642096253	0.140334069	0.071540881	0.035628844	0.055570603
TG 58:6 FA18:0	IL-6	2.388213291	0.919618752	0.015532595	0.021017674	0.002624254	2.30202E-08
TG 58:6 FA18:0	MCP1	1.164925058	1.195750509	0.339279846	0.003672951	0.000629915	4.42426E-06
TG 58:6 FA18:0	TNF-α	1.586130384	1.301995225	0.234508846	0.117324833	0.022813825	2.57742E-05
TG 58:6 FA18:1	IL-17A	0.01716846	0.992964175	0.986342427	0.001945318	0.004801426	0.688810316
TG 58:6 FA18:1	IL-1β	0.703144723	0.689140981	0.317349277	0.065256949	0.03842839	0.101895292
TG 58:6 FA18:1	IL-6	0.753994284	0.347848235	0.039914976	0.021582862	0.002551108	8.34971E-09
TG 58:6 FA18:1	MCP1	0.275218251	0.472338192	0.565333088	0.003684765	0.000639492	5.26908E-06
TG 58:6 FA18:1	TNF-α	0.027177948	0.523969668	0.959045059	0.114749595	0.023595862	5.31236E-05
TG 58:6 FA20:4	IL-17A	7.48192E-15	4.244283866	1	8.4132E-17	0.004820878	1
TG 58:6 FA20:4	IL-1β	-1.578064979	3.43505543	0.649764933	0.044294484	0.044994589	0.333972013
TG 58:6 FA20:4	IL-6	3.682565229	1.999387973	0.076932876	0.023169631	0.003441221	3.82028E-07
TG 58:6 FA20:4	MCP1	0.538028418	2.002844248	0.790332821	0.0038454	0.000628372	1.80975E-06
TG 58:6 FA20:4	TNF-α	-0.368220002	2.217652365	0.869409555	0.116224543	0.023287317	3.44219E-05
TG 58:6 FA22:4	IL-17A	0.022625309	1.176725555	0.984787055	0.001945942	0.004087709	0.637489466
TG 58:6 FA22:4	IL-1β	0.775099126	0.868445934	0.37922168	0.080341978	0.029658459	0.011044934
TG 58:6 FA22:4	IL-6	0.710895131	0.628359486	0.26687047	0.019635847	0.003081526	4.95159E-07
TG 58:6 FA22:4	MCP1	0.158200631	0.663804573	0.813250128	0.003621537	0.000602532	1.35383E-06
TG 58:6 FA22:4	TNF-α	0.403211905	0.865182382	0.644551162	0.107100308	0.026584544	0.000352894
TG 58:6 FA22:5	IL-17A	1.344227961	1.104727816	0.233168386	0.001398184	0.002971129	0.641337084
TG 58:6 FA22:5	IL-1β	1.856919371	1.112590458	0.105521152	0.086652272	0.029417279	0.006176945

TG 58:6 FA22:5	IL-6	1.513905204	0.773272312	0.059612935	0.019915988	0.002935969	1.59815E-07
TG 58:6 FA22:5	MCP1	0.913653231	0.806949336	0.266506174	0.003591294	0.000567084	5.51942E-07
TG 58:6 FA22:5	TNF-α	1.783485681	1.065106708	0.10443461	0.108343567	0.025338216	0.000178003
TG 58:7 FA16:0	IL-17A	0.012323576	1.427385426	0.993168572	0.001942239	0.00409037	0.638345165
TG 58:7 FA16:0	IL-1β	0.65376566	1.175428128	0.582203634	0.07776749	0.033114512	0.025636044
TG 58:7 FA16:0	IL-6	0.121290022	0.929124563	0.897008864	0.018933748	0.003758791	2.09742E-05
TG 58:7 FA16:0	MCP1	-0.000622892	0.816170721	0.999396116	0.003708642	0.000611135	1.15165E-06
TG 58:7 FA16:0	TNF-α	-0.152606687	1.131029344	0.893570608	0.106741436	0.028668958	0.000812017
TG 58:7 FA18:0	IL-17A	0.046007995	1.377628142	0.973579594	0.001944744	0.004082099	0.637235441
TG 58:7 FA18:0	IL-1β	1.164930778	0.960403155	0.23461	0.079584872	0.027977316	0.007934992
TG 58:7 FA18:0	IL-6	1.056741003	0.753047131	0.170797575	0.020173504	0.003150116	4.53294E-07
TG 58:7 FA18:0	MCP1	0.553365127	0.748676584	0.465575671	0.003692803	0.00057967	4.9734E-07
TG 58:7 FA18:0	TNF-α	0.838424113	1.054845356	0.432955107	0.109514026	0.027647573	0.000424916
TG 58:7 FA18:1	IL-17A	0.010103272	0.476518953	0.983224717	0.001953739	0.004092041	0.636503002
TG 58:7 FA18:1	IL-1β	0.316818724	0.340274148	0.359250425	0.077755406	0.028726966	0.0111037
TG 58:7 FA18:1	IL-6	0.317142686	0.25644193	0.225791534	0.019848768	0.003108864	4.78384E-07
TG 58:7 FA18:1	MCP1	0.094402755	0.265393241	0.724547654	0.003607709	0.000595504	1.18492E-06
TG 58:7 FA18:1	TNF-α	0.142793466	0.352829123	0.688561955	0.108687861	0.026800381	0.000327792
TG 58:7 FA18:2	IL-17A	0.072090869	1.270216163	0.955143595	0.001955289	0.004080248	0.635512049
TG 58:7 FA18:2	IL-1β	1.025622806	0.928112284	0.278537745	0.071851116	0.033468005	0.040609899
TG 58:7 FA18:2	IL-6	1.201222271	0.605835612	0.057283668	0.020906878	0.0027993	3.90885E-08
TG 58:7 FA18:2	MCP1	0.521709249	0.611267238	0.40063151	0.003777886	0.000521271	6.87049E-08
TG 58:7 FA18:2	TNF-α	1.082585142	0.824852144	0.200022342	0.123124003	0.024526255	2.62511E-05
TG 58:7 FA20:4	IL-17A	-2.629817857	2.858440103	0.366355803	0.002274179	0.00396092	0.570993769
TG 58:7 FA20:4	IL-1β	-2.6077108	2.761543105	0.354056436	0.034781881	0.044129228	0.438000236
TG 58:7 FA20:4	IL-6	1.852577532	1.491378931	0.225692976	0.021768886	0.003134422	2.79652E-07
TG 58:7 FA20:4	MCP1	-0.851019738	1.762023978	0.633313689	0.00359759	0.000683635	1.89272E-05
TG 58:7 FA20:4	TNF-α	-1.469038559	1.677036733	0.389384114	0.110454715	0.021642263	2.85091E-05
TG 58:7 FA22:4	IL-17A	0.018184104	1.649909871	0.99128463	0.00194529	0.004511574	0.669640768
TG 58:7 FA22:4	IL-1β	1.058132421	1.145412745	0.36348351	0.086064898	0.032366817	0.01281159
TG 58:7 FA22:4	IL-6	1.32989152	0.827902081	0.119419867	0.020848391	0.003313737	8.38306E-07
TG 58:7 FA22:4	MCP1	0.388152563	0.798335386	0.630607387	0.003769673	0.00058918	6.31173E-07
TG 58:7 FA22:4	TNF-α	0.271070096	1.011432797	0.790659216	0.117842893	0.025585812	8.14367E-05
TG 58:7 FA22:5	IL-17A	0.022329903	0.410694813	0.957000029	0.001932201	0.004065942	0.638074757
TG 58:7 FA22:5	IL-1β	0.375325158	0.301362497	0.222614648	0.080405671	0.029331356	0.010213426
TG 58:7 FA22:5	IL-6	0.381760206	0.225271775	0.100496929	0.019740132	0.003148485	6.57667E-07
TG 58:7 FA22:5	MCP1	0.154986172	0.22839245	0.502595549	0.003668804	0.000590825	7.77163E-07
TG 58:7 FA22:5	TNF-α	0.338806111	0.306389953	0.277602225	0.111694927	0.026830774	0.000243568
TG 58:7 FA22:6	IL-17A	0.013808801	0.580513518	0.981185263	0.001937969	0.003046678	0.529704808
TG 58:7 FA22:6	IL-1β	0.33626698	0.587785493	0.571668627	0.052365737	0.033587255	0.129821412
TG 58:7 FA22:6	IL-6	0.435056926	0.41077433	0.298290026	0.019971253	0.003050603	3.59747E-07
TG 58:7 FA22:6	MCP1	0.269538954	0.389146089	0.494043886	0.003764546	0.000534317	9.48491E-08
TG 58:7 FA22:6	TNF-α	0.1632928	0.534770614	0.762279772	0.110333465	0.024823634	0.00011829
TG 58:8 FA18:2	IL-17A	0.02760014	0.940350547	0.976779119	0.001952442	0.00410237	0.637571686
TG 58:8 FA18:2	IL-1β	0.621114704	0.70267301	0.383762604	0.075361613	0.030136948	0.018088776
TG 58:8 FA18:2	IL-6	0.77652425	0.511701382	0.139600377	0.020138099	0.00315148	4.71195E-07
TG 58:8 FA18:2	MCP1	0.273328722	0.517457299	0.601236013	0.003633157	0.000589867	8.94051E-07
TG 58:8 FA18:2	TNF-α	0.463738777	0.711597355	0.519564817	0.109270023	0.027459718	0.000404204
TG 58:8 FA20:3	IL-17A	-0.007430725	3.433482384	0.998289744	0.001943796	0.004574983	0.674423344
TG 58:8 FA20:3	IL-1β	-0.995873052	2.58457592	0.703139229	0.042476126	0.039714466	0.294656696

TG 58:8 FA20:3	IL-6	2.322917734	1.487460031	0.130457348	0.02091627	0.003003265	2.1468E-07
TG 58:8 FA20:3	MCP1	0.451124642	1.701993731	0.793056016	0.003751768	0.000626413	2.53092E-06
TG 58:8 FA20:3	TNF-α	-0.1940422	1.88410962	0.918762113	0.116324426	0.02320946	3.25628E-05
TG 58:8 FA20:4	IL-17A	-0.01647255	1.521371667	0.991443781	0.001944919	0.004573033	0.674116348
TG 58:8 FA20:4	IL-1β	-0.866774681	1.221029593	0.484102037	0.043977323	0.042325299	0.308356726
TG 58:8 FA20:4	IL-6	1.10852708	0.7154128	0.133351135	0.022106428	0.003258513	3.36101E-07
TG 58:8 FA20:4	MCP1	-0.320879308	0.756337162	0.674870232	0.003700305	0.000627961	3.24932E-06
TG 58:8 FA20:4	TNF-α	-0.542608159	0.790106479	0.498318159	0.114138746	0.021956288	1.99192E-05
TG 58:8 FA22:5	IL-17A	0.013661688	0.441375397	0.975512401	0.001949208	0.004094452	0.637479881
TG 58:8 FA22:5	IL-1β	0.311645716	0.341008516	0.368059395	0.079603883	0.031099532	0.015757925
TG 58:8 FA22:5	IL-6	0.326855786	0.250793915	0.202391913	0.019888156	0.003284411	1.19464E-06
TG 58:8 FA22:5	MCP1	0.187257893	0.25209089	0.463368669	0.003673436	0.000611054	1.34966E-06
TG 58:8 FA22:5	TNF-α	0.312211832	0.339811533	0.365539525	0.109733217	0.027883192	0.000455812
TG 58:8 FA22:6	IL-17A	-0.001238286	0.187940034	0.994786611	0.001942966	0.004094644	0.63856945
TG 58:8 FA22:6	IL-1β	-0.059591056	0.165339726	0.721061495	0.074789071	0.035414004	0.043135982
TG 58:8 FA22:6	IL-6	0.040536625	0.121562959	0.741105128	0.019480053	0.003738961	1.28733E-05
TG 58:8 FA22:6	MCP1	-0.011948934	0.109955884	0.914187641	0.003688015	0.000625965	1.88777E-06
TG 58:8 FA22:6	TNF-α	-0.032029256	0.145255684	0.826973514	0.106418074	0.027992823	0.000656662
TG 58:9 FA18:1	IL-1β	-0.271336954	0.698335273	0.700354246	0.075494595	0.035275189	0.040591355
TG 58:9 FA18:1	IL-6	0.160535541	0.51980995	0.759581593	0.019504906	0.003770525	1.42924E-05
TG 58:9 FA18:1	MCP1	-0.063950997	0.470743775	0.892846596	0.003681812	0.00063201	2.27254E-06
TG 58:9 FA18:1	TNF-α	-0.156116621	0.613325351	0.800814168	0.106392165	0.027874817	0.00063015
TG 58:9 FA18:2	IL-1β	-0.061092168	0.676123755	0.928604524	0.071642443	0.03362394	0.041426712
TG 58:9 FA18:2	IL-6	0.240694017	0.516194921	0.644379614	0.019637753	0.003686278	9.24493E-06
TG 58:9 FA18:2	MCP1	-0.006134485	0.460965043	0.98947024	0.003712139	0.00060929	1.07672E-06
TG 58:9 FA18:2	TNF-α	-0.060027308	0.613586976	0.922717877	0.108711009	0.027454548	0.00042655
TG 58:9 FA20:4	IL-17A	-0.275368586	0.434173394	0.531263928	0.001977055	0.003739721	0.601358
TG 58:9 FA20:4	IL-1β	-0.375591482	0.426246778	0.386008312	0.047011126	0.042205811	0.275159495
TG 58:9 FA20:4	IL-6	-0.038707304	0.313254069	0.902574812	0.018886562	0.003963353	5.7233E-05
TG 58:9 FA20:4	MCP1	-0.131179486	0.251554382	0.606287002	0.003655923	0.000585972	1.12958E-06
TG 58:9 FA20:4	TNF-α	-0.369849928	0.258633051	0.164182682	0.111012758	0.020610032	1.08045E-05
TG 58:9 FA22:5	IL-17A	0.025609846	0.828169917	0.975535429	0.001951529	0.004109734	0.638328994
TG 58:9 FA22:5	IL-1β	0.456495481	0.619607114	0.46699734	0.072231181	0.030228156	0.023356721
TG 58:9 FA22:5	IL-6	0.495776145	0.496840511	0.326329399	0.019928837	0.00348068	3.00932E-06
TG 58:9 FA22:5	MCP1	0.397752233	0.460658402	0.394744868	0.003729347	0.000597321	7.07445E-07
TG 58:9 FA22:5	TNF-α	0.503129679	0.621162181	0.424329571	0.110107959	0.02726566	0.000343623
TG 58:9 FA22:6	IL-1β	-0.06226605	0.176461927	0.726661955	0.072899421	0.034713098	0.044237262
TG 58:9 FA22:6	IL-6	0.030802387	0.138942364	0.826055966	0.019457526	0.003924903	2.62699E-05
TG 58:9 FA22:6	MCP1	-0.021139395	0.119705379	0.861013159	0.003669625	0.000625878	2.04511E-06
TG 58:9 FA22:6	TNF-α	-0.045471597	0.159792551	0.777931459	0.106191496	0.028282294	0.000745779
TG 60:10 FA22:5	IL-17A	0.033905678	2.889971409	0.990728848	0.001949321	0.00460075	0.675271993
TG 60:10 FA22:5	IL-1β	0.848511441	2.171836906	0.699210514	0.045425253	0.039871911	0.264974588
TG 60:10 FA22:5	IL-6	1.868640582	1.487059526	0.220071158	0.020833966	0.003587212	4.04707E-06
TG 60:10 FA22:5	MCP1	2.385483805	1.361722024	0.091594888	0.003996875	0.000598786	4.41794E-07
TG 60:10 FA22:5	TNF-α	1.349447149	1.585839789	0.40257443	0.126421647	0.023339879	1.1232E-05
TG 60:10 FA22:6	IL-17A	-2.124202536	2.713800831	0.441434875	0.002161184	0.004058184	0.599241954
TG 60:10 FA22:6	IL-1β	-2.564510489	2.715248808	0.354334184	0.054534427	0.047418413	0.261446866
TG 60:10 FA22:6	IL-6	0.656054818	1.863318242	0.72784402	0.020092687	0.00433041	0.000103751
TG 60:10 FA22:6	MCP1	-0.942678206	1.683985813	0.580810418	0.003533957	0.000718131	5.07825E-05
TG 60:10 FA22:6	TNF-α	-0.910602138	1.752044215	0.608007822	0.109588741	0.024530649	0.000160908

TG 60:11 FA22:5	IL-17A	-0.154213077	4.532071757	0.973136994	0.001949392	0.005160733	0.708947192
TG 60:11 FA22:5	IL-1β	-4.570272901	3.736879857	0.233196583	0.076480311	0.049694394	0.136882872
TG 60:11 FA22:5	IL-6	0.408293708	2.723829509	0.882098531	0.019462335	0.004820397	0.000479255
TG 60:11 FA22:5	MCP1	2.80913995	2.303386708	0.234478319	0.004054526	0.000747985	1.43848E-05
TG 60:11 FA22:5	TNF-α	-0.079795433	2.633217937	0.976075688	0.114255336	0.028074476	0.000441727
TG 60:11 FA22:6	IL-17A	-1.966174886	2.064185598	0.349953443	0.002259596	0.004622558	0.629230466
TG 60:11 FA22:6	IL-1β	-1.505373714	1.914995639	0.439198126	0.055684865	0.05007563	0.276711242
TG 60:11 FA22:6	IL-6	0.466930382	1.408825239	0.74308018	0.020203141	0.00489629	0.000358063
TG 60:11 FA22:6	MCP1	-0.166506551	1.098822996	0.880772465	0.003665331	0.000692741	1.7583E-05
TG 60:11 FA22:6	TNF-α	-0.543001729	1.155697847	0.642536619	0.113731129	0.02406562	7.58213E-05
TG 60:12 FA22:6	IL-17A	-1.990844676	1.452352236	0.182628155	0.002177441	0.003998599	0.590887198
TG 60:12 FA22:6	IL-1β	-2.192070221	1.567878934	0.17436132	0.037159356	0.050436764	0.468131638
TG 60:12 FA22:6	IL-6	0.230053889	1.210045104	0.850750353	0.020425562	0.005547438	0.001115744
TG 60:12 FA22:6	MCP1	-0.394626864	0.876373532	0.656378297	0.003526863	0.000685072	2.54137E-05
TG 60:12 FA22:6	TNF-α	-0.779224575	0.93245256	0.411255396	0.111345335	0.025357657	0.000180633

n Control	n Anxiety
17	17
17	17
17	17
17	17
17	17
17	17
17	17
17	17
17	17
17	17
17	17
17	17
17	17
17	17
17	17
17	17
17	17
17	17
17	17
17	17
17	17
17	17
17	17
17	17
17	17
17	17
17	17
17	17
17	17
13	14
13	14
13	14
13	14
13	14
17	17
17	17
17	17
17	17
17	17
17	17
17	17
17	17
17	17
17	17
17	17
17	17
17	17
17	17

17	17
17	17
17	17
17	17
17	17
17	17
17	16
17	16
17	16
17	16
17	16
17	17
17	17
17	17
17	17
17	17
17	17
17	17
17	17
17	17
17	17
17	17
17	17
17	17
17	17
17	17
17	17
17	17
17	17
17	17
17	17
17	17
17	17
17	17
17	17
17	17
17	17
17	17
17	17
17	17
17	17
17	17
17	17
17	17
17	17
17	17
17	17
17	17
17	17
17	17

15	17
15	17
15	17
15	17
15	17
17	17
17	17
17	17
17	17
17	17
17	17
17	17
17	17
17	17
17	17
17	17
17	17
17	17
17	17
17	17
17	17
17	17
17	17
17	17
17	17
17	17
17	17
17	17
17	17
17	17
17	17
17	17
17	17
17	17
17	17
17	17
17	17
17	17
17	17
17	17
17	17
17	17
17	17
17	17
17	17
17	17
17	17
17	17
17	17
17	17

17	17
17	17
17	17
17	17
17	17
17	17
17	17
17	17
17	17
17	17
17	17
17	17
17	17
17	17
17	17
17	17
17	17
17	17
17	17
17	17
17	17
17	17
17	17
13	17
13	17
13	17
13	17
13	17
12	12
12	12
12	12
12	12
12	12
17	17
17	17
17	17
17	17
17	17
17	17
17	17
17	17
17	17
17	17
17	17
17	17
17	17
17	17
17	17
17	17
17	17

17	17
17	17
17	17
10	13
10	13
10	13
10	13
10	13
17	17
17	17
17	17
17	17
17	17
17	17
17	17
17	17
17	17
17	17
17	16
17	16
17	16
17	16
17	16
17	17
17	17
17	17
17	17
17	17
17	17
17	17
17	17
17	17
17	17
17	17
17	17
17	17
17	17
17	17
17	17
17	17
17	17
17	17
17	17
17	17
17	17
17	17
17	17
17	17
17	17
17	17

17	17
17	17
17	17
17	17
17	17
17	17
17	17
17	17
17	17
17	17
17	17
17	17
17	17
17	17
17	17
17	17
17	17
17	17
17	17
17	17
17	17
17	17
17	17
17	17
17	17
15	15
15	15
15	15
15	15
15	15
15	15
15	15
15	15
15	15
15	15
10	10
10	10
10	10
10	10
10	10
15	17
15	17
15	17
15	17
15	17
14	15
14	15
14	15
14	15
14	15

17	16
17	16
17	16
17	16
17	17
17	17
17	17
17	17
17	17
17	17
17	17
17	17
17	17
17	17
17	17
17	17
17	17
17	17
17	17
13	14
13	14
13	14
13	14
13	14
14	15
14	15
14	15
14	15
14	15
15	16
15	16
15	16
15	16
15	16
13	15
13	15
13	15
13	15
13	15
15	12
15	12
15	12
15	12
15	12
17	17
17	17
17	17
17	17
17	17
17	17

17	17
17	17
17	17
17	17
15	15
15	15
15	15
15	15
15	15
13	11
13	11
13	11
13	11
13	11
17	16
17	16
17	16
17	16
17	16
17	17
17	17
17	17
17	17
17	17
17	17
17	17
17	17
17	17
17	17
15	15
15	15
15	15
15	15
15	15
17	17
17	17
17	17
17	17
17	17
17	17
17	17
17	17
17	17
17	17
17	17
17	17
17	17
17	17
17	17
17	17

17	17
17	17
17	17
15	15
15	15
15	15
15	15
15	15
12	14
12	14
12	14
12	14
12	14
17	17
17	17
17	17
17	17
17	17
17	17
17	17
17	17
17	17
17	17
17	17
17	17
17	17
17	17
13	7
13	7
13	7
13	7
17	17
17	17
17	17
17	17
17	17
15	16
15	16
15	16
15	16
15	16
17	14
17	14
17	14
17	14
17	14
14	14
14	14
14	14
14	14

14	14
17	16
17	16
17	16
17	16
17	16
15	17
15	17
15	17
15	17
15	17
9	6
9	6
9	6
9	6
13	14
13	14
13	14
13	14
13	14
14	8
14	8
14	8
14	8
14	8
15	17
15	17
15	17
15	17
15	17
17	17
17	17
17	17
17	17
17	17
17	17
17	17
17	17
17	17
17	17
17	17
17	17
17	17
17	17
17	17
17	17
17	17
17	17
17	17

17	17
17	17
17	17
17	17
17	17
17	17
17	17
17	17
17	17
17	17
17	17
17	17
17	17
17	17
17	17
17	17
17	17
17	17
17	17
17	17
17	17
17	17
17	17
17	17
17	17
17	17
17	17
17	17
17	17
17	17
17	17
17	17
17	17
17	17
17	17
17	17
17	17
17	17
17	17
17	17
17	17
17	17
17	17
17	17
17	17
17	17
17	17
17	17
17	17
17	17

17	17
17	17
17	17
17	17
17	17
17	17
17	17
17	17
17	17
17	17
17	17
17	17
17	17
17	17
17	17
17	17
17	17
17	17
17	17
17	17
17	17
17	17
17	17
17	17
17	17
17	17
17	17
17	17
17	17
17	17
17	17
17	17
17	17
17	17
17	17
17	17
17	17
17	17
17	17
17	17
17	17
17	17
17	17
17	17
17	17
17	17
17	17
17	17
17	17
17	17

17	17
17	17
17	17
17	17
8	9
8	9
8	9
8	9
8	9
6	7
6	7
6	7
6	7
6	7
8	9
8	9
8	9
8	9
8	9
8	9
8	9
8	9
8	9
8	9
8	9
8	9
8	9
8	9
8	9
17	17
17	17
17	17
17	17
17	17
17	17
17	17
17	17
17	17
17	17
17	17
17	17
17	17
17	17
17	17
17	17
17	17
17	17
17	17
17	17
17	17

17	17
17	17
17	17
17	17
17	17
17	17
17	17
17	17
17	17
17	17
17	17
17	17
17	17
17	17
17	17
17	17
17	17
17	17
17	17
17	17
17	17
17	17
17	17
17	17
15	17
15	17
15	17
15	17
15	17
17	17
17	17
17	17
17	17
17	17
17	17
17	17
17	17
17	17
17	17
17	17
17	17
17	17
17	17
17	17
17	17
17	17
17	17
17	17
17	17
17	17

17	17
17	17
17	17
17	17
17	17
17	17
17	17
17	17
17	17
17	17
17	17
17	17
17	17
17	17
17	17
17	17
17	17
13	14
13	14
13	14
13	14
13	14
17	17
17	17
17	17
17	17
17	17
17	17
17	17
17	17
17	17
17	17
17	17
17	17
17	17
17	17
17	17
17	17
17	17
17	17
17	17
15	16
15	16
15	16
15	16
15	16
7	10
7	10
7	10

7	10
7	10
17	16
17	16
17	16
17	16
17	16
15	17
15	17
15	17
15	17
15	17
17	17
17	17
17	17
17	17
17	17
17	17
17	17
17	17
17	17
6	10
6	10
6	10
6	10
6	10
15	17
15	17
15	17
15	17
15	17
17	17
17	17
17	17
17	17
17	17
17	17
17	17
17	17
17	17
10	13
10	13
10	13
10	13
10	13
13	15
13	15
13	15
13	15
13	15

14	16
14	16
14	16
14	16
14	16
17	17
17	17
17	17
17	17
17	17
17	17
17	17
17	17
17	17
17	17
17	17
17	17
17	17
17	17
17	17
17	17
17	17
17	17
17	17
17	17
17	17
17	17
17	17
17	17
17	17
17	17
17	17
17	17
17	17
17	17
17	17
17	17
17	17
17	17
17	17
17	17
17	17
17	17
17	17
17	17
15	17
15	17
15	17
15	17
15	17

17	17
17	17
17	17
17	17
17	17
17	17
17	17
17	17
17	17
17	17
17	17
17	17
17	17
17	17
17	17
17	17
17	17
17	17
17	17
17	17
11	13
11	13
11	13
11	13
11	13
17	17
17	17
17	17
17	17
17	17
17	17
17	17
17	17
17	17
17	17
17	17
17	17
17	17
17	17
17	17
17	17
17	17
17	17
17	17
17	17
17	17
17	17
17	17
17	17
17	17

17	17
17	17
17	17
17	17
17	17
17	17
17	17
17	17
17	17
17	17
17	17
17	17
17	17
17	17
17	17
17	17
17	17
17	17
17	17
17	17
17	17
17	17
17	17
17	17
17	17
17	17
17	17
17	17
17	17
17	17
17	17
17	17
17	17
17	17
17	17
15	17
15	17
15	17
15	17
15	17
17	17
17	17
17	17
17	17
17	17
17	17
17	17
17	17
17	17
17	17

17	17
17	17
17	17
17	17
17	17
17	17
17	17
17	17
17	17
17	17
17	16
17	16
17	16
17	16
17	16
17	17
17	17
17	17
17	17
17	17
17	17
17	17
17	17
17	17
17	17
17	17
17	17
17	17
17	17
17	17
17	17
17	17
17	17
17	17
17	17
17	17
17	17
17	17
17	17
17	17
17	17
17	17
17	17
17	17
17	17
17	17
17	17
17	17
17	17
17	17

17	17
17	17
17	17
17	17
17	17
17	17
17	17
17	17
17	17
17	17
17	17
17	17
17	17
17	17
17	17
17	17
17	17
17	17
17	17
17	17
17	17
17	17
17	17
17	17
17	17
17	17
17	17
17	17
17	17
17	17
17	17
17	17
17	17
17	17
17	17
17	17
17	17
17	17
17	17
17	17
17	17
17	17
17	17
17	17
17	17
17	17
17	17
17	17
17	17
17	17

17	17
17	17
17	17
17	17
17	17
17	17
17	17
17	17
17	17
17	17
17	17
17	17
17	17
17	17
17	17
17	17
17	17
17	17
17	17
17	17
17	17
17	17
17	17
17	17
17	17
17	17
17	17
17	17
17	17
17	17
17	17
17	17
17	17
17	17
17	17
15	15
15	15
15	15
15	15
15	15
15	17
15	17
15	17
15	17
15	17
17	17
17	17
17	17
17	17
17	17

17	17
17	17
17	17
17	17
17	17
17	17
17	17
17	17
17	17
17	17
17	17
17	17
17	17
17	17
17	17
17	17
17	17
17	17
17	17
17	17
14	13
14	13
14	13
14	13
14	13
17	16
17	16
17	16
17	16
17	16
17	17
17	17
17	17
17	17
17	17
17	17
17	17
17	17
17	17
17	17
17	17
17	17
17	17
17	17
17	17
17	17
17	17
17	17
17	17
17	17

17	17
17	17
17	17
17	17
17	17
17	17
17	17
17	17
17	17
17	17
17	17
17	17
17	17
17	17
17	17
17	17
17	17
17	17
17	17
17	17
17	17
17	17
17	17
17	17
17	17
17	17
17	17
17	17
17	17
17	17
17	17
17	17
17	17
17	17
17	17
17	17
17	17
17	17
17	17
17	17
17	17
17	17
17	17
17	17
17	17
17	17
17	17
17	17
17	17
17	17

17	17
17	17
17	17
17	17
17	17
17	17
17	17
17	17
17	17
17	17
17	17
17	17
17	17
17	17
17	17
17	17
17	17
17	17
17	17
17	17
17	17
17	17
17	17
17	17
17	17
17	17
17	17
17	17
17	17
17	17
17	17
17	17
17	17
17	17
17	17
17	17
17	17
17	17
17	17
17	17
17	17
17	17
17	17
17	17
17	17
17	17
17	17
17	17
17	17
17	17

17	17
17	17
17	17
17	17
17	17
17	17
17	17
17	17
17	17
17	17
17	17
17	17
17	17
17	17
17	17
17	17
17	17
17	17
17	17
17	17
17	17
17	17
17	17
17	17
17	17
17	17
17	17
17	17
17	17
17	17
17	17
17	17
17	17
17	17
17	17
17	17
17	17
17	17
17	17
17	17
17	17
17	17
17	17
17	17
17	17
17	17
17	17
17	17
17	17
17	17

17	17
17	17
17	17
17	17
17	17
17	17
17	17
17	17
17	17
15	15
15	15
15	15
15	15
15	15
17	17
17	17
17	17
17	17
17	17
17	17
17	17
17	17
17	17
17	17
17	17
17	17
17	17
17	17
17	17
17	17
17	17
17	17
17	17
17	16
17	16
17	16
17	16
17	16
14	16
14	16
14	16
14	16
14	16
17	17
17	17
17	17
17	17
17	17
17	17

17	17
17	17
17	17
17	17
17	17
17	17
17	17
17	17
17	16
17	16
17	16
17	16
17	16
17	17
17	17
17	17
17	17
17	17
17	16
17	16
17	16
17	16
17	16
12	12
12	12
12	12
12	12
15	15
15	15
15	15
15	15
15	15
15	15
15	15
15	15
15	15
15	15
17	17
17	17
17	17
17	17
17	17
17	17
17	17
17	17
17	17
17	17
17	17
17	17
17	17

17	17
17	17
17	17
17	17
17	17
17	17
17	17
17	17
17	17
17	17
17	17
17	17
17	17
17	17
17	17
17	17
17	17
17	17
17	17
17	17
17	17
17	17
17	17
17	17
17	17
17	17
17	17
17	17
17	17
17	17
17	17
17	17
17	17
17	17
17	17
17	17
17	17
17	17
17	17
17	17
17	17
17	17
17	17
17	17
17	17
17	17
17	17
17	17
17	17
17	17

17	17
17	17
17	17
17	17
17	17
17	17
17	17
17	17
17	17
17	17
17	17
17	17
17	17
17	17
17	17
17	17
17	17
17	17
17	17
17	17
17	17
17	17
15	17
15	17
15	17
15	17
15	17
17	17
17	17
17	17
17	17
17	17
17	17
17	17
17	17
17	17
17	17
17	17
17	17
17	17
17	17
17	17
15	15
15	15
15	15
15	15
15	15
17	17
17	17
17	17

17	17
17	17
17	17
17	17
17	17
17	17
17	17
17	17
17	17
17	17
17	17
17	17
17	17
17	17
17	17
17	17
17	17
17	17
17	17
17	17
17	17
17	17
17	17
17	17
17	17
17	17
17	17
17	17
17	17
17	17
17	17
17	17
17	17
17	17
17	17
17	17
17	17
17	17
17	17
17	17
17	17
17	17
17	17
17	17
17	17
17	17
17	17
17	17
17	17
17	17

17	17
17	17
17	17
17	17
17	17
17	17
17	17
17	17
17	17
17	17
17	17
17	17
17	17
17	17
17	17
17	17
17	17
17	17
17	17
17	17
17	17
17	17
17	17
17	17
17	17
17	17
17	17
17	17
17	17
17	17
17	17
17	17
17	17
17	17
17	17
17	17
17	17
17	17
17	17
17	17
17	17
17	17
17	17
17	17
17	17
17	17
17	17
17	17
17	17
17	17

17	17
17	17
15	16
15	16
15	16
15	16
15	16
17	17
17	17
17	17
17	17
17	17
17	17
17	17
17	17
17	17
17	17
17	17
17	17
17	17
17	17
15	17
15	17
15	17
15	17
17	17
17	17
17	17
17	17
17	17
17	16
17	16
17	16
17	16
17	16
17	17
17	17
17	17
17	17
17	17
17	17
17	17
17	17
17	17
17	17
17	17
17	17
17	17
17	17
17	17

17	17
17	17
17	17
17	17
17	17
17	17
17	17
17	17
17	17
17	17
14	15
14	15
14	15
14	15
14	15
15	15
15	15
15	15
15	15
15	15
17	17
17	17
17	17
17	17
17	17
17	17
17	17
17	17
17	17
17	17
17	17
17	17
17	17
17	17
17	17
17	17
17	17
17	17
17	17
17	17
17	17
17	17
17	17
17	17
17	17
15	14
15	14
15	14
15	14
15	14

17	16
17	16
17	16
17	16
17	16
14	14
14	14
14	14
14	14
14	14
17	17
17	17
17	17
17	17
17	17
17	17
17	17
17	17
17	17
17	17
17	17
17	17
17	17
17	17
17	17
17	17
17	17
17	17
17	17
17	17
17	17
17	17
17	17
17	17
17	17
17	17
17	17
17	17
17	17
17	17
17	17
17	17
17	17
17	17
17	17
17	17
17	17
17	17
17	17
17	17

17	17
17	17
17	17
17	17
17	17
17	17
17	17
17	17
17	17
17	17
17	17
17	17
17	17
17	17
17	17
17	17
17	17
17	17
17	17
17	17
17	17
17	17
17	17
17	17
17	17
17	17
17	17
17	17
17	17
17	17
17	17
17	17
17	17
17	17
17	17
17	17
17	17
17	17
17	17
17	17
17	17
17	17
17	17
17	17
17	17
17	17
17	17
17	17
17	17
17	17

15	16
15	16
15	16
15	16
15	16
17	17
17	17
17	17
17	17
17	17
17	17
17	17
17	17
17	17
17	17
17	17
17	17
17	17
17	17
17	17
15	15
15	15
15	15
15	15
15	15
15	14
15	14
15	14
15	14
15	14
15	15
15	15
15	15
15	15
15	15
14	15
14	15
14	15
14	15
14	15
17	17
17	17
17	17
17	17
17	17
17	17
17	17
17	17
17	17
17	17

17	17
17	17
17	17
17	17
17	17
17	17
17	17
17	17
17	17
17	17
17	17
17	17
17	17
17	17
17	17
17	17
17	17
17	17
17	17
17	17
17	17
17	17
17	17
17	17
17	17
17	17
17	17
17	17
17	17
17	17
17	17
17	17
17	17
17	17
17	17
17	17
17	17
17	17
17	17
17	17
17	17
17	17
17	17
17	17
17	17
17	17
17	17
17	17
17	17
17	17

17	17
17	17
17	17
17	17
17	17
17	17
17	17
17	17
17	17
17	17
17	17
17	17
17	17
17	17
17	17
17	17
17	17
17	17
17	17
17	17
17	17
17	17
17	17
17	17
17	17
17	17
17	17
17	17
17	17
17	17
17	17
17	17
17	17
17	17
17	17
17	17
17	17
17	17
17	17
17	17
17	17
17	17
17	17
17	17
17	17
17	17
17	17
17	17
17	17
17	17

17	17
17	17
17	17
17	17
17	17
17	17
17	17
17	17
17	17
17	17
17	17
17	17
17	17
17	17
17	17
17	17
17	17
17	17
17	17
17	17
17	17
17	17
17	17
17	17
17	17
17	17
17	17
17	17
17	17
17	17
17	17
17	17
17	17
17	17
17	17
15	16
15	16
15	16
15	16
15	16
17	16
17	16
17	16
17	16
17	16
17	17
17	17
17	17
17	17
17	17

17	17
17	17
17	17
17	17
17	17
17	16
17	16
17	16
17	16
17	16
17	17
17	17
17	17
17	17
17	17
17	17
17	17
17	17
17	17
17	17
17	17
17	17
17	17
17	17
17	17
17	16
17	16
17	16
17	16
17	16
17	16
17	16
17	16
17	16
17	16
17	17
17	17
17	17
17	17
17	17
17	17
17	17
17	17
17	17
17	17
14	15
14	15
14	15
14	15
14	15

14	17
14	17
14	17
14	17
14	17
15	15
15	15
15	15
15	15
15	15
17	17
17	17
17	17
17	17
17	17
17	17
17	17
17	17
17	17
17	17
17	17
17	17
17	17
17	17
17	17
17	17
17	17
17	17
17	17
17	17
17	17
17	17
17	17
17	17
17	17
17	16
17	16
17	16
17	16
17	16
17	17
17	17
17	17
17	17
17	17
17	17
17	17
17	17
17	17
17	17

17	17
17	17
17	17
17	17
17	17
15	14
15	14
15	14
15	14
15	14
17	17
17	17
17	17
17	17
17	17
17	17
17	17
17	17
17	17
17	17
17	17
17	17
17	17
17	17
17	17
17	17
17	17
17	17
17	17
17	17
15	16
15	16
15	16
15	16
15	16
17	17
17	17
17	17
17	17
17	17
15	17
15	17
15	17
15	17
15	17
17	17
17	17
17	17
17	17
17	17

6	6
6	6
6	6
6	6
6	6
14	14
14	14
14	14
14	14
14	14
15	15
15	15
15	15
15	15
15	15
17	17
17	17
17	17
17	17
17	17
12	15
12	15
12	15
12	15
12	15
17	17
17	17
17	17
17	17
17	17
17	17
17	17
17	17
17	17
17	17
17	17
17	17
17	17
17	17
17	17
17	17
17	17
17	17
17	17
17	17
17	17
17	17
17	17
17	17

17	17
17	17
17	17
17	17
17	17
17	17
17	17
17	17
17	17
17	17
17	17
17	17
17	17
17	17
17	17
17	17
17	17
17	17
17	17
17	17
17	17
17	17
17	17
17	17
17	17
17	17
17	17
17	17
17	17
17	17
17	17
17	17
17	17
17	17
17	17
17	17
17	17
17	17
17	17
17	17
17	17
17	17
17	17
17	17
17	17
17	17
17	17
17	17
17	17
17	17

17	15
17	15
17	15
17	15
17	15
15	15
15	15
15	15
15	15
15	15
15	15
15	15
15	15
15	15
15	15
14	14
14	14
14	14
14	14
14	14
17	17
17	17
17	17
17	17
17	17
17	17
17	17
17	17
17	17
17	17
17	17
17	17
17	17
17	17
17	17
17	17
17	17
17	17
17	17
17	17
17	17
17	17
17	17
17	17
17	17
17	17
17	17
17	17
17	17

17	17
17	17
17	17
17	17
17	17
17	17
17	17
17	17
17	17
17	17
17	17
17	17
17	17
17	17
17	17
17	17
17	17
17	17
17	17
17	17
17	17
17	17
17	17
17	17
17	17
17	17
17	17
17	17
17	17
17	17
17	17
17	17
17	17
17	17
17	17
17	17
17	17
17	17
17	17
17	17
17	17
17	17
17	17
17	17
17	17
17	17
17	17
17	17
17	17
17	17

17	17
17	17
17	17
17	17
17	17
17	17
17	17
17	17
17	17
17	17
17	17
17	17
17	17
17	17
17	17
17	17
17	17
17	17
17	17
17	17
17	17
17	17
17	17
17	17
17	17
17	17
17	17
17	17
17	17
17	17
17	17
17	17
17	17
17	17
17	17
17	17
17	17
17	17
17	17
17	17
17	17
17	17
17	17
17	17
17	17
17	17
17	17
17	17
17	17
17	17

17	17
17	17
17	17
17	17
17	17
17	17
17	17
17	17
17	17
17	17
17	17
17	17
17	17
17	17
17	17
17	16
17	16
17	16
17	16
17	16
17	17
17	17
17	17
17	17
17	17
17	17
17	17
17	17
17	17
17	17
17	17
17	17
17	17
17	17
17	15
17	15
17	15
17	15
17	15
15	17
15	17
15	17
15	17
15	17
17	17
17	17
17	17
17	17
17	17
17	17

17	17
17	17
17	17
17	17
17	17
17	17
17	17
17	17
17	17
17	17
17	17
17	17
17	17
17	17
17	17
17	17
17	17
17	17
17	17
17	17
17	17
17	17
17	17
17	17
17	17
17	17
17	17
17	17
17	17
17	17
17	17
17	17
17	17
17	17
17	17
17	17
17	17
17	17
17	17
17	17
17	17
17	17
17	17
17	17
17	17
17	17
17	17
17	17
17	17
15	17

15	17
15	17
15	17
15	17
17	17
17	17
17	17
17	17
17	17
17	17
17	17
17	17
17	17
17	17
17	16
17	16
17	16
17	16
17	16
17	17
17	17
17	17
17	17
17	17
17	17
17	17
17	17
17	17
17	17
17	17
17	17
17	17
17	17
17	17
17	17
17	17
17	17
17	17
17	17
17	17
17	17
17	17
17	17
17	17
17	17
17	17
17	17
17	17
17	17
17	17

17	17
17	17
17	17
17	17
17	17
17	17
17	17
17	17
17	17
15	17
15	17
15	17
15	17
15	17
17	17
17	17
17	17
17	17
17	17
17	17
17	17
17	17
17	17
17	17
17	17
17	17
17	17
17	17
17	17
17	17
17	17
17	17
17	17
15	16
15	16
15	16
15	16
15	16
17	17
17	17
17	17
17	17
17	17
17	17
17	17
17	17
17	17
17	17
17	17
17	17

17	17
17	17
17	17
14	15
14	15
14	15
14	15
14	15
13	15
13	15
13	15
13	15
13	15
15	15
15	15
15	15
15	15
15	15
17	17
17	17
17	17
17	17
17	17
17	17
17	17
17	17
17	17
17	17
17	17
17	17
17	17
17	17
17	17
17	17
17	17
17	17
17	17
17	17
17	17
17	17
17	17
17	17
17	17
17	17
17	17
17	17
17	17
17	17
17	17
17	17

17	17
17	17
17	17
17	17
17	17
17	17
17	17
17	17
17	16
17	16
17	16
17	16
17	16
17	17
17	17
17	17
17	17
17	17
15	15
15	15
15	15
15	15
15	15
17	17
17	17
17	17
17	17
14	17
14	17
14	17
14	17
14	17
17	17
17	17
17	17
17	17
17	17
17	17
17	17
17	17
17	17
17	17
17	17
17	17
17	17
17	17
17	17
17	17
17	17

17	17
17	17
17	17
17	17
17	17
17	17
17	17
17	17
17	17
17	17
17	17
17	17
17	17
17	17
17	17
17	17
17	17
15	15
15	15
15	15
15	15
15	15
17	17
17	17
17	17
17	17
17	17
17	17
17	17
17	17
17	17
17	17
17	17
17	17
17	17
17	17
17	17
17	16
17	16
17	16
17	16
17	16
17	17
17	17
17	17
17	17
17	17
15	15
15	15
15	15

15	15
15	15
15	17
15	17
15	17
15	17
15	17
17	17
17	17
17	17
17	17
17	17
17	17
17	17
17	17
17	17
17	17
17	17
17	17
17	17
17	17
17	17
17	17
17	17
17	17
17	17
17	17
17	17
17	17
17	17
17	17
17	17
17	16
17	16
17	16
17	16
17	16
17	17
17	17
17	17
17	17
17	17
14	16
14	16
14	16
14	16
14	16
17	17
17	17
17	17

17	17
17	17
17	17
17	17
17	17
17	17
17	17
17	17
17	17
17	17
17	17
17	17
17	17
17	17
17	17
17	17
17	17
17	17
17	17
17	17
17	17
17	17
17	17
17	17
17	17
17	17
17	17
17	17
17	17
17	17
17	17
17	17
17	17
17	17
17	17
17	17
17	17
17	17
17	17
17	17
17	17
17	17
17	17
17	17
17	17
17	17
17	17
17	17
17	17
17	17

17	17
17	17
13	15
13	15
13	15
13	15
13	15
17	16
17	16
17	16
17	16
17	16
17	17
17	17
17	17
17	17
17	17
14	16
14	16
14	16
14	16
14	16
17	17
17	17
17	17
17	17
17	17
17	17
17	17
17	17
17	17
17	17
17	17
17	17
17	17
17	17
17	17
17	17
17	17
17	17
17	17
17	17
17	17
17	17
17	17
17	17
17	17
17	17
17	17
17	17

17	17
17	17
17	17
17	17
17	17
17	17
17	17
17	17
17	17
17	17
17	17
17	17
17	17
17	17
17	17
17	17
17	17
17	17
17	17
17	17
17	17
17	17
17	17
17	17
17	17
17	17
17	17
17	17
17	17
17	17
17	17
14	15
14	15
14	15
14	15
14	15
17	17
17	17
17	17
17	17
17	17
17	17
17	17
17	17
17	17
17	17
17	17
17	17
17	17
17	17

15	15
15	15
15	15
15	15
15	15
17	17
17	17
17	17
17	17
17	17
17	17
17	17
17	17
17	17
17	17
17	17
17	17
17	17
17	17
17	17
17	17
17	17
17	17
17	17
17	17
17	17
17	17
17	17
17	17
17	17
15	17
15	17
15	17
15	17
15	17
17	17
17	17
17	17
17	17
17	17
17	17
17	17
17	17
17	17
15	17
15	17
15	17
15	17
15	17
17	17

17	17
17	17
17	17
17	17
14	15
14	15
14	15
14	15
14	15
17	17
17	17
17	17
17	17
17	17
15	17
15	17
15	17
15	17
15	17
17	17
17	17
17	17
17	17
17	17
17	17
17	17
17	17
17	17
17	17
17	17
17	17
17	17
17	17
15	16
15	16
15	16
15	16
15	16
15	15
15	15
15	15
15	15
15	15
13	14
13	14
13	14
13	14
13	14
15	17
15	17

15	17
15	17
15	17
17	17
17	17
17	17
17	17
17	17
17	17
17	17
17	17
17	17
17	17
17	17
17	17
17	17
17	17
17	17
17	16
17	16
17	16
17	16
17	16
17	17
17	17
17	17
17	17
17	17
13	15
13	15
13	15
13	15
13	15
13	15
13	15
13	15
13	15
13	15
14	15
14	15
14	15
14	15
14	15
17	17
17	17
17	17
17	17
17	17
17	17
17	17

17	17
17	17
17	17
17	17
17	17
17	17
17	17
17	17
17	17
17	17
17	17
17	17
17	17
17	17
17	17
17	17
17	17
17	17
17	15
17	15
17	15
17	15
17	15
13	15
13	15
13	15
13	15
13	15
15	16
15	16
15	16
15	16
15	16
17	17
17	17
17	17
17	17
17	17
17	16
17	16
17	16
17	16
17	16
17	17
17	17
17	17
17	17
17	17
14	15
14	15

14	15
14	15
14	15
14	15
14	15
14	15
14	15
14	15
17	17
17	17
17	17
17	17
17	17
17	17
17	17
17	17
17	17
17	17
17	17
17	17
17	17
17	17
17	17
17	17
17	17
17	17
15	15
15	15
15	15
15	15
15	15
17	17
17	17
17	17
17	17
17	17
17	17
17	17
17	17
17	17
14	15
14	15
14	15
14	15
14	15
13	14
13	14
13	14
13	14
13	14

13	14
13	14
13	14
13	14
13	14
14	14
14	14
14	14
14	14
14	14
14	14
14	14
14	14
14	14
14	14
